# Silencing FLI or targeting CD13/ANPEP lead to dephosphorylation of EPHA2, a mediator of BRAF inhibitor resistance, and induce growth arrest or apoptosis in melanoma cells

**DOI:** 10.1038/cddis.2017.406

**Published:** 2017-08-31

**Authors:** Alireza Azimi, Rainer Tuominen, Fernanda Costa Svedman, Stefano Caramuta, Maria Pernemalm, Marianne Frostvik Stolt, Lena Kanter, Pedram Kharaziha, Janne Lehtiö, Carolina Hertzman Johansson, Veronica Höiom, Johan Hansson, Suzanne Egyhazi Brage

**Affiliations:** 1 Cancer Center Karolinska, Department of Oncology and Pathology, Karolinska Institutet, Stockholm, Sweden; 2 Science for Life Laboratory, Department of Oncology and Pathology, Karolinska Institutet, Stockholm, Sweden; 3 Department of Physiology and Pharmacology, Karolinska Institutet, Stockholm, Sweden

## Abstract

A majority of patients with BRAF-mutated metastatic melanoma respond to therapy with BRAF inhibitors (BRAFi), but relapses are common owing to acquired resistance. To unravel BRAFi resistance mechanisms we have performed gene expression and mass spectrometry based proteome profiling of the sensitive parental A375 BRAF V600E-mutated human melanoma cell line and of daughter cell lines with induced BRAFi resistance. Increased expression of two novel resistance candidates, aminopeptidase-*N* (CD13/ANPEP) and ETS transcription factor FLI1 was observed in the BRAFi-resistant daughter cell lines. In addition, increased levels of the previously reported resistance mediators, receptor tyrosine kinase ephrine receptor A2 (EPHA2) and the hepatocyte growth factor receptor MET were also identified. The expression of these proteins was assessed in matched tumor samples from melanoma patients obtained before BRAFi and after disease progression. MET was overexpressed in all progression samples while the expression of the other candidates varied between the individual patients. Targeting CD13/ANPEP by a blocking antibody induced apoptosis in both parental A375- and BRAFi-resistant daughter cells as well as in melanoma cells with intrinsic BRAFi resistance and led to dephosphorylation of EPHA2 on S897, previously demonstrated to cause inhibition of the migratory capacity. AKT and RSK, both reported to induce EPHA2 S897 phosphorylation, were also dephosphorylated after inhibition of CD13/ANPEP. FLI1 silencing also caused decreases in EPHA2 S897 phosphorylation and in total MET protein expression. In addition, silencing of FLI1 sensitized the resistant cells to BRAFi. Furthermore, we show that BRAFi in combination with the multi kinase inhibitor dasatinib can abrogate BRAFi resistance and decrease both EPHA2 S897 phosphorylation and total FLI1 protein expression. This is the first report presenting CD13/ANPEP and FLI1 as important mediators of resistance to BRAF inhibition with potential as drug targets in BRAFi refractory melanoma.

Cytotoxic chemotherapy in disseminated cutaneous malignant melanoma (CMM) results in a low proportion of clinical responses and no improved survival.^1^ However, during the last years, novel targeted therapies have been introduced and opened up the possibility for successful development of personalized medicine. Treatment of disseminated CMM-carrying activating BRAF mutations (V600E/K) with inhibitors targeting the mitogen-activated protein kinase (MAPK) signaling pathway, either as single agent treatment with BRAF inhibitor ((BRAFi) dabrafenib or vemurafenib) or in combination with MEK inhibitor ((MEKi) trametinib) significantly prolongs overall survival in patients with BRAF-mutated CMM.^2, 3, 4, 5^ Still, remissions with these agents are often not durable and research aimed at improving existing therapies by identifying predictive factors for long response and at reversing both intrinsic and acquired resistance to targeted therapies has a high priority.

Investigations of the underlying mechanisms of resistance to BRAFi have led to identification of several genetic alterations^6^ including *BRAF* splice variants,^7^ amplification of *BRAF*,^8^ secondary activating mutations in *NRAS*, *KRAS* and *MEK*, and *PTEN* deletions.^9, 10^

In addition, proteome and phosphoproteome alterations contributing to drug resistance have been reported in cancer cells. Overexpression of a number of receptor tyrosine kinases (RTKs) such as PDGFR*β*, EGFR, MET and recently ephrine receptor A2 (EPHA2) have been associated with acquired BRAFi resistance.^11, 12, 13, 14, 15, 16, 17, 18^ Miao *et al.*^11^ have recently shown that EPHA2 S897 phosphorylation is increased in vemurafenib-resistant melanoma cells and also demonstrated that EPHA2 can be a potential novel therapeutic target. Further studies are required to investigate other contributing and co-occurring mechanisms of resistance to BRAFi that can be used as either predictive biomarkers and/or druggable targets.

To identify alterations of mRNA and protein expression associated with acquired BRAFi resistance we performed qPCR and mass spectrometry based protein profiling of a parental BRAFi-sensitive A375 human melanoma cell line and three daughter cell lines with induced BRAFi resistance.

In the mass spectrometry based protein profiling we identified two novel resistance candidates, aminopeptidase-N (CD13/ANPEP) and ETS transcription factor FLI1, which are associated with resistance to vemurafenib and also confirmed a number of recently reported druggable resistance mediators such as MET, EGFR and EPHA2. In addition, we could demonstrate that targeting CD13/ANPEP or silencing FLI1 decreased EPHA2 S897 phosphorylation, which has previously been shown to lead to inhibition of cell migration.

## Results

### The MAPK pathway was reactivated in the BRAFi-resistant melanoma cells

**Figure 1:**
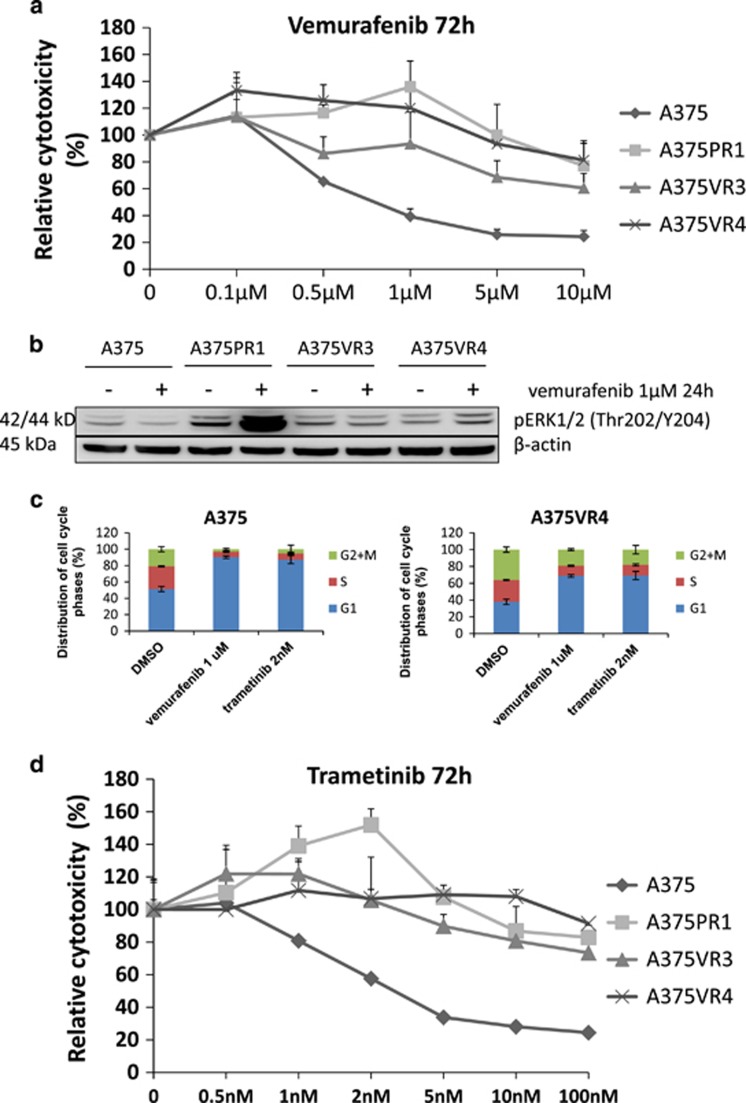
Melanoma cells with cross resistance to BRAFi vemurafenib and the MEKi trametinib show MAPK pathway reactivation and less G1 cell cycle arrest after vemurafenib and trametinib treatment. (**a**) Dose–response curves after 72 h exposure to vemurafenib in parental A375 and three BRAFi-resistant sublines, showing cytoxic effect as % of DMSO control. (Error bars represent S.D. of the mean of three independent MTS experiments). Parental A375 cells had an IC50 of <1 *μ*M for vemurafenib, evaluated by MTS colorimetric assay. In contrast, IC50 was not reached in the resistant sublines by treatment with vemurafenib with concentrations up to 10 *μ*M. A low concentration of BRAFi, 1 *μ*M for A375PR1 and 0.1 *μ*M for A375VR4, significantly induced proliferation (*P*<0.05). (**b**) Protein expression levels of pERK analyzed by immunoblotting after 24 h exposure to 1 *μ*M of vemurafenib (or DMSO as control treatment) demonstrated reactivation of the MAPK pathway in the resistant cell lines. (**c**) The cell cycle distribution effect in parental A375 and A375VR4 after 24 h treatment with 1 *μ*M vemurafenib or 2 nM trametinib (Error bars represent S.D. of the mean of 3–6 replicates). (**d**) Dose–response curves after 72 h exposure to trametinib in parental A375 and three resistant sublines. (Error bars represent S.D. of the mean of three independent MTS experiments). In addition, a significant induction of proliferation of A375PR1 was also observed after treatment with 2 nM trametinib (*P*<0.05)

To investigate underlying mechanisms of acquired resistance to BRAFi we induced resistance by repeatedly exposing the parental A375 BRAF V600E mutated human melanoma cell line to increasing concentrations of the BRAFi PLX4720 or the clinically used vemurafenib. Three stably resistant daughter cell lines were obtained and characterized: A375-PLX4720R1 (A375PR1), A375-vemurafenibR3 (A375VR3) and A375-vemurafenibR4 (A375VR4) (Figure 1a). We determined whether reactivation of the MAPK pathway contributes to BRAFi resistance in these resistant cells by analyzing ERK phosphorylation after vemurafenib treatment in sensitive and resistant sublines (Figure 1b). The MAPK pathway was reactivated when analyzing ERK phosphorylation after 24 h treatment with 1 *μ*M vemurafenib, especially in A375PR1 supporting the MTS results. We have also analyzed effects on cell cycle distribution in A375 and A375VR4 after 24 h treatment with 1 *μ*M vemurafenib. As expected, the G1 cell cycle block and reduction of S-phase cells was more pronounced in the sensitive parental cells than in the vemurafenib-resistant cells (Figure 1c). These results are consistent with lack of inhibition of proliferation at 1 *μ*M in the resistant A375VR4 cells. Vemurafenib induced more apoptosis in A375 than in A375VR4, in agreement with the results from the MTS assay (Supplementary Figure S1). The resistant cells were also cross-resistant to the MEKi trametinib (Figure 1d) with a similarly reduced effect on cell cycle distribution, compared with parental cells (Figure 1c).

### MAPK reactivation was not due to secondary BRAF, NRAS or MEK mutations in BRAFi-resistant melanoma cells

To investigate if the MAPK pathway reactivation could be due to presence of secondary mutations in BRAFi-resistant sublines, mutational analysis of MAPK signaling-related genes including *BRAF*, *NRAS* and *MEK* was performed using targeted next-generation sequencing. The expected mutation pattern was evidenced by the sequence data, whereas no secondary mutations of particular interest was detected. For more information see supplementary data.

### Targeted MAPK pathway mRNA array confirmed transcriptional changes associated with BRAFi resistance

MAPK pathway qPCR array analysis was performed to investigate whether there were any differences in basal mRNA levels for components of the MAPK signaling between parental A375 and the BRAFi-resistant sublines. Table 1 shows log2 fold changes of mRNA in the resistant daughter cell lines compared with the parental A375 cell line for a number of key factors of the MAPK pathway. With a cutoff of at least a log2 fold change of 1.0 BRAF and NRAS were not altered at the mRNA level. However, a log2 fold change of 1.0 or higher elevation in gene expression of a number of genes including *EGFR* and *RAF1*, and genes involved in cell cycle regulation such as CCND1 was observed in the vemurafenib-resistant cell lines confirming previous reports.

### In-depth proteome profiling identified novel potential BRAFi resistance candidates – CD13/ANPEP and FLI1

Mass spectrometry based proteome analysis was performed on parental A375 and the BRAFi-resistant sublines to detect novel candidates associated with BRAFi resistance. The analysis resulted in the detection of over 7700 proteins, and 5865 of these were detected in all samples (the raw data have been uploaded to proteome Xchange accession PXD001682). No significant outliers in replicate analyses were detected using principal component analyses (Supplementary Figure S2). Paired comparison of parental A375 cells with each of the BRAFi-resistant sublines was performed using SAM analysis to identify significantly differentially expressed proteins, with a false discovery rate of 0.5% (Supplementary Table S2). In total 678, 229 and 616 proteins were upregulated in A375PR1, A375VR3 and A375VR4, respectively. Of these proteins, 49 were overexpressed in all three resistant sublines while 156 proteins were only altered in A375VR3 and A375VR4 as shown in the Venn diagram (Supplementary Figure S3). None of the proteins were consistently downregulated in the three resistant cell lines (Supplementary Figure S4). The differentially expressed proteins are correlated to 28 protein classes, as shown by PANTHER classification system (Supplementary Table S3).

Among the significantly differentially expressed proteins we focused on two novel potential candidates; CD13/ANPEP and FLI1, both with markedly higher protein expression in the BRAFi-resistant cells, and also on the recently reported BRAFi resistance mediator EPHA2 (Table 2).^11^ In addition, a number of known BRAFi resistance regulators as well as stemness-related proteins such as SOX10, MITF and CD166 were altered in the proteomics analysis (Table 2). Similar to previous reports PTEN was found to be downregulated and higher levels of CCND1, EGFR and MET were observed (Table 2).^6, 10, 12^ These candidates also have corresponding mRNA alterations in most of the cases in the BRAFi-resistant sublines as detected by qPCR and MAPK pathway arrays (Tables 1 and 2).

### Vemurafenib-resistant cells have higher proliferation and migration

We studied if there were any differences in growth characteristics and morphology between parental A375 and resistant sublines. The vemurafenib-resistant A375VR4 cell line had a higher proliferation rate than parental A375 and PLX4720-resistant A375PR1 cells and reached sub-confluent state already within 72 h after seeding (Supplementary Figure S5). In addition, A375VR4 was more migratory compared with the parental A375 and A375PR1 cell lines and filled the gap in a scratch assay within 24 h (Supplementary Figure S6). Interestingly, the cellular morphology also differs; A375VR4 cells are cuboidal, whereas the others are more spindle shaped (Supplementary Figure S5). EPHA2 is shown to induce mesenchymal to amoeboidal-like transition in melanoma cells and enhance the invasiveness of cells through increased migratory capacity.^19^ The cuboidal morphology together with EPHA2 overexpression suggests transition from a mesenchymal to an ameboid-like phenotype. In addition, alterations in levels of stemness-related proteins such as low SOX10, low MITF and high CD166 have also been associated with invasive type (Table 2,Supplementary Figure S7).

The overlap in proteome features between the vemurafenib-resistant sublines A375VR3 and A375VR4 as presented in the Venn diagrams (supplementary Figures S3 and S4) led us to focus on A375VR4 cells, as they display higher vemurafenib/trametinib resistance than A375VR3 (Figures 1a and c).

### Targeting the novel potential BRAFi resistance candidate CD13/ANPEP leads to dephosphorylation of EPHA2 on S897 and induces apoptosis in melanoma cells

**Figure 2:**
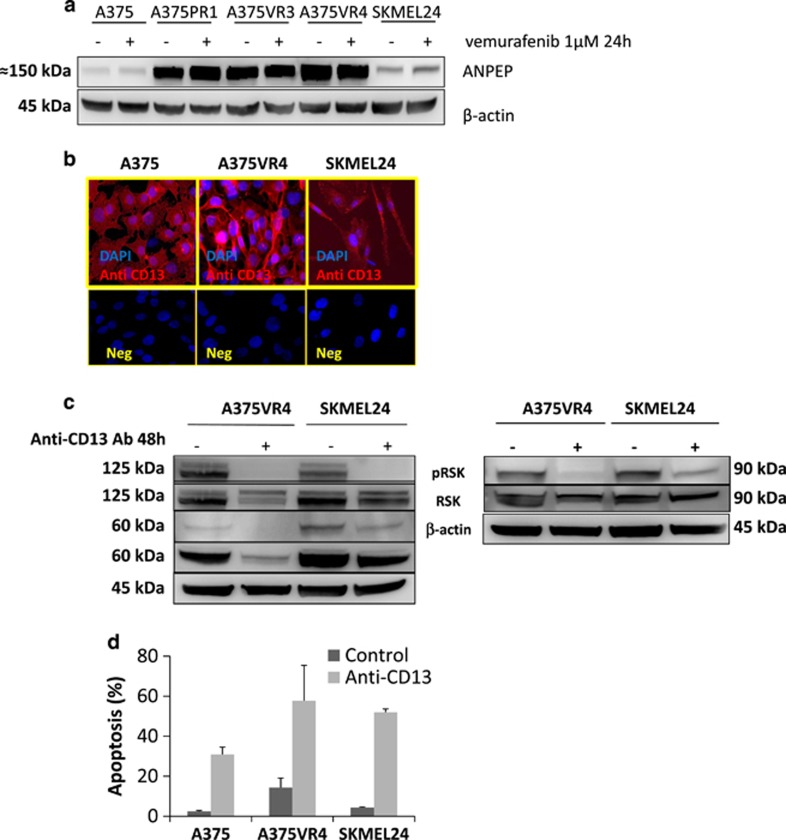
Potential BRAFi resistance candidates. Blocking of CD13/ANPEP leads to dephosphorylation of EPHA2 on S897 and increased apoptosis. (**a**) Protein expression levels of the potential resistance candidate protein CD13/ANPEP analyzed by immunoblotting in presence of 1 *μ*M of vemurafenib or DMSO as control treatment for 24 h in parental A375 and three BRAFi-resistant sublines. *β*-actin was used as control. (**b**) Immunofluoroscence showing CD13 expression in A375, A375VR4 and SKMEL24 cells. CD13 is stained with Alexa Fluor 594 (red) and nucleus is counterstained with DAPI (blue). (**c**) EPHA2, AKT1 and RSK phosphorylation was decreased after 48 h treatment with anti-CD13 blocking antibody. (**d**) Induction of apoptosis after 48 h treatment with anti-CD13 blocking antibody in A375, A375VR4 and SKMEL24 cells. Apoptosis was measured by flow cytometry based annexin V-PI staining. (Error bars represent S.D. of the mean of two independent experiments each in three replicates)

CD13/ANPEP is a zinc-dependent ectopeptidase with preference for cleavage of *N*-terminal neutral amino acids in extracellular proteins, with recognized activity toward RTKs and signal transduction pathways including MAPK.^20^ It is also suggested to have a role in cell proliferation and metastatic progression of tumors.^21^ CD13 is most highly expressed in A375VR4 cells (Figures 2a and b) with a higher migratory capacity and EPHA2 expression. To investigate if CD13 has an impact on EPHA2 we targeted CD13 with a blocking antibody and found that it led to complete dephosphorylation of EPHA2 on S897 without altering the total EPHA2 protein level. These results suggest that inhibition of CD13 reduces migration through dephosphorylation of EPHA2 (Figure 2c). A similar effect was observed in another BRAF-mutated cell line with intrinsic resistance to BRAFi, SKMEL24, which displays high basal EPHA2 phosphorylation (Figure 2c). It has previously been reported that phosphorylation of EPHA2 on S897 can be mediated by AKT and RSK.^22, 23^ Recently, it has also been shown that the specific AKT1 promotes melanoma metastasis and also mediates EPHA2 S897 phosphorylation.^24, 25^ We therefore investigated if the CD13 blocking antibody had an effect on AKT1 and RSK phosphorylation. We observed that both AKT1 and RSK were dephosphorylated (Figure 2c) and in addition total AKT1 was also downregulated.

Antibody-mediated inhibition of CD13 led to elevated apoptosis in both BRAFi-sensitive and -resistant cell lines (Figure 2d).

### Silencing of the novel potential BRAFi resistance candidate FLI1 re-sensitizes the melanoma cells to BRAFi

**Figure 3:**
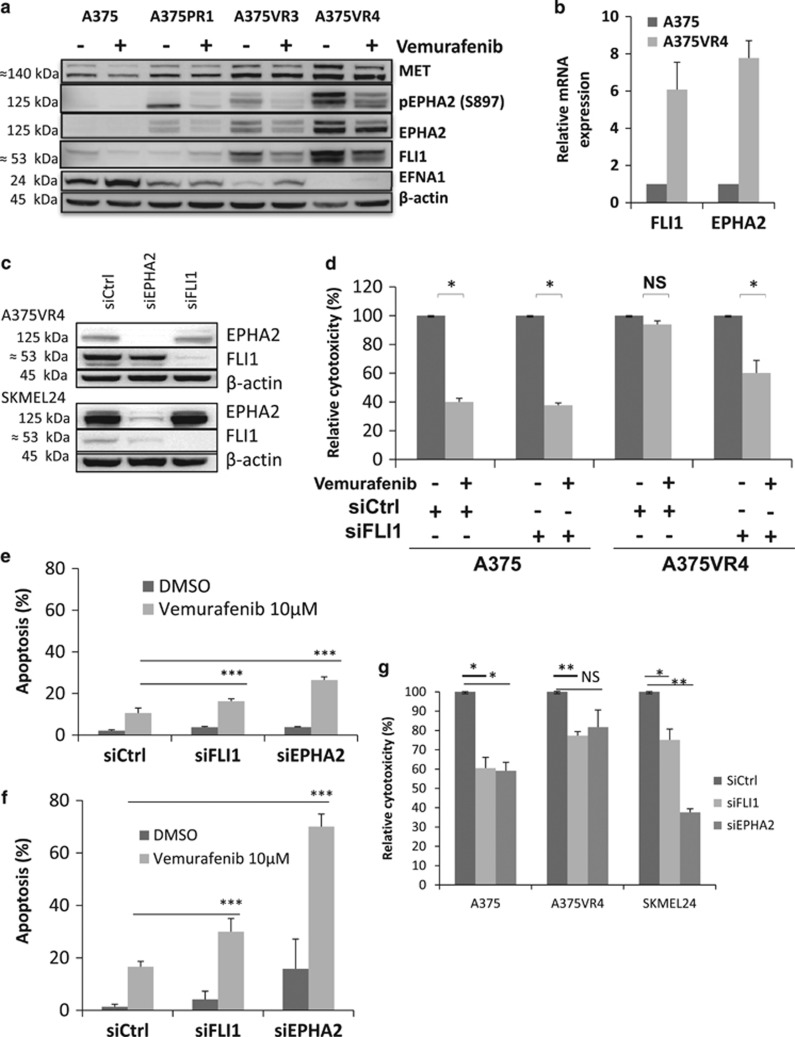
Potential BRAFi resistance candidates. Silencing of FLI1 or EPHA2 re-sensitizes the resistant A375VR4 and SKMEL24 sublines to vemurafenib. (**a**) Protein expression levels of MET, FLI1, pEPHA2, EPHA2 and EFNA1 analyzed by immunoblotting in presence of 1 *μ*M of vemurafenib or DMSO as control treatment for 24 h. *β*-actin was used as control. (**b**) Basal mRNA expression of FLI1 and EPHA2 in A375 and A375VR4 cell lines. (Error bars represent S.D. of the mean of three independent experiments). (**c**) Representative immunoblots showing downregulation of FLI1 and EPHA2 in A375VR4 and SKMEL24 after 48 h transfection with 100 nM siFLI1 or siEPHA2. (**d**) Silencing of FLI1 re-sensitized the A375VR4 cells to 5 *μ*M of vemurafenib. Bar graph showing cytoxic effect as % of DMSO control measured by MTS colorimetric assay in A375 and A375VR4 cells transfected with siFLI1 or sicontrol in presence of vemurafenib (or DMSO). (Error bars represent S.D. of the mean of three independent experiments). (**e**) Silencing of FLI1 or EPHA2 in A375VR4 cells increased the number of apoptotic cells after 10 *μ*M vemurafenib treatment. Bar graph showing percentage of apoptotic cells measured by annexin V-PI staining assay in A375VR4 cells transfected with siFLI1, siEPHA2 or sicontrol in presence of vemurafenib (or DMSO). (Error bars represent S.D. of the mean of three independent experiments). (**f**) Silencing of FLI1 or EPHA2 re-sensitized the SKMEL24 cells to vemurafenib. Bar graph showing percentage of apoptotic cells measured by annexin V staining assay in SKMEL24 transfected with siFLI1, siEPHA2 or sicontrol in presence of 10 *μ*M vemurafenib (or DMSO). (Error bars represent S.D. of the mean of three independent experiments). (**g**) Silencing of FLI1 and EPHA2 with siRNA affects cell proliferation. Bar graph showing percentage of viable cells measured by MTS colorimetric assay in A375, A375VR4 and SKMEL24 cells transfected with siEPHA2, siFLI1 or sicontrol without any treatment. (Error bars represent S.D. of the mean of at least three independent experiments). Paired *t*-test was used to determine differences between groups and *P*<0.05 was considered significant. *P*<0.05 * *P*<0.01 ** *P*<0.001 ***

FLI1, previously shown to be positively correlated to proliferation and with higher expression in metastatic melanoma tumors,^26^ was found to be overexpressed in our vemurafenib-resistant cells in the proteomics analysis and confirmed by immunoblotting (WB) (Figure 3a). Higher mRNA expression of FLI1 was also observed in the A375VR4 cells implying alteration at the transcriptional level (Figure 3b). We demonstrated that silencing of FLI1 in A375VR4 cells by siRNA re-sensitized the resistant cell lines to vemurafenib (Figures 3c and d) and increased vemurafenib induced apoptosis (Figure 3e). A similar result was also found in the SKMEL24 cells supporting the impact of FLI1 on BRAFi resistance (Figures 3c and f). In addition, a decreased proliferation of the cells in the absence of drug treatment was also observed in both parental and BRAFi-resistant cells (Figure 3g).

### Silencing of EPHA2 re-sensitizes the melanoma cells to BRAFi

The proteomics data and the immunoblots showed significant increase of EPHA2 and MET protein expression levels in the cell lines with acquired BRAFi resistance consistent with recent publications (Figure 3a).^11, 14^ SKMEL24, A375VR3 and A375VR4 cells have high levels of total EPHA2 as well as S897- phosphorylated EPHA2 (Figures 2c and 3a). Moreover the mRNA expression level of EPHA2 is highly increased in A375VR4 cells indicating a transcriptional upregulation (Figure 3b). An investigation by Taqman assay showed that the increased EPHA2 expression is not caused by further amplification of the EPHA2 gene (Supplementary Table S4).

SiEPHA2 transfected A375VR4 and SKMEL24 cells showed significantly elevated apoptosis in response to vemurafenib after 72 h treatment compared with cells transfected with control siRNA (Figures 3e and f). Moreover, silencing of EPHA2 with siRNA in A375 and A375VR4 cell lines led to a significantly lower number of colonies after treatment with vemurafenib compared with cells transfected with control siRNA after 14 days of treatment (Supplementary Figure S8). In addition, similar decreases in proliferation after FLI1 knockdown were also observed after EPHA2 silencing in A375, A375VR4 and SKMEL24 cells in the absence of drug treatment (Figure 3g) suggesting that EPHA2 has an impact on proliferation (Figure 3g) as well as being a mediator of vemurafenib resistance as reported previously.^11^

### Silencing of FLI1 decreases total MET and S897-phosphorylated EPHA2

**Figure 4:**
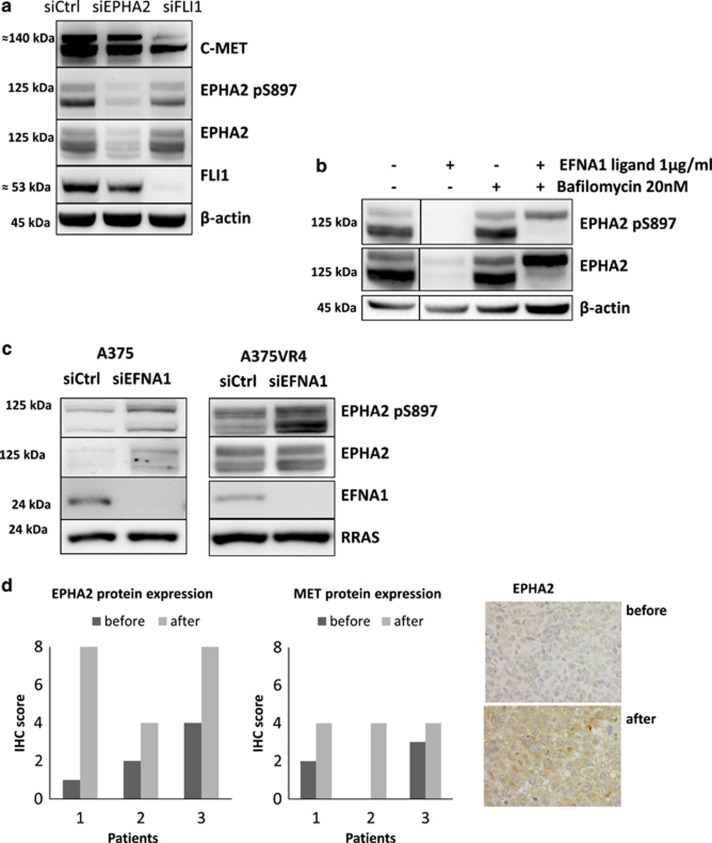
Higher EPHA2 and MET protein expression after progression. EFNA1 ligand downregulation leads to increased EPHA2/pEPHA2 protein expression. (**a**) EPHA2 and FLI1 protein expression downregulation after 48 h transfection with siRNA against EPHA2, FLI1 and negative sicontrol in A375VR4 cell line. (**b**) EPHA2 degradation in presence of 1 *μ*g/ml of EFNA1 ligand (2 h) and rescue from degradation by adding 20 nM bafilomycin 1 h before ligand treatment in A375VR4 cells. (**c**) The impact of EFNA1 downregulation on EPHA2/pEPHA2 protein expression level after 48 h transfection using siRNA against EFNA1 and sicontrol in A375 and A375VR4 cell lines. RRAS protein was used as loading control (**d**). EPHA2 and MET protein expression in FFPE tumor samples excised before treatment and after progression from three melanoma patients, who recieved first line treatment with BRAFi using IHC. Images show staining of EPHA2 expression in tumors from one patient before treatment and after progression

FLI1 and EPHA2 were silenced by siRNA to find out if the co-occurring upregulation of these proteins could be due to indirect or direct induction of each other. FLI1 silencing decreased total MET and S897-phosphorylated EPHA2, but not total EPHA2, in A375VR4 cells (Figure 4a). EPHA2 silencing decreased total FLI1 but not MET (Figure 4a).

### Re-discovered EPHA2 ligand-independent signaling in vemurafenib-resistant cells

The membrane bound ephrin-A1 (EFNA1) is a ligand for EPHA2^27^ and binding of EFNA1 to EPHA2 leads to degradation of EPHA2^28^ through the lysosomal degradation pathway. Constitutive downregulation of EFNA1 was observed in the BRAFi-resistant cells (Figure 3a) and addition of EFNA1 ligand to the A375VR4 cells led to EPHA2 degradation as expected (Figure 4b). Furthermore, inhibition of lysosomal activity by bafilomycin pretreatment (1 h) in A375VR4 abrogated EPHA2 degradation induced by EFNA1 treatment (Figure 4b). siRNA knockdown of EFNA1 in A375 and A375VR4 cell lines resulted in an increased total EPHA2 protein in A375 and hyper-phosphorylated EPHA2 in both cell lines with marked effect in A375VR4 (Figure 4c). Therefore, decreased EFNA1 expression may partially explain the higher EPHA2 phosphorylation in the A375VR4 cell line and indicate a shift from ligand-dependent to ligand-independent signaling. Also, treatment with EFNA1 decreased the migratory capacity of the A375VR4 cells as demonstrated in a scratch assay (Supplementary Figure S9).

### Expression of CD13/ANPEP, FLI1, EPHA2 and MET in matched tumor samples from melanoma patients obtained before BRAFi treatment and after disease progression

To demonstrate that upregulation of EPHA2 and MET also occurs in tumors of patients who develop resistance to BRAFi therapy we have analyzed tumor samples obtained before treatment and after progression in three melanoma patients receiving treatment with BRAFi. All three patients were responders to BRAFi therapy. Tumors from these patients (Figure 4d) had a lower expression of both EPHA2 and MET before treatment compared with after progression, in agreement with the *in vitro* findings shown in Figure 3a. In addition, targeted sequencing of mRNA from matched fresh frozen tumor biopsies obtained before treatment and after progression from two more patients was performed using the Ion AmpliSeq transcriptome human panel. One of the patients was a non-responder and the other was a responder. The non-responder had >10 times higher basal FLI1 and EPHA2 levels than the responder but lower mRNA expression of ANPEP and MET. However, MET mRNA was two to threefold increased after progression in both cases, which is in concordance with the immunohistochemistry (IHC) analysis of the other three patients. A three to six-fold increase of FLI1 and EPHA2 mRNA was also observed in the responder after progression but not in the non-responder. ANPEP was increased in the non-responder but not in the responder after progression.

Analyses to confirm the ampliseq finding was performed using qPCR. The mRNA MET and ANPEP data were confirmed but FLI1 differed for the responder, showing downregulation after progression. No EPHA2 mRNA could be detected with qPCR in the pretreatment sample from the responder. The ampliseq is a more sensitive assay, whereas the qPCR is a SybrGreen-based assay that can be a limitation when analyzing low amounts of mRNA.

Although protein expression was assessed in three of the cases and mRNA expression in two of the cases, overexpression of MET was observed in all progression samples while the expression of the other candidate markers varied between the individual patients.

### The multi kinase inhibitor dasatinib decreases EPHA2 S897 phosphorylation and FLI1 expression and shows synergistic effect in combination with BRAFi

**Figure 5:**
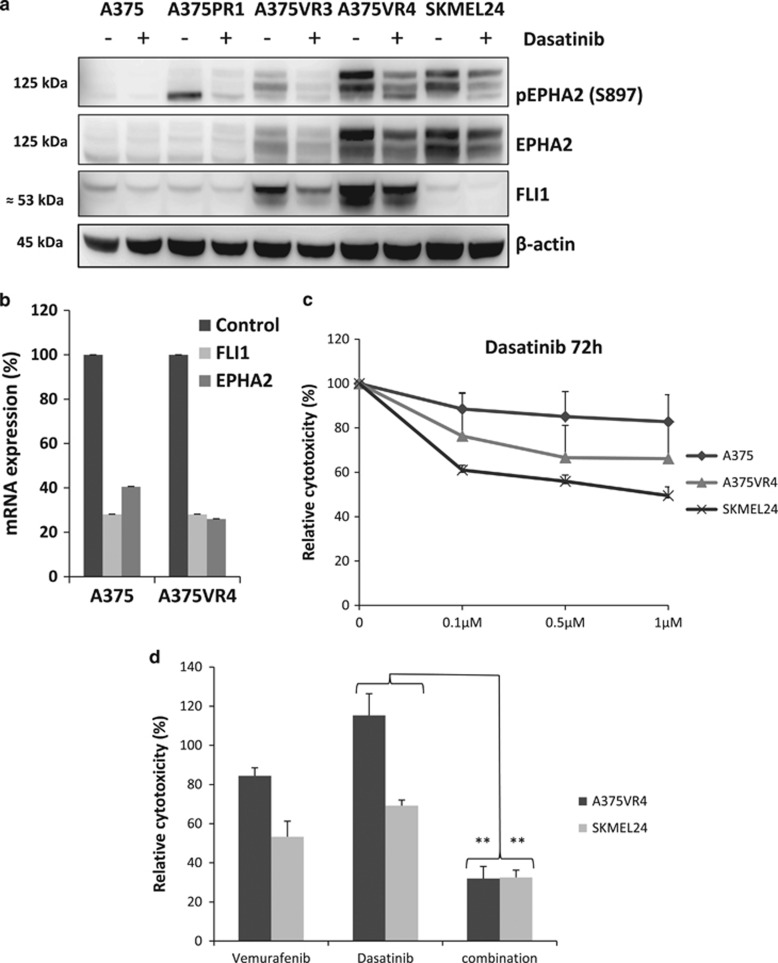
EPHA2 S897 phosphorylation and FLI1 expression is decreased by dasatinib treatment and shows synergistic effect in combination with vemurafenib. (**a**) Protein expression levels of EPHA2/pEPHA2 and FLI1 analyzed by immunoblotting in presence of 1 *μ*M dasatinib or DMSO as control treatment for 24 h. The lowest band for pEPHA2 in A375PR1 is unspecific. (**b**) Relative expression of FLI1 and EPHA2 mRNA after 24 h dasatinib treatment compared with DMSO control (Error bars represent S.D. of mean of three independent experiments). (**c**) Dose–response curves for 72 h dasatinib treatment of A375, A375VR4 and SKMEL24 cell lines showing cytoxic effect as % of DMSO control measured by MTS colorimetric assay. (Error bars represent S.D. of the mean of three independent experiments). (**d**) Single or combination treatment with vemurafenib+/-dasatinib for 72 h, showing cytoxic effect as % of DMSO control. Effect of low concentration of dasatinib combined (100 nM) with 10 *μ*M vemurafenib in A375VR4 and SKMEL24 cell lines measured by MTS colorimetric assay. (Error bars represent S.D. of mean of three independent experiments)

Dasatinib is a small molecule and multi kinase inhibitor previously shown to inhibit the SRC family^29^ and autophosphorylate of EPHA2.^30^ It has recently been demonstrated that treatment with dasatinib can overcome BRAFi resistance.^13^ Treatment with dasatinib led to decreased EPHA2 S897 phosphorylation in A375VR3, A375VR4 and SKMEL24 cells. Moreover, it also downregulated FLI1 protein expression in the BRAFi-resistant cells (Figure 5a). Dasatinib also downregulated EPHA2 and FLI1 mRNA expression levels compared with DMSO control (Figure 5b). The EPHA2 expressing A375VR4 and SKMEL24 cells were more sensitive to dasatinib compared with parental A375 (Figure 5c). We found that by combining dasatinib (at a 20 times lower concentration than IC50 for single treatment) with vemurafenib for 72 h could overcome vemurafenib resistance in a cooperating manner in A375VR4 and SKMEL24 cells.

## Discussion

This is the first report demonstrating that CD13/ANPEP and FLI1 are potential mediators of BRAFi resistance. Elevated expression of CD13 has been correlated to adverse clinical outcome in different cancers.^31, 32, 33^ CD13 has been shown to have variable expression during melanoma progression with low levels in melanocytes and high expression in melanoma cells.^34^ Through inhibition and blocking of CD13, its role in angiogenesis and invasive capacity during melanoma progression was uncovered.^35, 36, 37, 38^ Moreover, CD13/ANPEP has been shown to be a transcriptional target for the MAPK pathway.^34^ In the present study, the vemurafenib-resistant A375VR4 cells showed elevated protein expression of CD13/ANPEP compared with parental A375 cells. In line with CD13/ANPEP’s role in tumor progression, targeting the protein by a blocking antibody induced massive apoptosis and also abrogated the EPHA2 S897 phosphorylation, which has previously been demonstrated to cause inhibition of the migratory capacity.^22^ RSK and AKT have been demonstrated to induce EPHA2 S897 phosphorylation and thereby having an impact on the migratory capacity.^22, 23, 39^ Both are dephosphorylated by treatment with CD13 blocking antibody suggesting that both the MEK/ERK/RSK and PI3K/AKT pathways might be affected by the CD13 blocking antibody. Further studies are needed to understand the correlation between CD13 and RSK/AKT1.

In another study melanoma cell lines with overexpression of CD13/ANPEP, were shown to have reduced expression of melanocytic markers such as gp100, MART-1 and S100B suggestive of a low grade of differentiation.^40^ In the A375VR4 cell line low expression of SOX10 and MITF and increased expression of CD166 was observed. Previously Agnarsdottir *et al.*^41^ found an inverse correlation between SOX10 expression and the proliferation marker Ki-67 in melanoma tumors. This may partially explain the paradoxical characteristics of A375VR4 cells; altered expression of stemness associated markers, high migratory capacity but also high proliferative capacity. Reflecting the exposure to vemurafenib, the resistant A375VR3 and A375VR4 display more similar patterns of mRNA and protein levels compared with the parental A375. The similarities and dissimilarities of the sublines mimic melanoma intra tumor heterogeneity as observed in Kemper's recent paper^42^ and is a well-known problem for all types of therapy for melanoma.

FLI1 is a member of the large ETS transcription factor family^43^ and has been associated with an increased proliferation, differentiation and evasion from apoptosis in human cancer cells.^44, 45^ Aberrant FLI1 activation induces dysregulated cell division and malignant transformation,^46, 47^ and in the NIH3T3 murine cell line with induced overexpression of FLI1, drug-induced apoptosis was inhibited.^47^ In endothelial cells, ETS phosphorylation via the RAS/MAPK pathway is required for CD13 induction,^48^ suggesting that there may be a link between the ETS transcription factor family and CD13.

The A375VR4 cells overexpress FLI1, and silencing of FLI1 re-sensitized the cells to the drug, suggesting that FLI1 contributes to vemurafenib resistance. The effect was also shown in SKMEL24 with moderate FLI expression. Besides increased apoptosis in vemurafenib treated A375VR4/SKMEL24 cells, silencing of FLI1 also inhibited proliferation of these cells as well as parental A375 cells. In addition, silencing FLI1 resulted in downregulation of total MET protein and pEPHA2 but had no effect on total EPHA2 or MET mRNA levels, suggesting an indirect regulatory effect of FLI1 on MET and EPHA2 phosphorylation.

EPHA2 has recently been shown to mediate resistance to vemurafenib^11^ and is overexpressed in A375VR4 cells in a ligand-independent manner, confirming previous findings. Melanoma tumors expressing high EPHA2 form new metastatic sites under drug-induced pressure.^49^ Inhibition of upstream signaling combined with BRAF inhibition may reverse BRAFi resistance; this has been demonstrated by combining vemurafenib with EGFR inhibitors in cells with EGFR overexpression.^13^ As also observed, dasatinib inhibited growth and metastasis in a vemurafenib-resistant melanoma patient xenograft in immunocompromised mice.^13^ Concordant with previous studies, the SKMEL24 and A375VR4 cells with high EPHA2/pEPHA2 were more responsive to dasatinib treatment compared with parental A375 with low EPHA2 expression. Dasatinib decreased EPHA2 S897 phosphorylation and total FLI1 and cooperated with vemurafenib to inhibit proliferation. This potentiating effect of dasatinib has also been addressed by Montero *et al.*^50^

The small number of cell lines included in our study is a limitation but many of the previously reported BRAFi resistance candidates such as MET and EPHA2 were also identified in our study as strongly associated with resistance to BRAFi. In addition, we have not included any animal model, which can be considered as a limitation. However, the strength is that we could verify expression of resistance factors in patient samples after clinical tumor progression.

In summary, by gene expression and proteome profiling of the BRAFi-sensitive A375 melanoma cell line and of three daughter cell lines with induced resistance, we identified both previously known and novel mediators of resistance. For the first time, we show that CD13/ANPEP and FLI1 overexpression can mediate resistance to BRAFi in melanoma cells. Elucidation of links between these proteins can lead to identification of novel targets and more efficient cancer treatments. One such approach might be targeting CD13/ANPEP in tumors with elevated CD13/ANPEP expression. It remains to be elucidated whether high expression of CD13/ANPEP is associated with propensity of melanoma cells to exhibit ligand-independent EPHA2 signaling. Further understanding of the underlying resistance mechanisms to vemurafenib such as CD13/ANPEP and FLI1 overexpression holds great promise for designing therapy regimens in tumors with innate and acquired resistance to BRAFi.

## Materials and Methods

### Cell lines

The original A375 cells were purchased from ATCC. The cells were cultured in MEM media (Cat.no. 21090, Life Technologies, Carlsbad, CA, USA, Gibco) supplemented with 10% FCS, 1 × MEM non-essential amino acids (Cat. no. 11140-050, Life Technologies, Gibco, Carlsbad, CA, USA), 1 mM sodium pyruvate (Cat. no. 11360039, Life Technologies, Gibco), 100 U/ml penicillin and 100 *μ*g/ml streptomycin.

### Tumor samples

We included only CMM patients with tumor stage M1c who received BRAFi based (vemurafenib or dabrafenib) first line treatment at Karolinska University Hospital and with access to archival FFPE or fresh frozen melanoma tumor samples collected both before treatment and after progression. We could identify five patients that fulfilled the above mentioned criteria. Patient data are described in the supplementary. This study has obtained ethical approval from the regional ethics committee in Stockholm, Sweden and was performed in accordance with the ethical principles given in the Helsinki Declaration. Informed consent was obtained from the patients.

### BRAFi resistance induction

Resistance was induced in the A375 cells (purchased from ATCC CRL-1619) by repeated exposures to increasing concentrations of the vemurafenib and its analog PLX4720. These daughter cell lines are referred to as A375-PLX4720R1 (A375PR1), A375-vemurafenibR3 (A375VR3) and A375-vemurafenibR4 (A375VR4). Resistance in all the different sublines was induced independently of each other.

The A375 cells are hemizygous for the activating BRAF V600E mutation, and the parental A375 cells have an IC_50_ of ~<1 *μ*M vemurafenib.

Inhibitors PLX4720 (Cat. no. 553015) was purchased from EMD Chemicals (Gibbstown, NJ, USA), vemurafenib (Catalog No.S1267), trametinib (GSK1120212, Catalog No.S2673) and dasatinib (Catalog No.S1021) were purchased from Selleckchem (Munich, Germany). Bafilomycin A1 (Catalog No. 1793) is from Sigma-Aldrich (St. Louis, MO, USA).

### MTS colorimetric assay

CellTiter 96 AQueous One Solution Cell Proliferation Assay (MTS) was purchased from Promega (Cat. no. G3582, Promega, Madison, WI, USA) for colorimetric based method for determination of inhibitory concentration of the inhibitors at 490 nm wave length for absorbance and 690 nm for background

### Cell cycle analysis

Cells were plated at 5 × 10^4^ cells/well in 24-well-plates using appropriate growth medium 24 h before treatments. Cells were treated with vemurafenib 1 *μ*M, trametinib 2 nM or anti-CD13 blocking antibody 5 *μ*g/ml for 24 h. Then cells were harvested and collected by tryspsinization and washed with 1 × PBS and fixed with 4% formaldehyde overnight at room temperature. Next day cells were centrifuged and formaldehyde was replaced with 70% ethanol. For analysis, ethanol was removed and cell pellets were rinsed with milliQ water and pelleted again. They were then resuspended and incubated with protease at 37 °C for 1h, then cells were stained with DAPI and analyzed by BD LSRII flow cytometer (BD Biosciences, San Jose, CA, USA).

### Gene silencing with siRNA

EPHA2 siRNA FlexTube (cat no.: SI02223508) and AllStars Negative control siRNA (cat no. 1027280) were purchased from Qiagen, Hilden, Germany. ON-TARGET plus human MET siRNA (cat no. L-003156-00) and ON-TARGET plus human FLI1 siRNA (cat no. L-003892-00) were purchased from Dharmacon, GE lifesciences (Lafayette, CO, USA).

### DNA and RNA extraction

Cell line DNA and RNA extraction was performed using the product manual using RNeasy kit (cat no. 74104) and DNA mini kit (cat no. 51304) both from Qiagen. Quality and quantity control was performed using Agilent Bioanalyzer 2000 instrument and Nanodrop.

### Gene expression analyses

Analysis of gene expression was performed using Qiagen pathway analysis (RT^2^ Profiler PCR Array, Qiagen) and RT-qPCR for genes indicated to be differentially expressed in the parental A375 cells *versus* BRAFi-resistant sublines. The RNA extraction for RT-qPCR was performed using RNeasy Mini Kit (Qiagen) and cDNA synthesis of 1 *μ*g DNAse I treated RNA as measured by Agilent Bioanalyzer 2000 instrument (Agilent, Santa Clara, CA, USA) was performed utilizing Superscript III RT with 3:1 molar ratio of random hexamer-anchored oligo-dT (both Eurofins, Luxembourg, Luxembourg) priming mixture. The qPCR was performed in technical duplicates from biological triplicates from separate experiments using reference genes; for mRNA expression primer sequences see supplementary Table S5. The analyses are based on the delta Ct method and fold changes are converted to log2 values. Analyses of mRNA expression alterations were based on the delta Ct method and paired comparison with each of the BRAFi-resistant sublines. The fold change was converted to log2 values.

### Targeted sequencing using Ion AmpliSeq

Targeted sequencing of 20,802 different transcripts was performed using the Ion AmpliSeq transcriptome human panel that recognize >95% of all RefSeq genes with one amplicon designed for each gene. RNA was used as input material and amplicons were sequenced using the Ion Proton systems from Life Technologies as a service at the Uppsala Genome Center, Uppsala University, Sweden.

BAM-files were imported into the Genomics Suite software from Partek and analyzed using their built-in RNA-seq workflow. In brief, for each sample, total number of alignments, total number of reads, percentage of reads that overlap completely, partially or not with exonic regions etc, were determined. Number of counts for each transcript was normalized using the reads per kilobase per million reads (RPKM) method. Comparison of mRNA abundance of candidate transcripts among samples was done using the RPKM values.

### Targeted next-generation sequencing

To examine a set of known resistance factors and other potential candidates of biological relevance for melanoma, including *BRAF*, *NRAS* and *MEK,* we have performed a targeted sequencing using the Agilent HaloPlex technology followed by next-generation sequencing (Illumina Hiseq). For validation, genomic Sanger sequencing was performed using the BigDye Terminator v.3.1 system in ABI 3700 capillary electrophoresis system (both Applied Biosystems, Carlsbad, CA, USA). Genomic primers used are presented in supplementary Table S5.

### Copy number analyses

EPHA2 gene copy number was analyzed with custom TaqMan Copy Number Assay Hs01450667_cn relating the target gene abundancy with the TaqMan Copy Number Reference Assay RNase P (both Applied Biosystems). The analyses were performed according to the kit instructions in technical quadruplicates using ABI7900HT real-time instrument, using pooled DNA from healthy volunteers (blood donors) for confirming the diploid copy number estimate. The resulting CT values were analyzed using Copy Caller software v. 2.0 (Applied Biosystems).

### Protein extraction and acetone precipitation for mass spectrometry

RIPA lysis buffer system was used for cellular protein extraction. Around 12–14 million cells were cultured for 24 h in three consecutive passages. Cell pellets were mixed with the lysis buffer on ice for 20 min, sonicated at medium power for 5 min and centrifuged at 10,000 × *g* for 10 min and the supernatant were collected. Protein concentrations of the lysates were measured using the DC protein assay kit 2 (BioRad).

### Mass spectrometry analysis

The mass spectrometry analysis was performed using an Orbitrap Velos as previously described.^51^ Orbitrap data were searched by Sequest under the software platform Proteome Discoverer 1.3 (Thermo, Rockford, IL, USA) against the human Swissprot (2012-02) protein sequence database (supplementary text 2).

### Protein extraction and concentration measurement for WB

For protein extraction, RIPA lysis buffer system for mammalian cell and tissue lysis buffer were purchased from SantaCruz biotechnology (Dallas, TX, USA) (Catalog No. sc-24948). Protein extraction was performed using the product manual and concentrations were measured by Micro BCA Protein Assay Kit (Life Technologies cat no. 23235) at 562 nm wave length according to the product manual. Equal amounts of soluble proteins (30–50 *μ*g) were mixed with NuPAGE sample reducing agent (Life technologies) and heat denatured at 95ºc for 5 min and loaded on 10–12% Bis-Tris pre made gels (Life technologies) and transferred to 0.45 *μ*m PVDF membrane (Thermo Scientific). After blocking in 5% non-fat dry milk or BSA in 1 × TBS-T for 1 h and probing with a specific primary antibody and a horseradish peroxidase-conjugated secondary antibody, the protein bands were detected by chemiluminescence (Supersignal, Pierce) and CCD camera. Protein loading was normalized by using anti-*β* actin antibody.

### WB

To validate selected protein candidates, protein extracts from SKMEL24, A375 and the BRAFi-resistant sublines were analyzed by WB using NuPAGE Novex Bis-Tris Gel (Life Technologies) and PVDF membranes (Thermo Scientific), according to the manufacturer’s standard protocol. Antibodies for IHC and WB are purchased from: Anti-EPHA2 (sc-924) (IHC and WB) and anti-MET (sc-10) (IHC) SantaCruz Biotechnology INC.; Anti-EFNA1 (GTX63281) (WB) GeneTex; Anti-EFNA1 NBP1-30503 (IHC) and anti-FLI1 NBP1-95688 (IHC and WB) Novus Biologicals LLC (Littleton, CO, USA); Anti-phospho EPHA2 (Ser897) (WB) #6347, anti-MET (WB) #8198,, anti-pAKT1 (S129) #13461, anti-AKT1 #75692, anti-P-p90RSK (S380) #11989 and anti-RSK1/RSK2/RSK3 # 9355 Cell Signaling Technology (Danvers, MA, USA); anti-CD13/ANPEP #HPA004625 Protein Atlas; anti-*β*-actin (A5441) (WB) and anti-RRAS (WH0006237M1) (WB) Sigma life Science, Anti-CD13 (ab7417) (blocking antibody) Abcam, Cambridge, UK.

### Scratch assay (wound-healing assay)

Human Ephrin-A1 recombinant protein (EFNA1 ligand, His &FC tagged) (10882-H03H) was purchased from Sino Biological Inc. (Beijing, China) A375 and A375VR4 cell lines were seeded in six-well plates for 24 h to reach ~90% confluency and with the tip of a sterile tip a scratch were made along the well and medium was changed and the gap filling was measure in the presence and absence of Vemurafenib, EFNA1 ligand and combination of Vemurafenib with EFNA1 ligand.

### Colony forming assay

In 4-cm plates, 200 cells were plated and 24 h after seeding they were treated with Vemurafenib or siRNA against EPHA2. The number of colonies formed are fixed in crystal violet and counted 14 days after seeding. Culture media of the cells were changed every 3 days but the treatments were done only once.

### Flow cytometry based immunostaining and apoptosis/necrosis analysis

To evaluate the presence of apoptosis/necrosis, we used annexin V-Fluos (cat. no. 11828681001 Roche) and Propidium iodide (PI) and analyzed by NovoCyte flow cytometer (ACEA biosciences, Inc. San Diego, CA, USA). Between 4 and 10 × 10^4^ cells per well were cultured and treated with vemurafenib for 72 h or transfected with siRNA 48 h prior to the 72 h vemurafenib treatment. Then the cells were collected and rinsed in PBS, pelleted, and resuspended in incubation buffer (10 mmol/l HEPES/NaOH, pH 7.4, 140 mmol/l NaCl, 5 mmol/l CaCl_2_) containing 1% annexin V and 1% PI for 10 min. For immunostaining against CD13 surface expression, cells were harvested and stained with FITC conjugated CD13 antibody (product No. F083101-2, Dako Sweden AB, Stockholm, Sweden) for 30 min at 4 °C in the dark followed by analysis using NovoCyte flow cytometer (ACEA biosciences).

### Immunofluorescence microscopy

Hundred thousand cells were seeded on coverslips in 12-well-plates and after 24-h incubation in 37 °C CO_2_ incubator; the cells were rinsed with 1 × PBS and fixed in 4% formaldehyde for 15 min. Cell membrane was permeabilized with 0.1% triton X100 diluted in PBS and for minimizing the risk of unspecific binding, cells were treated with blocking buffer containing 5% horse serum diluted in PBS for 1 h. Then cells on coverslip were incubated overnight with primary antibody against pAb anti-CD13 (ab7417 Abnova diluted 1:100 in blocking buffer, Taipei, Taiwan) at 4 °C overnight. Then cells were rinsed in PBS 3 × 5 min and incubated with goat anti-Mouse IgG (H+L) secondary antibody, Alexa Fluor 594 conjugated for 1 h at room temperature in the dark. At last, coverslips were sealed by adding mounting medium containing DAPI. The images were captured by Axioplan 2 fluorescence microscope equipped with CCD camera and AxioVision Rel. 4.7 software.

### IHC

IHC was performed on formalin fixed, paraffin embedded 3–4 *μ*m thick tumor sample sections. Antigen retrieval was induced by heating the sections in humid decloaking heat chambers (Biocare, Concord, CA, USA) in citrate buffer (pH 6.0) according to the manufacturer’s instruction followed by 10 min incubation with 3% hydrogen peroxide at room temperature and wash with 1 × TBS buffer. To avoid unspecific binding, the sections were incubated with 2.5% horse serum for 20 min at room temperature followed by overnight incubation at 4 °C with primary antibodies against Epha2 and MET C-MET in TBS buffer containing 1.5% horse serum. Negative staining controls were incubated with the same TBS buffer without the primary antibody.

Incubation with the secondary antibody plus streptavidin/ peroxidase was performed according to the manufacturer’s instructions (Vectastain Universal Quick Kit, Vector) and developed with 3,3’′-diaminobenzidine (DAB kit, Vector Laboratories Inc., Burlingame, CA, USA). As the final step, slides are rinsed in water and counterstained with Mayer‘s haematoxylin, rinsed with water, dehydrated and sealed with glass lamella and mounting solution Mountex (Histolab, Askim, Sweden).

Four observers (FCS, MFS, LK and SEB), blinded to clinical data, independently evaluated the whole area of the tumors. Discrepancies were solved by further review by all the observers and a consensus was reached.

For the IHC analysis we have categorized the samples using semiscores by multiplying the intensity (0–3) of the EPHA2 and MET protein expression with the percentage of positive melanoma cells (0, 1=1–24%, 2=25–49%, 3=50–74% and 4=75–100%).

### STR profiling

STR profiling of A375 cells and all the BRAFi-resistant sublines were performed to confirm the authenticity of the cell lines.

## Publisher’s Note

Springer Nature remains neutral with regard to jurisdictional claims in published maps and institutional affiliations.

## Figures and Tables

**Table 1:** Alterations of mRNA and protein expression in the BRAFi-resistant cell lines compared with parental A375 cells

**MAPK qPCR pathway array (log2)**				**Proteomics (log2)**			
**Symbol**	**A375PR1**	**A375VR3**	**A375VR4**	**Symbol**	**A375PR1**	**A375VR3**	**A375VR4**
CCNA2	−0.41	1.02	1.11	CCNA2	0.29	0.10	−0.07
CCND1	1.00	3.70	4.05	CCND1	−0.30	1.02	1.28
CDC42	0.01	1.51	1.31	CDC42	0.14	−0.16	0.14
CDK2	1.34	2.21	1.59	CDK2	−0.25	0.03	−0.32
CDK6	−1.27	0.47	0.25	CDK6	−1.26	−0.27	−0.73
CDKN1A	−0.77	0.51	1.03	CDKN1A	−0.13	0.85	1.53
CDKN2C	−0.25	1.46	1.28	CDKN2C	−0.36	0.66	0.68
CREBBP	−0.33	2.92	2.84	CREBBP	0.09	0.18	0.19
EGFR	0.69	1.14	2.00	EGFR	0.63	0.56	1.24
ETS1	−2.45	2.17	2.48	ETS1	−1.17	−0.15	−0.34
GRB2	−0.16	1.08	0.96	GRB2	−1.00	0.05	−0.22
HSPA5	0.86	1.10	1.02	HSPA5	0.38	0.18	0.31
HSPB1	1.31	−1.07	−3.34	HSPB1	0.08	0.20	−0.84
JUN	−1.48	1.73	1.39	JUN	0.29	0.14	0.28
KRAS	−0.02	1.86	1.73	KRAS	0.15	0.01	−0.16
MAP2K1	−0.10	1.87	1.98	MAP2K1	−0.56	0.52	0.55
LAMTOR3	−0.32	−3.22	−3.77	LAMTOR3	0.40	0.22	0.16
MAP2K2	−1.40	1.23	1.03	MAP2K2	−1.22	−0.53	−0.51
MAP2K3	0.10	1.47	0.99	MAP2K3	−0.51	−0.39	−0.75
MAP2K4	−0.89	0.82	1.22	MAP2K4	−1.03	0.38	0.77
MAP2K7	−0.86	3.16	3.56	MAP2K7	−1.33	0.08	0.003
MAPK3	0.15	1.61	1.37	MAPK3	−0.76	−0.31	−0.58
MAX	−0.52	1.02	0.79	MAX	0.09	−0.07	−0.14
PAK1	−0.003	2.13	2.27	PAK1	−0.81	−0.34	−0.50
RAF1	−0.71	1.34	1.86	RAF1	−0.45	−0.13	−0.31
SMAD4	−0.05	1.07	0.79	SMAD4	−0.48	−0.17	−0.30

MAPK pathway array and corresponding protein data from proteomics indicate gene/protein expression alterations in the BRAFi-resistant cells. A negative log2 value is indicative of downregulation and a positive log2 value shows upregulation of mRNA/protein expression

**Table 2:** Anlysis of selected mRNA and protein expression alterations in the BRAFi-resistant cell lines compared with A375 parental cells

	**RNA**			**Protein**			
**Genes**	**A375PR1**	**A375RV3**	**A375RV4**	**A375PR1**	**A375VR3**	**A375VR4**	**Protein**
ALCAM	ND	ND	1.21	−0.15	0.83	1.23	CD166
ANPEP	ND	ND	3.8	1.88	1.96	2.73	CD13
CCND1	1	3.7	4.05	−0.3	1.02	1.28	CCND1
EFNA1	ND	ND	−0.45	−0.42	−1.17	−1.27	EFNA1*
EGFR	0.69	1.14	2	0.63	0.56	1.24	EGFR
EPHA2	1.32	2.77	3.29	0.46	1.86	2.3	EPHA2
FLI1	ND	ND	2.7	−1.16	0.79	1.61	FLI1*
MET	-0.07	0.5	0.88	1.08	0.89	1.28	MET
MITF	ND	ND	−2.43	−0.42	−1	−1.34	MITF
PTEN	ND	ND	ND	−1.19	−0.5	−0.48	PTEN
SOX10	ND	ND	−2.03	−0.36	−0.81	−1.13	SOX10

A negative log2 value is indicative of downregulation and a positive log2 value shows upregulation of mRNA or protein expression*Measurement from immunoblotting
