# Supplementary material for: Silencing FLI or targeting CD13/ANPEP lead to dephosphorylation of EPHA2, a mediator of BRAF inhibitor resistance, and induce growth arrest or apoptosis in melanoma cells
Source: Cell Death Dis. 2017 Aug 31;8(8):e3029–. doi: 10.1038/cddis.2017.406 (PMC5596587; doi:10.1038/cddis.2017.406)
Supplement: Supplementary Information [file cddis2017406x1.docx]

**Material and methods**

**Clinical patients data**

Patient 1 was a woman born in 1946 who was diagnosed in 2007 with a BRAFV600 mutated T3bN1M0 acral lentiginous melanoma on the right foot, with a regional lymph node metastasis (T3bN1M0). Approximately 6 months after the primary surgery she presented with a satellite metastasis on her right leg which was totally resected. Over the following years she was operated several times for recurrent melanoma, received treatment with hyperthermic limb perfusion with melphalan, local treatment with miltefostine and local radiotherapy due to progressing resistant cutaneous and subcutaneous metastases on the right leg. In October 2011 two brain metastases were treated with gamma knife. In December 2011 treatment with vemurafenib started. She experienced a good clinical response initially but in April 2012 a new small metastatic lesion developed in the brain which was treated with gamma knife. Treatment with vemurafenib continued until August 2012 when several new lesions appeared in the brain. One of the lesions had been irradiated previously and was resected with the purpose of symptom relief. The rest of the lesions were treated with gamma knife again but after further progression in the brain whole brain radiotherapy in combination with temozolomide was performed. She died in January 2013.

The tumor sample before treatment start was obtained from a cutaneous metastasis in 2007 and biopsy material at progression from the brain metastasis removed in August 2012.

Patient 2 was a young man born in 1989 who was diagnosed in 2010 with a T3aN0M0 superficial spreading CMM on his left ear. One year later he had developed a metastatic lesion in the left parotid gland and massive lymph node metastases in the left side of the neck on and underwent surgery followed by post-operative radiation therapy. In June 2012 he was diagnosed with metastatic BRAF mutated CMM to intra abdominal lymph nodes and with subcutaneous metastases. Treatment with dabrafenib started in July 2012 and the patient had initially a partial response. However, in January 2013 the patient had progressive disease and went on to receive 4 cycles with ipilimumab as second line treatment, with mixed response. In May 2013 he underwent surgery due to bowel obstruction from an intestinal metastasis. In April 2013 he was diagnosed with a brain metastasis and received treatment with gamma knife and in August 2013 he was treated with carboplatin and paclitaxel (without signs of response) due to progressive disease intra-abdominally. The patient died from metastatic disease in December 2013.

The analyzed tumor samples were obtained before treatment start from metastatic lymph nodes in his neck and at progression from his metastatic bowel lesion resected in May 2013.

Patient 3 was a man born in 1959. He was operated in 2011 due to a stage III superficial spreading CMM of the right leg (T4bN1M0). In April 2012 local recurrences, with BRAFV600 mutation, were diagnosed by fine needle biopsy. CT showed unresectable lymph node metastases in the right groin and pelvis. Treatment with dabrafenib and trametinib started in June 2012 with partial response. Progressive disease was documented in April 2013 and ipilimumab treatment was started. This was interrupted and whole brain radiotherapy combined with temozolomide was delivered due to multiple brain metastases. Treatment with ipilimumab was then completed. The patient progressed in the brain after the fourth cycle of ipilimumab and treatment with temozolomide was re-initiated. The patient died due to progressive disease in November 2013.

The tumor samples obtained before treatment start were from lymph nodes in the right groin and at recurrence from a cutaneous metastasis of the right leg in May 2013.

Patient 4 was a man born in 1946 who was diagnosed in 1998 with a T1aN0M0 superficial spreading CMM in the right lumbar area. In February 2012 a fine needle biopsy of an enlarged lymph node in the right axilla confirmed regional metastasis. A complete regional lymph node dissection was performed. In total, metastases were found in 8 of 29 lymph nodes. In April, 2010 the patient was randomized in the EORTC18071/CA184-029 double blind phase III trial of adjuvant ipilimumab versus placebo in patients with totally resected stage III CMM.

In September, 2010 a lymph node dissection, of the left axilla, was performed showing lymph node metastases in 11 out of 23 lymph nodes and post-operative radiotherapy with 30 Gy was delivered.

In January 2012 a positron emisson tomography-CT (PET-CT) showed evidence of disease in supraclavicular fossa bilaterally and retrosternal and a new fine needle biopsy confirmed metastatic CMM with BRAF V600 mutation. Palliative treatment with vemurafenib was started in March 2012 for stage IV M1c melanoma (elevated lactate dehydrogenase – LDH).

A new PET-CT in May 2015 showed progressive disease with a new enlarged intra abdominal lymph nodes and a solitary brain metastasis. However, the lymph nodes in supraclavicular fossa responded to treatment. The solitary brain metastasis was treated with gamma knife and treatment with vemurafenib was continued beyond progression. New evaluation with PET-CT in August 2012 confirmed general disease progression. The patient started a second line treatment with temozolomide but he unfortunately died in December 2012. The two tumor samples were both obtained from the same metastasis in right supraclavicular fossa before start of vemurafenib treatment in March 2012and at progression in August 2012.

Patient 5 was a woman born in 1957 who in March, 2013was diagnosed with a T4aN1M0 CMM in the left foot followed by post-operative radiotherapy. in the left groin.

In October 2013 she had a verified recurrence of BRAF V600 mutated CMM in the left groin and subcutaneous metastases in the lower left area of the abdominal wall. Imaging showed disseminated disease in lungs, liver, lymph nodes and soft tissues. Vemurafenib was started in October 2013. This patient had initially a good response but in June 2014 the disease progressed with new subcutaneous lesions in the left flank and with an increase of a previous metastatic lymph node in the left groin. She has received second, third and fourth line treatment with ipilimumab, temozolomide and anti-PD1 but died in March 2015 due to progressive disease.

The tumor samples were obtained before vemurafenib treatment start from a subcutaneous metastasis in the abdominal wall in October 2013 and at progression from a lymph node in the left groin in July 2014.

**Proteomics**

*Digestion, iTRAQ labeling and clean-up of protein samples*

Samples were digested using the filter aided sample (FASP) preparation method and labeled according to a standard iTRAQ 8-plex protocol (AB Sciex). One hundred microgram of each sample was labeled and two pools were made each with seven samples and one internal standard for normalization. In each of the pools two of the cell lines were included in triplicates (A375 and A375PR1 in pool A and, A375VR3, A375VR4 in pool B) and for the internal standard one independent replicate of A375 cells were used for normalization. Pooled iTRAQ labelled digests were applied to 1ml Strata X-C 33 µm polymeric strong cation exchange (SCX) microcolumns (Phenomenex). The microcolumns were initially washed with 1 ml 100% methanol followed by 1 ml MilliQ grade water. The sample was adjusted to 500 µl 0.1% formic acid and then applied to the columns. After wash with 1 ml 30% methanol and 0.1% formic acid the samples were eluted with 30% methanol and 5% ammonium hydroxide. Samples were then dried in a SpeedVac system.

*Narrow range isoelectric focusing*

Briefly, narrow range isoelectric focusing (IEF) was performed as follows: Samples were added to pH 3.3 loading strips, kindly provided by GE healthcare, and allowed to re-swell overnight. Loading strips were then put on re-swelled 24 cm narrow range isoelectric focusing strips pH 3.7-4.9 also provided by GE healthcare. Samples were focused until 100 kVhs was reached. The peptides were passively eluted in 72 fractions of 150µl milliQ water using in-house robot. Eluted peptide samples were then dried in a speedvac system.

*Mass spectrometry analysis*

A precursor mass tolerance of 10 ppm, and product mass tolerances of 0.02 Da for HCD-FTMS and 0.8 Da for CID-ITMS were used. Further settings used were: trypsin with 1 missed cleavage; carbamidomethylation on cysteine and iTRAQ-8plex on lysine and N-terminal as fixed modifications; and oxidation of methionine and phosphorylation of serine, tyrosine and threonine as variable modification. The phosphorylation sites were determined by the phosphoRS node and false discovery rate (FDR) calculations were performed using Percolator, both within Proteome discoverer. The quantification of iTRAQ-8plex reporter ions was done by Proteome Discoverer on HCD-FTMS tandem mass spectra using a integration window tolerance of 20ppm. In total 7810 proteins were identified with 1% peptide FDR cut off. Out of these 6697 proteins were detected in all samples. The mass spectrometry proteomics data have been deposited to the ProteomeXchange Consortium^3^ via the PRIDE partner repository with the dataset identifier PXD001682

*Statistical analysis*

Quality assessment of the LC/MS/MS data was done using principal component analysis (PCA) to check for outliers (SIMCA statistical analysis software, Umetrics, Sweden). Multivariate orthogonal partial least squares (OPLS) analysis was performed, also using SIMCA. For optimization of OPLS models we used VIP (Variable Importance in projection) value to judge protein importance in the model. The OPLS models were validated by sevenfold cross validation. Proteins with significant VIP throughout the cross validation of the model were selected for the optimized model. CV-ANOVA was used to judge the model validity (REF PMID: 15448969). Biological pathways analyses were performed using the Ingenuity IPA software (Ingenuity Systems Inc, USA).

**Sequencing**

*Targeted next generation sequencing (NGS)*

Mutational analysis for melanoma related genes including *BRAF*, *NRAS* and *MEK,* was performed utilizing the Agilent HaloPlex technology followed by massive parallel sequencing for the A375, A375PR1 and A375VR3 cells. The design was for >21 000 amplicons encompassing the coding regions, intron-exon borders and partial 3’ and 5’ untranslated regions of the selected genes, comprising in total 485 879 bases with a target coverage of over 98%. Capture and enrichment of the target regions was performed using the HaloPlex Target Enrichment kit (fast protocol), according to the manufacturer’s instructions. Sequence reads were mapped against the human reference genome (GRCh37 (hg19)) using BWA version 0.7.10 and variants were called using the GATK pipeline; in a customized HaloPlex pipeline (more information is available upon request). The GATK tool VariantEval was run to obtain quality control information for the variant calls. Annotations of variants were executed using the Annovar software and custom scripts.

**Results**

***No secondary mutations detected in BRAF, NRAS or MEK by targeted next-generation sequencing and Sanger sequencing***

To investigate the presence of secondary mutations in BRAFi resistant sublines mutational analysis of MAPK signaling related genes including *BRAF*, *NRAS* and *MEK,* was performed with targeted next-generation sequencing (NGS) utilizing the Agilent HaloPlex technology followed by massive parallel sequencing (Illumina Hiseq 2000) in A375, A375PR1 and A375VR3.

Validation of the observed genetic variants, zygosity of previously determined genetic variants and absence of secondary mutations were confirmed by Sanger sequencing in all resistant sublines including A375VR4. As expected, a hemizygous mutational status for *BRAF* (g.171429T>A: V600E) was seen in A375 and confirmed in all daughter cell lines using quantitative qPCR (data not shown). No *BRAF* splice variants were identified in the BRAFi resistant sublines and the absence of novel mutations in *BRAF*, *NRAS*, *RRAS*, *KRAS, RAC1, MET or PTEN*was demonstrated by NGS/Sanger sequencing (Supplementary Table S1). A detailed analysis description is available upon request.

All cell lines were found to carry mutations in *MAP2K2* c.T254G:p.V85G (*MEK2*) and *MAP3K9* c.113_115del:p.38_39del (*MEKK9*), but lack mutations in *MAP2K1* (*MEK1*) apart from a shared SNP (single nucleotide polymorphism) (rs368099654). No additional secondary mutations were found in *MEK2* and *MEKK9* in the resistant cell lines.

Similarly, absence of deleterious mutations and presence of a heterozygous, non-synonymous amino acid change g.G7753C: p.P72R in exon 4 of the *TP53* gene was confirmed. Also, the status of homo/hemizygous truncating mutations in exon 2 of *CDKN2A* was observed not to differ between the parental cell line and any of the sub-lines (NM_000077:c.G181T:p.61EX and NM_000077:c.G205T:p.E69X).

*RRAS* was found to carry 3 different, genetic variants without amino acid sequence altering effects g. 49636788G>A; g.49636811G>A; NM_006270:c.C333T: p.111N>N, g.49636835C>T.

In validation of the positive findings from NGS, a possibly deleterious variant in PRDM2 gene NM_001135610:c.A214C:p.N72H was confirmed to be present in heterozygous form in parental and A375PR1 cell lines, but absent in A375VR3 and A375VR4 sublines. In addition, two intronic SNPs were confirmed: rs35643856 (all cell lines) and rs35153743 (parental A375 only), the latter suggesting a zygosity change.

Similarly, *TP53BP1*:NM_001141979:c.A3421C:p.K1141Q (rs2602141) variant was confirmed to be heterozygous in all cell lines except A375PR1 that displayed a homo/hemizygous alteration. Corresponding difference between A375PR1 and other cell lines was indicated for *TP53BP1* variant NM_001141979: c.G1249A:p.G417S (rs689647), but the zygosity difference was neither rejected nor confirmed by Sanger sequencing due to low signal. The genomic positions refer to the genomic reference GRCh37 (hg19). For primer sequences see supplementary table S5.
