# Supplementary material for: Silencing FLI or targeting CD13/ANPEP lead to dephosphorylation of EPHA2, a mediator of BRAF inhibitor resistance, and induce growth arrest or apoptosis in melanoma cells
Source: Cell Death Dis. 2017 Aug 31;8(8):e3029–. doi: 10.1038/cddis.2017.406 (PMC5596587; doi:10.1038/cddis.2017.406)
Supplement: Supplementary Table S1 [file cddis2017406x2.pdf]

Supplementary Table 1. Mutation screening/validation results for A375 and three BRAF inhibitor resistant sub-lines, consensus denotes according to database sequence

Genes	A375parental	A375PR1	A375VR3	A375VR4
**NRAS**	consensus	consensus	consensus	consensus
**KRAS**	consensus	consensus	consensus	consensus
**RRAS**<br>g.49635672G>A; g. 49636788G>A;<br>g.49636811G>A;<br>NM_006270:c.C333T: p.111N>N<br>g.49636835C>T	all four variants (heterozygous)	all four variants (heterozygous)	all four variants (heterozygous)	all four variants (heterozygous)
**BRAF**<br>g.171429T>A:p.V600E	p.V600E	p.V600E	p.V600E	p.V600E
**RAC1**<br>g.6387309C>T, g.6392059C>T	g.6387309CC>TT<br>g.6392059CC>TT	g.6387309CC>TT<br>g.6392059CC>TT	g.6387309CC>TT<br>g.6392059CC>TT	g.6387309CC>TT<br>g.6392059CC>TT
**MET**<br>(exons 1-3, 16-20)	consensus	consensus	consensus	consensus
**PTEN**<br>(exon 1, exons 3-6, exons 10-13)	consensus	consensus	consensus	Consensus
