# Supplementary material for: Silencing FLI or targeting CD13/ANPEP lead to dephosphorylation of EPHA2, a mediator of BRAF inhibitor resistance, and induce growth arrest or apoptosis in melanoma cells
Source: Cell Death Dis. 2017 Aug 31;8(8):e3029–. doi: 10.1038/cddis.2017.406 (PMC5596587; doi:10.1038/cddis.2017.406)
Supplement: Supplementary Table S2 [file cddis2017406x3.pdf]

A375 vs. A375PR1

Gene ID	Gene Name	Fold Change	q-value(%)
O75525-2	Isoform 2 of KH domain-containing, RNA-binding, signal transduction-associated protein 3 OS=Homo sapiens GN=KI	19,15	0,00
Q17RY6	Lymphocyte antigen 6K OS=Homo sapiens GN=LY6K PE=1 SV=2 - [LY6K_HUMAN]	55,72	0,00
Q6UX53	Methyltransferase-like protein 7B OS=Homo sapiens GN=METTL7B PE=2 SV=2 - [MET7B_HUMAN]	13,49	0,00
P43355	Melanoma-associated antigen 1 OS=Homo sapiens GN=MAGEA1 PE=1 SV=1 - [MAGA1_HUMAN]	50,18	0,00
O14684	Prostaglandin E synthase OS=Homo sapiens GN=PTGES PE=1 SV=2 - [PTGES_HUMAN]	739171214,08	0,00
P15144	Aminopeptidase N OS=Homo sapiens GN=ANPEP PE=1 SV=4 - [AMPN_HUMAN]	6,38	0,00
Q96FQ6	Protein S100-A16 OS=Homo sapiens GN=S100A16 PE=1 SV=1 - [S10AG_HUMAN]	3,38	0,00
Q68CR1-3	Isoform 3 of Protein sel-1 homolog 3 OS=Homo sapiens GN=SEL1L3 - [SE1L3_HUMAN]	48,51	0,00
Q16698	2,4-dienoyl-CoA reductase, mitochondrial OS=Homo sapiens GN=DECR1 PE=1 SV=1 - [DECR_HUMAN]	3,12	0,00
Q9NP90	Ras-related protein Rab-9B OS=Homo sapiens GN=RAB9B PE=1 SV=1 - [RAB9B_HUMAN]	5,89	0,00
Q12797	Aspartyl/asparaginyl beta-hydroxylase OS=Homo sapiens GN=ASPH PE=1 SV=3 - [ASPH_HUMAN]	4,27	0,00
Q9Y5S1	Transient receptor potential cation channel subfamily V member 2 OS=Homo sapiens GN=TRPV2 PE=1 SV=1 - [TRPV	6,41	0,00
Q11201	CMP-N-acetylneuraminate-beta-galactosamide-alpha-2,3-sialyltransferase 1 OS=Homo sapiens GN=ST3GAL1 PE=2 S	6,79	0,00
P11498	Pyruvate carboxylase, mitochondrial OS=Homo sapiens GN=PC PE=1 SV=2 - [PYC_HUMAN]	3,59	0,00
P43304	Glycerol-3-phosphate dehydrogenase, mitochondrial OS=Homo sapiens GN=GPD2 PE=1 SV=3 - [GPDM_HUMAN]	2,58	0,00
Q16762	Thiosulfate sulfurtransferase OS=Homo sapiens GN=TST PE=1 SV=4 - [THTR_HUMAN]	2,60	0,00
Q9UKA9	Polypyrimidine tract-binding protein 2 OS=Homo sapiens GN=PTBP2 PE=1 SV=1 - [PTBP2_HUMAN]	6,84	0,00
Q9UBG0	C-type mannose receptor 2 OS=Homo sapiens GN=MRC2 PE=1 SV=2 - [MRC2_HUMAN]	2,75	0,00
Q9UKU7	Isobutyryl-CoA dehydrogenase, mitochondrial OS=Homo sapiens GN=ACAD8 PE=1 SV=1 - [ACAD8_HUMAN]	3,04	0,00
O60513	Beta-1,4-galactosyltransferase 4 OS=Homo sapiens GN=B4GALT4 PE=1 SV=1 - [B4GT4_HUMAN]	3,03	0,00
Q9NTG7	NAD-dependent deacetylase sirtuin-3, mitochondrial OS=Homo sapiens GN=SIRT3 PE=1 SV=2 - [SIRT3_HUMAN]	2,09	0,00
P10253	Lysosomal alpha-glucosidase OS=Homo sapiens GN=GAA PE=1 SV=4 - [LYAG_HUMAN]	2,30	0,00
P33897	ATP-binding cassette sub-family D member 1 OS=Homo sapiens GN=ABCD1 PE=1 SV=2 - [ABCD1_HUMAN]	3,55	0,00
Q8TB22-3	Isoform 3 of Spermatogenesis-associated protein 20 OS=Homo sapiens GN=SPATA20 - [SPT20_HUMAN]	2,69	0,00
Q7Z7M9	Polypeptide N-acetylgalactosaminyltransferase 5 OS=Homo sapiens GN=GALNT5 PE=1 SV=1 - [GALT5_HUMAN]	4,74	0,00
Q9Y6C2	EMILIN-1 OS=Homo sapiens GN=EMILIN1 PE=1 SV=2 - [EMIL1_HUMAN]	2,67	0,00
Q9BRX8-2	Isoform 2 of Redox-regulatory protein FAM213A OS=Homo sapiens GN=FAM213A - [F213A_HUMAN]	5,75	0,00
Q92673	Sortilin-related receptor OS=Homo sapiens GN=SORL1 PE=1 SV=2 - [SORL_HUMAN]	2,86	0,00
P49748-2	Isoform 2 of Very long-chain specific acyl-CoA dehydrogenase, mitochondrial OS=Homo sapiens GN=ACADVL - [ACA	3,25	0,00
P32004-2	Isoform 2 of Neural cell adhesion molecule L1 OS=Homo sapiens GN=L1CAM - [L1CAM_HUMAN]	4,57	0,00
Q9Y305-3	Isoform 3 of Acyl-coenzyme A thioesterase 9, mitochondrial OS=Homo sapiens GN=ACOT9 - [ACOT9_HUMAN]	2,48	0,00
Q15118	[Pyruvate dehydrogenase [lipoamide]] kinase isozyme 1, mitochondrial OS=Homo sapiens GN=PDK1 PE=1 SV=1 - [P	3,76	0,00

A375 vs. A375PR1

Q9ULI3	Protein HEG homolog 1 OS=Homo sapiens GN=HEG1 PE=1 SV=3 - [HEG1_HUMAN]	2,47	0,00
Q9UHK6	Alpha-methylacyl-CoA racemase OS=Homo sapiens GN=AMACR PE=1 SV=2 - [AMACR_HUMAN]	5,32	0,00
Q6ZMZ3	Nesprin-3 OS=Homo sapiens GN=C14orf49 PE=1 SV=2 - [SYNE3_HUMAN]	2,09	0,00
Q8NFV4-4	Isoform 4 of Abhydrolase domain-containing protein 11 OS=Homo sapiens GN=ABHD11 - [ABHDB_HUMAN]	1,98	0,00
Q70UQ0	Inhibitor of nuclear factor kappa-B kinase-interacting protein OS=Homo sapiens GN=IKBIP PE=1 SV=1 - [IKIP_HUMA	2,26	0,00
O15484	Calpain-5 OS=Homo sapiens GN=CAPN5 PE=1 SV=2 - [CAN5_HUMAN]	2,59	0,00
Q5T3J3-2	Isoform 2 of Ligand-dependent nuclear receptor-interacting factor 1 OS=Homo sapiens GN=LRIF1 - [LRIF1_HUMAN]	2,01	0,00
Q9BQE5	Apolipoprotein L2 OS=Homo sapiens GN=APOL2 PE=1 SV=1 - [APOL2_HUMAN]	3,07	0,00
Q13636	Ras-related protein Rab-31 OS=Homo sapiens GN=RAB31 PE=1 SV=1 - [RAB31_HUMAN]	2,18	0,00
Q16134	Electron transfer flavoprotein-ubiquinone oxidoreductase, mitochondrial OS=Homo sapiens GN=ETFDH PE=1 SV=2 -	2,18	0,00
Q8TB37-2	Isoform 2 of Iron-sulfur protein NUBPL OS=Homo sapiens GN=NUBPL - [NUBPL_HUMAN]	2,21	0,00
Q3KR37-2	Isoform 2 of GRAM domain-containing protein 1B OS=Homo sapiens GN=GRAMD1B - [GRM1B_HUMAN]	4,85	0,00
O60353	Frizzled-6 OS=Homo sapiens GN=FZD6 PE=1 SV=2 - [FZD6_HUMAN]	2,11	0,00
P21589	5'-nucleotidase OS=Homo sapiens GN=NT5E PE=1 SV=1 - [5NTD_HUMAN]	2,42	0,00
Q15120	[Pyruvate dehydrogenase [lipoamide]] kinase isozyme 3, mitochondrial OS=Homo sapiens GN=PDK3 PE=1 SV=1 - [P	2,86	0,00
P53794	Sodium/myo-inositol cotransporter OS=Homo sapiens GN=SLC5A3 PE=2 SV=2 - [SC5A3_HUMAN]	2,70	0,00
Q13488	V-type proton ATPase 116 kDa subunit a isoform 3 OS=Homo sapiens GN=TCIRG1 PE=1 SV=3 - [VPP3_HUMAN]	1,87	0,00
Q5T5S1	Uncharacterized coiled-coil domain-containing protein KIAA1984 OS=Homo sapiens GN=KIAA1984 PE=2 SV=3 - [K1	3,73	0,00
P08294	Extracellular superoxide dismutase [Cu-Zn] OS=Homo sapiens GN=SOD3 PE=1 SV=2 - [SODE_HUMAN]	4,67	0,00
Q9HBH5	Retinol dehydrogenase 14 OS=Homo sapiens GN=RDH14 PE=1 SV=1 - [RDH14_HUMAN]	2,07	0,00
Q9UBS9	Protein osteopotentia homolog OS=Homo sapiens GN=C1orf9 PE=2 SV=1 - [OSPT_HUMAN]	2,63	0,00
Q96AQ6-2	Isoform 2 of Pre-B-cell leukemia transcription factor-interacting protein 1 OS=Homo sapiens GN=PBXIP1 - [PBIP1_H	2,31	0,00
Q9UQ53-2	Isoform 2 of Alpha-1,3-mannosyl-glycoprotein 4-beta-N-acetylglucosaminyltransferase B OS=Homo sapiens GN=M	2,36	0,00
Q9NRW1	Ras-related protein Rab-6B OS=Homo sapiens GN=RAB6B PE=1 SV=1 - [RAB6B_HUMAN]	1,91	0,00
Q53TN4	Cytochrome b reductase 1 OS=Homo sapiens GN=CYBRD1 PE=1 SV=1 - [CYBR1_HUMAN]	2,89	0,00
Q7Z402	Transmembrane channel-like protein 7 OS=Homo sapiens GN=TMC7 PE=2 SV=1 - [TMC7_HUMAN]	2,16	0,00
P40616	ADP-ribosylation factor-like protein 1 OS=Homo sapiens GN=ARL1 PE=1 SV=1 - [ARL1_HUMAN]	1,85	0,00
Q02809	Procollagen-lysine,2-oxoglutarate 5-dioxygenase 1 OS=Homo sapiens GN=PLOD1 PE=1 SV=2 - [PLOD1_HUMAN]	2,17	0,00
P07099	Epoxide hydrolase 1 OS=Homo sapiens GN=EPHX1 PE=1 SV=1 - [HYEP_HUMAN]	2,93	0,00
Q99536	Synaptic vesicle membrane protein VAT-1 homolog OS=Homo sapiens GN=VAT1 PE=1 SV=2 - [VAT1_HUMAN]	2,01	0,00
P01033	Metalloproteinase inhibitor 1 OS=Homo sapiens GN=TIMP1 PE=1 SV=1 - [TIMP1_HUMAN]	1,97	0,00
Q9H1J7	Protein Wnt-5b OS=Homo sapiens GN=WNT5B PE=2 SV=2 - [WNT5B_HUMAN]	2,92	0,00
O00220	Tumor necrosis factor receptor superfamily member 10A OS=Homo sapiens GN=TNFRSF10A PE=1 SV=3 - [TR10A_H	2,05	0,00

A375 vs. A375PR1

Q12983	BCL2/adenovirus E1B 19 kDa protein-interacting protein 3 OS=Homo sapiens GN=BNIP3 PE=1 SV=2 - [BNIP3_HUMA	6,84	0,00
Q7L3T8	Probable proline--tRNA ligase, mitochondrial OS=Homo sapiens GN=PARS2 PE=1 SV=1 - [SYPM_HUMAN]	2,11	0,00
Q9H2D1	Mitochondrial folate transporter/carrier OS=Homo sapiens GN=SLC25A32 PE=1 SV=2 - [MFTC_HUMAN]	2,10	0,00
P55290	Cadherin-13 OS=Homo sapiens GN=CDH13 PE=1 SV=1 - [CAD13_HUMAN]	3,92	0,00
Q9H568	Actin-like protein 8 OS=Homo sapiens GN=ACTL8 PE=2 SV=1 - [ACTL8_HUMAN]	2,71	0,00
Q7Z2K6	Endoplasmic reticulum metallopeptidase 1 OS=Homo sapiens GN=ERMP1 PE=1 SV=2 - [ERMP1_HUMAN]	1,91	0,00
O00194	Ras-related protein Rab-27B OS=Homo sapiens GN=RAB27B PE=1 SV=4 - [RB27B_HUMAN]	3,88	0,00
Q70UQ0-4	Isoform 4 of Inhibitor of nuclear factor kappa-B kinase-interacting protein OS=Homo sapiens GN=IKBIP - [IKIP_HUM	2,03	0,00
Q9HBA0-4	Isoform 4 of Transient receptor potential cation channel subfamily V member 4 OS=Homo sapiens GN=TRPV4 - [TRI	2,69	0,00
P98155-2	Isoform Short of Very low-density lipoprotein receptor OS=Homo sapiens GN=VLDLR - [VLDLR_HUMAN]	3,16	0,00
P08581	Hepatocyte growth factor receptor OS=Homo sapiens GN=MET PE=1 SV=4 - [MET_HUMAN]	2,12	0,00
P35914	Hydroxymethylglutaryl-CoA lyase, mitochondrial OS=Homo sapiens GN=HMGCL PE=1 SV=2 - [HMGCL_HUMAN]	2,77	0,00
Q86SJ2	Amphoterin-induced protein 2 OS=Homo sapiens GN=AMIGO2 PE=1 SV=1 - [AMGO2_HUMAN]	2,10	0,00
Q6P5R6	60S ribosomal protein L22-like 1 OS=Homo sapiens GN=RPL22L1 PE=1 SV=2 - [RL22L_HUMAN]	3,22	0,00
Q8IX05	CD302 antigen OS=Homo sapiens GN=CD302 PE=1 SV=1 - [CD302_HUMAN]	2,15	0,00
Q7Z698	Sprouty-related, EVH1 domain-containing protein 2 OS=Homo sapiens GN=SPRED2 PE=1 SV=2 - [SPRE2_HUMAN]	1,73	0,00
P10301	Ras-related protein R-Ras OS=Homo sapiens GN=RRAS PE=1 SV=1 - [RRAS_HUMAN]	1,72	0,00
Q5FBB7-6	Isoform 6 of Shugoshin-like 1 OS=Homo sapiens GN=SGOL1 - [SGOL1_HUMAN]	1,71	0,00
Q969S9-2	Isoform 2 of Ribosome-releasing factor 2, mitochondrial OS=Homo sapiens GN=GFM2 - [RRF2M_HUMAN]	1,85	0,00
P17936	Insulin-like growth factor-binding protein 3 OS=Homo sapiens GN=IGFBP3 PE=1 SV=2 - [IBP3_HUMAN]	2,95	0,00
Q6ZMK1-2	Isoform 2 of Cysteine and histidine-rich protein 1 OS=Homo sapiens GN=CYHR1 - [CYHR1_HUMAN]	2,54	0,00
Q04941	Proteolipid protein 2 OS=Homo sapiens GN=PLP2 PE=1 SV=1 - [PLP2_HUMAN]	1,79	0,00
Q6IC98	GRAM domain-containing protein 4 OS=Homo sapiens GN=GRAMD4 PE=1 SV=1 - [GRAM4_HUMAN]	2,23	0,00
P27701	CD82 antigen OS=Homo sapiens GN=CD82 PE=1 SV=1 - [CD82_HUMAN]	3,64	0,00
O15460	Prolyl 4-hydroxylase subunit alpha-2 OS=Homo sapiens GN=P4HA2 PE=1 SV=1 - [P4HA2_HUMAN]	3,78	0,00
Q9NWU1	3-oxoacyl-[acyl-carrier-protein] synthase, mitochondrial OS=Homo sapiens GN=OXSM PE=1 SV=1 - [OXSM_HUMAN	1,92	0,00
Q96BS2	Tescalcin OS=Homo sapiens GN=TESC PE=1 SV=3 - [TESC_HUMAN]	2,69	0,00
P14373-2	Isoform Beta of Zinc finger protein RFP OS=Homo sapiens GN=TRIM27 - [TRI27_HUMAN]	2,00	0,00
O15240	Neurosecretory protein VGF OS=Homo sapiens GN=VGF PE=1 SV=2 - [VGF_HUMAN]	314,21	0,00
Q96B96	Promethin OS=Homo sapiens GN=TMEM159 PE=2 SV=2 - [TM159_HUMAN]	1,68	0,00
O00562-2	Isoform 2 of Membrane-associated phosphatidylinositol transfer protein 1 OS=Homo sapiens GN=PITPNM1 - [PITM	2,44	0,00
Q9BXJ8-2	Isoform 2 of Transmembrane protein 120A OS=Homo sapiens GN=TMEM120A - [T120A_HUMAN]	1,76	0,00
P08174	Complement decay-accelerating factor OS=Homo sapiens GN=CD55 PE=1 SV=4 - [DAF_HUMAN]	2,06	0,00

A375 vs. A375PR1

Q53R41-2	Isoform 2 of FAST kinase domain-containing protein 1 OS=Homo sapiens GN=FASTKD1 - [FAKD1_HUMAN]	2,50	0,00
O14880	Microsomal glutathione S-transferase 3 OS=Homo sapiens GN=MGST3 PE=1 SV=1 - [MGST3_HUMAN]	2,41	0,00
Q8WZA1	Protein O-linked-mannose beta-1,2-N-acetylglucosaminyltransferase 1 OS=Homo sapiens GN=POMGNT1 PE=1 SV=2	1,76	0,00
Q9UQ03-2	Isoform 2 of Coronin-2B OS=Homo sapiens GN=CORO2B - [COR2B_HUMAN]	2,24	0,00
Q9BZC7-2	Isoform 2 of ATP-binding cassette sub-family A member 2 OS=Homo sapiens GN=ABCA2 - [ABCA2_HUMAN]	1,90	0,00
Q9UHQ9	NADH-cytochrome b5 reductase 1 OS=Homo sapiens GN=CYB5R1 PE=1 SV=1 - [NB5R1_HUMAN]	1,90	0,00
Q96N66-3	Isoform 3 of Lysophospholipid acyltransferase 7 OS=Homo sapiens GN=MBOAT7 - [MBOA7_HUMAN]	2,17	0,00
Q7L311	Armadillo repeat-containing X-linked protein 2 OS=Homo sapiens GN=ARMCX2 PE=2 SV=1 - [ARMX2_HUMAN]	1,75	0,00
Q8IWB1	Inositol 1,4,5-triphosphate receptor-interacting protein OS=Homo sapiens GN=ITPRIP PE=1 SV=1 - [IPRI_HUMAN]	2,49	0,00
Q9BYC5	Alpha-(1,6)-fucosyltransferase OS=Homo sapiens GN=FUT8 PE=1 SV=2 - [FUT8_HUMAN]	1,94	0,00
P55268	Laminin subunit beta-2 OS=Homo sapiens GN=LAMB2 PE=1 SV=2 - [LAMB2_HUMAN]	2,47	0,00
Q8N474	Secreted frizzled-related protein 1 OS=Homo sapiens GN=SFRP1 PE=1 SV=1 - [SFRP1_HUMAN]	1,80	0,00
Q8WUJ3-2	Isoform 2 of Protein KIAA1199 OS=Homo sapiens GN=KIAA1199 - [K1199_HUMAN]	3,26	0,00
Q9NWQ8	Phosphoprotein associated with glycosphingolipid-enriched microdomains 1 OS=Homo sapiens GN=PAG1 PE=1 SV=	1,86	0,00
P07858	Cathepsin B OS=Homo sapiens GN=CTSB PE=1 SV=3 - [CATB_HUMAN]	2,23	0,00
P58335-4	Isoform 4 of Anthrax toxin receptor 2 OS=Homo sapiens GN=ANTXR2 - [ANTR2_HUMAN]	1,84	0,00
Q16394	Exostosin-1 OS=Homo sapiens GN=EXT1 PE=1 SV=2 - [EXT1_HUMAN]	2,10	0,00
P01130	Low-density lipoprotein receptor OS=Homo sapiens GN=LDLR PE=1 SV=1 - [LDLR_HUMAN]	1,91	0,00
Q3T906	N-acetylglucosamine-1-phosphotransferase subunits alpha/beta OS=Homo sapiens GN=GNPTAB PE=1 SV=1 - [GNPT	2,04	0,00
Q07065	Cytoskeleton-associated protein 4 OS=Homo sapiens GN=CKAP4 PE=1 SV=2 - [CKAP4_HUMAN]	1,81	0,00
Q86WA9	Sodium-independent sulfate anion transporter OS=Homo sapiens GN=SLC26A11 PE=1 SV=2 - [S2611_HUMAN]	1,98	0,00
P55084	Trifunctional enzyme subunit beta, mitochondrial OS=Homo sapiens GN=HADHB PE=1 SV=3 - [ECHB_HUMAN]	1,87	0,00
O00469-2	Isoform 2 of Procollagen-lysine,2-oxoglutarate 5-dioxygenase 2 OS=Homo sapiens GN=PLOD2 - [PLOD2_HUMAN]	2,56	0,00
P27144	Adenylate kinase isoenzyme 4, mitochondrial OS=Homo sapiens GN=AK4 PE=1 SV=1 - [KAD4_HUMAN]	1,92	0,00
P23786	Carnitine O-palmitoyltransferase 2, mitochondrial OS=Homo sapiens GN=CPT2 PE=1 SV=2 - [CPT2_HUMAN]	1,80	0,00
Q8N4T8-2	Isoform 2 of Carbonyl reductase family member 4 OS=Homo sapiens GN=CBR4 - [CBR4_HUMAN]	1,87	0,00
O14735	CDP-diacylglycerol--inositol 3-phosphatidyltransferase OS=Homo sapiens GN=CDIPT PE=1 SV=1 - [CDIPT_HUMAN]	1,70	0,00
Q5RI15	Protein FAM36A OS=Homo sapiens GN=FAM36A PE=1 SV=2 - [FA36A_HUMAN]	1,67	0,00
O95870	Abhydrolase domain-containing protein 16A OS=Homo sapiens GN=ABHD16A PE=1 SV=3 - [ABHGA_HUMAN]	1,75	0,00
P43121	Cell surface glycoprotein MUC18 OS=Homo sapiens GN=MCAM PE=1 SV=2 - [MUC18_HUMAN]	1,92	0,00
Q96D53	Uncharacterized aarF domain-containing protein kinase 4 OS=Homo sapiens GN=ADCK4 PE=1 SV=2 - [ADCK4_HUM	1,75	0,00
P43362	Melanoma-associated antigen 9 OS=Homo sapiens GN=MAGEA9 PE=2 SV=1 - [MAGA9_HUMAN]	1,84	0,00
Q8TD43-2	Isoform 2 of Transient receptor potential cation channel subfamily M member 4 OS=Homo sapiens GN=TRPM4 - [T	1,94	0,00

A375 vs. A375PR1

Q16363-2	Isoform 2 of Laminin subunit alpha-4 OS=Homo sapiens GN=LAMA4 - [LAMA4_HUMAN]	2,24	0,00
Q9BZG1	Ras-related protein Rab-34 OS=Homo sapiens GN=RAB34 PE=1 SV=1 - [RAB34_HUMAN]	1,79	0,00
Q6NXT6	Transmembrane anterior posterior transformation protein 1 homolog OS=Homo sapiens GN=TAPT1 PE=1 SV=1 - [T/	2,14	0,00
O95573	Long-chain-fatty-acid--CoA ligase 3 OS=Homo sapiens GN=ACSL3 PE=1 SV=3 - [ACSL3_HUMAN]	2,13	0,00
P06858	Lipoprotein lipase OS=Homo sapiens GN=LPL PE=1 SV=1 - [LIPL_HUMAN]	2,30	0,00
Q96AQ8	Coiled-coil domain-containing protein 90A, mitochondrial OS=Homo sapiens GN=CCDC90A PE=2 SV=1 - [CC90A_HU	1,88	0,00
Q15526-2	Isoform 2 of Surfeit locus protein 1 OS=Homo sapiens GN=SURF1 - [SURF1_HUMAN]	1,89	0,00
P23368	NAD-dependent malic enzyme, mitochondrial OS=Homo sapiens GN=ME2 PE=1 SV=1 - [MAOM_HUMAN]	1,97	0,00
O95571	Protein ETHE1, mitochondrial OS=Homo sapiens GN=ETHE1 PE=1 SV=2 - [ETHE1_HUMAN]	1,86	0,00
Q9BXT8-2	Isoform 3 of RING finger protein 17 OS=Homo sapiens GN=RNF17 - [RNF17_HUMAN]	1,97	0,00
P42126-2	Isoform 2 of Enoyl-CoA delta isomerase 1, mitochondrial OS=Homo sapiens GN=ECI1 - [ECI1_HUMAN]	1,89	0,00
Q13427	Peptidyl-prolyl cis-trans isomerase G OS=Homo sapiens GN=PPIG PE=1 SV=2 - [PPIG_HUMAN]	1,81	0,00
Q6STE5-2	Isoform 2 of SWI/SNF-related matrix-associated actin-dependent regulator of chromatin subfamily D member 3 OS=	1,86	0,00
Q02978	Mitochondrial 2-oxoglutarate/malate carrier protein OS=Homo sapiens GN=SLC25A11 PE=1 SV=3 - [M2OM_HUMA	2,07	0,00
Q86UE4	Protein LYRIC OS=Homo sapiens GN=MTDH PE=1 SV=2 - [LYRIC_HUMAN]	1,82	0,00
Q9P2E5-2	Isoform 2 of Chondroitin sulfate glucuronyltransferase OS=Homo sapiens GN=CHPF2 - [CHPF2_HUMAN]	1,75	0,00
Q96C36	Pyrroline-5-carboxylate reductase 2 OS=Homo sapiens GN=PYCR2 PE=1 SV=1 - [P5CR2_HUMAN]	2,02	0,00
Q8NBQ5	Estradiol 17-beta-dehydrogenase 11 OS=Homo sapiens GN=HSD17B11 PE=1 SV=3 - [DHB11_HUMAN]	1,77	0,00
P26006	Integrin alpha-3 OS=Homo sapiens GN=ITGA3 PE=1 SV=5 - [ITA3_HUMAN]	2,17	0,00
Q9BU23-3	Isoform 3 of Lipase maturation factor 2 OS=Homo sapiens GN=LMF2 - [LMF2_HUMAN]	1,63	0,00
Q13393-4	Isoform PLD1D of Phospholipase D1 OS=Homo sapiens GN=PLD1 - [PLD1_HUMAN]	1,94	0,00
Q9UH62	Armadillo repeat-containing X-linked protein 3 OS=Homo sapiens GN=ARMCX3 PE=1 SV=1 - [ARMX3_HUMAN]	1,84	0,00
Q13586	Stromal interaction molecule 1 OS=Homo sapiens GN=STIM1 PE=1 SV=3 - [STIM1_HUMAN]	1,63	0,00
P61020	Ras-related protein Rab-5B OS=Homo sapiens GN=RAB5B PE=1 SV=1 - [RAB5B_HUMAN]	1,81	0,00
Q92805	Golgin subfamily A member 1 OS=Homo sapiens GN=GOLGA1 PE=1 SV=3 - [GOGA1_HUMAN]	1,81	0,00
Q9UDX5	Mitochondrial fission process protein 1 OS=Homo sapiens GN=MTFP1 PE=1 SV=1 - [MTFP1_HUMAN]	1,74	0,00
Q14573	Inositol 1,4,5-trisphosphate receptor type 3 OS=Homo sapiens GN=ITPR3 PE=1 SV=2 - [ITPR3_HUMAN]	1,80	0,00
Q8NBX0	Saccharopine dehydrogenase-like oxidoreductase OS=Homo sapiens GN=SCCPDH PE=1 SV=1 - [SCPDL_HUMAN]	1,61	0,00
Q15149-5	Isoform 5 of Plectin OS=Homo sapiens GN=PLEC - [PLEC_HUMAN]	2,82	0,00
P14927	Cytochrome b-c1 complex subunit 7 OS=Homo sapiens GN=UQCRB PE=1 SV=2 - [QCR7_HUMAN]	1,65	0,00
Q8N441	Fibroblast growth factor receptor-like 1 OS=Homo sapiens GN=FGFRL1 PE=1 SV=1 - [FGRL1_HUMAN]	2,08	0,00
O75110	Probable phospholipid-transporting ATPase IIA OS=Homo sapiens GN=ATP9A PE=1 SV=3 - [ATP9A_HUMAN]	1,77	0,00
Q14728	Major facilitator superfamily domain-containing protein 10 OS=Homo sapiens GN=MFSD10 PE=2 SV=1 - [MFS10_HU	1,63	0,00

A375 vs. A375PR1

P07093-2	Isoform 2 of Glia-derived nexin OS=Homo sapiens GN=SERPINE2 - [GDN_HUMAN]	1,63	0,00
Q8TCT9-5	Isoform 5 of Minor histocompatibility antigen H13 OS=Homo sapiens GN=HM13 - [HM13_HUMAN]	1,86	0,00
Q9H0X9	Oxysterol-binding protein-related protein 5 OS=Homo sapiens GN=OSBPL5 PE=1 SV=1 - [OSBL5_HUMAN]	1,63	0,00
Q2PZI1-2	Isoform 2 of Protein dpy-19 homolog 1 OS=Homo sapiens GN=DPY19L1 - [D19L1_HUMAN]	2,00	0,00
Q9HD26-2	Isoform 2 of Golgi-associated PDZ and coiled-coil motif-containing protein OS=Homo sapiens GN=GOPC - [GOPC_HU	1,84	0,00
Q6P2I3	Fumarylacetoacetate hydrolase domain-containing protein 2B OS=Homo sapiens GN=FAHD2B PE=1 SV=1 - [FAH2B_	1,82	0,00
Q99541	Perilipin-2 OS=Homo sapiens GN=PLIN2 PE=1 SV=2 - [PLIN2_HUMAN]	1,78	0,00
Q2TB10	Zinc finger protein 800 OS=Homo sapiens GN=ZNF800 PE=1 SV=1 - [ZN800_HUMAN]	1,73	0,00
Q08379	Golgin subfamily A member 2 OS=Homo sapiens GN=GOLGA2 PE=1 SV=3 - [GOGA2_HUMAN]	1,70	0,00
O60906	Sphingomyelin phosphodiesterase 2 OS=Homo sapiens GN=SMPD2 PE=1 SV=2 - [NSMA_HUMAN]	1,84	0,00
O14949	Cytochrome b-c1 complex subunit 8 OS=Homo sapiens GN=UQCRQ PE=1 SV=4 - [QCR8_HUMAN]	1,57	0,00
Q8IXB1-2	Isoform 2 of DnaJ homolog subfamily C member 10 OS=Homo sapiens GN=DNAJC10 - [DJC10_HUMAN]	1,65	0,00
Q92520	Protein FAM3C OS=Homo sapiens GN=FAM3C PE=1 SV=1 - [FAM3C_HUMAN]	1,78	0,00
O43824	Putative GTP-binding protein 6 OS=Homo sapiens GN=GTPBP6 PE=2 SV=3 - [GTPB6_HUMAN]	2,38	0,00
Q08174-2	Isoform 2 of Protocadherin-1 OS=Homo sapiens GN=PCDH1 - [PCDH1_HUMAN]	1,71	0,00
Q8IZV5	Retinol dehydrogenase 10 OS=Homo sapiens GN=RDH10 PE=1 SV=1 - [RDH10_HUMAN]	1,79	0,00
Q8IXT5	RNA-binding protein 12B OS=Homo sapiens GN=RBM12B PE=1 SV=2 - [RB12B_HUMAN]	1,70	0,00
Q9BVA6	Adenosine monophosphate-protein transferase FICD OS=Homo sapiens GN=FICD PE=1 SV=2 - [FICD_HUMAN]	1,88	0,00
P07711	Cathepsin L1 OS=Homo sapiens GN=CTSL1 PE=1 SV=2 - [CATL1_HUMAN]	2,14	0,00
Q9Y3B3	Transmembrane emp24 domain-containing protein 7 OS=Homo sapiens GN=TMED7 PE=1 SV=2 - [TMED7_HUMAN]	1,62	0,00
Q14993	Collagen alpha-1(XIX) chain OS=Homo sapiens GN=COL19A1 PE=1 SV=3 - [COJA1_HUMAN]	2,26	0,00
Q96ND0	Protein FAM210A OS=Homo sapiens GN=FAM210A PE=2 SV=2 - [F210A_HUMAN]	1,80	0,00
Q9UPA5	Protein bassoon OS=Homo sapiens GN=BSN PE=1 SV=4 - [BSN_HUMAN]	70500608897,67	0,00
Q6PKC3-2	Isoform 2 of Thioredoxin domain-containing protein 11 OS=Homo sapiens GN=TXNDC11 - [TXD11_HUMAN]	1,61	0,00
P48723	Heat shock 70 kDa protein 13 OS=Homo sapiens GN=HSPA13 PE=1 SV=1 - [HSP13_HUMAN]	1,86	0,00
Q9NR19	Acetyl-coenzyme A synthetase, cytoplasmic OS=Homo sapiens GN=ACSS2 PE=1 SV=1 - [ACSA_HUMAN]	2,25	0,00
P84157-2	Isoform 2 of Matrix-remodeling-associated protein 7 OS=Homo sapiens GN=MXRA7 - [MXRA7_HUMAN]	1,69	0,00
Q9UMF0	Intercellular adhesion molecule 5 OS=Homo sapiens GN=ICAM5 PE=1 SV=3 - [ICAM5_HUMAN]	1,90	0,00
P05091	Aldehyde dehydrogenase, mitochondrial OS=Homo sapiens GN=ALDH2 PE=1 SV=2 - [ALDH2_HUMAN]	1,73	0,00
Q8WY21-3	Isoform 3 of VPS10 domain-containing receptor SorCS1 OS=Homo sapiens GN=SORCS1 - [SORC1_HUMAN]	2,38	0,00
O00154-6	Isoform 6 of Cytosolic acyl coenzyme A thioester hydrolase OS=Homo sapiens GN=ACOT7 - [BACH_HUMAN]	1,80	0,00
O43166-2	Isoform 2 of Signal-induced proliferation-associated 1-like protein 1 OS=Homo sapiens GN=SIPA1L1 - [SI1L1_HUMA	1,86	0,00
Q9Y653-2	Isoform 2 of G-protein coupled receptor 56 OS=Homo sapiens GN=GPR56 - [GPR56_HUMAN]	3,17	0,00

Q5TF58	Intermediate filament family orphan 2 OS=Homo sapiens GN=IFFO2 PE=2 SV=3 - [IFFO2_HUMAN]	1,61	0,00
P41219	Peripherin OS=Homo sapiens GN=PRPH PE=1 SV=2 - [PERI_HUMAN]	1960125726426,86	0,00
Q9HBL7	Transmembrane protein C9orf46 OS=Homo sapiens GN=C9orf46 PE=2 SV=1 - [CI046_HUMAN]	1,63	0,00
P04156-2	Isoform 2 of Major prion protein OS=Homo sapiens GN=PRNP - [PRIO_HUMAN]	1,60	0,00
P28290-2	Isoform 2 of Sperm-specific antigen 2 OS=Homo sapiens GN=SSFA2 - [SSFA2_HUMAN]	1,72	0,00
P62760	Visinin-like protein 1 OS=Homo sapiens GN=VSNL1 PE=1 SV=2 - [VISL1_HUMAN]	2,36	0,00
Q9NZV5-2	Isoform 2 of Selenoprotein N OS=Homo sapiens GN=SEPN1 - [SELN_HUMAN]	1,59	0,00
Q8N0V3	Putative ribosome-binding factor A, mitochondrial OS=Homo sapiens GN=RBFA PE=1 SV=3 - [RBFA_HUMAN]	1,72	0,00
P19256-2	Isoform 2 of Lymphocyte function-associated antigen 3 OS=Homo sapiens GN=CD58 - [LFA3_HUMAN]	1,56	0,00
Q9NUJ1	Abhydrolase domain-containing protein 10, mitochondrial OS=Homo sapiens GN=ABHD10 PE=1 SV=1 - [ABHDA_HU	1,98	0,00
P22033	Methylmalonyl-CoA mutase, mitochondrial OS=Homo sapiens GN=MUT PE=1 SV=4 - [MUTA_HUMAN]	1,80	0,00
Q8N9Z2	Uncharacterized protein C7orf74 OS=Homo sapiens GN=C7orf74 PE=2 SV=2 - [CG074_HUMAN]	1,64	0,00
Q9BTZ2-5	Isoform 5 of Dehydrogenase/reductase SDR family member 4 OS=Homo sapiens GN=DHRS4 - [DHRS4_HUMAN]	1,89	0,00
Q8N8U2	Chromodomain Y-like protein 2 OS=Homo sapiens GN=CDYL2 PE=2 SV=2 - [CDYL2_HUMAN]	2,09	0,00
Q9UHG3	Prenylcysteine oxidase 1 OS=Homo sapiens GN=PCYOX1 PE=1 SV=3 - [PCYOX_HUMAN]	1,83	0,00
Q9Y5J7	Mitochondrial import inner membrane translocase subunit Tim9 OS=Homo sapiens GN=TIMM9 PE=1 SV=1 - [TIM9_	1,70	0,00
Q9C0E8-2	Isoform 2 of Protein lunapark OS=Homo sapiens GN=LNP - [LNP_HUMAN]	1,70	0,00
P40939	Trifunctional enzyme subunit alpha, mitochondrial OS=Homo sapiens GN=HADHA PE=1 SV=2 - [ECHA_HUMAN]	1,66	0,00
Q8IYQ7	Threonine synthase-like 1 OS=Homo sapiens GN=THNSL1 PE=1 SV=2 - [THNS1_HUMAN]	1,59	0,00
Q5HYI7	Metaxin-3 OS=Homo sapiens GN=MTX3 PE=1 SV=2 - [MTX3_HUMAN]	1,60	0,00
Q9Y4K0	Lysyl oxidase homolog 2 OS=Homo sapiens GN=LOXL2 PE=1 SV=1 - [LOXL2_HUMAN]	1,79	0,00
Q02252	Methylmalonate-semialdehyde dehydrogenase [acylating], mitochondrial OS=Homo sapiens GN=ALDH6A1 PE=1 SV	2,46	0,00
Q9NQE9	Histidine triad nucleotide-binding protein 3 OS=Homo sapiens GN=HINT3 PE=1 SV=1 - [HINT3_HUMAN]	1,57	0,00
P53007	Tricarboxylate transport protein, mitochondrial OS=Homo sapiens GN=SLC25A1 PE=1 SV=2 - [TXTP_HUMAN]	1,57	0,00
Q68DH5	LMBR1 domain-containing protein 2 OS=Homo sapiens GN=LMBRD2 PE=1 SV=1 - [LMBD2_HUMAN]	1,60	0,00
Q9BZ76	Contactin-associated protein-like 3 OS=Homo sapiens GN=CNTNAP3 PE=2 SV=3 - [CNTP3_HUMAN]	1,59	0,00
Q58EX7-2	Isoform 2 of Puratrophin-1 OS=Homo sapiens GN=PLEKHG4 - [PKHG4_HUMAN]	1,90	0,00
Q96RQ3	Methylcrotonoyl-CoA carboxylase subunit alpha, mitochondrial OS=Homo sapiens GN=MCCC1 PE=1 SV=3 - [MCCA_	1,92	0,00
Q9BUR5-2	Isoform 2 of Apolipoprotein O OS=Homo sapiens GN=APOO - [APOO_HUMAN]	1,61	0,00
Q9BVV7	Mitochondrial import inner membrane translocase subunit Tim21 OS=Homo sapiens GN=TIMM21 PE=1 SV=1 - [TIM	1,56	0,00
O43293	Death-associated protein kinase 3 OS=Homo sapiens GN=DAPK3 PE=1 SV=1 - [DAPK3_HUMAN]	1,65	0,00
Q8N4A0	Polypeptide N-acetylgalactosaminyltransferase 4 OS=Homo sapiens GN=GALNT4 PE=1 SV=2 - [GALT4_HUMAN]	1,83	0,00
Q15011-3	Isoform 3 of Homocysteine-responsive endoplasmic reticulum-resident ubiquitin-like domain member 1 protein OS	1,59	0,00

A375 vs. A375PR1

Q8N2G8	GH3 domain-containing protein OS=Homo sapiens GN=GHDC PE=1 SV=2 - [GHDC_HUMAN]	1,46	0,00
Q15392	Delta(24)-sterol reductase OS=Homo sapiens GN=DHCR24 PE=1 SV=2 - [DHC24_HUMAN]	2,31	0,00
Q86VU5	Catechol O-methyltransferase domain-containing protein 1 OS=Homo sapiens GN=COMTD1 PE=1 SV=1 - [CMTD1_H	1,67	0,00
P08574	Cytochrome c1, heme protein, mitochondrial OS=Homo sapiens GN=CYC1 PE=1 SV=3 - [CY1_HUMAN]	1,78	0,00
P50336	Protoporphyrinogen oxidase OS=Homo sapiens GN=PPOX PE=1 SV=1 - [PPOX_HUMAN]	1,58	0,00
Q15149-6	Isoform 6 of Plectin OS=Homo sapiens GN=PLEC - [PLEC_HUMAN]	1,95	0,00
Q3SXM5	Inactive hydroxysteroid dehydrogenase-like protein 1 OS=Homo sapiens GN=HSDL1 PE=1 SV=3 - [HSDL1_HUMAN]	2,11	0,00
Q6KCM7-6	Isoform 6 of Calcium-binding mitochondrial carrier protein SCaMC-2 OS=Homo sapiens GN=SLC25A25 - [SCMC2_HU	1,53	0,00
Q9BYT1-2	Isoform 2 of Solute carrier family 17 member 9 OS=Homo sapiens GN=SLC17A9 - [S17A9_HUMAN]	2,33	0,00
Q8TDW0	Leucine-rich repeat-containing protein 8C OS=Homo sapiens GN=LRRC8C PE=1 SV=2 - [LRC8C_HUMAN]	1,84	0,00
O00258	Tail-anchored protein insertion receptor WRB OS=Homo sapiens GN=WRB PE=1 SV=2 - [WRB_HUMAN]	1,69	0,00
O94901	SUN domain-containing protein 1 OS=Homo sapiens GN=SUN1 PE=1 SV=3 - [SUN1_HUMAN]	1,65	0,00
Q8IWA4	Mitofusin-1 OS=Homo sapiens GN=MFN1 PE=1 SV=2 - [MFN1_HUMAN]	1,54	0,00
P02545-3	Isoform ADelta10 of Prelamin-A/C OS=Homo sapiens GN=LMNA - [LMNA_HUMAN]	1,66	0,00
Q71RG4-3	Isoform 3 of Transmembrane and ubiquitin-like domain-containing protein 2 OS=Homo sapiens GN=TMUB2 - [TMU	1,70	0,00
Q9NVH6-2	Isoform B of Trimethyllysine dioxygenase, mitochondrial OS=Homo sapiens GN=TMLHE - [TMLH_HUMAN]	1,71	0,00
P09471-2	Isoform Alpha-2 of Guanine nucleotide-binding protein G(o) subunit alpha OS=Homo sapiens GN=GNAO1 - [GNAO_	1,61	0,00
Q9ULX9	Transcription factor MafF OS=Homo sapiens GN=MAFF PE=1 SV=2 - [MAFF_HUMAN]	1,52	0,00
Q8WW59	SPRY domain-containing protein 4 OS=Homo sapiens GN=SPRYD4 PE=1 SV=2 - [SPRY4_HUMAN]	1,68	0,00
P20340	Ras-related protein Rab-6A OS=Homo sapiens GN=RAB6A PE=1 SV=3 - [RAB6A_HUMAN]	1,62	0,00
Q8IVL5	Prolyl 3-hydroxylase 2 OS=Homo sapiens GN=LEPREL1 PE=1 SV=1 - [P3H2_HUMAN]	1,66	0,00
Q53EP0	Fibronectin type III domain-containing protein 3B OS=Homo sapiens GN=FNDC3B PE=1 SV=2 - [FND3B_HUMAN]	1,66	0,00
O94905	Erlin-2 OS=Homo sapiens GN=ERLIN2 PE=1 SV=1 - [ERLN2_HUMAN]	1,55	0,00
P48681	Nestin OS=Homo sapiens GN=NES PE=1 SV=2 - [NEST_HUMAN]	1,64	0,00
Q9NRX5	Serine incorporator 1 OS=Homo sapiens GN=SERINC1 PE=1 SV=1 - [SERC1_HUMAN]	1,45	0,00
P37275	Zinc finger E-box-binding homeobox 1 OS=Homo sapiens GN=ZEB1 PE=1 SV=2 - [ZEB1_HUMAN]	1,65	0,00
P11117	Lysosomal acid phosphatase OS=Homo sapiens GN=ACP2 PE=1 SV=3 - [PPAL_HUMAN]	1,52	0,00
Q14807	Kinesin-like protein KIF22 OS=Homo sapiens GN=KIF22 PE=1 SV=5 - [KIF22_HUMAN]	1,54	0,00
P49590	Probable histidine--tRNA ligase, mitochondrial OS=Homo sapiens GN=HARS2 PE=1 SV=1 - [SYHM_HUMAN]	1,68	0,00
O94766	Galactosylgalactosylxylosylprotein 3-beta-glucuronosyltransferase 3 OS=Homo sapiens GN=B3GAT3 PE=1 SV=2 - [B3	1,59	0,00
Q9H0V1	Transmembrane protein 168 OS=Homo sapiens GN=TMEM168 PE=1 SV=2 - [TM168_HUMAN]	1,46	0,00
P18084	Integrin beta-5 OS=Homo sapiens GN=ITGB5 PE=1 SV=1 - [ITB5_HUMAN]	1,76	0,00
P13674	Prolyl 4-hydroxylase subunit alpha-1 OS=Homo sapiens GN=P4HA1 PE=1 SV=2 - [P4HA1_HUMAN]	2,02	0,00

A375 vs. A375PR1

P61165	UPF0197 transmembrane protein C11orf10 OS=Homo sapiens GN=C11orf10 PE=1 SV=1 - [CK010_HUMAN]	1,64	0,00
Q8IZR5-3	Isoform 3 of CKLF-like MARVEL transmembrane domain-containing protein 4 OS=Homo sapiens GN=CMTM4 - [CKLI	1,72	0,00
Q9Y6M9	NADH dehydrogenase [ubiquinone] 1 beta subcomplex subunit 9 OS=Homo sapiens GN=NDUFB9 PE=1 SV=3 - [NDU	1,67	0,00
Q8N5G0-2	Isoform 2 of Uncharacterized protein C4orf52 OS=Homo sapiens GN=C4orf52 - [CD052_HUMAN]	1,62	0,00
Q7Z388	Protein dpy-19 homolog 4 OS=Homo sapiens GN=DPY19L4 PE=2 SV=1 - [D19L4_HUMAN]	1,70	0,00
Q14764	Major vault protein OS=Homo sapiens GN=MVP PE=1 SV=4 - [MVP_HUMAN]	1,90	0,00
P61019	Ras-related protein Rab-2A OS=Homo sapiens GN=RAB2A PE=1 SV=1 - [RAB2A_HUMAN]	1,62	0,00
Q9BSA9	Transmembrane protein 175 OS=Homo sapiens GN=TMEM175 PE=1 SV=1 - [TM175_HUMAN]	1,79	0,00
Q8N2H3	Pyridine nucleotide-disulfide oxidoreductase domain-containing protein 2 OS=Homo sapiens GN=PYROXD2 PE=2 SV	2,05	0,00
Q8WVC6	Dephospho-CoA kinase domain-containing protein OS=Homo sapiens GN=DCAKD PE=1 SV=1 - [DCAKD_HUMAN]	1,66	0,00
Q14554	Protein disulfide-isomerase A5 OS=Homo sapiens GN=PDIA5 PE=1 SV=1 - [PDIA5_HUMAN]	1,80	0,00
Q14156-3	Isoform 3 of Protein EFR3 homolog A OS=Homo sapiens GN=EFR3A - [EFR3A_HUMAN]	1,46	0,00
A6NJ78	Probable methyltransferase-like protein 15 OS=Homo sapiens GN=METTL15 PE=2 SV=1 - [MET15_HUMAN]	1,58	0,00
Q6NXT4	Zinc transporter 6 OS=Homo sapiens GN=SLC30A6 PE=1 SV=2 - [ZNT6_HUMAN]	1,69	0,00
P19438	Tumor necrosis factor receptor superfamily member 1A OS=Homo sapiens GN=TNFRSF1A PE=1 SV=1 - [TNR1A_HUI	1,50	0,00
Q562F6-2	Isoform 2 of Shugoshin-like 2 OS=Homo sapiens GN=SGOL2 - [SGOL2_HUMAN]	2,38	0,00
P30084	Enoyl-CoA hydratase, mitochondrial OS=Homo sapiens GN=ECHS1 PE=1 SV=4 - [ECHM_HUMAN]	1,62	0,00
Q9Y394-2	Isoform 2 of Dehydrogenase/reductase SDR family member 7 OS=Homo sapiens GN=DHRS7 - [DHRS7_HUMAN]	1,70	0,00
Q96IR7	4-hydroxyphenylpyruvate dioxygenase-like protein OS=Homo sapiens GN=HPDL PE=1 SV=1 - [HPDL_HUMAN]	1,79	0,00
Q14534	Squalene monooxygenase OS=Homo sapiens GN=SQLE PE=2 SV=3 - [ERG1_HUMAN]	1,55	0,00
O14874	[3-methyl-2-oxobutanoate dehydrogenase [lipoamide]] kinase, mitochondrial OS=Homo sapiens GN=BCKDK PE=1 S	1,59	0,00
O15554	Intermediate conductance calcium-activated potassium channel protein 4 OS=Homo sapiens GN=KCNN4 PE=1 SV=1	1,61	0,00
O75964	ATP synthase subunit g, mitochondrial OS=Homo sapiens GN=ATP5L PE=1 SV=3 - [ATP5L_HUMAN]	1,62	0,00
P28331	NADH-ubiquinone oxidoreductase 75 kDa subunit, mitochondrial OS=Homo sapiens GN=NDUFS1 PE=1 SV=3 - [NDU	1,59	0,00
P49821-2	Isoform 2 of NADH dehydrogenase [ubiquinone] flavoprotein 1, mitochondrial OS=Homo sapiens GN=NDUFV1 - [NI	1,60	0,00
Q86TM6-2	Isoform 2 of E3 ubiquitin-protein ligase synoviolin OS=Homo sapiens GN=SYVN1 - [SYVN1_HUMAN]	1,64	0,00
Q4ZIN3-2	Isoform 2 of Membralin OS=Homo sapiens GN=C19orf6 - [MBRL_HUMAN]	1,47	0,00
Q8WWM9	Cytoglobin OS=Homo sapiens GN=CYGB PE=1 SV=1 - [CYGB_HUMAN]	2,16	0,00
Q15067	Peroxisomal acyl-coenzyme A oxidase 1 OS=Homo sapiens GN=ACOX1 PE=1 SV=3 - [ACOX1_HUMAN]	1,59	0,00
Q6PI48	Aspartate--tRNA ligase, mitochondrial OS=Homo sapiens GN=DARS2 PE=1 SV=1 - [SYDM_HUMAN]	1,60	0,00
Q9Y276	Mitochondrial chaperone BCS1 OS=Homo sapiens GN=BCS1L PE=1 SV=1 - [BCS1_HUMAN]	1,56	0,00
P17252	Protein kinase C alpha type OS=Homo sapiens GN=PRKCA PE=1 SV=4 - [KPCA_HUMAN]	1,80	0,00
Q9H5V8-2	Isoform 2 of CUB domain-containing protein 1 OS=Homo sapiens GN=CDCP1 - [CDCP1_HUMAN]	1,63	0,00

A375 vs. A375PR1

Q96LZ7	Regulator of microtubule dynamics protein 2 OS=Homo sapiens GN=FAM82A1 PE=1 SV=2 - [RMD2_HUMAN]	1,55	0,00
O60934	Nibrin OS=Homo sapiens GN=NBN PE=1 SV=1 - [NBN_HUMAN]	1,53	0,00
Q9BUE6	Iron-sulfur cluster assembly 1 homolog, mitochondrial OS=Homo sapiens GN=ISCA1 PE=2 SV=1 - [ISCA1_HUMAN]	1,49	0,00
Q8TBA6-2	Isoform 2 of Golgin subfamily A member 5 OS=Homo sapiens GN=GOLGA5 - [GOGA5_HUMAN]	1,64	0,00
Q92830	Histone acetyltransferase KAT2A OS=Homo sapiens GN=KAT2A PE=1 SV=3 - [KAT2A_HUMAN]	1,49	0,00
Q96E39	RNA binding motif protein, X-linked-like-1 OS=Homo sapiens GN=RBMXL1 PE=1 SV=1 - [RMXL1_HUMAN]	1,51	0,00
Q14571	Inositol 1,4,5-trisphosphate receptor type 2 OS=Homo sapiens GN=ITPR2 PE=1 SV=2 - [ITPR2_HUMAN]	1,50	0,00
Q9UH99	SUN domain-containing protein 2 OS=Homo sapiens GN=SUN2 PE=1 SV=3 - [SUN2_HUMAN]	1,56	0,00
P21912	Succinate dehydrogenase [ubiquinone] iron-sulfur subunit, mitochondrial OS=Homo sapiens GN=SDHB PE=1 SV=3 -	1,57	0,00
Q16610	Extracellular matrix protein 1 OS=Homo sapiens GN=ECM1 PE=1 SV=2 - [ECM1_HUMAN]	2,26	0,00
Q07954	Prolow-density lipoprotein receptor-related protein 1 OS=Homo sapiens GN=LRP1 PE=1 SV=2 - [LRP1_HUMAN]	2,00	0,00
Q96DB5	Regulator of microtubule dynamics protein 1 OS=Homo sapiens GN=FAM82B PE=1 SV=1 - [RMD1_HUMAN]	1,74	0,00
Q9UBV2	Protein sel-1 homolog 1 OS=Homo sapiens GN=SEL1L PE=1 SV=3 - [SE1L1_HUMAN]	1,54	0,00
Q86Y91-2	Isoform 2 of Kinesin-like protein KIF18B OS=Homo sapiens GN=KIF18B - [KI18B_HUMAN]	2,12	0,00
P80303	Nucleobindin-2 OS=Homo sapiens GN=NUCB2 PE=1 SV=2 - [NUCB2_HUMAN]	1,96	0,00
Q10570	Cleavage and polyadenylation specificity factor subunit 1 OS=Homo sapiens GN=CPSF1 PE=1 SV=2 - [CPSF1_HUMAN	1,47	0,00
P31930	Cytochrome b-c1 complex subunit 1, mitochondrial OS=Homo sapiens GN=UQCRC1 PE=1 SV=3 - [QCR1_HUMAN]	1,47	0,00
Q9ULW0	Targeting protein for Xklp2 OS=Homo sapiens GN=TPX2 PE=1 SV=2 - [TPX2_HUMAN]	1,77	0,00
Q9UBF1	Melanoma-associated antigen C2 OS=Homo sapiens GN=MAGEC2 PE=1 SV=1 - [MAGC2_HUMAN]	1,72	0,00
Q9BQA9	Uncharacterized protein C17orf62 OS=Homo sapiens GN=C17orf62 PE=1 SV=1 - [CQ062_HUMAN]	1,93	0,00
Q9NSE4	Isoleucine--tRNA ligase, mitochondrial OS=Homo sapiens GN=IARS2 PE=1 SV=2 - [SYIM_HUMAN]	1,52	0,00
P36957	Dihydrolipoyllysine-residue succinyltransferase component of 2-oxoglutarate dehydrogenase complex, mitochondr	1,51	0,00
Q9H9B4	Sideroflexin-1 OS=Homo sapiens GN=SFXN1 PE=1 SV=4 - [SFXN1_HUMAN]	1,51	0,00
Q5SXM8	DNL-type zinc finger protein OS=Homo sapiens GN=DNLZ PE=2 SV=1 - [DNLZ_HUMAN]	1,54	0,00
Q86UT6	NLR family member X1 OS=Homo sapiens GN=NLRX1 PE=1 SV=1 - [NLRX1_HUMAN]	1,87	0,00
Q9BUP3-3	Isoform 3 of Oxidoreductase HTATIP2 OS=Homo sapiens GN=HTATIP2 - [HTAI2_HUMAN]	1,65	0,00
Q96E29-2	Isoform 2 of mTERF domain-containing protein 1, mitochondrial OS=Homo sapiens GN=MTERFD1 - [MTER1_HUMA	1,65	0,00
Q8WUY1	UPF0670 protein C8orf55 OS=Homo sapiens GN=C8orf55 PE=1 SV=2 - [CH055_HUMAN]	1,54	0,00
Q9H330-3	Isoform 3 of Transmembrane protein C9orf5 OS=Homo sapiens GN=C9orf5 - [CI005_HUMAN]	1,74	0,00
P54851	Epithelial membrane protein 2 OS=Homo sapiens GN=EMP2 PE=2 SV=1 - [EMP2_HUMAN]	1,84	0,00
Q9BV94-2	Isoform 2 of ER degradation-enhancing alpha-mannosidase-like 2 OS=Homo sapiens GN=EDEM2 - [EDEM2_HUMAN	1,58	0,00
Q9NUT2-2	Isoform Short of ATP-binding cassette sub-family B member 8, mitochondrial OS=Homo sapiens GN=ABCB8 - [ABCB	1,50	0,00
P05106-2	Isoform Beta-3B of Integrin beta-3 OS=Homo sapiens GN=ITGB3 - [ITB3_HUMAN]	2,02	0,00

A375 vs. A375PR1

Q86WA6-2	Isoform 2 of Valacyclovir hydrolase OS=Homo sapiens GN=BPHL - [BPHL_HUMAN]	1,73	0,00
P61916	Epididymal secretory protein E1 OS=Homo sapiens GN=NPC2 PE=1 SV=1 - [NPC2_HUMAN]	2,38	0,00
O75251	NADH dehydrogenase [ubiquinone] iron-sulfur protein 7, mitochondrial OS=Homo sapiens GN=NDUFS7 PE=1 SV=3 -	1,54	0,00
O43766	Lipoyl synthase, mitochondrial OS=Homo sapiens GN=LIAS PE=2 SV=3 - [LIAS_HUMAN]	1,51	0,00
P56199	Integrin alpha-1 OS=Homo sapiens GN=ITGA1 PE=1 SV=2 - [ITA1_HUMAN]	1,66	0,00
Q8WU67	Abhydrolase domain-containing protein 3 OS=Homo sapiens GN=ABHD3 PE=2 SV=2 - [ABHD3_HUMAN]	1,68	0,00
Q9Y277	Voltage-dependent anion-selective channel protein 3 OS=Homo sapiens GN=VDAC3 PE=1 SV=1 - [VDAC3_HUMAN]	1,46	0,00
Q9NVT9	Armadillo repeat-containing protein 1 OS=Homo sapiens GN=ARMC1 PE=1 SV=1 - [ARMC1_HUMAN]	1,68	0,00
O60266	Adenylate cyclase type 3 OS=Homo sapiens GN=ADCY3 PE=1 SV=3 - [ADCY3_HUMAN]	1,67	0,00
Q15582	Transforming growth factor-beta-induced protein ig-h3 OS=Homo sapiens GN=TGFBI PE=1 SV=1 - [BGH3_HUMAN]	1,46	0,15
Q9H9P8-2	Isoform 2 of L-2-hydroxyglutarate dehydrogenase, mitochondrial OS=Homo sapiens GN=L2HGDH - [L2HDH_HUMAN	1,51	0,15
Q9Y666	Solute carrier family 12 member 7 OS=Homo sapiens GN=SLC12A7 PE=1 SV=3 - [S12A7_HUMAN]	1,55	0,15
Q8IW92	Beta-galactosidase-1-like protein 2 OS=Homo sapiens GN=GLB1L2 PE=2 SV=1 - [GLBL2_HUMAN]	1,61	0,15
Q10588	ADP-ribosyl cyclase 2 OS=Homo sapiens GN=BST1 PE=1 SV=2 - [BST1_HUMAN]	1,58	0,15
Q5T160	Probable arginine--tRNA ligase, mitochondrial OS=Homo sapiens GN=RARS2 PE=1 SV=1 - [SYRM_HUMAN]	1,46	0,15
Q8TE73	Dynein heavy chain 5, axonemal OS=Homo sapiens GN=DNAH5 PE=1 SV=3 - [DYH5_HUMAN]	1,37	0,15
O60427	Fatty acid desaturase 1 OS=Homo sapiens GN=FADS1 PE=1 SV=1 - [FADS1_HUMAN]	1,78	0,15
Q9NZJ7-2	Isoform 2 of Mitochondrial carrier homolog 1 OS=Homo sapiens GN=MTCH1 - [MTCH1_HUMAN]	1,52	0,15
Q14980	Nuclear mitotic apparatus protein 1 OS=Homo sapiens GN=NUMA1 PE=1 SV=2 - [NUMA1_HUMAN]	1,69	0,15
Q658P3-4	Isoform 4 of Metalloreductase STEAP3 OS=Homo sapiens GN=STEAP3 - [STEA3_HUMAN]	1,68	0,15
Q9NPG3-2	Isoform 2 of Ubinuclein-1 OS=Homo sapiens GN=UBN1 - [UBN1_HUMAN]	1,57	0,15
P52848	Bifunctional heparan sulfate N-deacetylase/N-sulfotransferase 1 OS=Homo sapiens GN=NDST1 PE=1 SV=1 - [NDST1	1,57	0,15
Q6YN16-2	Isoform 2 of Hydroxysteroid dehydrogenase-like protein 2 OS=Homo sapiens GN=HSDL2 - [HSDL2_HUMAN]	1,63	0,15
Q9BW19	Kinesin-like protein KIFC1 OS=Homo sapiens GN=KIFC1 PE=1 SV=2 - [KIFC1_HUMAN]	1,90	0,15
Q5T3F8	Transmembrane protein 63B OS=Homo sapiens GN=TMEM63B PE=1 SV=1 - [TM63B_HUMAN]	1,47	0,15
Q9BRK5	45 kDa calcium-binding protein OS=Homo sapiens GN=SDF4 PE=1 SV=1 - [CAB45_HUMAN]	1,62	0,15
Q15643	Thyroid receptor-interacting protein 11 OS=Homo sapiens GN=TRIP11 PE=1 SV=3 - [TRIPB_HUMAN]	1,41	0,15
Q9BSH4	Translational activator of cytochrome c oxidase 1 OS=Homo sapiens GN=TACO1 PE=1 SV=1 - [TACO1_HUMAN]	1,46	0,15
O94919	Endonuclease domain-containing 1 protein OS=Homo sapiens GN=ENDOD1 PE=1 SV=2 - [ENDD1_HUMAN]	1,58	0,15
P21926	CD9 antigen OS=Homo sapiens GN=CD9 PE=1 SV=4 - [CD9_HUMAN]	1,95	0,15
Q5J8M3	Transmembrane protein 85 OS=Homo sapiens GN=TMEM85 PE=1 SV=2 - [TMM85_HUMAN]	1,46	0,15
P17096-2	Isoform HMG-Y of High mobility group protein HMG-I/HMG-Y OS=Homo sapiens GN=HMGA1 - [HMGA1_HUMAN]	1,75	0,15
Q15006	Tetratricopeptide repeat protein 35 OS=Homo sapiens GN=TTC35 PE=1 SV=1 - [TTC35_HUMAN]	1,50	0,15

A375 vs. A375PR1

Q9UHQ4	B-cell receptor-associated protein 29 OS=Homo sapiens GN=BCAP29 PE=1 SV=2 - [BAP29_HUMAN]	1,50	0,15
P57105	Synaptojanin-2-binding protein OS=Homo sapiens GN=SYNJ2BP PE=1 SV=2 - [SYJ2B_HUMAN]	1,54	0,15
Q9Y4K3	TNF receptor-associated factor 6 OS=Homo sapiens GN=TRAF6 PE=1 SV=1 - [TRAF6_HUMAN]	1,36	0,15
P30048	Thioredoxin-dependent peroxide reductase, mitochondrial OS=Homo sapiens GN=PRDX3 PE=1 SV=3 - [PRDX3_HUM	1,49	0,15
Q9UBU6	Protein FAM8A1 OS=Homo sapiens GN=FAM8A1 PE=1 SV=1 - [FA8A1_HUMAN]	1,46	0,15
Q9NV64	Transmembrane protein 39A OS=Homo sapiens GN=TMEM39A PE=2 SV=1 - [TM39A_HUMAN]	1,44	0,15
O95772-2	Isoform 2 of MLN64 N-terminal domain homolog OS=Homo sapiens GN=STARD3NL - [MENTO_HUMAN]	1,57	0,15
P49454	Centromere protein F OS=Homo sapiens GN=CENPF PE=1 SV=2 - [CENPF_HUMAN]	1,63	0,15
Q969Q5	Ras-related protein Rab-24 OS=Homo sapiens GN=RAB24 PE=1 SV=1 - [RAB24_HUMAN]	1,43	0,15
O15118	Niemann-Pick C1 protein OS=Homo sapiens GN=NPC1 PE=1 SV=2 - [NPC1_HUMAN]	1,60	0,15
Q14108	Lysosome membrane protein 2 OS=Homo sapiens GN=SCARB2 PE=1 SV=2 - [SCRB2_HUMAN]	1,42	0,15
P51649	Succinate-semialdehyde dehydrogenase, mitochondrial OS=Homo sapiens GN=ALDH5A1 PE=1 SV=2 - [SSDH_HUMA	1,71	0,15
P42892-3	Isoform C of Endothelin-converting enzyme 1 OS=Homo sapiens GN=ECE1 - [ECE1_HUMAN]	1,52	0,15
Q9H936	Mitochondrial glutamate carrier 1 OS=Homo sapiens GN=SLC25A22 PE=1 SV=1 - [GHC1_HUMAN]	1,54	0,15
P08582	Melanotransferrin OS=Homo sapiens GN=MFI2 PE=1 SV=2 - [TRFM_HUMAN]	1,74	0,15
Q6SZW1	Sterile alpha and TIR motif-containing protein 1 OS=Homo sapiens GN=SARM1 PE=1 SV=1 - [SARM1_HUMAN]	1,64	0,15
Q6P1M0	Long-chain fatty acid transport protein 4 OS=Homo sapiens GN=SLC27A4 PE=1 SV=1 - [S27A4_HUMAN]	1,52	0,15
P51970	NADH dehydrogenase [ubiquinone] 1 alpha subcomplex subunit 8 OS=Homo sapiens GN=NDUFA8 PE=1 SV=3 - [NDU	1,53	0,15
O75489	NADH dehydrogenase [ubiquinone] iron-sulfur protein 3, mitochondrial OS=Homo sapiens GN=NDUFS3 PE=1 SV=1	1,45	0,15
P33527-3	Isoform 3 of Multidrug resistance-associated protein 1 OS=Homo sapiens GN=ABCC1 - [MRP1_HUMAN]	1,43	0,15
P00367	Glutamate dehydrogenase 1, mitochondrial OS=Homo sapiens GN=GLUD1 PE=1 SV=2 - [DHE3_HUMAN]	1,50	0,15
O15400-2	Isoform 2 of Syntaxin-7 OS=Homo sapiens GN=STX7 - [STX7_HUMAN]	1,51	0,15
Q8IXI1	Mitochondrial Rho GTPase 2 OS=Homo sapiens GN=RHOT2 PE=1 SV=2 - [MIRO2_HUMAN]	1,59	0,15
Q96GQ5	UPF0420 protein C16orf58 OS=Homo sapiens GN=C16orf58 PE=1 SV=2 - [CP058_HUMAN]	1,52	0,15
Q96IG2-2	Isoform 2 of F-box/LRR-repeat protein 20 OS=Homo sapiens GN=FBXL20 - [FXL20_HUMAN]	1,48	0,15
O15321	Transmembrane 9 superfamily member 1 OS=Homo sapiens GN=TM9SF1 PE=2 SV=2 - [TM9S1_HUMAN]	1,51	0,15
Q8NAP3	Zinc finger and BTB domain-containing protein 38 OS=Homo sapiens GN=ZBTB38 PE=1 SV=2 - [ZBT38_HUMAN]	1,71	0,15
Q15390	Mitochondrial fission regulator 1 OS=Homo sapiens GN=MTFR1 PE=1 SV=2 - [MTFR1_HUMAN]	1,53	0,15
Q99569-2	Isoform 2 of Plakophilin-4 OS=Homo sapiens GN=PKP4 - [PKP4_HUMAN]	1,47	0,15
O14656	Torsin-1A OS=Homo sapiens GN=TOR1A PE=1 SV=1 - [TOR1A_HUMAN]	1,45	0,15
Q9Y2G8	DnaJ homolog subfamily C member 16 OS=Homo sapiens GN=DNAJC16 PE=2 SV=3 - [DJC16_HUMAN]	1,52	0,15
O43676	NADH dehydrogenase [ubiquinone] 1 beta subcomplex subunit 3 OS=Homo sapiens GN=NDUFB3 PE=1 SV=3 - [NDU	1,54	0,15
P51151	Ras-related protein Rab-9A OS=Homo sapiens GN=RAB9A PE=1 SV=1 - [RAB9A_HUMAN]	1,56	0,15

A375 vs. A375PR1

Q96LW7-2	Isoform 2 of Bcl10-interacting CARD protein OS=Homo sapiens GN=C9orf89 - [BINCA_HUMAN]	1,47	0,15
P48449	Lanosterol synthase OS=Homo sapiens GN=LSS PE=1 SV=1 - [ERG7_HUMAN]	1,49	0,15
Q9BWM7	Sideroflexin-3 OS=Homo sapiens GN=SFXN3 PE=2 SV=2 - [SFXN3_HUMAN]	1,55	0,15
Q02318	Sterol 26-hydroxylase, mitochondrial OS=Homo sapiens GN=CYP27A1 PE=1 SV=1 - [CP27A_HUMAN]	1,50	0,15
Q9NUL7	Probable ATP-dependent RNA helicase DDX28 OS=Homo sapiens GN=DDX28 PE=2 SV=2 - [DDX28_HUMAN]	1,48	0,15
Q08722-2	Isoform OA3-293 of Leukocyte surface antigen CD47 OS=Homo sapiens GN=CD47 - [CD47_HUMAN]	1,50	0,15
Q9P0J1	[Pyruvate dehydrogenase [acetyl-transferring]]-phosphatase 1, mitochondrial OS=Homo sapiens GN=PDP1 PE=1 SV	1,46	0,15
P08910	Abhydrolase domain-containing protein 2 OS=Homo sapiens GN=ABHD2 PE=2 SV=1 - [ABHD2_HUMAN]	1,43	0,15
Q02818	Nucleobindin-1 OS=Homo sapiens GN=NUCB1 PE=1 SV=4 - [NUCB1_HUMAN]	1,47	0,15
Q15629	Translocating chain-associated membrane protein 1 OS=Homo sapiens GN=TRAM1 PE=1 SV=3 - [TRAM1_HUMAN]	1,54	0,15
P45954	Short/branched chain specific acyl-CoA dehydrogenase, mitochondrial OS=Homo sapiens GN=ACADSB PE=1 SV=1 -	1,47	0,15
Q96QE5	Transcription elongation factor, mitochondrial OS=Homo sapiens GN=TEFM PE=1 SV=1 - [TEFM_HUMAN]	1,41	0,15
P08559	Pyruvate dehydrogenase E1 component subunit alpha, somatic form, mitochondrial OS=Homo sapiens GN=PDHA1	1,45	0,15
O43464-2	Isoform 2 of Serine protease HTRA2, mitochondrial OS=Homo sapiens GN=HTRA2 - [HTRA2_HUMAN]	1,59	0,15
P05496	ATP synthase lipid-binding protein, mitochondrial OS=Homo sapiens GN=ATP5G1 PE=2 SV=2 - [AT5G1_HUMAN]	1,49	0,15
Q86Y39	NADH dehydrogenase [ubiquinone] 1 alpha subcomplex subunit 11 OS=Homo sapiens GN=NDUFA11 PE=1 SV=3 - [N	1,96	0,15
Q8WVM0	Dimethyladenosine transferase 1, mitochondrial OS=Homo sapiens GN=TFB1M PE=1 SV=1 - [TFB1M_HUMAN]	1,55	0,15
Q92878	DNA repair protein RAD50 OS=Homo sapiens GN=RAD50 PE=1 SV=1 - [RAD50_HUMAN]	1,49	0,15
Q12846	Syntaxin-4 OS=Homo sapiens GN=STX4 PE=1 SV=2 - [STX4_HUMAN]	1,44	0,15
Q9NUQ7	Ufm1-specific protease 2 OS=Homo sapiens GN=UFSP2 PE=2 SV=3 - [UFSP2_HUMAN]	1,52	0,15
Q8NBZ7	UDP-glucuronic acid decarboxylase 1 OS=Homo sapiens GN=UXS1 PE=1 SV=1 - [UXS1_HUMAN]	1,57	0,15
Q9NX40	OCIA domain-containing protein 1 OS=Homo sapiens GN=OCIAD1 PE=1 SV=1 - [OCAD1_HUMAN]	1,47	0,15
P18433-2	Isoform 2 of Receptor-type tyrosine-protein phosphatase alpha OS=Homo sapiens GN=PTPRA - [PTPRA_HUMAN]	1,56	0,22
O00478	Butyrophilin subfamily 3 member A3 OS=Homo sapiens GN=BTN3A3 PE=1 SV=1 - [BT3A3_HUMAN]	1,44	0,22
Q8NFJ5	Retinoic acid-induced protein 3 OS=Homo sapiens GN=GPRC5A PE=1 SV=2 - [RAI3_HUMAN]	1,77	0,22
Q9BU61	NADH dehydrogenase [ubiquinone] 1 alpha subcomplex assembly factor 3 OS=Homo sapiens GN=NDUFAF3 PE=1 SV	1,49	0,22
O15119-3	Isoform III of T-box transcription factor TBX3 OS=Homo sapiens GN=TBX3 - [TBX3_HUMAN]	1,54	0,22
Q8TE99-2	Isoform 2 of Acid phosphatase-like protein 2 OS=Homo sapiens GN=ACPL2 - [ACPL2_HUMAN]	1,55	0,22
Q16718	NADH dehydrogenase [ubiquinone] 1 alpha subcomplex subunit 5 OS=Homo sapiens GN=NDUFA5 PE=1 SV=3 - [NDI	1,47	0,22
Q8N138-4	Isoform 2 of ORM1-like protein 3 OS=Homo sapiens GN=ORMDL3 - [ORML3_HUMAN]	1,45	0,22
P17152	Transmembrane protein 11, mitochondrial OS=Homo sapiens GN=TMEM11 PE=1 SV=1 - [TMM11_HUMAN]	1,43	0,22
P05166	Propionyl-CoA carboxylase beta chain, mitochondrial OS=Homo sapiens GN=PCCB PE=1 SV=3 - [PCCB_HUMAN]	1,44	0,22
Q99627	COP9 signalosome complex subunit 8 OS=Homo sapiens GN=COPS8 PE=1 SV=1 - [CSN8_HUMAN]	1,42	0,22

A375 vs. A375PR1

P11177-2	Isoform 2 of Pyruvate dehydrogenase E1 component subunit beta, mitochondrial OS=Homo sapiens GN=PDHB - [OI	1,45	0,22
Q5ST30	Valine--tRNA ligase, mitochondrial OS=Homo sapiens GN=VARS2 PE=1 SV=2 - [SYVM_HUMAN]	1,42	0,22
P11169	Solute carrier family 2, facilitated glucose transporter member 3 OS=Homo sapiens GN=SLC2A3 PE=1 SV=1 - [GTR3_	1,70	0,22
Q5UCC4-2	Isoform 2 of UPF0510 protein INM02 OS=Homo sapiens GN=C19orf63 - [INM02_HUMAN]	1,46	0,22
Q13011	Delta(3,5)-Delta(2,4)-dienoyl-CoA isomerase, mitochondrial OS=Homo sapiens GN=ECH1 PE=1 SV=2 - [ECH1_HUMA	1,47	0,22
Q9HBM0	Vezatin OS=Homo sapiens GN=VEZT PE=1 SV=3 - [VEZA_HUMAN]	1,55	0,22
Q9H6X2-3	Isoform 3 of Anthrax toxin receptor 1 OS=Homo sapiens GN=ANTXR1 - [ANTR1_HUMAN]	1,54	0,22
Q9UGL1	Lysine-specific demethylase 5B OS=Homo sapiens GN=KDM5B PE=1 SV=3 - [KDM5B_HUMAN]	1,63	0,22
Q709F0-3	Isoform 3 of Acyl-CoA dehydrogenase family member 11 OS=Homo sapiens GN=ACAD11 - [ACD11_HUMAN]	1,40	0,22
Q02218	2-oxoglutarate dehydrogenase, mitochondrial OS=Homo sapiens GN=OGDH PE=1 SV=3 - [ODO1_HUMAN]	1,56	0,22
Q9H7Z7	Prostaglandin E synthase 2 OS=Homo sapiens GN=PTGES2 PE=1 SV=1 - [PGES2_HUMAN]	1,50	0,22
Q99805	Transmembrane 9 superfamily member 2 OS=Homo sapiens GN=TM9SF2 PE=1 SV=1 - [TM9S2_HUMAN]	1,45	0,22
O94788-2	Isoform 2 of Retinal dehydrogenase 2 OS=Homo sapiens GN=ALDH1A2 - [AL1A2_HUMAN]	1,46	0,22
Q8TC12-2	Isoform 2 of Retinol dehydrogenase 11 OS=Homo sapiens GN=RDH11 - [RDH11_HUMAN]	1,65	0,22
P62341	Selenoprotein T OS=Homo sapiens GN=SELT PE=2 SV=2 - [SELT_HUMAN]	1,57	0,22
Q8IW50-6	Isoform 6 of Uncharacterized protein C9orf25 OS=Homo sapiens GN=C9orf25 - [CI025_HUMAN]	1,66	0,22
O00400	Acetyl-coenzyme A transporter 1 OS=Homo sapiens GN=SLC33A1 PE=1 SV=1 - [ACATN_HUMAN]	1,56	0,22
O43493-2	Isoform TGN46 of Trans-Golgi network integral membrane protein 2 OS=Homo sapiens GN=TGOLN2 - [TGON2_HUI	1,48	0,22
P36542-2	Isoform Heart of ATP synthase subunit gamma, mitochondrial OS=Homo sapiens GN=ATP5C1 - [ATPG_HUMAN]	1,57	0,22
Q13439-3	Isoform 3 of Golgin subfamily A member 4 OS=Homo sapiens GN=GOLGA4 - [GOGA4_HUMAN]	1,44	0,22
Q5JTV8	Torsin-1A-interacting protein 1 OS=Homo sapiens GN=TOR1AIP1 PE=1 SV=2 - [TOIP1_HUMAN]	1,45	0,22
Q96D31	Calcium release-activated calcium channel protein 1 OS=Homo sapiens GN=ORAI1 PE=1 SV=2 - [CRCM1_HUMAN]	1,37	0,22
O75306	NADH dehydrogenase [ubiquinone] iron-sulfur protein 2, mitochondrial OS=Homo sapiens GN=NDUFS2 PE=1 SV=2 -	1,42	0,22
Q01850	Cerebellar degeneration-related protein 2 OS=Homo sapiens GN=CDR2 PE=1 SV=2 - [CDR2_HUMAN]	1,36	0,22
P13056	Nuclear receptor subfamily 2 group C member 1 OS=Homo sapiens GN=NR2C1 PE=1 SV=2 - [NR2C1_HUMAN]	1,34	0,22
Q86UD1	Out at first protein homolog OS=Homo sapiens GN=OAF PE=2 SV=1 - [OAF_HUMAN]	1,50	0,22
O43678	NADH dehydrogenase [ubiquinone] 1 alpha subcomplex subunit 2 OS=Homo sapiens GN=NDUFA2 PE=1 SV=3 - [NDI	1,48	0,22
Q9BV23	Monoacylglycerol lipase ABHD6 OS=Homo sapiens GN=ABHD6 PE=2 SV=1 - [ABHD6_HUMAN]	1,70	0,22
P30536	Translocator protein OS=Homo sapiens GN=TSPO PE=1 SV=3 - [TSPOA_HUMAN]	1,54	0,22
Q7L8L6	FAST kinase domain-containing protein 5 OS=Homo sapiens GN=FASTKD5 PE=1 SV=1 - [FAKD5_HUMAN]	1,50	0,22
Q9UN79	Transcription factor SOX-13 OS=Homo sapiens GN=SOX13 PE=1 SV=3 - [SOX13_HUMAN]	1,53	0,22
Q9NP92	28S ribosomal protein S30, mitochondrial OS=Homo sapiens GN=MRPS30 PE=1 SV=2 - [RT30_HUMAN]	1,53	0,22
Q9GZT6-2	Isoform 2 of Coiled-coil domain-containing protein 90B, mitochondrial OS=Homo sapiens GN=CCDC90B - [CC90B_H	1,58	0,22

O95140	Mitofusin-2 OS=Homo sapiens GN=MFN2 PE=1 SV=3 - [MFN2_HUMAN]	1,39	0,22
Q9P2I0	Cleavage and polyadenylation specificity factor subunit 2 OS=Homo sapiens GN=CPSF2 PE=1 SV=2 - [CPSF2_HUMAN	1,39	0,22
Q86UP2	Kinectin OS=Homo sapiens GN=KTN1 PE=1 SV=1 - [KTN1_HUMAN]	1,43	0,22
Q04724	Transducin-like enhancer protein 1 OS=Homo sapiens GN=TLE1 PE=1 SV=2 - [TLE1_HUMAN]	1,60	0,22
Q9NX62	Inositol monophosphatase 3 OS=Homo sapiens GN=IMPAD1 PE=1 SV=1 - [IMPA3_HUMAN]	1,76	0,22
Q8WVX9	Fatty acyl-CoA reductase 1 OS=Homo sapiens GN=FAR1 PE=1 SV=1 - [FACR1_HUMAN]	1,44	0,22
Q9NNW7-2	Isoform 2 of Thioredoxin reductase 2, mitochondrial OS=Homo sapiens GN=TXNRD2 - [TRXR2_HUMAN]	1,49	0,22
O95169	NADH dehydrogenase [ubiquinone] 1 beta subcomplex subunit 8, mitochondrial OS=Homo sapiens GN=NDUFB8 PE	1,44	0,22
Q8TEK3-2	Isoform 1 of Histone-lysine N-methyltransferase, H3 lysine-79 specific OS=Homo sapiens GN=DOT1L - [DOT1L_HUM	1,43	0,22
P30837	Aldehyde dehydrogenase X, mitochondrial OS=Homo sapiens GN=ALDH1B1 PE=1 SV=3 - [AL1B1_HUMAN]	1,46	0,22
Q7Z7H5-3	Isoform 3 of Transmembrane emp24 domain-containing protein 4 OS=Homo sapiens GN=TMED4 - [TMED4_HUMA	1,47	0,22
P00387-2	Isoform 2 of NADH-cytochrome b5 reductase 3 OS=Homo sapiens GN=CYB5R3 - [NB5R3_HUMAN]	1,44	0,22
Q96I36	Uncharacterized protein C12orf62 OS=Homo sapiens GN=C12orf62 PE=2 SV=1 - [CL062_HUMAN]	1,51	0,22
Q9UJJ9	N-acetylglucosamine-1-phosphotransferase subunit gamma OS=Homo sapiens GN=GNPTG PE=1 SV=1 - [GNPTG_HU	1,61	0,22
Q4G176	Acyl-CoA synthetase family member 3, mitochondrial OS=Homo sapiens GN=ACSF3 PE=1 SV=3 - [ACSF3_HUMAN]	1,44	0,22
Q93063	Exostosin-2 OS=Homo sapiens GN=EXT2 PE=1 SV=1 - [EXT2_HUMAN]	1,39	0,22
O14686	Histone-lysine N-methyltransferase MLL2 OS=Homo sapiens GN=MLL2 PE=1 SV=2 - [MLL2_HUMAN]	1,77	0,22
O94923	D-glucuronyl C5-epimerase OS=Homo sapiens GN=GLCE PE=1 SV=3 - [GLCE_HUMAN]	1,49	0,22
Q15746-4	Isoform 3B of Myosin light chain kinase, smooth muscle OS=Homo sapiens GN=MYLK - [MYLK_HUMAN]	1,40	0,22
O95674	Phosphatidate cytidylyltransferase 2 OS=Homo sapiens GN=CDS2 PE=1 SV=1 - [CDS2_HUMAN]	1,65	0,22
Q96HE9	Proline-rich protein 11 OS=Homo sapiens GN=PRR11 PE=1 SV=1 - [PRR11_HUMAN]	1,51	0,22
P43307	Translocon-associated protein subunit alpha OS=Homo sapiens GN=SSR1 PE=1 SV=3 - [SSRA_HUMAN]	1,44	0,22
P49069	Calcium signal-modulating cyclophilin ligand OS=Homo sapiens GN=CAMLG PE=1 SV=1 - [CAMLG_HUMAN]	1,48	0,22
Q9NUQ2	1-acyl-sn-glycerol-3-phosphate acyltransferase epsilon OS=Homo sapiens GN=AGPAT5 PE=1 SV=3 - [PLCE_HUMAN]	1,41	0,22
P24539	ATP synthase subunit b, mitochondrial OS=Homo sapiens GN=ATP5F1 PE=1 SV=2 - [AT5F1_HUMAN]	1,42	0,22
Q8TBF5	Phosphatidylinositol-glycan biosynthesis class X protein OS=Homo sapiens GN=PIGX PE=2 SV=3 - [PIGX_HUMAN]	1,45	0,22
Q8NBJ4-2	Isoform 2 of Golgi membrane protein 1 OS=Homo sapiens GN=GOLM1 - [GOLM1_HUMAN]	1,35	0,22
Q9P2E9	Ribosome-binding protein 1 OS=Homo sapiens GN=RRBP1 PE=1 SV=4 - [RRBP1_HUMAN]	1,43	0,22
Q9HCU5	Prolactin regulatory element-binding protein OS=Homo sapiens GN=PREB PE=1 SV=2 - [PREB_HUMAN]	1,39	0,22
Q96DZ1	Endoplasmic reticulum lectin 1 OS=Homo sapiens GN=ERLEC1 PE=1 SV=1 - [ERLEC_HUMAN]	1,58	0,22
Q9BTC8-2	Isoform 2 of Metastasis-associated protein MTA3 OS=Homo sapiens GN=MTA3 - [MTA3_HUMAN]	1,42	0,22
P50402	Emerin OS=Homo sapiens GN=EMD PE=1 SV=1 - [EMD_HUMAN]	1,41	0,22
O95639-3	Isoform 3 of Cleavage and polyadenylation specificity factor subunit 4 OS=Homo sapiens GN=CPSF4 - [CPSF4_HUM/	1,46	0,22

Q9BT17	Mitochondrial GTPase 1 OS=Homo sapiens GN=MTG1 PE=1 SV=2 - [MTG1_HUMAN]	1,38	0,22
O15126-2	Isoform 2 of Secretory carrier-associated membrane protein 1 OS=Homo sapiens GN=SCAMP1 - [SCAM1_HUMAN]	1,45	0,22
Q9BT40-2	Isoform 2 of Inositol polyphosphate 5-phosphatase K OS=Homo sapiens GN=INPP5K - [INP5K_HUMAN]	1,46	0,22
Q9UMS0-2	Isoform 2 of NFU1 iron-sulfur cluster scaffold homolog, mitochondrial OS=Homo sapiens GN=NFU1 - [NFU1_HUMA	1,45	0,22
O14524	Transmembrane protein 194A OS=Homo sapiens GN=TMEM194A PE=1 SV=2 - [T194A_HUMAN]	1,43	0,22
Q9Y4P3	Transducin beta-like protein 2 OS=Homo sapiens GN=TBL2 PE=1 SV=1 - [TBL2_HUMAN]	1,41	0,22
Q00325-2	Isoform B of Phosphate carrier protein, mitochondrial OS=Homo sapiens GN=SLC25A3 - [MPCP_HUMAN]	1,43	0,22
Q6DD88	Atlastin-3 OS=Homo sapiens GN=ATL3 PE=1 SV=1 - [ATLA3_HUMAN]	1,58	0,22
P04626-4	Isoform 4 of Receptor tyrosine-protein kinase erbB-2 OS=Homo sapiens GN=ERBB2 - [ERBB2_HUMAN]	1,39	0,22
P04920-2	Isoform B1 of Anion exchange protein 2 OS=Homo sapiens GN=SLC4A2 - [B3A2_HUMAN]	1,51	0,22
P45880-2	Isoform 2 of Voltage-dependent anion-selective channel protein 2 OS=Homo sapiens GN=VDAC2 - [VDAC2_HUMAN	1,41	0,22
Q9UQ90	Paraplegin OS=Homo sapiens GN=SPG7 PE=1 SV=2 - [SPG7_HUMAN]	1,48	0,22
O75052	Carboxyl-terminal PDZ ligand of neuronal nitric oxide synthase protein OS=Homo sapiens GN=NOS1AP PE=1 SV=3 -	1,65	0,22
Q1L5Z9	LON peptidase N-terminal domain and RING finger protein 2 OS=Homo sapiens GN=LONRF2 PE=2 SV=3 - [LONF2_H	1,47	0,22
P24752	Acetyl-CoA acetyltransferase, mitochondrial OS=Homo sapiens GN=ACAT1 PE=1 SV=1 - [THIL_HUMAN]	1,50	0,22
O14662-2	Isoform A of Syntaxin-16 OS=Homo sapiens GN=STX16 - [STX16_HUMAN]	1,42	0,22
Q15771	Ras-related protein Rab-30 OS=Homo sapiens GN=RAB30 PE=1 SV=2 - [RAB30_HUMAN]	1,42	0,22
Q53HL2	Borealin OS=Homo sapiens GN=CDCA8 PE=1 SV=2 - [BOREA_HUMAN]	1,57	0,22
P42167	Lamina-associated polypeptide 2, isoforms beta/gamma OS=Homo sapiens GN=TMPO PE=1 SV=2 - [LAP2B_HUMAN	1,46	0,22
O75746	Calcium-binding mitochondrial carrier protein Aralar1 OS=Homo sapiens GN=SLC25A12 PE=1 SV=2 - [CMC1_HUMA	1,49	0,22
Q96L58	Beta-1,3-galactosyltransferase 6 OS=Homo sapiens GN=B3GALT6 PE=2 SV=2 - [B3GT6_HUMAN]	1,43	0,22
Q9BVS5	Potential tRNA (adenine(58)-N(1))-methyltransferase catalytic subunit TRMT61B OS=Homo sapiens GN=TRMT61B P	1,41	0,22
Q8WUM9	Sodium-dependent phosphate transporter 1 OS=Homo sapiens GN=SLC20A1 PE=1 SV=1 - [S20A1_HUMAN]	1,55	0,22
Q9H845	Acyl-CoA dehydrogenase family member 9, mitochondrial OS=Homo sapiens GN=ACAD9 PE=1 SV=1 - [ACAD9_HUM	1,41	0,22
Q587I9	Vesicle transport protein SFT2C OS=Homo sapiens GN=SFT2D3 PE=2 SV=1 - [SFT2C_HUMAN]	1,62	0,22
O94826	Mitochondrial import receptor subunit TOM70 OS=Homo sapiens GN=TOMM70A PE=1 SV=1 - [TOM70_HUMAN]	1,43	0,22
P51636-3	Isoform C of Caveolin-2 OS=Homo sapiens GN=CAV2 - [CAV2_HUMAN]	1,58	0,22
Q53EU6	Glycerol-3-phosphate acyltransferase 3 OS=Homo sapiens GN=AGPAT9 PE=1 SV=2 - [GPAT3_HUMAN]	1,46	0,22
Q96JB6	Lysyl oxidase homolog 4 OS=Homo sapiens GN=LOXL4 PE=1 SV=1 - [LOXL4_HUMAN]	1,95	0,22
P47985	Cytochrome b-c1 complex subunit Rieske, mitochondrial OS=Homo sapiens GN=UQCRFS1 PE=1 SV=2 - [UCRI_HUMA	1,49	0,22
Q16775	Hydroxyacylglutathione hydrolase, mitochondrial OS=Homo sapiens GN=HAGH PE=1 SV=2 - [GLO2_HUMAN]	1,51	0,22
Q96AE7	Tetratricopeptide repeat protein 17 OS=Homo sapiens GN=TTC17 PE=1 SV=1 - [TTC17_HUMAN]	1,37	0,22
P82932	28S ribosomal protein S6, mitochondrial OS=Homo sapiens GN=MRPS6 PE=1 SV=3 - [RT06_HUMAN]	1,45	0,22

A375 vs. A375PR1

Q13214-2	Isoform 2 of Semaphorin-3B OS=Homo sapiens GN=SEMA3B - [SEM3B_HUMAN]	1,70	0,22
P56385	ATP synthase subunit e, mitochondrial OS=Homo sapiens GN=ATP5I PE=1 SV=2 - [ATP5I_HUMAN]	1,40	0,22
O95298	NADH dehydrogenase [ubiquinone] 1 subunit C2 OS=Homo sapiens GN=NDUFC2 PE=1 SV=1 - [NDUC2_HUMAN]	1,45	0,22
Q9BVT8	Transmembrane and ubiquitin-like domain-containing protein 1 OS=Homo sapiens GN=TMUB1 PE=1 SV=1 - [TMUB	1,44	0,22
Q9Y2R0	Coiled-coil domain-containing protein 56 OS=Homo sapiens GN=CCDC56 PE=1 SV=1 - [CCD56_HUMAN]	1,48	0,22
P51572	B-cell receptor-associated protein 31 OS=Homo sapiens GN=BCAP31 PE=1 SV=3 - [BAP31_HUMAN]	1,49	0,22
A8MXV4	Nucleoside diphosphate-linked moiety X motif 19, mitochondrial OS=Homo sapiens GN=NUDT19 PE=1 SV=1 - [NUD	1,60	0,22
Q96MT1	RING finger protein 145 OS=Homo sapiens GN=RNF145 PE=2 SV=2 - [RN145_HUMAN]	1,46	0,22
Q6PI78	Transmembrane protein 65 OS=Homo sapiens GN=TMEM65 PE=1 SV=2 - [TMM65_HUMAN]	1,42	0,22
Q9HAT2-2	Isoform 2 of Sialate O-acetylesterase OS=Homo sapiens GN=SIAE - [SIAE_HUMAN]	1,73	0,22
Q08431-3	Isoform 3 of Lactadherin OS=Homo sapiens GN=MFGE8 - [MFGM_HUMAN]	1,53	0,22
O43819	Protein SCO2 homolog, mitochondrial OS=Homo sapiens GN=SCO2 PE=1 SV=3 - [SCO2_HUMAN]	1,46	0,22
P17568	NADH dehydrogenase [ubiquinone] 1 beta subcomplex subunit 7 OS=Homo sapiens GN=NDUFB7 PE=1 SV=4 - [NDU	1,39	0,22
Q9NX18	Succinate dehydrogenase assembly factor 2, mitochondrial OS=Homo sapiens GN=SDHAF2 PE=1 SV=1 - [SDHF2_HU	1,43	0,22
P56817-4	Isoform D of Beta-secretase 1 OS=Homo sapiens GN=BACE1 - [BACE1_HUMAN]	1,36	0,22
Q96RK0	Protein capicua homolog OS=Homo sapiens GN=CIC PE=1 SV=2 - [CIC_HUMAN]	1,41	0,22
Q6PCB7	Long-chain fatty acid transport protein 1 OS=Homo sapiens GN=SLC27A1 PE=2 SV=1 - [S27A1_HUMAN]	1,45	0,22
P22695	Cytochrome b-c1 complex subunit 2, mitochondrial OS=Homo sapiens GN=UQCRC2 PE=1 SV=3 - [QCR2_HUMAN]	1,40	0,22
O60907-2	Isoform 2 of F-box-like/WD repeat-containing protein TBL1X OS=Homo sapiens GN=TBL1X - [TBL1X_HUMAN]	1,43	0,22
O00217	NADH dehydrogenase [ubiquinone] iron-sulfur protein 8, mitochondrial OS=Homo sapiens GN=NDUFS8 PE=1 SV=1	1,42	0,22
P56181-2	Isoform 2 of NADH dehydrogenase [ubiquinone] flavoprotein 3, mitochondrial OS=Homo sapiens GN=NDUFV3 - [NI	1,49	0,22
Q9BSJ8	Extended synaptotagmin-1 OS=Homo sapiens GN=ESYT1 PE=1 SV=1 - [ESYT1_HUMAN]	1,47	0,22
O60220	Mitochondrial import inner membrane translocase subunit Tim8 A OS=Homo sapiens GN=TIMM8A PE=1 SV=1 - [TII	1,39	0,22
Q9HAV7	GrpE protein homolog 1, mitochondrial OS=Homo sapiens GN=GRPEL1 PE=1 SV=2 - [GRPE1_HUMAN]	1,46	0,22
P25705	ATP synthase subunit alpha, mitochondrial OS=Homo sapiens GN=ATP5A1 PE=1 SV=1 - [ATPA_HUMAN]	1,38	0,22
O15155	BET1 homolog OS=Homo sapiens GN=BET1 PE=1 SV=1 - [BET1_HUMAN]	1,40	0,22
Q96S59	Ran-binding protein 9 OS=Homo sapiens GN=RANBP9 PE=1 SV=1 - [RANB9_HUMAN]	1,43	0,22
Q5THJ4-2	Isoform 2 of Vacuolar protein sorting-associated protein 13D OS=Homo sapiens GN=VPS13D - [VP13D_HUMAN]	1,39	0,22
Q13232	Nucleoside diphosphate kinase 3 OS=Homo sapiens GN=NME3 PE=1 SV=2 - [NDK3_HUMAN]	1,56	0,22
Q9H0L4	Cleavage stimulation factor subunit 2 tau variant OS=Homo sapiens GN=CSTF2T PE=1 SV=1 - [CSTFT_HUMAN]	1,29	0,22
P15151-3	Isoform Gamma of Poliovirus receptor OS=Homo sapiens GN=PVR - [PVR_HUMAN]	1,53	0,22
Q13641	Trophoblast glycoprotein OS=Homo sapiens GN=TPBG PE=1 SV=1 - [TPBG_HUMAN]	1,43	0,22
Q9BQD7	Protein FAM173A OS=Homo sapiens GN=FAM173A PE=2 SV=1 - [F173A_HUMAN]	1,41	0,22

A375 vs. A375PR1

Q9UDW1	Cytochrome b-c1 complex subunit 9 OS=Homo sapiens GN=UQCR10 PE=1 SV=3 - [QCR9_HUMAN]	1,45	0,22
Q8WWX9	Selenoprotein M OS=Homo sapiens GN=SELM PE=1 SV=3 - [SELM_HUMAN]	1,50	0,22
P61018	Ras-related protein Rab-4B OS=Homo sapiens GN=RAB4B PE=1 SV=1 - [RAB4B_HUMAN]	1,53	0,22
O43674	NADH dehydrogenase [ubiquinone] 1 beta subcomplex subunit 5, mitochondrial OS=Homo sapiens GN=NDUFB5 PE	1,46	0,29
O94901-3	Isoform 3 of SUN domain-containing protein 1 OS=Homo sapiens GN=SUN1 - [SUN1_HUMAN]	1,62	0,29
P27824	Calnexin OS=Homo sapiens GN=CANX PE=1 SV=2 - [CALX_HUMAN]	1,45	0,29
Q69YH5	Cell division cycle-associated protein 2 OS=Homo sapiens GN=CDCA2 PE=1 SV=2 - [CDCA2_HUMAN]	1,46	0,29
Q96A26	Protein FAM162A OS=Homo sapiens GN=FAM162A PE=1 SV=2 - [F162A_HUMAN]	1,73	0,29
Q9C004	Protein sprouty homolog 4 OS=Homo sapiens GN=SPRY4 PE=1 SV=2 - [SPY4_HUMAN]	1,61	0,29
P11310	Medium-chain specific acyl-CoA dehydrogenase, mitochondrial OS=Homo sapiens GN=ACADM PE=1 SV=1 - [ACADM	1,48	0,29
O00592-2	Isoform 2 of Podocalyxin OS=Homo sapiens GN=PODXL - [PODXL_HUMAN]	1,50	0,29
Q9H5Y7	SLIT and NTRK-like protein 6 OS=Homo sapiens GN=SLITRK6 PE=1 SV=3 - [SLIK6_HUMAN]	1,50	0,29
Q9NX47	E3 ubiquitin-protein ligase MARCH5 OS=Homo sapiens GN=MARCH5 PE=1 SV=1 - [MARH5_HUMAN]	1,36	0,29
Q86UL3	Glycerol-3-phosphate acyltransferase 4 OS=Homo sapiens GN=AGPAT6 PE=1 SV=1 - [GPAT4_HUMAN]	1,50	0,29
Q9NVH0	Exonuclease 3'-5' domain-containing protein 2 OS=Homo sapiens GN=EXD2 PE=1 SV=1 - [EXD2_HUMAN]	1,40	0,29
Q8N2F6-3	Isoform 3 of Armadillo repeat-containing protein 10 OS=Homo sapiens GN=ARMC10 - [ARM10_HUMAN]	1,42	0,29
Q9BRB3-3	Isoform 3 of Phosphatidylinositol N-acetylglucosaminyltransferase subunit Q OS=Homo sapiens GN=PIGQ - [PIGQ_H	1,44	0,29
P55055	Oxysterols receptor LXR-beta OS=Homo sapiens GN=NR1H2 PE=1 SV=2 - [NR1H2_HUMAN]	1,34	0,29
O15392-5	Isoform 5 of Baculoviral IAP repeat-containing protein 5 OS=Homo sapiens GN=BIRC5 - [BIRC5_HUMAN]	1,68	0,29
Q9Y2Z9	Ubiquinone biosynthesis monooxygenase COQ6 OS=Homo sapiens GN=COQ6 PE=1 SV=2 - [COQ6_HUMAN]	1,40	0,29
Q8WVI0	UPF0640 protein C3orf78 OS=Homo sapiens GN=C3orf78 PE=2 SV=2 - [CC078_HUMAN]	1,45	0,29
O95197-2	Isoform 2 of Reticulon-3 OS=Homo sapiens GN=RTN3 - [RTN3_HUMAN]	1,46	0,29
P35908	Keratin, type II cytoskeletal 2 epidermal OS=Homo sapiens GN=KRT2 PE=1 SV=2 - [K22E_HUMAN]	1,65	0,29
Q5JRX3	Presequence protease, mitochondrial OS=Homo sapiens GN=PITRM1 PE=1 SV=2 - [PREP_HUMAN]	1,38	0,29
Q9UIW2	Plexin-A1 OS=Homo sapiens GN=PLXNA1 PE=1 SV=3 - [PLXA1_HUMAN]	1,35	0,29
Q6UVK1	Chondroitin sulfate proteoglycan 4 OS=Homo sapiens GN=CSPG4 PE=1 SV=2 - [CSPG4_HUMAN]	1,49	0,29
Q9HDC9	Adipocyte plasma membrane-associated protein OS=Homo sapiens GN=APMAP PE=1 SV=2 - [APMAP_HUMAN]	1,43	0,29
Q9BS91	Probable UDP-sugar transporter protein SLC35A5 OS=Homo sapiens GN=SLC35A5 PE=1 SV=2 - [S35A5_HUMAN]	1,38	0,29
Q96BY9	Transmembrane protein 66 OS=Homo sapiens GN=TMEM66 PE=1 SV=1 - [TMM66_HUMAN]	1,48	0,29
P49448	Glutamate dehydrogenase 2, mitochondrial OS=Homo sapiens GN=GLUD2 PE=1 SV=2 - [DHE4_HUMAN]	1,41	0,29
Q96HY7	Probable 2-oxoglutarate dehydrogenase E1 component DHKTD1, mitochondrial OS=Homo sapiens GN=DHTKD1 PE=	1,44	0,29
Q13724	Mannosyl-oligosaccharide glucosidase OS=Homo sapiens GN=MOGS PE=1 SV=5 - [MOGS_HUMAN]	1,39	0,29
Q9UI09	NADH dehydrogenase [ubiquinone] 1 alpha subcomplex subunit 12 OS=Homo sapiens GN=NDUFA12 PE=1 SV=1 - [N	1,46	0,29

Q96S66	Chloride channel CLIC-like protein 1 OS=Homo sapiens GN=CLCC1 PE=1 SV=1 - [CLCC1_HUMAN]	1,34	0,29
Q5SWX8	Protein odr-4 homolog OS=Homo sapiens GN=ODR4 PE=2 SV=1 - [ODR4_HUMAN]	1,58	0,29
P30049	ATP synthase subunit delta, mitochondrial OS=Homo sapiens GN=ATP5D PE=1 SV=2 - [ATPD_HUMAN]	1,38	0,29
O15173	Membrane-associated progesterone receptor component 2 OS=Homo sapiens GN=PGRMC2 PE=1 SV=1 - [PGRC2_H	1,40	0,29
Q96CG8	Collagen triple helix repeat-containing protein 1 OS=Homo sapiens GN=CTHRC1 PE=1 SV=1 - [CTHR1_HUMAN]	1,76	0,29
Q9H7D7-2	Isoform 2 of WD repeat-containing protein 26 OS=Homo sapiens GN=WDR26 - [WDR26_HUMAN]	1,29	0,29
Q86SR1-3	Isoform 3 of Polypeptide N-acetylgalactosaminyltransferase 10 OS=Homo sapiens GN=GALNT10 - [GLT10_HUMAN]	1,35	0,29
Q86Y82	Syntaxin-12 OS=Homo sapiens GN=STX12 PE=1 SV=1 - [STX12_HUMAN]	1,29	0,29
O00461	Golgi integral membrane protein 4 OS=Homo sapiens GN=GOLIM4 PE=1 SV=1 - [GOLI4_HUMAN]	1,41	0,29
Q9BS26	Endoplasmic reticulum resident protein 44 OS=Homo sapiens GN=ERP44 PE=1 SV=1 - [ERP44_HUMAN]	1,44	0,29
Q5W111	SPRY domain-containing protein 7 OS=Homo sapiens GN=SPRYD7 PE=1 SV=2 - [SPRY7_HUMAN]	1,35	0,29
Q92896	Golgi apparatus protein 1 OS=Homo sapiens GN=GLG1 PE=1 SV=2 - [GSLG1_HUMAN]	1,40	0,29
Q8IY95-2	Isoform 2 of Transmembrane protein 192 OS=Homo sapiens GN=TMEM192 - [TM192_HUMAN]	1,38	0,29
Q8WUK0-2	Isoform 2 of Protein-tyrosine phosphatase mitochondrial 1 OS=Homo sapiens GN=PTPMT1 - [PTPM1_HUMAN]	1,37	0,29
Q9H6V9	UPF0554 protein C2orf43 OS=Homo sapiens GN=C2orf43 PE=1 SV=1 - [CB043_HUMAN]	1,41	0,29
Q6UWP7-2	Isoform 2 of Lysocardiolipin acyltransferase 1 OS=Homo sapiens GN=LCLAT1 - [LCLT1_HUMAN]	1,48	0,29
Q96E11-3	Isoform 3 of Ribosome-recycling factor, mitochondrial OS=Homo sapiens GN=MRRF - [RRFM_HUMAN]	1,43	0,29
Q99650-2	Isoform 2 of Oncostatin-M-specific receptor subunit beta OS=Homo sapiens GN=OSMR - [OSMR_HUMAN]	1,46	0,29
O43920	NADH dehydrogenase [ubiquinone] iron-sulfur protein 5 OS=Homo sapiens GN=NDUFS5 PE=1 SV=3 - [NDUS5_HUM	1,44	0,29
Q96T83	Sodium/hydrogen exchanger 7 OS=Homo sapiens GN=SLC9A7 PE=1 SV=1 - [SL9A7_HUMAN]	1,36	0,29
O43181	NADH dehydrogenase [ubiquinone] iron-sulfur protein 4, mitochondrial OS=Homo sapiens GN=NDUFS4 PE=1 SV=1	1,45	0,29
Q9UBV7	Beta-1,4-galactosyltransferase 7 OS=Homo sapiens GN=B4GALT7 PE=1 SV=1 - [B4GT7_HUMAN]	1,72	0,29
Q9P032	NADH dehydrogenase [ubiquinone] 1 alpha subcomplex assembly factor 4 OS=Homo sapiens GN=NDUFAF4 PE=1 S\	1,41	0,29
Q92545	Transmembrane protein 131 OS=Homo sapiens GN=TMEM131 PE=1 SV=3 - [TM131_HUMAN]	1,34	0,36
Q96I45	Transmembrane protein 141 OS=Homo sapiens GN=TMEM141 PE=2 SV=1 - [TM141_HUMAN]	1,39	0,36
Q5SNT2	Transmembrane protein 201 OS=Homo sapiens GN=TMEM201 PE=1 SV=1 - [TM201_HUMAN]	1,43	0,36
P49959	Double-strand break repair protein MRE11A OS=Homo sapiens GN=MRE11A PE=1 SV=3 - [MRE11_HUMAN]	1,27	0,36
Q8NC60	Nitric oxide-associated protein 1 OS=Homo sapiens GN=NOA1 PE=1 SV=2 - [NOA1_HUMAN]	1,39	0,36
Q92804-2	Isoform Short of TATA-binding protein-associated factor 2N OS=Homo sapiens GN=TAF15 - [RBP56_HUMAN]	1,36	0,36
Q9P0J0	NADH dehydrogenase [ubiquinone] 1 alpha subcomplex subunit 13 OS=Homo sapiens GN=NDUFA13 PE=1 SV=3 - [N	1,45	0,36
Q8N3D4	EH domain-binding protein 1-like protein 1 OS=Homo sapiens GN=EHBP1L1 PE=1 SV=2 - [EH1L1_HUMAN]	1,38	0,36
O75947	ATP synthase subunit d, mitochondrial OS=Homo sapiens GN=ATP5H PE=1 SV=3 - [ATP5H_HUMAN]	1,39	0,36
P13645	Keratin, type I cytoskeletal 10 OS=Homo sapiens GN=KRT10 PE=1 SV=6 - [K1C10_HUMAN]	1,68	0,36

A375 vs. A375PR1

P51687	Sulfite oxidase, mitochondrial OS=Homo sapiens GN=SUOX PE=1 SV=2 - [SUOX_HUMAN]	1,40	0,36
P48552	Nuclear receptor-interacting protein 1 OS=Homo sapiens GN=NRIP1 PE=1 SV=2 - [NRIP1_HUMAN]	1,53	0,36
Q86TV6	Tetratricopeptide repeat protein 7B OS=Homo sapiens GN=TTC7B PE=1 SV=3 - [TTC7B_HUMAN]	1,74	0,36
P42785	Lysosomal Pro-X carboxypeptidase OS=Homo sapiens GN=PRCP PE=1 SV=1 - [PCP_HUMAN]	1,46	0,36
Q16643	Drebrin OS=Homo sapiens GN=DBN1 PE=1 SV=4 - [DREB_HUMAN]	1,29	0,36
P62072	Mitochondrial import inner membrane translocase subunit Tim10 OS=Homo sapiens GN=TIMM10 PE=1 SV=1 - [TIM	1,38	0,36
P42166	Lamina-associated polypeptide 2, isoform alpha OS=Homo sapiens GN=TMPO PE=1 SV=2 - [LAP2A_HUMAN]	1,61	0,36
Q5TEC3	Zinc finger protein 697 OS=Homo sapiens GN=ZNF697 PE=2 SV=2 - [ZN697_HUMAN]	1,58	0,36
Q9NYM9	BET1-like protein OS=Homo sapiens GN=BET1L PE=1 SV=1 - [BET1L_HUMAN]	1,36	0,36
O00479	High mobility group nucleosome-binding domain-containing protein 4 OS=Homo sapiens GN=HMGN4 PE=1 SV=3 - [	1,43	0,36
Q99674	Cell growth regulator with EF hand domain protein 1 OS=Homo sapiens GN=CGREF1 PE=2 SV=2 - [CGRE1_HUMAN]	1,39	0,36
Q6PL18	ATPase family AAA domain-containing protein 2 OS=Homo sapiens GN=ATAD2 PE=1 SV=1 - [ATAD2_HUMAN]	1,49	0,36
O75477	Erlin-1 OS=Homo sapiens GN=ERLIN1 PE=1 SV=1 - [ERLN1_HUMAN]	1,43	0,36
Q99828	Calcium and integrin-binding protein 1 OS=Homo sapiens GN=CIB1 PE=1 SV=4 - [CIB1_HUMAN]	1,36	0,36
P51648	Fatty aldehyde dehydrogenase OS=Homo sapiens GN=ALDH3A2 PE=1 SV=1 - [AL3A2_HUMAN]	1,43	0,36
O76024	Wolframin OS=Homo sapiens GN=WFS1 PE=1 SV=2 - [WFS1_HUMAN]	1,46	0,36
P46199	Translation initiation factor IF-2, mitochondrial OS=Homo sapiens GN=MTIF2 PE=1 SV=2 - [IF2M_HUMAN]	1,38	0,36
Q13608	Peroxisome assembly factor 2 OS=Homo sapiens GN=PEX6 PE=1 SV=2 - [PEX6_HUMAN]	1,35	0,36
Q6UW78	UPF0723 protein C11orf83 OS=Homo sapiens GN=C11orf83 PE=1 SV=2 - [CK083_HUMAN]	1,32	0,36
O75208	Ubiquinone biosynthesis protein COQ9, mitochondrial OS=Homo sapiens GN=COQ9 PE=1 SV=1 - [COQ9_HUMAN]	1,32	0,36
Q8IWT6	Leucine-rich repeat-containing protein 8A OS=Homo sapiens GN=LRRC8A PE=1 SV=1 - [LRC8A_HUMAN]	1,32	0,36
Q9H0R6	Glutamyl-tRNA(Gln) amidotransferase subunit A, mitochondrial OS=Homo sapiens GN=QRSL1 PE=1 SV=2 - [GATA_H	1,33	0,36
Q96EL2	28S ribosomal protein S24, mitochondrial OS=Homo sapiens GN=MRPS24 PE=1 SV=1 - [RT24_HUMAN]	1,37	0,36
P07196	Neurofilament light polypeptide OS=Homo sapiens GN=NEFL PE=1 SV=3 - [NFL_HUMAN]	1,34	0,36
Q9Y3D6	Mitochondrial fission 1 protein OS=Homo sapiens GN=FIS1 PE=1 SV=2 - [FIS1_HUMAN]	1,40	0,36
Q6PJG2	Uncharacterized protein C14orf43 OS=Homo sapiens GN=C14orf43 PE=1 SV=2 - [CN043_HUMAN]	1,38	0,36
Q6DKK2	Tetratricopeptide repeat protein 19, mitochondrial OS=Homo sapiens GN=TTC19 PE=1 SV=4 - [TTC19_HUMAN]	1,40	0,36
Q15738	Sterol-4-alpha-carboxylate 3-dehydrogenase, decarboxylating OS=Homo sapiens GN=NSDHL PE=1 SV=2 - [NSDHL_H	1,38	0,36
P61587	Rho-related GTP-binding protein RhoE OS=Homo sapiens GN=RND3 PE=1 SV=1 - [RND3_HUMAN]	1,74	0,36
Q9BSJ5-2	Isoform 2 of Uncharacterized protein C17orf80 OS=Homo sapiens GN=C17orf80 - [CQ080_HUMAN]	1,34	0,36
Q9NZ45	CDGSH iron-sulfur domain-containing protein 1 OS=Homo sapiens GN=CISD1 PE=1 SV=1 - [CISD1_HUMAN]	1,46	0,36
P45877	Peptidyl-prolyl cis-trans isomerase C OS=Homo sapiens GN=PPIC PE=1 SV=1 - [PPIC_HUMAN]	1,49	0,36
Q05639	Elongation factor 1-alpha 2 OS=Homo sapiens GN=EEF1A2 PE=1 SV=1 - [EF1A2_HUMAN]	3,93	0,36

A375 vs. A375PR1

Q12996	Cleavage stimulation factor subunit 3 OS=Homo sapiens GN=CSTF3 PE=1 SV=1 - [CSTF3_HUMAN]	1,37	0,36
Q9Y6X4	Protein FAM169A OS=Homo sapiens GN=FAM169A PE=1 SV=2 - [F169A_HUMAN]	1,33	0,36
Q03519-2	Isoform 2 of Antigen peptide transporter 2 OS=Homo sapiens GN=TAP2 - [TAP2_HUMAN]	1,39	0,36
Q14CZ7	FAST kinase domain-containing protein 3 OS=Homo sapiens GN=FASTKD3 PE=2 SV=2 - [FAKD3_HUMAN]	1,40	0,36
P07305-2	Isoform 2 of Histone H1,0 OS=Homo sapiens GN=H1F0 - [H10_HUMAN]	1,41	0,36
Q14254	Flotillin-2 OS=Homo sapiens GN=FLOT2 PE=1 SV=2 - [FLOT2_HUMAN]	1,34	0,36
Q99653	Calcium-binding protein p22 OS=Homo sapiens GN=CHP PE=1 SV=3 - [CHP1_HUMAN]	1,34	0,36
Q68CZ1-2	Isoform 2 of Protein fantom OS=Homo sapiens GN=RPGRIP1L - [FTM_HUMAN]	1,42	0,36
P53355	Death-associated protein kinase 1 OS=Homo sapiens GN=DAPK1 PE=1 SV=6 - [DAPK1_HUMAN]	1,32	0,36
P0DJ93	UPF0766 protein C6orf228 OS=Homo sapiens GN=C6orf228 PE=3 SV=1 - [CF228_HUMAN]	1,41	0,36
O95182	NADH dehydrogenase [ubiquinone] 1 alpha subcomplex subunit 7 OS=Homo sapiens GN=NDUFA7 PE=1 SV=3 - [NDI	1,35	0,36
Q9Y2K6	Ubiquitin carboxyl-terminal hydrolase 20 OS=Homo sapiens GN=USP20 PE=1 SV=2 - [UBP20_HUMAN]	1,40	0,36
Q96FL9-2	Isoform 2 of Polypeptide N-acetylgalactosaminyltransferase 14 OS=Homo sapiens GN=GALNT14 - [GLT14_HUMAN]	1,33	0,36
O14657	Torsin-1B OS=Homo sapiens GN=TOR1B PE=1 SV=2 - [TOR1B_HUMAN]	1,37	0,36
Q9NSI6-3	Isoform C of Bromodomain and WD repeat-containing protein 1 OS=Homo sapiens GN=BRWD1 - [BRWD1_HUMAN	1,32	0,36
O75054	Immunoglobulin superfamily member 3 OS=Homo sapiens GN=IGSF3 PE=1 SV=3 - [IGSF3_HUMAN]	1,36	0,36
P42765	3-ketoacyl-CoA thiolase, mitochondrial OS=Homo sapiens GN=ACAA2 PE=1 SV=2 - [THIM_HUMAN]	1,42	0,36
Q96GD4	Aurora kinase B OS=Homo sapiens GN=AURKB PE=1 SV=3 - [AURKB_HUMAN]	1,44	0,36
Q8WZ42-5	Isoform 5 of Titin OS=Homo sapiens GN=TTN - [TITIN_HUMAN]	1,40	0,36
**Gene ID**	**Gene Name**	**Fold Change**	**q-value(%)**
P07355	Annexin A2 OS=Homo sapiens GN=ANXA2 PE=1 SV=2 - [ANXA2_HUMAN]	0,55	0,17
P01906	HLA class II histocompatibility antigen, DQ alpha 2 chain OS=Homo sapiens GN=HLA-DQA2 PE=2 SV=2 - [DQA2_HUN	0,56	0,36

A375 vs. A375VR3

Gene ID	Gene Name	Fold Change	q-value(%)
P25815	Protein S100-P OS=Homo sapiens GN=S100P PE=1 SV=2 - [S100P_HUMAN]	805,55	0,00
O60732	Melanoma-associated antigen C1 OS=Homo sapiens GN=MAGEC1 PE=1 SV=3 - [MAGC1_HUMAN]	638279,27	0,00
Q99959-2	Isoform 1 of Plakophilin-2 OS=Homo sapiens GN=PKP2 - [PKP2_HUMAN]	5,35	0,00
Q96Q89-4	Isoform 4 of Kinesin-like protein KIF20B OS=Homo sapiens GN=KIF20B - [KI20B_HUMAN]	6,60	0,00
Q01543-2	Isoform 2 of Friend leukemia integration 1 transcription factor OS=Homo sapiens GN=FLI1 - [FLI1_HUMAN]	6,34	0,00
Q9NX02-3	Isoform 3 of NACHT, LRR and PYD domains-containing protein 2 OS=Homo sapiens GN=NLRP2 - [NALP2_HUMAN]	1740207,92	0,00
Q9Y446	Plakophilin-3 OS=Homo sapiens GN=PKP3 PE=1 SV=1 - [PKP3_HUMAN]	18,94	0,00
Q17RY6	Lymphocyte antigen 6K OS=Homo sapiens GN=LY6K PE=1 SV=2 - [LY6K_HUMAN]	21,50	0,00
Q7Z7L1	Schlafen family member 11 OS=Homo sapiens GN=SLFN11 PE=1 SV=2 - [SLN11_HUMAN]	7,48	0,00
P31321	cAMP-dependent protein kinase type I-beta regulatory subunit OS=Homo sapiens GN=PRKAR1B PE=1 SV=4 - [KAP1_HUMAI	2,77	0,00
P82970	High mobility group nucleosome-binding domain-containing protein 5 OS=Homo sapiens GN=HMGN5 PE=1 SV=1 - [HMGN5	17,58	0,00
P20138	Myeloid cell surface antigen CD33 OS=Homo sapiens GN=CD33 PE=1 SV=2 - [CD33_HUMAN]	11,56	0,00
P20337	Ras-related protein Rab-3B OS=Homo sapiens GN=RAB3B PE=1 SV=2 - [RAB3B_HUMAN]	2,34	0,00
Q9H6S3	Epidermal growth factor receptor kinase substrate 8-like protein 2 OS=Homo sapiens GN=EPS8L2 PE=1 SV=2 - [ES8L2_HUM	4,87	0,00
Q6KB66-2	Isoform 2 of Keratin, type II cytoskeletal 80 OS=Homo sapiens GN=KRT80 - [K2C80_HUMAN]	6,50	0,00
O75369-6	Isoform 6 of Filamin-B OS=Homo sapiens GN=FLNB - [FLNB_HUMAN]	4,14	0,00
Q99608	Necdin OS=Homo sapiens GN=NDN PE=2 SV=1 - [NECD_HUMAN]	6,65	0,00
Q13635-4	Isoform S of Protein patched homolog 1 OS=Homo sapiens GN=PTCH1 - [PTC1_HUMAN]	2,77	0,00
Q9H4G0-4	Isoform 4 of Band 4,1-like protein 1 OS=Homo sapiens GN=EPB41L1 - [E41L1_HUMAN]	3,44	0,00
Q96CF2	Charged multivesicular body protein 4c OS=Homo sapiens GN=CHMP4C PE=1 SV=1 - [CHM4C_HUMAN]	7,20	0,00
Q8TCU4-3	Isoform 3 of Alstrom syndrome protein 1 OS=Homo sapiens GN=ALMS1 - [ALMS1_HUMAN]	2,30	0,00
P20908	Collagen alpha-1(V) chain OS=Homo sapiens GN=COL5A1 PE=1 SV=3 - [CO5A1_HUMAN]	7,57	0,00
O75525-2	Isoform 2 of KH domain-containing, RNA-binding, signal transduction-associated protein 3 OS=Homo sapiens GN=KHDRBS3	16,06	0,00
Q6P9B6	TLD domain-containing protein KIAA1609 OS=Homo sapiens GN=KIAA1609 PE=1 SV=2 - [K1609_HUMAN]	1,84	0,00
P32189-1	Isoform 1 of Glycerol kinase OS=Homo sapiens GN=GK - [GLPK_HUMAN]	7,90	0,00
P35612	Beta-adducin OS=Homo sapiens GN=ADD2 PE=1 SV=3 - [ADDB_HUMAN]	3,71	0,00
Q9UBF1	Melanoma-associated antigen C2 OS=Homo sapiens GN=MAGEC2 PE=1 SV=1 - [MAGC2_HUMAN]	2,36	0,00
Q9BV40	Vesicle-associated membrane protein 8 OS=Homo sapiens GN=VAMP8 PE=1 SV=1 - [VAMP8_HUMAN]	4,84	0,00
Q92506	Estradiol 17-beta-dehydrogenase 8 OS=Homo sapiens GN=HSD17B8 PE=1 SV=2 - [DHB8_HUMAN]	2,51	0,00
O00220	Tumor necrosis factor receptor superfamily member 10A OS=Homo sapiens GN=TNFRSF10A PE=1 SV=3 - [TR10A_HUMAN]	4,94	0,00
P05120	Plasminogen activator inhibitor 2 OS=Homo sapiens GN=SERPINB2 PE=1 SV=2 - [PAI2_HUMAN]	36,00	0,00
Q15147-2	Isoform 1 of 1-phosphatidylinositol-4,5-bisphosphate phosphodiesterase beta-4 OS=Homo sapiens GN=PLCB4 - [PLCB4_HU	3,07	0,00

A375 vs. A375VR3

P26022	Pentraxin-related protein PTX3 OS=Homo sapiens GN=PTX3 PE=1 SV=3 - [PTX3_HUMAN]	4,59	0,00
P16144-4	Isoform Beta-4D of Integrin beta-4 OS=Homo sapiens GN=ITGB4 - [ITB4_HUMAN]	200,88	0,00
Q6P5R6	60S ribosomal protein L22-like 1 OS=Homo sapiens GN=RPL22L1 PE=1 SV=2 - [RL22L_HUMAN]	3,88	0,00
P05787	Keratin, type II cytoskeletal 8 OS=Homo sapiens GN=KRT8 PE=1 SV=7 - [K2C8_HUMAN]	22,65	0,00
P29317	Ephrin type-A receptor 2 OS=Homo sapiens GN=EPHA2 PE=1 SV=2 - [EPHA2_HUMAN]	6,52	0,00
Q9UKU6	Thyrotropin-releasing hormone-degrading ectoenzyme OS=Homo sapiens GN=TRHDE PE=2 SV=1 - [TRHDE_HUMAN]	60,82	0,00
A8K2U0	Alpha-2-macroglobulin-like protein 1 OS=Homo sapiens GN=A2ML1 PE=1 SV=3 - [A2ML1_HUMAN]	2,69	0,00
Q6ZMK1-2	Isoform 2 of Cysteine and histidine-rich protein 1 OS=Homo sapiens GN=CYHR1 - [CYHR1_HUMAN]	2,21	0,00
Q13188	Serine/threonine-protein kinase 3 OS=Homo sapiens GN=STK3 PE=1 SV=2 - [STK3_HUMAN]	1,84	0,00
Q9NUL3-3	Isoform 3 of Double-stranded RNA-binding protein Staufen homolog 2 OS=Homo sapiens GN=STAU2 - [STAU2_HUMAN]	1,76	0,00
Q5JRK9	Putative G antigen family E member 3 OS=Homo sapiens GN=PAGE2B PE=2 SV=1 - [GGEE3_HUMAN]	1341883,75	0,00
Q9BT78	COP9 signalosome complex subunit 4 OS=Homo sapiens GN=COPS4 PE=1 SV=1 - [CSN4_HUMAN]	1,74	0,00
P10620	Microsomal glutathione S-transferase 1 OS=Homo sapiens GN=MGST1 PE=1 SV=1 - [MGST1_HUMAN]	341,92	0,00
P43155-2	Isoform 2 of Carnitine O-acetyltransferase OS=Homo sapiens GN=CRAT - [CACP_HUMAN]	2,52	0,00
Q92466-2	Isoform D1 of DNA damage-binding protein 2 OS=Homo sapiens GN=DDB2 - [DDB2_HUMAN]	2,45	0,00
Q9UQ49	Sialidase-3 OS=Homo sapiens GN=NEU3 PE=1 SV=1 - [NEUR3_HUMAN]	2,48	0,00
P43003	Excitatory amino acid transporter 1 OS=Homo sapiens GN=SLC1A3 PE=1 SV=1 - [EAA1_HUMAN]	6,44	0,00
Q6ZUT6	Uncharacterized protein C15orf52 OS=Homo sapiens GN=C15orf52 PE=1 SV=1 - [CO052_HUMAN]	1,60	0,00
Q92905	COP9 signalosome complex subunit 5 OS=Homo sapiens GN=COPS5 PE=1 SV=4 - [CSN5_HUMAN]	1,68	0,00
P07203	Glutathione peroxidase 1 OS=Homo sapiens GN=GPX1 PE=1 SV=4 - [GPX1_HUMAN]	1,63	0,00
Q04727	Transducin-like enhancer protein 4 OS=Homo sapiens GN=TLE4 PE=1 SV=3 - [TLE4_HUMAN]	1,73	0,00
Q9P2K5-2	Isoform 2 of Myelin expression factor 2 OS=Homo sapiens GN=MYEF2 - [MYEF2_HUMAN]	4,34	0,00
Q9Y2T3	Guanine deaminase OS=Homo sapiens GN=GDA PE=1 SV=1 - [GUAD_HUMAN]	327,76	0,00
Q8IZD6	Solute carrier family 22 member 15 OS=Homo sapiens GN=SLC22A15 PE=2 SV=1 - [S22AF_HUMAN]	1,63	0,00
O60437	Periplakin OS=Homo sapiens GN=PPL PE=1 SV=4 - [PEPL_HUMAN]	4,73	0,00
Q15599	Na(+)/H(+) exchange regulatory cofactor NHE-RF2 OS=Homo sapiens GN=SLC9A3R2 PE=1 SV=2 - [NHRF2_HUMAN]	1,85	0,00
Q6XQN6-3	Isoform 3 of Nicotinate phosphoribosyltransferase OS=Homo sapiens GN=NAPRT1 - [PNCB_HUMAN]	155,90	0,00
P10301	Ras-related protein R-Ras OS=Homo sapiens GN=RRAS PE=1 SV=1 - [RRAS_HUMAN]	2,33	0,00
P56817-4	Isoform D of Beta-secretase 1 OS=Homo sapiens GN=BACE1 - [BACE1_HUMAN]	3,11	0,00
Q96IF1	LIM domain-containing protein ajuba OS=Homo sapiens GN=AJUBA PE=1 SV=1 - [AJUBA_HUMAN]	1,99	0,00
Q01955	Collagen alpha-3(IV) chain OS=Homo sapiens GN=COL4A3 PE=1 SV=3 - [CO4A3_HUMAN]	1,67	0,00
Q8N3D4	EH domain-binding protein 1-like protein 1 OS=Homo sapiens GN=EHBP1L1 PE=1 SV=2 - [EH1L1_HUMAN]	1,64	0,00
P15144	Aminopeptidase N OS=Homo sapiens GN=ANPEP PE=1 SV=4 - [AMPN_HUMAN]	7,47	0,00

A375 vs. A375VR3

Q9H6F2	Trimeric intracellular cation channel type A OS=Homo sapiens GN=TMEM38A PE=1 SV=1 - [TM38A_HUMAN]	3,80	0,00
Q8IWR1	Tripartite motif-containing protein 59 OS=Homo sapiens GN=TRIM59 PE=2 SV=1 - [TRI59_HUMAN]	2,67	0,00
P15328	Folate receptor alpha OS=Homo sapiens GN=FOLR1 PE=1 SV=3 - [FOLR1_HUMAN]	7,44	0,00
P10696	Alkaline phosphatase, placental-like OS=Homo sapiens GN=ALPPL2 PE=1 SV=4 - [PPBN_HUMAN]	13,02	0,00
O14657	Torsin-1B OS=Homo sapiens GN=TOR1B PE=1 SV=2 - [TOR1B_HUMAN]	3,61	0,00
Q13887	Krueppel-like factor 5 OS=Homo sapiens GN=KLF5 PE=1 SV=2 - [KLF5_HUMAN]	3,23	0,00
Q86UL8-2	Isoform 2 of Membrane-associated guanylate kinase, WW and PDZ domain-containing protein 2 OS=Homo sapiens GN=MA	21,86	0,00
Q9NS25	Sperm protein associated with the nucleus on the X chromosome B/F OS=Homo sapiens GN=SPANXB1 PE=2 SV=1 - [SPNXB	35,35	0,00
Q86WV6	Transmembrane protein 173 OS=Homo sapiens GN=TMEM173 PE=1 SV=1 - [TM173_HUMAN]	2,89	0,00
Q16270	Insulin-like growth factor-binding protein 7 OS=Homo sapiens GN=IGFBP7 PE=1 SV=1 - [IBP7_HUMAN]	2,02	0,00
Q9HAU0-5	Isoform 5 of Pleckstrin homology domain-containing family A member 5 OS=Homo sapiens GN=PLEKHA5 - [PKHA5_HUMAN	2,05	0,00
Q86SJ2	Amphoterin-induced protein 2 OS=Homo sapiens GN=AMIGO2 PE=1 SV=1 - [AMGO2_HUMAN]	17,25	0,00
P08779	Keratin, type I cytoskeletal 16 OS=Homo sapiens GN=KRT16 PE=1 SV=4 - [K1C16_HUMAN]	1,62	0,00
Q8N8U2	Chromodomain Y-like protein 2 OS=Homo sapiens GN=CDYL2 PE=2 SV=2 - [CDYL2_HUMAN]	2,19	0,00
Q8IZV5	Retinol dehydrogenase 10 OS=Homo sapiens GN=RDH10 PE=1 SV=1 - [RDH10_HUMAN]	2,12	0,00
Q9UBW8	COP9 signalosome complex subunit 7a OS=Homo sapiens GN=COPS7A PE=1 SV=1 - [CSN7A_HUMAN]	1,70	0,00
P07196	Neurofilament light polypeptide OS=Homo sapiens GN=NEFL PE=1 SV=3 - [NFL_HUMAN]	7,16	0,00
P32970	CD70 antigen OS=Homo sapiens GN=CD70 PE=1 SV=2 - [CD70_HUMAN]	1,83	0,00
Q969U6	F-box/WD repeat-containing protein 5 OS=Homo sapiens GN=FBXW5 PE=1 SV=1 - [FBXW5_HUMAN]	1,97	0,00
O15020-2	Isoform 2 of Spectrin beta chain, brain 2 OS=Homo sapiens GN=SPTBN2 - [SPTN2_HUMAN]	2,06	0,00
Q9H936	Mitochondrial glutamate carrier 1 OS=Homo sapiens GN=SLC25A22 PE=1 SV=1 - [GHC1_HUMAN]	2,62	0,00
Q9NQS3	Poliovirus receptor-related protein 3 OS=Homo sapiens GN=PVRL3 PE=1 SV=1 - [PVRL3_HUMAN]	1,78	0,00
Q02930-4	Isoform 4 of Cyclic AMP-responsive element-binding protein 5 OS=Homo sapiens GN=CREB5 - [CREB5_HUMAN]	1,88	0,00
Q14160	Protein scribble homolog OS=Homo sapiens GN=SCRIB PE=1 SV=4 - [SCRIB_HUMAN]	1,64	0,00
Q5T5S1	Uncharacterized coiled-coil domain-containing protein KIAA1984 OS=Homo sapiens GN=KIAA1984 PE=2 SV=3 - [K1984_HU	41,46	0,00
Q96K19-5	Isoform 5 of RING finger protein 170 OS=Homo sapiens GN=RNF170 - [RN170_HUMAN]	1,73	0,00
Q08AF3	Schlafen family member 5 OS=Homo sapiens GN=SLFN5 PE=1 SV=1 - [SLFN5_HUMAN]	1,72	0,00
Q9NSV4-1	Isoform 1 of Protein diaphanous homolog 3 OS=Homo sapiens GN=DIAPH3 - [DIAP3_HUMAN]	1,83	0,00
O95466	Formin-like protein 1 OS=Homo sapiens GN=FMNL1 PE=1 SV=3 - [FMNL_HUMAN]	1,75	0,00
Q9HBH0	Rho-related GTP-binding protein RhoF OS=Homo sapiens GN=RHOF PE=2 SV=1 - [RHOF_HUMAN]	20,55	0,00
Q6SJ93	Protein FAM111B OS=Homo sapiens GN=FAM111B PE=2 SV=1 - [F111B_HUMAN]	1,48	0,00
Q8N3E9	1-phosphatidylinositol-4,5-bisphosphate phosphodiesterase delta-3 OS=Homo sapiens GN=PLCD3 PE=1 SV=3 - [PLCD3_HUN	1,80	0,00
Q8N4A0	Polypeptide N-acetylgalactosaminyltransferase 4 OS=Homo sapiens GN=GALNT4 PE=1 SV=2 - [GALT4_HUMAN]	1,68	0,00

A375 vs. A375VR3

Q9NPH2-2	Isoform 2 of Inositol-3-phosphate synthase 1 OS=Homo sapiens GN=ISYNA1 - [INO1_HUMAN]	2,34	0,00
Q15746-4	Isoform 3B of Myosin light chain kinase, smooth muscle OS=Homo sapiens GN=MYLK - [MYLK_HUMAN]	1,69	0,00
P0C2W1	F-box/SPRY domain-containing protein 1 OS=Homo sapiens GN=FBXO45 PE=1 SV=1 - [FBSP1_HUMAN]	1,54	0,00
Q9UIV1	CCR4-NOT transcription complex subunit 7 OS=Homo sapiens GN=CNOT7 PE=1 SV=3 - [CNOT7_HUMAN]	1,42	0,00
P20592	Interferon-induced GTP-binding protein Mx2 OS=Homo sapiens GN=MX2 PE=1 SV=1 - [MX2_HUMAN]	1,45	0,00
Q9NVH6-2	Isoform B of Trimethyllysine dioxygenase, mitochondrial OS=Homo sapiens GN=TMLHE - [TMLH_HUMAN]	1,80	0,00
Q0ZGT2-2	Isoform 2 of Nexilin OS=Homo sapiens GN=NEXN - [NEXN_HUMAN]	2,10	0,00
O14639	Actin-binding LIM protein 1 OS=Homo sapiens GN=ABLIM1 PE=1 SV=3 - [ABLM1_HUMAN]	1,63	0,00
P42785	Lysosomal Pro-X carboxypeptidase OS=Homo sapiens GN=PRCP PE=1 SV=1 - [PCP_HUMAN]	1,36	0,00
A5D8V6	Vacuolar protein sorting-associated protein 37C OS=Homo sapiens GN=VPS37C PE=1 SV=2 - [VP37C_HUMAN]	1,93	0,00
O15479	Melanoma-associated antigen B2 OS=Homo sapiens GN=MAGEB2 PE=1 SV=3 - [MAGB2_HUMAN]	2,28	0,00
P29034	Protein S100-A2 OS=Homo sapiens GN=S100A2 PE=1 SV=3 - [S10A2_HUMAN]	2,89	0,00
O15455	Toll-like receptor 3 OS=Homo sapiens GN=TLR3 PE=1 SV=1 - [TLR3_HUMAN]	1,77	0,00
Q8IWS0	PHD finger protein 6 OS=Homo sapiens GN=PHF6 PE=1 SV=1 - [PHF6_HUMAN]	1,36	0,00
P13987	CD59 glycoprotein OS=Homo sapiens GN=CD59 PE=1 SV=1 - [CD59_HUMAN]	1,96	0,00
Q6PEV8-2	Isoform 2 of Protein FAM199X OS=Homo sapiens GN=FAM199X - [F199X_HUMAN]	1,76	0,00
Q14764	Major vault protein OS=Homo sapiens GN=MVP PE=1 SV=4 - [MVP_HUMAN]	2,27	0,00
P26006	Integrin alpha-3 OS=Homo sapiens GN=ITGA3 PE=1 SV=5 - [ITA3_HUMAN]	1,78	0,00
O60763	General vesicular transport factor p115 OS=Homo sapiens GN=USO1 PE=1 SV=2 - [USO1_HUMAN]	1,75	0,00
O15344-2	Isoform 2 of Midline-1 OS=Homo sapiens GN=MID1 - [TRI18_HUMAN]	1,65	0,00
Q07157	Tight junction protein ZO-1 OS=Homo sapiens GN=TJP1 PE=1 SV=3 - [ZO1_HUMAN]	1,37	0,00
Q9NY43	BarH-like 2 homeobox protein OS=Homo sapiens GN=BARHL2 PE=2 SV=2 - [BARH2_HUMAN]	1,40	0,00
Q9HBM0	Vezatin OS=Homo sapiens GN=VEZT PE=1 SV=3 - [VEZA_HUMAN]	1,63	0,00
Q53EU6	Glycerol-3-phosphate acyltransferase 3 OS=Homo sapiens GN=AGPAT9 PE=1 SV=2 - [GPAT3_HUMAN]	2,40	0,00
Q13015	Protein AF1q OS=Homo sapiens GN=MLLT11 PE=2 SV=1 - [AF1Q_HUMAN]	1,88	0,00
Q8N4T8-2	Isoform 2 of Carbonyl reductase family member 4 OS=Homo sapiens GN=CBR4 - [CBR4_HUMAN]	1,67	0,00
Q96C92-4	Isoform 4 of Serologically defined colon cancer antigen 3 OS=Homo sapiens GN=SDCCAG3 - [SDCG3_HUMAN]	1,42	0,00
O60831	PRA1 family protein 2 OS=Homo sapiens GN=PRAF2 PE=1 SV=1 - [PRAF2_HUMAN]	1,73	0,00
P53365	Arfaptin-2 OS=Homo sapiens GN=ARFIP2 PE=1 SV=1 - [ARFP2_HUMAN]	1,74	0,00
Q8IZR5-3	Isoform 3 of CKLF-like MARVEL transmembrane domain-containing protein 4 OS=Homo sapiens GN=CMTM4 - [CKLF4_HUM	1,72	0,00
Q9BSU3	N-alpha-acetyltransferase 11 OS=Homo sapiens GN=NAA11 PE=1 SV=3 - [NAA11_HUMAN]	2,15	0,00
O95816	BAG family molecular chaperone regulator 2 OS=Homo sapiens GN=BAG2 PE=1 SV=1 - [BAG2_HUMAN]	1,56	0,00
O43790	Keratin, type II cuticular Hb6 OS=Homo sapiens GN=KRT86 PE=1 SV=1 - [KRT86_HUMAN]	1,45	0,00

A375 vs. A375VR3

O75051	Plexin-A2 OS=Homo sapiens GN=PLXNA2 PE=1 SV=4 - [PLXA2_HUMAN]	2,00	0,00
Q9UN86-2	Isoform B of Ras GTPase-activating protein-binding protein 2 OS=Homo sapiens GN=G3BP2 - [G3BP2_HUMAN]	1,52	0,00
Q96B70	Leukocyte receptor cluster member 9 OS=Homo sapiens GN=LENG9 PE=2 SV=2 - [LENG9_HUMAN]	2,17	0,00
P11117	Lysosomal acid phosphatase OS=Homo sapiens GN=ACP2 PE=1 SV=3 - [PPAL_HUMAN]	1,59	0,00
Q9BUK0	Coiled-coil-helix-coiled-coil-helix domain-containing protein 7 OS=Homo sapiens GN=CHCHD7 PE=2 SV=1 - [CHCH7_HUMAN	1,74	0,00
Q15149-8	Isoform 8 of Plectin OS=Homo sapiens GN=PLEC - [PLEC_HUMAN]	2,25	0,00
Q9UK99-3	Isoform 3 of F-box only protein 3 OS=Homo sapiens GN=FBXO3 - [FBX3_HUMAN]	1,48	0,00
Q5XG87	DNA polymerase sigma OS=Homo sapiens GN=PAPD7 PE=1 SV=2 - [PAPD7_HUMAN]	1,42	0,00
P22570	NADPH:adrenodoxin oxidoreductase, mitochondrial OS=Homo sapiens GN=FDXR PE=1 SV=3 - [ADRO_HUMAN]	1,72	0,00
Q00013	55 kDa erythrocyte membrane protein OS=Homo sapiens GN=MPP1 PE=1 SV=2 - [EM55_HUMAN]	1,34	0,00
Q92820	Gamma-glutamyl hydrolase OS=Homo sapiens GN=GGH PE=1 SV=2 - [GGH_HUMAN]	1,44	0,00
Q9UNW1	Multiple inositol polyphosphate phosphatase 1 OS=Homo sapiens GN=MINPP1 PE=1 SV=1 - [MINP1_HUMAN]	1,91	0,00
Q86UT6	NLR family member X1 OS=Homo sapiens GN=NLRX1 PE=1 SV=1 - [NLRX1_HUMAN]	2,04	0,00
P42166	Lamina-associated polypeptide 2, isoform alpha OS=Homo sapiens GN=TMPO PE=1 SV=2 - [LAP2A_HUMAN]	1,67	0,00
Q16531	DNA damage-binding protein 1 OS=Homo sapiens GN=DDB1 PE=1 SV=1 - [DDB1_HUMAN]	1,37	0,00
O60427	Fatty acid desaturase 1 OS=Homo sapiens GN=FADS1 PE=1 SV=1 - [FADS1_HUMAN]	2,94	0,00
P14210-6	Isoform 6 of Hepatocyte growth factor OS=Homo sapiens GN=HGF - [HGF_HUMAN]	3,83	0,00
Q14332	Frizzled-2 OS=Homo sapiens GN=FZD2 PE=2 SV=1 - [FZD2_HUMAN]	1,58	0,00
P02538	Keratin, type II cytoskeletal 6A OS=Homo sapiens GN=KRT6A PE=1 SV=3 - [K2C6A_HUMAN]	6,68	0,00
Q7L5N1	COP9 signalosome complex subunit 6 OS=Homo sapiens GN=COPS6 PE=1 SV=1 - [CSN6_HUMAN]	1,40	0,00
Q8N3V7-2	Isoform 2 of Synaptopodin OS=Homo sapiens GN=SYNPO - [SYNPO_HUMAN]	1,68	0,00
Q13813	Spectrin alpha chain, brain OS=Homo sapiens GN=SPTAN1 PE=1 SV=3 - [SPTA2_HUMAN]	1,73	0,00
Q8TEM1	Nuclear pore membrane glycoprotein 210 OS=Homo sapiens GN=NUP210 PE=1 SV=3 - [PO210_HUMAN]	3,01	0,00
A6NP61	ZAR1-like protein OS=Homo sapiens GN=ZAR1L PE=2 SV=2 - [ZAR1L_HUMAN]	2,53	0,00
Q8TB37-2	Isoform 2 of Iron-sulfur protein NUBPL OS=Homo sapiens GN=NUBPL - [NUBPL_HUMAN]	1,62	0,00
Q8IVF2-3	Isoform 3 of Protein AHNAK2 OS=Homo sapiens GN=AHNAK2 - [AHNK2_HUMAN]	1,46	0,00
Q52LW3	Rho GTPase-activating protein 29 OS=Homo sapiens GN=ARHGAP29 PE=1 SV=2 - [RHG29_HUMAN]	1,60	0,00
Q15834	Coiled-coil domain-containing protein 85B OS=Homo sapiens GN=CCDC85B PE=1 SV=2 - [CC85B_HUMAN]	1,55	0,00
P56199	Integrin alpha-1 OS=Homo sapiens GN=ITGA1 PE=1 SV=2 - [ITA1_HUMAN]	1,91	0,00
Q13825-2	Isoform 2 of Methylglutaconyl-CoA hydratase, mitochondrial OS=Homo sapiens GN=AUH - [AUHM_HUMAN]	1,50	0,00
Q71RG4-3	Isoform 3 of Transmembrane and ubiquitin-like domain-containing protein 2 OS=Homo sapiens GN=TMUB2 - [TMUB2_HUM	1,73	0,00
P80723	Brain acid soluble protein 1 OS=Homo sapiens GN=BASP1 PE=1 SV=2 - [BASP1_HUMAN]	3,01	0,00
P04004	Vitronectin OS=Homo sapiens GN=VTN PE=1 SV=1 - [VTNC_HUMAN]	2,61	0,00

A375 vs. A375VR3

P40200-2	Isoform 2 of T-cell surface protein tactile OS=Homo sapiens GN=CD96 - [TACT_HUMAN]	2,05	0,00
P49748-2	Isoform 2 of Very long-chain specific acyl-CoA dehydrogenase, mitochondrial OS=Homo sapiens GN=ACADVL - [ACADV_HUI	1,54	0,00
O75386	Tubby-related protein 3 OS=Homo sapiens GN=TULP3 PE=1 SV=2 - [TULP3_HUMAN]	1,36	0,00
Q99653	Calcium-binding protein p22 OS=Homo sapiens GN=CHP PE=1 SV=3 - [CHP1_HUMAN]	1,67	0,00
Q9Y4K1	Absent in melanoma 1 protein OS=Homo sapiens GN=AIM1 PE=1 SV=3 - [AIM1_HUMAN]	1,50	0,33
O95772-2	Isoform 2 of MLN64 N-terminal domain homolog OS=Homo sapiens GN=STARD3NL - [MENTO_HUMAN]	2,05	0,33
P08581	Hepatocyte growth factor receptor OS=Homo sapiens GN=MET PE=1 SV=4 - [MET_HUMAN]	1,77	0,33
O60907-2	Isoform 2 of F-box-like/WD repeat-containing protein TBL1X OS=Homo sapiens GN=TBL1X - [TBL1X_HUMAN]	1,55	0,33
Q9H8Y8-2	Isoform 2 of Golgi reassembly-stacking protein 2 OS=Homo sapiens GN=GORASP2 - [GORS2_HUMAN]	1,33	0,33
Q96EQ0	Small glutamine-rich tetratricopeptide repeat-containing protein beta OS=Homo sapiens GN=SGTB PE=1 SV=1 - [SGTB_HUM	1,45	0,33
O94766	Galactosylgalactosylxylosylprotein 3-beta-glucuronosyltransferase 3 OS=Homo sapiens GN=B3GAT3 PE=1 SV=2 - [B3GA3_H	2,22	0,33
P50895	Basal cell adhesion molecule OS=Homo sapiens GN=BCAM PE=1 SV=2 - [BCAM_HUMAN]	1,62	0,33
Q8IY33-3	Isoform 3 of MICAL-like protein 2 OS=Homo sapiens GN=MICALL2 - [MILK2_HUMAN]	1,44	0,33
O60220	Mitochondrial import inner membrane translocase subunit Tim8 A OS=Homo sapiens GN=TIMM8A PE=1 SV=1 - [TIM8A_HU	1,82	0,33
Q13488	V-type proton ATPase 116 kDa subunit a isoform 3 OS=Homo sapiens GN=TCIRG1 PE=1 SV=3 - [VPP3_HUMAN]	1,53	0,33
P84095	Rho-related GTP-binding protein RhoG OS=Homo sapiens GN=RHOG PE=1 SV=1 - [RHOG_HUMAN]	1,72	0,33
Q9BW91-2	Isoform 2 of ADP-ribose pyrophosphatase, mitochondrial OS=Homo sapiens GN=NUDT9 - [NUDT9_HUMAN]	1,60	0,33
Q5QP82-2	Isoform 2 of DDB1- and CUL4-associated factor 10 OS=Homo sapiens GN=DCAF10 - [DCA10_HUMAN]	1,51	0,33
Q92985-3	Isoform C of Interferon regulatory factor 7 OS=Homo sapiens GN=IRF7 - [IRF7_HUMAN]	1,57	0,33
P43353-2	Isoform 2 of Aldehyde dehydrogenase family 3 member B1 OS=Homo sapiens GN=ALDH3B1 - [AL3B1_HUMAN]	2,12	0,33
P26885	Peptidyl-prolyl cis-trans isomerase FKBP2 OS=Homo sapiens GN=FKBP2 PE=1 SV=2 - [FKBP2_HUMAN]	1,79	0,33
P51828	Adenylate cyclase type 7 OS=Homo sapiens GN=ADCY7 PE=2 SV=1 - [ADCY7_HUMAN]	1,44	0,33
Q9UQ03-2	Isoform 2 of Coronin-2B OS=Homo sapiens GN=CORO2B - [COR2B_HUMAN]	1,43	0,33
Q01650	Large neutral amino acids transporter small subunit 1 OS=Homo sapiens GN=SLC7A5 PE=1 SV=2 - [LAT1_HUMAN]	1,78	0,33
Q13938	Calcyphosin OS=Homo sapiens GN=CAPS PE=1 SV=1 - [CAYP1_HUMAN]	6,80	0,33
Q15526-2	Isoform 2 of Surfeit locus protein 1 OS=Homo sapiens GN=SURF1 - [SURF1_HUMAN]	2,06	0,33
Q96RT1-7	Isoform 7 of Protein LAP2 OS=Homo sapiens GN=ERBB2IP - [LAP2_HUMAN]	1,40	0,33
Q16877	6-phosphofructo-2-kinase/fructose-2,6-biphosphatase 4 OS=Homo sapiens GN=PFKFB4 PE=2 SV=6 - [F264_HUMAN]	1,32	0,33
P52758	Ribonuclease UK114 OS=Homo sapiens GN=HRSP12 PE=1 SV=1 - [UK114_HUMAN]	1,60	0,33
Q8NBJ7-2	Isoform 2 of Sulfatase-modifying factor 2 OS=Homo sapiens GN=SUMF2 - [SUMF2_HUMAN]	1,57	0,33
O60716-5	Isoform 1A of Catenin delta-1 OS=Homo sapiens GN=CTNND1 - [CTND1_HUMAN]	1,29	0,33
Q9NZI8	Insulin-like growth factor 2 mRNA-binding protein 1 OS=Homo sapiens GN=IGF2BP1 PE=1 SV=2 - [IF2B1_HUMAN]	1,53	0,33
Q9HBH5	Retinol dehydrogenase 14 OS=Homo sapiens GN=RDH14 PE=1 SV=1 - [RDH14_HUMAN]	1,69	0,33

A375 vs. A375VR3

P79522-2	Isoform 2 of Proline-rich protein 3 OS=Homo sapiens GN=PRR3 - [PRR3_HUMAN]	1,41	0,33
P50222	Homeobox protein MOX-2 OS=Homo sapiens GN=MEOX2 PE=1 SV=2 - [MEOX2_HUMAN]	1,71	0,33
Q96C36	Pyrroline-5-carboxylate reductase 2 OS=Homo sapiens GN=PYCR2 PE=1 SV=1 - [P5CR2_HUMAN]	1,68	0,33
P29279-2	Isoform 2 of Connective tissue growth factor OS=Homo sapiens GN=CTGF - [CTGF_HUMAN]	1,67	0,33
Q12959-4	Isoform 4 of Disks large homolog 1 OS=Homo sapiens GN=DLG1 - [DLG1_HUMAN]	1,46	0,33
P0C7T5	Ataxin-1-like OS=Homo sapiens GN=ATXN1L PE=1 SV=1 - [ATX1L_HUMAN]	1,36	0,33
Q9UPQ8	Dolichol kinase OS=Homo sapiens GN=DOLK PE=1 SV=1 - [DOLK_HUMAN]	1,82	0,33
Q8NHG7	Small VCP/p97-interacting protein OS=Homo sapiens GN=SVIP PE=2 SV=1 - [SVIP_HUMAN]	1,52	0,33
Q9NTG7	NAD-dependent deacetylase sirtuin-3, mitochondrial OS=Homo sapiens GN=SIRT3 PE=1 SV=2 - [SIRT3_HUMAN]	1,63	0,33
Q96P48-5	Isoform 5 of Arf-GAP with Rho-GAP domain, ANK repeat and PH domain-containing protein 1 OS=Homo sapiens GN=ARAP1	1,94	0,33
O00622	Protein CYR61 OS=Homo sapiens GN=CYR61 PE=1 SV=1 - [CYR61_HUMAN]	1,96	0,33
Q96SW2-2	Isoform 2 of Protein cereblon OS=Homo sapiens GN=CRBN - [CRBN_HUMAN]	1,89	0,33
P30044-2	Isoform Cytoplasmic+peroxisomal of Peroxiredoxin-5, mitochondrial OS=Homo sapiens GN=PRDX5 - [PRDX5_HUMAN]	1,33	0,33
O14686	Histone-lysine N-methyltransferase MLL2 OS=Homo sapiens GN=MLL2 PE=1 SV=2 - [MLL2_HUMAN]	1,61	0,33
Q9BQ69	MACRO domain-containing protein 1 OS=Homo sapiens GN=MACROD1 PE=1 SV=2 - [MACD1_HUMAN]	1,38	0,33
Q9H3Q1	Cdc42 effector protein 4 OS=Homo sapiens GN=CDC42EP4 PE=1 SV=1 - [BORG4_HUMAN]	1,67	0,33
Q9UHL4	Dipeptidyl peptidase 2 OS=Homo sapiens GN=DPP7 PE=1 SV=3 - [DPP2_HUMAN]	1,35	0,33
Q9Y2H1	Serine/threonine-protein kinase 38-like OS=Homo sapiens GN=STK38L PE=1 SV=3 - [ST38L_HUMAN]	1,72	0,33
Q14534	Squalene monooxygenase OS=Homo sapiens GN=SQLE PE=2 SV=3 - [ERG1_HUMAN]	1,57	0,33
Q8WUK0-2	Isoform 2 of Protein-tyrosine phosphatase mitochondrial 1 OS=Homo sapiens GN=PTPMT1 - [PTPM1_HUMAN]	1,74	0,33
O00488	Zinc finger protein 593 OS=Homo sapiens GN=ZNF593 PE=1 SV=2 - [ZN593_HUMAN]	1,41	0,33
Q9BRU9	rRNA-processing protein UTP23 homolog OS=Homo sapiens GN=UTP23 PE=2 SV=2 - [UTP23_HUMAN]	1,37	0,33
P78358-2	Isoform 2 of Cancer/testis antigen 1 OS=Homo sapiens GN=CTAG1A - [CTG1B_HUMAN]	1,42	0,33
Q9BSM1-3	Isoform 2 of Polycomb group RING finger protein 1 OS=Homo sapiens GN=PCGF1 - [PCGF1_HUMAN]	1,85	0,33
Q9BSD7	Cancer-related nucleoside-triphosphatase OS=Homo sapiens GN=NTPCR PE=1 SV=1 - [NTPCR_HUMAN]	1,51	0,33
Q3KR37-2	Isoform 2 of GRAM domain-containing protein 1B OS=Homo sapiens GN=GRAMD1B - [GRM1B_HUMAN]	1,80	0,33
Q5JTD0-2	Isoform 2 of Tight junction-associated protein 1 OS=Homo sapiens GN=TJAP1 - [TJAP1_HUMAN]	1,63	0,33
Q96RS6	NudC domain-containing protein 1 OS=Homo sapiens GN=NUDCD1 PE=1 SV=2 - [NUDC1_HUMAN]	1,54	0,33
O00469-2	Isoform 2 of Procollagen-lysine,2-oxoglutarate 5-dioxygenase 2 OS=Homo sapiens GN=PLOD2 - [PLOD2_HUMAN]	1,46	0,33
P08195	4F2 cell-surface antigen heavy chain OS=Homo sapiens GN=SLC3A2 PE=1 SV=3 - [4F2_HUMAN]	1,63	0,33
Q9H568	Actin-like protein 8 OS=Homo sapiens GN=ACTL8 PE=2 SV=1 - [ACTL8_HUMAN]	2,28	0,33
Q53EL6	Programmed cell death protein 4 OS=Homo sapiens GN=PDCD4 PE=1 SV=2 - [PDCD4_HUMAN]	1,69	0,33
**Gene ID**	**Gene Name**	**Fold Change**	**q-value(%)**

A375 vs. A375VR3

Q9UHT9	Putative uncharacterized protein PRO1768 OS=Homo sapiens GN=PRO1768 PE=5 SV=1 - [YN005_HUMAN]	0,51	0,00
Q96RW7-2	Isoform 2 of Hemicentin-1 OS=Homo sapiens GN=HMCN1 - [HMCN1_HUMAN]	0,60	0,00
Q96MK3	Protein FAM20A OS=Homo sapiens GN=FAM20A PE=1 SV=4 - [FA20A_HUMAN]	0,57	0,00
Q92626	Peroxidasin homolog OS=Homo sapiens GN=PXDN PE=1 SV=2 - [PXDN_HUMAN]	0,60	0,00
P98196	Probable phospholipid-transporting ATPase IH OS=Homo sapiens GN=ATP11A PE=2 SV=3 - [AT11A_HUMAN]	0,65	0,00
Q8N5Z5-2	Isoform 2 of BTB/POZ domain-containing protein KCTD17 OS=Homo sapiens GN=KCTD17 - [KCD17_HUMAN]	0,65	0,00
Q9H4M7	Pleckstrin homology domain-containing family A member 4 OS=Homo sapiens GN=PLEKHA4 PE=1 SV=2 - [PKHA4_HUMAN]	0,67	0,00
Q6ZNJ1-4	Isoform 4 of Neurobeachin-like protein 2 OS=Homo sapiens GN=NBEAL2 - [NBEL2_HUMAN]	0,68	0,00
Q96HQ2	CDKN2AIP N-terminal-like protein OS=Homo sapiens GN=CDKN2AIPNL PE=1 SV=1 - [C2AIL_HUMAN]	0,65	0,00
Q14644	Ras GTPase-activating protein 3 OS=Homo sapiens GN=RASA3 PE=1 SV=3 - [RASA3_HUMAN]	0,65	0,00
P46013-2	Isoform Short of Antigen KI-67 OS=Homo sapiens GN=MKI67 - [KI67_HUMAN]	0,69	0,00
P24557	Thromboxane-A synthase OS=Homo sapiens GN=TBXAS1 PE=1 SV=3 - [THAS_HUMAN]	0,59	0,00
Q9HA77	Probable cysteine--tRNA ligase, mitochondrial OS=Homo sapiens GN=CARS2 PE=1 SV=1 - [SYCM_HUMAN]	0,68	0,00
P09471-2	Isoform Alpha-2 of Guanine nucleotide-binding protein G(o) subunit alpha OS=Homo sapiens GN=GNAO1 - [GNAO_HUMAN	0,66	0,00
P16112-3	Isoform 3 of Aggrecan core protein OS=Homo sapiens GN=ACAN - [PGCA_HUMAN]	0,63	0,00
Q7L311	Armadillo repeat-containing X-linked protein 2 OS=Homo sapiens GN=ARMCX2 PE=2 SV=1 - [ARMX2_HUMAN]	0,66	0,00
Q8NI37	Protein phosphatase PTC7 homolog OS=Homo sapiens GN=PPTC7 PE=2 SV=1 - [PPTC7_HUMAN]	0,68	0,00
Q9P2F8	Signal-induced proliferation-associated 1-like protein 2 OS=Homo sapiens GN=SIPA1L2 PE=1 SV=2 - [SI1L2_HUMAN]	0,74	0,00
P06858	Lipoprotein lipase OS=Homo sapiens GN=LPL PE=1 SV=1 - [LIPL_HUMAN]	0,59	0,00
Q8WY21-3	Isoform 3 of VPS10 domain-containing receptor SorCS1 OS=Homo sapiens GN=SORCS1 - [SORC1_HUMAN]	0,62	0,00
Q9H246	Uncharacterized protein C1orf21 OS=Homo sapiens GN=C1orf21 PE=1 SV=1 - [CA021_HUMAN]	0,70	0,00
P20336	Ras-related protein Rab-3A OS=Homo sapiens GN=RAB3A PE=1 SV=1 - [RAB3A_HUMAN]	0,68	0,00
Q9NS69	Mitochondrial import receptor subunit TOM22 homolog OS=Homo sapiens GN=TOMM22 PE=1 SV=3 - [TOM22_HUMAN]	0,53	0,00
P15907	Beta-galactoside alpha-2,6-sialyltransferase 1 OS=Homo sapiens GN=ST6GAL1 PE=1 SV=1 - [SIAT1_HUMAN]	0,65	0,00
Q96AH8	Ras-related protein Rab-7b OS=Homo sapiens GN=RAB7B PE=2 SV=1 - [RAB7B_HUMAN]	0,59	0,00
Q9Y4D1-2	Isoform 2 of Disheveled-associated activator of morphogenesis 1 OS=Homo sapiens GN=DAAM1 - [DAAM1_HUMAN]	0,70	0,00
O94832	Myosin-Id OS=Homo sapiens GN=MYO1D PE=1 SV=2 - [MYO1D_HUMAN]	0,67	0,00
Q86TV6	Tetratricopeptide repeat protein 7B OS=Homo sapiens GN=TTC7B PE=1 SV=3 - [TTC7B_HUMAN]	0,50	0,00
Q9ULX9	Transcription factor MafF OS=Homo sapiens GN=MAFF PE=1 SV=2 - [MAFF_HUMAN]	0,70	0,37
Q96C34-2	Isoform 2 of RUN domain-containing protein 1 OS=Homo sapiens GN=RUNDC1 - [RUND1_HUMAN]	0,75	0,37
P13612	Integrin alpha-4 OS=Homo sapiens GN=ITGA4 PE=1 SV=3 - [ITA4_HUMAN]	0,75	0,37
P80404	4-aminobutyrate aminotransferase, mitochondrial OS=Homo sapiens GN=ABAT PE=1 SV=3 - [GABT_HUMAN]	0,68	0,37
Q6UX07-2	Isoform 2 of Dehydrogenase/reductase SDR family member 13 OS=Homo sapiens GN=DHRS13 - [DHR13_HUMAN]	0,70	0,37

Q14331	Protein FRG1 OS=Homo sapiens GN=FRG1 PE=1 SV=1 - [FRG1_HUMAN]	0,77	0,37
P20265-3	Isoform N-OCT 5B of POU domain, class 3, transcription factor 2 OS=Homo sapiens GN=POU3F2 - [PO3F2_HUMAN]	0,67	0,37
Q9H079	Uncharacterized protein C15orf29 OS=Homo sapiens GN=C15orf29 PE=2 SV=1 - [CO029_HUMAN]	0,73	0,37
Q86T65	Disheveled-associated activator of morphogenesis 2 OS=Homo sapiens GN=DAAM2 PE=2 SV=3 - [DAAM2_HUMAN]	0,67	0,37
O75600	2-amino-3-ketobutyrate coenzyme A ligase, mitochondrial OS=Homo sapiens GN=GCAT PE=2 SV=1 - [KBL_HUMAN]	0,70	0,37
Q14699	Raftlin OS=Homo sapiens GN=RFTN1 PE=1 SV=4 - [RFTN1_HUMAN]	0,70	0,37
O96020	G1/S-specific cyclin-E2 OS=Homo sapiens GN=CCNE2 PE=1 SV=1 - [CCNE2_HUMAN]	0,74	0,37
Q86XN7	Proline and serine-rich protein 1 OS=Homo sapiens GN=PROSER1 PE=1 SV=2 - [PRSR1_HUMAN]	0,75	0,37
Q5JWF2	Guanine nucleotide-binding protein G(s) subunit alpha isoforms XLas OS=Homo sapiens GN=GNAS PE=1 SV=2 - [GNAS1_HU	0,73	0,37
P51587	Breast cancer type 2 susceptibility protein OS=Homo sapiens GN=BRCA2 PE=1 SV=2 - [BRCA2_HUMAN]	0,76	0,37
Q08174-2	Isoform 2 of Protocadherin-1 OS=Homo sapiens GN=PCDH1 - [PCDH1_HUMAN]	0,67	0,37
P08294	Extracellular superoxide dismutase [Cu-Zn] OS=Homo sapiens GN=SOD3 PE=1 SV=2 - [SODE_HUMAN]	0,67	0,37
Q08431-3	Isoform 3 of Lactadherin OS=Homo sapiens GN=MFGE8 - [MFGM_HUMAN]	0,66	0,37

A375 vs. A375VR4

Gene ID	Gene Name	Fold Change	q-value(%)
Q9Y446	Plakophilin-3 OS=Homo sapiens GN=PKP3 PE=1 SV=1 - [PKP3_HUMAN]	45,51	0,00
O60732	Melanoma-associated antigen C1 OS=Homo sapiens GN=MAGEC1 PE=1 SV=3 - [MAGC1_HUMAN]	1283,76	0,00
P20337	Ras-related protein Rab-3B OS=Homo sapiens GN=RAB3B PE=1 SV=2 - [RAB3B_HUMAN]	6,97	0,00
Q99959-2	Isoform 1 of Plakophilin-2 OS=Homo sapiens GN=PKP2 - [PKP2_HUMAN]	10,32	0,00
Q9P2K5-2	Isoform 2 of Myelin expression factor 2 OS=Homo sapiens GN=MYEF2 - [MYEF2_HUMAN]	7,94	0,00
Q01543-2	Isoform 2 of Friend leukemia integration 1 transcription factor OS=Homo sapiens GN=FLI1 - [FLI1_HUMAN]	6,04	0,00
Q96Q89-4	Isoform 4 of Kinesin-like protein KIF20B OS=Homo sapiens GN=KIF20B - [KI20B_HUMAN]	18,14	0,00
Q6KB66-2	Isoform 2 of Keratin, type II cytoskeletal 80 OS=Homo sapiens GN=KRT80 - [K2C80_HUMAN]	12,54	0,00
O75525-2	Isoform 2 of KH domain-containing, RNA-binding, signal transduction-associated protein 3 OS=Homo sapiens GN=KHI	15,96	0,00
P16144-4	Isoform Beta-4D of Integrin beta-4 OS=Homo sapiens GN=ITGB4 - [ITB4_HUMAN]	57323,07	0,00
P05787	Keratin, type II cytoskeletal 8 OS=Homo sapiens GN=KRT8 PE=1 SV=7 - [K2C8_HUMAN]	192,03	0,00
Q9H6F2	Trimeric intracellular cation channel type A OS=Homo sapiens GN=TMEM38A PE=1 SV=1 - [TM38A_HUMAN]	4,81	0,00
Q99608	Necdin OS=Homo sapiens GN=NDN PE=2 SV=1 - [NECD_HUMAN]	6,78	0,00
Q16270	Insulin-like growth factor-binding protein 7 OS=Homo sapiens GN=IGFBP7 PE=1 SV=1 - [IBP7_HUMAN]	5,89	0,00
Q8IWR1	Tripartite motif-containing protein 59 OS=Homo sapiens GN=TRIM59 PE=2 SV=1 - [TRI59_HUMAN]	3,50	0,00
Q6ZUT6	Uncharacterized protein C15orf52 OS=Homo sapiens GN=C15orf52 PE=1 SV=1 - [CO052_HUMAN]	2,05	0,00
Q9Y2T3	Guanine deaminase OS=Homo sapiens GN=GDA PE=1 SV=1 - [GUAD_HUMAN]	907,34	0,00
Q9BRK0	Receptor expression-enhancing protein 2 OS=Homo sapiens GN=REEP2 PE=2 SV=2 - [REEP2_HUMAN]	3,08	0,00
P13284	Gamma-interferon-inducible lysosomal thiol reductase OS=Homo sapiens GN=IFI30 PE=1 SV=3 - [GILT_HUMAN]	2,67	0,00
P20138	Myeloid cell surface antigen CD33 OS=Homo sapiens GN=CD33 PE=1 SV=2 - [CD33_HUMAN]	23,91	0,00
Q92506	Estradiol 17-beta-dehydrogenase 8 OS=Homo sapiens GN=HSD17B8 PE=1 SV=2 - [DHB8_HUMAN]	2,87	0,00
O15479	Melanoma-associated antigen B2 OS=Homo sapiens GN=MAGEB2 PE=1 SV=3 - [MAGB2_HUMAN]	2,96	0,00
P56817-4	Isoform D of Beta-secretase 1 OS=Homo sapiens GN=BACE1 - [BACE1_HUMAN]	5,93	0,00
P80723	Brain acid soluble protein 1 OS=Homo sapiens GN=BASP1 PE=1 SV=2 - [BASP1_HUMAN]	31,11	0,00
Q9NUL3-3	Isoform 3 of Double-stranded RNA-binding protein Staufen homolog 2 OS=Homo sapiens GN=STAU2 - [STAU2_HUMA	1,85	0,00
P32189-1	Isoform 1 of Glycerol kinase OS=Homo sapiens GN=GK - [GLPK_HUMAN]	5,43	0,00
P56199	Integrin alpha-1 OS=Homo sapiens GN=ITGA1 PE=1 SV=2 - [ITA1_HUMAN]	2,57	0,00
Q9NS25	Sperm protein associated with the nucleus on the X chromosome B/F OS=Homo sapiens GN=SPANXB1 PE=2 SV=1 - [S	98,67	0,00
Q9UKU6	Thyrotropin-releasing hormone-degrading ectoenzyme OS=Homo sapiens GN=TRHDE PE=2 SV=1 - [TRHDE_HUMAN]	813,03	0,00
P26022	Pentraxin-related protein PTX3 OS=Homo sapiens GN=PTX3 PE=1 SV=3 - [PTX3_HUMAN]	9,13	0,00
P29317	Ephrin type-A receptor 2 OS=Homo sapiens GN=EPHA2 PE=1 SV=2 - [EPHA2_HUMAN]	16,50	0,00
O94851-2	Isoform 2 of Protein MICAL-2 OS=Homo sapiens GN=MICAL2 - [MICA2_HUMAN]	7,13	0,00

A375 vs. A375VR4

Q92466-2	Isoform D1 of DNA damage-binding protein 2 OS=Homo sapiens GN=DDB2 - [DDB2_HUMAN]	4,40	0,00
Q9H4G0-4	Isoform 4 of Band 4,1-like protein 1 OS=Homo sapiens GN=EPB41L1 - [E41L1_HUMAN]	5,06	0,00
P82970	High mobility group nucleosome-binding domain-containing protein 5 OS=Homo sapiens GN=HMGN5 PE=1 SV=1 - [H	32,20	0,00
Q9UHW9-6	Isoform 6 of Solute carrier family 12 member 6 OS=Homo sapiens GN=SLC12A6 - [S12A6_HUMAN]	5,01	0,00
P10620	Microsomal glutathione S-transferase 1 OS=Homo sapiens GN=MGST1 PE=1 SV=1 - [MGST1_HUMAN]	27392,53	0,00
Q9UJY1	Heat shock protein beta-8 OS=Homo sapiens GN=HSPB8 PE=1 SV=1 - [HSPB8_HUMAN]	1,75	0,00
Q8WUR7	UPF0235 protein C15orf40 OS=Homo sapiens GN=C15orf40 PE=1 SV=1 - [CO040_HUMAN]	1,91	0,00
Q7Z7L1	Schlafen family member 11 OS=Homo sapiens GN=SLFN11 PE=1 SV=2 - [SLN11_HUMAN]	6,08	0,00
P43155-2	Isoform 2 of Carnitine O-acetyltransferase OS=Homo sapiens GN=CRAT - [CACP_HUMAN]	2,32	0,00
Q17RY6	Lymphocyte antigen 6K OS=Homo sapiens GN=LY6K PE=1 SV=2 - [LY6K_HUMAN]	19,49	0,00
P40200-2	Isoform 2 of T-cell surface protein tactile OS=Homo sapiens GN=CD96 - [TACT_HUMAN]	5,46	0,00
Q9HBH0	Rho-related GTP-binding protein RhoF OS=Homo sapiens GN=RHOF PE=2 SV=1 - [RHOF_HUMAN]	245,32	0,00
Q9BV40	Vesicle-associated membrane protein 8 OS=Homo sapiens GN=VAMP8 PE=1 SV=1 - [VAMP8_HUMAN]	16,97	0,00
Q9BT78	COP9 signalosome complex subunit 4 OS=Homo sapiens GN=COPS4 PE=1 SV=1 - [CSN4_HUMAN]	1,91	0,00
O00220	Tumor necrosis factor receptor superfamily member 10A OS=Homo sapiens GN=TNFRSF10A PE=1 SV=3 - [TR10A_HU	12,26	0,00
Q9HAU0-5	Isoform 5 of Pleckstrin homology domain-containing family A member 5 OS=Homo sapiens GN=PLEKHA5 - [PKHA5_H	2,99	0,00
Q86WV6	Transmembrane protein 173 OS=Homo sapiens GN=TMEM173 PE=1 SV=1 - [TM173_HUMAN]	3,35	0,00
Q6ZMK1-2	Isoform 2 of Cysteine and histidine-rich protein 1 OS=Homo sapiens GN=CYHR1 - [CYHR1_HUMAN]	2,13	0,00
Q9H0R8	Gamma-aminobutyric acid receptor-associated protein-like 1 OS=Homo sapiens GN=GABARAPL1 PE=1 SV=1 - [GBRL1	2,19	0,00
P20592	Interferon-induced GTP-binding protein Mx2 OS=Homo sapiens GN=MX2 PE=1 SV=1 - [MX2_HUMAN]	1,67	0,00
P43003	Excitatory amino acid transporter 1 OS=Homo sapiens GN=SLC1A3 PE=1 SV=1 - [EAA1_HUMAN]	12,45	0,00
Q9UN86-2	Isoform B of Ras GTPase-activating protein-binding protein 2 OS=Homo sapiens GN=G3BP2 - [G3BP2_HUMAN]	2,03	0,00
P07196	Neurofilament light polypeptide OS=Homo sapiens GN=NEFL PE=1 SV=3 - [NFL_HUMAN]	24,47	0,00
P43353-2	Isoform 2 of Aldehyde dehydrogenase family 3 member B1 OS=Homo sapiens GN=ALDH3B1 - [AL3B1_HUMAN]	3,93	0,00
O75369-6	Isoform 6 of Filamin-B OS=Homo sapiens GN=FLNB - [FLNB_HUMAN]	6,72	0,00
P31321	cAMP-dependent protein kinase type I-beta regulatory subunit OS=Homo sapiens GN=PRKAR1B PE=1 SV=4 - [KAP1_H	2,75	0,00
Q53EU6	Glycerol-3-phosphate acyltransferase 3 OS=Homo sapiens GN=AGPAT9 PE=1 SV=2 - [GPAT3_HUMAN]	3,73	0,00
O60437	Periplakin OS=Homo sapiens GN=PPL PE=1 SV=4 - [PEPL_HUMAN]	10,25	0,00
Q9NY43	BarH-like 2 homeobox protein OS=Homo sapiens GN=BARHL2 PE=2 SV=2 - [BARH2_HUMAN]	1,60	0,00
Q9H6S3	Epidermal growth factor receptor kinase substrate 8-like protein 2 OS=Homo sapiens GN=EPS8L2 PE=1 SV=2 - [ES8L2	10,70	0,00
Q9BW91-2	Isoform 2 of ADP-ribose pyrophosphatase, mitochondrial OS=Homo sapiens GN=NUDT9 - [NUDT9_HUMAN]	2,15	0,00
O75051	Plexin-A2 OS=Homo sapiens GN=PLXNA2 PE=1 SV=4 - [PLXA2_HUMAN]	2,14	0,00
P25445-6	Isoform 6 of Tumor necrosis factor receptor superfamily member 6 OS=Homo sapiens GN=FAS - [TNR6_HUMAN]	2,27	0,00

A375 vs. A375VR4

P50222	Homeobox protein MOX-2 OS=Homo sapiens GN=MEOX2 PE=1 SV=2 - [MEOX2_HUMAN]	2,85	0,00
P32970	CD70 antigen OS=Homo sapiens GN=CD70 PE=1 SV=2 - [CD70_HUMAN]	3,03	0,00
Q86SJ2	Amphoterin-induced protein 2 OS=Homo sapiens GN=AMIGO2 PE=1 SV=1 - [AMGO2_HUMAN]	116,39	0,00
Q8IWS0	PHD finger protein 6 OS=Homo sapiens GN=PHF6 PE=1 SV=1 - [PHF6_HUMAN]	1,62	0,00
Q9NX02-3	Isoform 3 of NACHT, LRR and PYD domains-containing protein 2 OS=Homo sapiens GN=NLRP2 - [NALP2_HUMAN]	160518194,63	0,00
P29034	Protein S100-A2 OS=Homo sapiens GN=S100A2 PE=1 SV=3 - [S10A2_HUMAN]	5,23	0,00
Q8IZV5	Retinol dehydrogenase 10 OS=Homo sapiens GN=RDH10 PE=1 SV=1 - [RDH10_HUMAN]	2,14	0,00
Q02930-4	Isoform 4 of Cyclic AMP-responsive element-binding protein 5 OS=Homo sapiens GN=CREB5 - [CREB5_HUMAN]	2,24	0,00
Q9UPQ8	Dolichol kinase OS=Homo sapiens GN=DOLK PE=1 SV=1 - [DOLK_HUMAN]	2,61	0,00
Q13813	Spectrin alpha chain, brain OS=Homo sapiens GN=SPTAN1 PE=1 SV=3 - [SPTA2_HUMAN]	2,04	0,00
Q13887	Krueppel-like factor 5 OS=Homo sapiens GN=KLF5 PE=1 SV=2 - [KLF5_HUMAN]	4,60	0,00
P15328	Folate receptor alpha OS=Homo sapiens GN=FOLR1 PE=1 SV=3 - [FOLR1_HUMAN]	19,34	0,00
Q15526-2	Isoform 2 of Surfeit locus protein 1 OS=Homo sapiens GN=SURF1 - [SURF1_HUMAN]	2,58	0,00
P15144	Aminopeptidase N OS=Homo sapiens GN=ANPEP PE=1 SV=4 - [AMPN_HUMAN]	50,31	0,00
Q96CF2	Charged multivesicular body protein 4c OS=Homo sapiens GN=CHMP4C PE=1 SV=1 - [CHM4C_HUMAN]	8,89	0,00
O00425	Insulin-like growth factor 2 mRNA-binding protein 3 OS=Homo sapiens GN=IGF2BP3 PE=1 SV=2 - [IF2B3_HUMAN]	1,76	0,00
Q6P9B6	TLD domain-containing protein KIAA1609 OS=Homo sapiens GN=KIAA1609 PE=1 SV=2 - [K1609_HUMAN]	1,86	0,00
Q0ZGT2-2	Isoform 2 of Nexilin OS=Homo sapiens GN=NEXN - [NEXN_HUMAN]	4,46	0,00
Q9UBW8	COP9 signalosome complex subunit 7a OS=Homo sapiens GN=COPS7A PE=1 SV=1 - [CSN7A_HUMAN]	1,80	0,00
Q5T5S1	Uncharacterized coiled-coil domain-containing protein KIAA1984 OS=Homo sapiens GN=KIAA1984 PE=2 SV=3 - [K198	899,15	0,00
P35612	Beta-adducin OS=Homo sapiens GN=ADD2 PE=1 SV=3 - [ADDB_HUMAN]	4,24	0,00
Q8N3D4	EH domain-binding protein 1-like protein 1 OS=Homo sapiens GN=EHBP1L1 PE=1 SV=2 - [EH1L1_HUMAN]	1,77	0,00
P07203	Glutathione peroxidase 1 OS=Homo sapiens GN=GPX1 PE=1 SV=4 - [GPX1_HUMAN]	1,69	0,00
Q9UQ49	Sialidase-3 OS=Homo sapiens GN=NEU3 PE=1 SV=1 - [NEUR3_HUMAN]	3,69	0,00
Q6UWZ7	BRCA1-A complex subunit Abraxas OS=Homo sapiens GN=FAM175A PE=1 SV=2 - [F175A_HUMAN]	3,16	0,00
Q9NZV1	Cysteine-rich motor neuron 1 protein OS=Homo sapiens GN=CRIM1 PE=1 SV=1 - [CRIM1_HUMAN]	1,64	0,00
P52926	High mobility group protein HMGI-C OS=Homo sapiens GN=HMGA2 PE=1 SV=1 - [HMGA2_HUMAN]	1,68	0,00
Q9NZI8	Insulin-like growth factor 2 mRNA-binding protein 1 OS=Homo sapiens GN=IGF2BP1 PE=1 SV=2 - [IF2B1_HUMAN]	1,63	0,00
P10301	Ras-related protein R-Ras OS=Homo sapiens GN=RRAS PE=1 SV=1 - [RRAS_HUMAN]	3,02	0,00
Q15599	Na(+)/H(+) exchange regulatory cofactor NHE-RF2 OS=Homo sapiens GN=SLC9A3R2 PE=1 SV=2 - [NHRF2_HUMAN]	1,99	0,00
Q9UNW1	Multiple inositol polyphosphate phosphatase 1 OS=Homo sapiens GN=MINPP1 PE=1 SV=1 - [MINP1_HUMAN]	2,47	0,00
Q8N3K9	Cardiomyopathy-associated protein 5 OS=Homo sapiens GN=CMYA5 PE=1 SV=3 - [CMYA5_HUMAN]	1,66	0,00
Q9NVH6-2	Isoform B of Trimethyllysine dioxygenase, mitochondrial OS=Homo sapiens GN=TMLHE - [TMLH_HUMAN]	1,97	0,00

A375 vs. A375VR4

O60427	Fatty acid desaturase 1 OS=Homo sapiens GN=FADS1 PE=1 SV=1 - [FADS1_HUMAN]	4,43	0,00
O15020-2	Isoform 2 of Spectrin beta chain, brain 2 OS=Homo sapiens GN=SPTBN2 - [SPTN2_HUMAN]	2,02	0,00
P08581	Hepatocyte growth factor receptor OS=Homo sapiens GN=MET PE=1 SV=4 - [MET_HUMAN]	2,59	0,00
Q8NBJ7-2	Isoform 2 of Sulfatase-modifying factor 2 OS=Homo sapiens GN=SUMF2 - [SUMF2_HUMAN]	1,86	0,00
Q13740-2	Isoform 2 of CD166 antigen OS=Homo sapiens GN=ALCAM - [CD166_HUMAN]	2,60	0,00
Q8N3E9	1-phosphatidylinositol-4,5-bisphosphate phosphodiesterase delta-3 OS=Homo sapiens GN=PLCD3 PE=1 SV=3 - [PLCD	2,58	0,00
Q96C36	Pyrroline-5-carboxylate reductase 2 OS=Homo sapiens GN=PYCR2 PE=1 SV=1 - [P5CR2_HUMAN]	1,70	0,00
P26885	Peptidyl-prolyl cis-trans isomerase FKBP2 OS=Homo sapiens GN=FKBP2 PE=1 SV=2 - [FKBP2_HUMAN]	2,27	0,00
Q9H936	Mitochondrial glutamate carrier 1 OS=Homo sapiens GN=SLC25A22 PE=1 SV=1 - [GHC1_HUMAN]	3,35	0,00
Q92985-3	Isoform C of Interferon regulatory factor 7 OS=Homo sapiens GN=IRF7 - [IRF7_HUMAN]	1,74	0,00
Q86UN3	Reticulon-4 receptor-like 2 OS=Homo sapiens GN=RTN4RL2 PE=1 SV=1 - [R4RL2_HUMAN]	2,63	0,00
Q14697-2	Isoform 2 of Neutral alpha-glucosidase AB OS=Homo sapiens GN=GANAB - [GANAB_HUMAN]	1,64	0,00
P13987	CD59 glycoprotein OS=Homo sapiens GN=CD59 PE=1 SV=1 - [CD59_HUMAN]	2,49	0,00
Q15434	RNA-binding motif, single-stranded-interacting protein 2 OS=Homo sapiens GN=RBMS2 PE=1 SV=1 - [RBMS2_HUMAN	2,01	0,00
Q8NEW0	Zinc transporter 7 OS=Homo sapiens GN=SLC30A7 PE=1 SV=1 - [ZNT7_HUMAN]	1,71	0,00
Q13620-1	Isoform 2 of Cullin-4B OS=Homo sapiens GN=CUL4B - [CUL4B_HUMAN]	1,57	0,00
P30530-2	Isoform Short of Tyrosine-protein kinase receptor UFO OS=Homo sapiens GN=AXL - [UFO_HUMAN]	1,59	0,00
P14923	Junction plakoglobin OS=Homo sapiens GN=JUP PE=1 SV=3 - [PLAK_HUMAN]	1,66	0,00
Q99653	Calcium-binding protein p22 OS=Homo sapiens GN=CHP PE=1 SV=3 - [CHP1_HUMAN]	2,22	0,00
P62070	Ras-related protein R-Ras2 OS=Homo sapiens GN=RRAS2 PE=1 SV=1 - [RRAS2_HUMAN]	1,80	0,00
O14657	Torsin-1B OS=Homo sapiens GN=TOR1B PE=1 SV=2 - [TOR1B_HUMAN]	5,92	0,00
Q13015	Protein AF1q OS=Homo sapiens GN=MLLT11 PE=2 SV=1 - [AF1Q_HUMAN]	2,15	0,00
P14210-6	Isoform 6 of Hepatocyte growth factor OS=Homo sapiens GN=HGF - [HGF_HUMAN]	3,15	0,00
A0AV96-2	Isoform 2 of RNA-binding protein 47 OS=Homo sapiens GN=RBM47 - [RBM47_HUMAN]	1,61	0,00
Q96NZ8	WAP, kazal, immunoglobulin, kunitz and NTR domain-containing protein 1 OS=Homo sapiens GN=WFIKKN1 PE=1 SV=	2,40	0,00
Q9BY67	Cell adhesion molecule 1 OS=Homo sapiens GN=CADM1 PE=1 SV=2 - [CADM1_HUMAN]	2,33	0,00
Q9Y4K1	Absent in melanoma 1 protein OS=Homo sapiens GN=AIM1 PE=1 SV=3 - [AIM1_HUMAN]	1,78	0,00
P19532	Transcription factor E3 OS=Homo sapiens GN=TFE3 PE=1 SV=4 - [TFE3_HUMAN]	1,46	0,00
Q01955	Collagen alpha-3(IV) chain OS=Homo sapiens GN=COL4A3 PE=1 SV=3 - [CO4A3_HUMAN]	1,76	0,00
O95816	BAG family molecular chaperone regulator 2 OS=Homo sapiens GN=BAG2 PE=1 SV=1 - [BAG2_HUMAN]	1,70	0,00
Q6JQN1	Acyl-CoA dehydrogenase family member 10 OS=Homo sapiens GN=ACAD10 PE=2 SV=1 - [ACD10_HUMAN]	1,67	0,00
O95466	Formin-like protein 1 OS=Homo sapiens GN=FMNL1 PE=1 SV=3 - [FMNL_HUMAN]	1,76	0,00
Q92968	Peroxisomal membrane protein PEX13 OS=Homo sapiens GN=PEX13 PE=1 SV=2 - [PEX13_HUMAN]	1,49	0,00

A375 vs. A375VR4

Q12959-4	Isoform 4 of Disks large homolog 1 OS=Homo sapiens GN=DLG1 - [DLG1_HUMAN]	1,91	0,00
Q9ULS5	Transmembrane and coiled-coil domains protein 3 OS=Homo sapiens GN=TMCC3 PE=2 SV=3 - [TMCC3_HUMAN]	2,21	0,00
O14639	Actin-binding LIM protein 1 OS=Homo sapiens GN=ABLIM1 PE=1 SV=3 - [ABLM1_HUMAN]	2,02	0,00
P84095	Rho-related GTP-binding protein RhoG OS=Homo sapiens GN=RHOG PE=1 SV=1 - [RHOG_HUMAN]	2,26	0,00
Q14160	Protein scribble homolog OS=Homo sapiens GN=SCRIB PE=1 SV=4 - [SCRIB_HUMAN]	1,77	0,00
Q13586	Stromal interaction molecule 1 OS=Homo sapiens GN=STIM1 PE=1 SV=3 - [STIM1_HUMAN]	2,48	0,00
Q96B70	Leukocyte receptor cluster member 9 OS=Homo sapiens GN=LENG9 PE=2 SV=2 - [LENG9_HUMAN]	2,28	0,00
P60903	Protein S100-A10 OS=Homo sapiens GN=S100A10 PE=1 SV=2 - [S10AA_HUMAN]	1,58	0,00
P10696	Alkaline phosphatase, placental-like OS=Homo sapiens GN=ALPPL2 PE=1 SV=4 - [PPBN_HUMAN]	207,48	0,00
Q86XX4-5	Isoform 5 of Extracellular matrix protein FRAS1 OS=Homo sapiens GN=FRAS1 - [FRAS1_HUMAN]	2,65	0,00
Q14CZ8-2	Isoform 2 of Hepatocyte cell adhesion molecule OS=Homo sapiens GN=HEPACAM - [HECAM_HUMAN]	1,55	0,00
Q9BUK0	Coiled-coil-helix-coiled-coil-helix domain-containing protein 7 OS=Homo sapiens GN=CHCHD7 PE=2 SV=1 - [CHCH7_H	2,28	0,00
P13645	Keratin, type I cytoskeletal 10 OS=Homo sapiens GN=KRT10 PE=1 SV=6 - [K1C10_HUMAN]	1,50	0,00
P00533	Epidermal growth factor receptor OS=Homo sapiens GN=EGFR PE=1 SV=2 - [EGFR_HUMAN]	2,51	0,00
O14656	Torsin-1A OS=Homo sapiens GN=TOR1A PE=1 SV=1 - [TOR1A_HUMAN]	1,99	0,00
Q9Y3L5	Ras-related protein Rap-2c OS=Homo sapiens GN=RAP2C PE=1 SV=1 - [RAP2C_HUMAN]	2,37	0,00
Q96IF1	LIM domain-containing protein ajuba OS=Homo sapiens GN=AJUBA PE=1 SV=1 - [AJUBA_HUMAN]	2,46	0,00
Q15067	Peroxisomal acyl-coenzyme A oxidase 1 OS=Homo sapiens GN=ACOX1 PE=1 SV=3 - [ACOX1_HUMAN]	2,37	0,00
Q7Z402	Transmembrane channel-like protein 7 OS=Homo sapiens GN=TMC7 PE=2 SV=1 - [TMC7_HUMAN]	2,01	0,00
P07355	Annexin A2 OS=Homo sapiens GN=ANXA2 PE=1 SV=2 - [ANXA2_HUMAN]	1,71	0,00
P79522-2	Isoform 2 of Proline-rich protein 3 OS=Homo sapiens GN=PRR3 - [PRR3_HUMAN]	2,02	0,00
Q9HBH5	Retinol dehydrogenase 14 OS=Homo sapiens GN=RDH14 PE=1 SV=1 - [RDH14_HUMAN]	2,41	0,00
Q96I45	Transmembrane protein 141 OS=Homo sapiens GN=TMEM141 PE=2 SV=1 - [TM141_HUMAN]	2,45	0,00
Q9H3Q1	Cdc42 effector protein 4 OS=Homo sapiens GN=CDC42EP4 PE=1 SV=1 - [BORG4_HUMAN]	1,99	0,00
Q7L8J4	SH3 domain-binding protein 5-like OS=Homo sapiens GN=SH3BP5L PE=1 SV=1 - [3BP5L_HUMAN]	2,41	0,00
Q9UIV1	CCR4-NOT transcription complex subunit 7 OS=Homo sapiens GN=CNOT7 PE=1 SV=3 - [CNOT7_HUMAN]	1,46	0,00
P07339	Cathepsin D OS=Homo sapiens GN=CTSD PE=1 SV=1 - [CATD_HUMAN]	1,52	0,00
Q9UQ03-2	Isoform 2 of Coronin-2B OS=Homo sapiens GN=CORO2B - [COR2B_HUMAN]	1,77	0,00
Q15147-2	Isoform 1 of 1-phosphatidylinositol-4,5-bisphosphate phosphodiesterase beta-4 OS=Homo sapiens GN=PLCB4 - [PLCE	3,43	0,00
P11117	Lysosomal acid phosphatase OS=Homo sapiens GN=ACP2 PE=1 SV=3 - [PPAL_HUMAN]	1,89	0,00
P05161	Ubiquitin-like protein ISG15 OS=Homo sapiens GN=ISG15 PE=1 SV=5 - [ISG15_HUMAN]	1,86	0,00
Q09666	Neuroblast differentiation-associated protein AHNAK OS=Homo sapiens GN=AHNAK PE=1 SV=2 - [AHNK_HUMAN]	1,63	0,00
O94854-2	Isoform 2 of Uncharacterized protein KIAA0754 OS=Homo sapiens GN=KIAA0754 - [K0754_HUMAN]	1,68	0,00

A375 vs. A375VR4

Q9HC16	DNA dC->dU-editing enzyme APOBEC-3G OS=Homo sapiens GN=APOBEC3G PE=1 SV=1 - [ABC3G_HUMAN]	2,74	0,00
Q8N3V7-2	Isoform 2 of Synaptopodin OS=Homo sapiens GN=SYNPO - [SYNPO_HUMAN]	3,35	0,00
Q969G2	LIM/homeobox protein Lhx4 OS=Homo sapiens GN=LHX4 PE=1 SV=2 - [LHX4_HUMAN]	1,45	0,00
O14967	Calmegin OS=Homo sapiens GN=CLGN PE=1 SV=1 - [CLGN_HUMAN]	1,53	0,00
Q99828	Calcium and integrin-binding protein 1 OS=Homo sapiens GN=CIB1 PE=1 SV=4 - [CIB1_HUMAN]	1,52	0,00
Q9GZT6-2	Isoform 2 of Coiled-coil domain-containing protein 90B, mitochondrial OS=Homo sapiens GN=CCDC90B - [CC90B_HU	2,08	0,00
Q6UW68	Transmembrane protein 205 OS=Homo sapiens GN=TMEM205 PE=1 SV=1 - [TM205_HUMAN]	1,53	0,00
P55290	Cadherin-13 OS=Homo sapiens GN=CDH13 PE=1 SV=1 - [CAD13_HUMAN]	1,58	0,00
O00479	High mobility group nucleosome-binding domain-containing protein 4 OS=Homo sapiens GN=HMGN4 PE=1 SV=3 - [H	1,51	0,00
Q92692	Poliovirus receptor-related protein 2 OS=Homo sapiens GN=PVRL2 PE=1 SV=1 - [PVRL2_HUMAN]	2,36	0,00
Q8N4T8-2	Isoform 2 of Carbonyl reductase family member 4 OS=Homo sapiens GN=CBR4 - [CBR4_HUMAN]	1,60	0,00
O60220	Mitochondrial import inner membrane translocase subunit Tim8 A OS=Homo sapiens GN=TIMM8A PE=1 SV=1 - [TIM	2,05	0,00
Q6P4E1-2	Isoform 2 of Protein CASC4 OS=Homo sapiens GN=CASC4 - [CASC4_HUMAN]	2,05	0,00
Q8WWI1-3	Isoform 3 of LIM domain only protein 7 OS=Homo sapiens GN=LMO7 - [LMO7_HUMAN]	2,17	0,00
Q86UT6	NLR family member X1 OS=Homo sapiens GN=NLRX1 PE=1 SV=1 - [NLRX1_HUMAN]	2,48	0,00
Q9Y6X4	Protein FAM169A OS=Homo sapiens GN=FAM169A PE=1 SV=2 - [F169A_HUMAN]	1,83	0,00
P25815	Protein S100-P OS=Homo sapiens GN=S100P PE=1 SV=2 - [S100P_HUMAN]	953,99	0,00
Q86U28	Iron-sulfur cluster assembly 2 homolog, mitochondrial OS=Homo sapiens GN=ISCA2 PE=1 SV=2 - [ISCA2_HUMAN]	1,69	0,00
Q07817-2	Isoform Bcl-X(S) of Bcl-2-like protein 1 OS=Homo sapiens GN=BCL2L1 - [B2CL1_HUMAN]	1,76	0,00
Q15293	Reticulocalbin-1 OS=Homo sapiens GN=RCN1 PE=1 SV=1 - [RCN1_HUMAN]	1,50	0,00
Q96HR9	Receptor expression-enhancing protein 6 OS=Homo sapiens GN=REEP6 PE=1 SV=1 - [REEP6_HUMAN]	1,47	0,00
Q13188	Serine/threonine-protein kinase 3 OS=Homo sapiens GN=STK3 PE=1 SV=2 - [STK3_HUMAN]	1,65	0,00
A5D8V6	Vacuolar protein sorting-associated protein 37C OS=Homo sapiens GN=VPS37C PE=1 SV=2 - [VP37C_HUMAN]	2,16	0,00
Q15149-8	Isoform 8 of Plectin OS=Homo sapiens GN=PLEC - [PLEC_HUMAN]	2,82	0,00
O95772-2	Isoform 2 of MLN64 N-terminal domain homolog OS=Homo sapiens GN=STARD3NL - [MENTO_HUMAN]	3,16	0,00
Q8NHG7	Small VCP/p97-interacting protein OS=Homo sapiens GN=SVIP PE=2 SV=1 - [SVIP_HUMAN]	1,69	0,00
Q5T2D3	OTU domain-containing protein 3 OS=Homo sapiens GN=OTUD3 PE=1 SV=1 - [OTUD3_HUMAN]	1,36	0,00
Q9BSH5	Haloacid dehalogenase-like hydrolase domain-containing protein 3 OS=Homo sapiens GN=HDHD3 PE=1 SV=1 - [HDHD	1,59	0,00
Q8N4A0	Polypeptide N-acetylgalactosaminyltransferase 4 OS=Homo sapiens GN=GALNT4 PE=1 SV=2 - [GALT4_HUMAN]	1,81	0,00
Q96SW2-2	Isoform 2 of Protein cereblon OS=Homo sapiens GN=CRBN - [CRBN_HUMAN]	2,41	0,00
Q15262	Receptor-type tyrosine-protein phosphatase kappa OS=Homo sapiens GN=PTPRK PE=1 SV=2 - [PTPRK_HUMAN]	2,31	0,00
Q9BYD6	39S ribosomal protein L1, mitochondrial OS=Homo sapiens GN=MRPL1 PE=1 SV=2 - [RM01_HUMAN]	1,71	0,00
P30044-2	Isoform Cytoplasmic+peroxisomal of Peroxiredoxin-5, mitochondrial OS=Homo sapiens GN=PRDX5 - [PRDX5_HUMAN	1,41	0,00

A375 vs. A375VR4

Q4VC05	B-cell CLL/lymphoma 7 protein family member A OS=Homo sapiens GN=BCL7A PE=1 SV=1 - [BCL7A_HUMAN]	1,69	0,00
P48431	Transcription factor SOX-2 OS=Homo sapiens GN=SOX2 PE=1 SV=1 - [SOX2_HUMAN]	1,77	0,00
Q8NBM4-4	Isoform 4 of Ubiquitin-associated domain-containing protein 2 OS=Homo sapiens GN=UBAC2 - [UBAC2_HUMAN]	2,05	0,00
Q9HCE1	Putative helicase MOV-10 OS=Homo sapiens GN=MOV10 PE=1 SV=2 - [MOV10_HUMAN]	1,54	0,00
P20908	Collagen alpha-1(V) chain OS=Homo sapiens GN=COL5A1 PE=1 SV=3 - [CO5A1_HUMAN]	13,97	0,00
Q5JRK9	Putative G antigen family E member 3 OS=Homo sapiens GN=PAGE2B PE=2 SV=1 - [GGEE3_HUMAN]	24492356,65	0,00
Q01650	Large neutral amino acids transporter small subunit 1 OS=Homo sapiens GN=SLC7A5 PE=1 SV=2 - [LAT1_HUMAN]	2,85	0,00
Q9UBF1	Melanoma-associated antigen C2 OS=Homo sapiens GN=MAGEC2 PE=1 SV=1 - [MAGC2_HUMAN]	1,54	0,00
Q6SJ93	Protein FAM111B OS=Homo sapiens GN=FAM111B PE=2 SV=1 - [F111B_HUMAN]	1,51	0,00
O14832	Phytanoyl-CoA dioxygenase, peroxisomal OS=Homo sapiens GN=PHYH PE=1 SV=1 - [PAHX_HUMAN]	1,50	0,00
P05783	Keratin, type I cytoskeletal 18 OS=Homo sapiens GN=KRT18 PE=1 SV=2 - [K1C18_HUMAN]	1,62	0,00
Q9NYM9	BET1-like protein OS=Homo sapiens GN=BET1L PE=1 SV=1 - [BET1L_HUMAN]	1,80	0,00
O94766	Galactosylgalactosylxylosylprotein 3-beta-glucuronosyltransferase 3 OS=Homo sapiens GN=B3GAT3 PE=1 SV=2 - [B3(	3,08	0,00
Q13825-2	Isoform 2 of Methylglutaconyl-CoA hydratase, mitochondrial OS=Homo sapiens GN=AUH - [AUHM_HUMAN]	1,48	0,00
Q9UHN1	DNA polymerase subunit gamma-2, mitochondrial OS=Homo sapiens GN=POLG2 PE=1 SV=1 - [DPOG2_HUMAN]	1,65	0,00
P49184	Deoxyribonuclease-1-like 1 OS=Homo sapiens GN=DNASE1L1 PE=1 SV=1 - [DNSL1_HUMAN]	1,53	0,00
P78381-2	Isoform UGT1 of UDP-galactose translocator OS=Homo sapiens GN=SLC35A2 - [S35A2_HUMAN]	1,36	0,00
P22570	NADPH:adrenodoxin oxidoreductase, mitochondrial OS=Homo sapiens GN=FDXR PE=1 SV=3 - [ADRO_HUMAN]	1,95	0,00
Q92478	C-type lectin domain family 2 member B OS=Homo sapiens GN=CLEC2B PE=2 SV=2 - [CLC2B_HUMAN]	3,90	0,00
Q96GN5-2	Isoform 2 of Cell division cycle-associated 7-like protein OS=Homo sapiens GN=CDCA7L - [CDA7L_HUMAN]	1,60	0,00
P04264	Keratin, type II cytoskeletal 1 OS=Homo sapiens GN=KRT1 PE=1 SV=6 - [K2C1_HUMAN]	1,57	0,00
Q99456	Keratin, type I cytoskeletal 12 OS=Homo sapiens GN=KRT12 PE=1 SV=1 - [K1C12_HUMAN]	1,39	0,00
Q08AF3	Schlafen family member 5 OS=Homo sapiens GN=SLFN5 PE=1 SV=1 - [SLFN5_HUMAN]	2,21	0,00
P26006	Integrin alpha-3 OS=Homo sapiens GN=ITGA3 PE=1 SV=5 - [ITA3_HUMAN]	3,08	0,00
Q92581	Sodium/hydrogen exchanger 6 OS=Homo sapiens GN=SLC9A6 PE=1 SV=2 - [SL9A6_HUMAN]	2,00	0,00
Q9H061	Transmembrane protein 126A OS=Homo sapiens GN=TMEM126A PE=1 SV=1 - [T126A_HUMAN]	2,18	0,00
Q7Z3D6-5	Isoform 5 of UPF0317 protein C14orf159, mitochondrial OS=Homo sapiens GN=C14orf159 - [CN159_HUMAN]	1,41	0,00
Q8IVF2-3	Isoform 3 of Protein AHNAK2 OS=Homo sapiens GN=AHNAK2 - [AHNK2_HUMAN]	1,50	0,00
Q8N8U2	Chromodomain Y-like protein 2 OS=Homo sapiens GN=CDYL2 PE=2 SV=2 - [CDYL2_HUMAN]	2,05	0,00
Q9HAB3	Riboflavin transporter 3 OS=Homo sapiens GN=GPR172A PE=1 SV=1 - [RFT3_HUMAN]	1,50	0,00
O43790	Keratin, type II cuticular Hb6 OS=Homo sapiens GN=KRT86 PE=1 SV=1 - [KRT86_HUMAN]	1,97	0,00
P05120	Plasminogen activator inhibitor 2 OS=Homo sapiens GN=SERPINB2 PE=1 SV=2 - [PAI2_HUMAN]	153,05	0,00
Q86UD1	Out at first protein homolog OS=Homo sapiens GN=OAF PE=2 SV=1 - [OAF_HUMAN]	1,80	0,00

A375 vs. A375VR4

P38936	Cyclin-dependent kinase inhibitor 1 OS=Homo sapiens GN=CDKN1A PE=1 SV=3 - [CDN1A_HUMAN]	4,30	0,00
Q52LW3	Rho GTPase-activating protein 29 OS=Homo sapiens GN=ARHGAP29 PE=1 SV=2 - [RHG29_HUMAN]	1,85	0,00
O60831	PRA1 family protein 2 OS=Homo sapiens GN=PRAF2 PE=1 SV=1 - [PRAF2_HUMAN]	1,74	0,00
Q9NTG7	NAD-dependent deacetylase sirtuin-3, mitochondrial OS=Homo sapiens GN=SIRT3 PE=1 SV=2 - [SIRT3_HUMAN]	1,53	0,00
Q15149-4	Isoform 4 of Plectin OS=Homo sapiens GN=PLEC - [PLEC_HUMAN]	2,78	0,00
Q96DA6	Mitochondrial import inner membrane translocase subunit TIM14 OS=Homo sapiens GN=DNAJC19 PE=1 SV=3 - [TIM1	1,65	0,00
Q04727	Transducin-like enhancer protein 4 OS=Homo sapiens GN=TLE4 PE=1 SV=3 - [TLE4_HUMAN]	1,68	0,00
O60763	General vesicular transport factor p115 OS=Homo sapiens GN=USO1 PE=1 SV=2 - [USO1_HUMAN]	1,95	0,00
Q9NYJ1	Coiled-coil-helix-coiled-coil-helix domain-containing protein 8 OS=Homo sapiens GN=CHCHD8 PE=1 SV=2 - [CHCH8_H	2,10	0,00
Q96K19-5	Isoform 5 of RING finger protein 170 OS=Homo sapiens GN=RNF170 - [RN170_HUMAN]	2,99	0,00
Q96RT1-7	Isoform 7 of Protein LAP2 OS=Homo sapiens GN=ERBB2IP - [LAP2_HUMAN]	1,50	0,00
P52758	Ribonuclease UK114 OS=Homo sapiens GN=HRSP12 PE=1 SV=1 - [UK114_HUMAN]	1,39	0,00
Q8TCZ2-3	Isoform 3 of CD99 antigen-like protein 2 OS=Homo sapiens GN=CD99L2 - [C99L2_HUMAN]	1,41	0,00
Q8IY31	Intraflagellar transport protein 20 homolog OS=Homo sapiens GN=IFT20 PE=1 SV=1 - [IFT20_HUMAN]	1,33	0,00
Q16531	DNA damage-binding protein 1 OS=Homo sapiens GN=DDB1 PE=1 SV=1 - [DDB1_HUMAN]	1,47	0,00
Q2TAM9	Tumor suppressor candidate gene 1 protein OS=Homo sapiens GN=TUSC1 PE=1 SV=3 - [TUSC1_HUMAN]	1,69	0,00
Q15582	Transforming growth factor-beta-induced protein ig-h3 OS=Homo sapiens GN=TGFBI PE=1 SV=1 - [BGH3_HUMAN]	2,64	0,00
Q9NW68-2	Isoform 2 of BSD domain-containing protein 1 OS=Homo sapiens GN=BSDC1 - [BSDC1_HUMAN]	1,34	0,00
Q9NZ45	CDGSH iron-sulfur domain-containing protein 1 OS=Homo sapiens GN=CISD1 PE=1 SV=1 - [CISD1_HUMAN]	1,63	0,00
Q15746-4	Isoform 3B of Myosin light chain kinase, smooth muscle OS=Homo sapiens GN=MYLK - [MYLK_HUMAN]	1,75	0,00
P51114-2	Isoform 2 of Fragile X mental retardation syndrome-related protein 1 OS=Homo sapiens GN=FXR1 - [FXR1_HUMAN]	1,31	0,00
O60716-5	Isoform 1A of Catenin delta-1 OS=Homo sapiens GN=CTNND1 - [CTND1_HUMAN]	1,38	0,00
O15455	Toll-like receptor 3 OS=Homo sapiens GN=TLR3 PE=1 SV=1 - [TLR3_HUMAN]	1,67	0,00
Q8TEM1	Nuclear pore membrane glycoprotein 210 OS=Homo sapiens GN=NUP210 PE=1 SV=3 - [PO210_HUMAN]	2,61	0,00
Q92905	COP9 signalosome complex subunit 5 OS=Homo sapiens GN=COPS5 PE=1 SV=4 - [CSN5_HUMAN]	1,87	0,00
Q9HCP0-2	Isoform 1S of Casein kinase I isoform gamma-1 OS=Homo sapiens GN=CSNK1G1 - [KC1G1_HUMAN]	1,68	0,00
Q53HC5	Kelch-like protein 26 OS=Homo sapiens GN=KLHL26 PE=1 SV=2 - [KLH26_HUMAN]	1,49	0,00
Q9NZC3	Glycerophosphodiester phosphodiesterase 1 OS=Homo sapiens GN=GDE1 PE=1 SV=1 - [GDE1_HUMAN]	2,72	0,00
P35914	Hydroxymethylglutaryl-CoA lyase, mitochondrial OS=Homo sapiens GN=HMGCL PE=1 SV=2 - [HMGCL_HUMAN]	1,51	0,00
P98175-4	Isoform 4 of RNA-binding protein 10 OS=Homo sapiens GN=RBM10 - [RBM10_HUMAN]	2,07	0,00
Q8N5D0-4	Isoform 4 of WD and tetratricopeptide repeats protein 1 OS=Homo sapiens GN=WDTC1 - [WDTC1_HUMAN]	1,44	0,00
Q96LJ7	Dehydrogenase/reductase SDR family member 1 OS=Homo sapiens GN=DHRS1 PE=1 SV=1 - [DHRS1_HUMAN]	1,93	0,00
Q08554-2	Isoform 1B of Desmocollin-1 OS=Homo sapiens GN=DSC1 - [DSC1_HUMAN]	1,63	0,00

A375 vs. A375VR4

Q71RG4-3	Isoform 3 of Transmembrane and ubiquitin-like domain-containing protein 2 OS=Homo sapiens GN=TMUB2 - [TMUB2	2,11	0,00
Q8NG06	Tripartite motif-containing protein 58 OS=Homo sapiens GN=TRIM58 PE=2 SV=2 - [TRI58_HUMAN]	1,46	0,00
P49748-2	Isoform 2 of Very long-chain specific acyl-CoA dehydrogenase, mitochondrial OS=Homo sapiens GN=ACADVL - [ACAD	1,49	0,00
Q15834	Coiled-coil domain-containing protein 85B OS=Homo sapiens GN=CCDC85B PE=1 SV=2 - [CC85B_HUMAN]	1,60	0,00
O95167	NADH dehydrogenase [ubiquinone] 1 alpha subcomplex subunit 3 OS=Homo sapiens GN=NDUFA3 PE=1 SV=1 - [NDUA	1,86	0,00
P98160	Basement membrane-specific heparan sulfate proteoglycan core protein OS=Homo sapiens GN=HSPG2 PE=1 SV=4 - [	1,50	0,00
Q9Y297-2	Isoform 2 of F-box/WD repeat-containing protein 1A OS=Homo sapiens GN=BTRC - [FBW1A_HUMAN]	1,77	0,00
Q9NQS3	Poliovirus receptor-related protein 3 OS=Homo sapiens GN=PVRL3 PE=1 SV=1 - [PVRL3_HUMAN]	3,01	0,00
Q96FQ6	Protein S100-A16 OS=Homo sapiens GN=S100A16 PE=1 SV=1 - [S10AG_HUMAN]	2,07	0,00
A8K2U0	Alpha-2-macroglobulin-like protein 1 OS=Homo sapiens GN=A2ML1 PE=1 SV=3 - [A2ML1_HUMAN]	4,73	0,00
Q15697-2	Isoform 2 of Zinc finger protein 174 OS=Homo sapiens GN=ZNF174 - [ZN174_HUMAN]	1,44	0,00
Q8WUK0-2	Isoform 2 of Protein-tyrosine phosphatase mitochondrial 1 OS=Homo sapiens GN=PTPMT1 - [PTPM1_HUMAN]	1,98	0,00
P51828	Adenylate cyclase type 7 OS=Homo sapiens GN=ADCY7 PE=2 SV=1 - [ADCY7_HUMAN]	2,11	0,00
P42773	Cyclin-dependent kinase 4 inhibitor C OS=Homo sapiens GN=CDKN2C PE=1 SV=1 - [CDN2C_HUMAN]	1,49	0,00
Q7Z449	Cytochrome P450 2U1 OS=Homo sapiens GN=CYP2U1 PE=1 SV=1 - [CP2U1_HUMAN]	1,96	0,00
P08195	4F2 cell-surface antigen heavy chain OS=Homo sapiens GN=SLC3A2 PE=1 SV=3 - [4F2_HUMAN]	2,67	0,00
Q8IUX1-4	Isoform 4 of Transmembrane protein 126B OS=Homo sapiens GN=TMEM126B - [T126B_HUMAN]	1,66	0,00
O00468	Agrin OS=Homo sapiens GN=AGRN PE=1 SV=4 - [AGRIN_HUMAN]	1,68	0,00
Q03405-2	Isoform 2 of Urokinase plasminogen activator surface receptor OS=Homo sapiens GN=PLAUR - [UPAR_HUMAN]	1,68	0,00
Q9C0B5-2	Isoform 2 of Palmitoyltransferase ZDHHC5 OS=Homo sapiens GN=ZDHHC5 - [ZDHC5_HUMAN]	1,71	0,00
Q15149-5	Isoform 5 of Plectin OS=Homo sapiens GN=PLEC - [PLEC_HUMAN]	1,96	0,00
Q5TAQ9-2	Isoform 2 of DDB1- and CUL4-associated factor 8 OS=Homo sapiens GN=DCAF8 - [DCAF8_HUMAN]	1,64	0,00
P02538	Keratin, type II cytoskeletal 6A OS=Homo sapiens GN=KRT6A PE=1 SV=3 - [K2C6A_HUMAN]	2,77	0,00
Q7L5N1	COP9 signalosome complex subunit 6 OS=Homo sapiens GN=COPS6 PE=1 SV=1 - [CSN6_HUMAN]	1,41	0,00
Q9UK99-3	Isoform 3 of F-box only protein 3 OS=Homo sapiens GN=FBXO3 - [FBX3_HUMAN]	1,71	0,00
O95197-2	Isoform 2 of Reticulon-3 OS=Homo sapiens GN=RTN3 - [RTN3_HUMAN]	1,61	0,00
O94762	ATP-dependent DNA helicase Q5 OS=Homo sapiens GN=RECQL5 PE=1 SV=2 - [RECQ5_HUMAN]	1,57	0,00
Q8TA86	Retinitis pigmentosa 9 protein OS=Homo sapiens GN=RP9 PE=1 SV=2 - [RP9_HUMAN]	1,31	0,00
Q86XE3	EF-hand domain-containing family member A2 OS=Homo sapiens GN=EFHA2 PE=2 SV=1 - [EFHA2_HUMAN]	1,71	0,00
P55795	Heterogeneous nuclear ribonucleoprotein H2 OS=Homo sapiens GN=HNRNPH2 PE=1 SV=1 - [HNRH2_HUMAN]	1,49	0,00
O00443	Phosphatidylinositol-4-phosphate 3-kinase C2 domain-containing subunit alpha OS=Homo sapiens GN=PIK3C2A PE=1	1,44	0,00
Q5BJH7-3	Isoform 3 of Protein YIF1B OS=Homo sapiens GN=YIF1B - [YIF1B_HUMAN]	1,76	0,00
P04040	Catalase OS=Homo sapiens GN=CAT PE=1 SV=3 - [CATA_HUMAN]	1,65	0,00

A375 vs. A375VR4

Q6UXD5-3	Isoform 3 of Seizure 6-like protein 2 OS=Homo sapiens GN=SEZ6L2 - [SE6L2_HUMAN]	1,49	0,00
Q9H6E5	Speckle targeted PIP5K1A-regulated poly(A) polymerase OS=Homo sapiens GN=TUT1 PE=1 SV=2 - [STPAP_HUMAN]	1,40	0,00
P23497-4	Isoform Sp100-C of Nuclear autoantigen Sp-100 OS=Homo sapiens GN=SP100 - [SP100_HUMAN]	2,19	0,00
Q6UW63	KDEL motif-containing protein 1 OS=Homo sapiens GN=KDELC1 PE=1 SV=1 - [KDEL1_HUMAN]	1,90	0,00
Q9NSV4-1	Isoform 1 of Protein diaphanous homolog 3 OS=Homo sapiens GN=DIAPH3 - [DIAP3_HUMAN]	1,78	0,00
O43688	Lipid phosphate phosphohydrolase 2 OS=Homo sapiens GN=PPAP2C PE=1 SV=1 - [LPP2_HUMAN]	1,80	0,00
Q9BRU9	rRNA-processing protein UTP23 homolog OS=Homo sapiens GN=UTP23 PE=2 SV=2 - [UTP23_HUMAN]	1,52	0,00
Q96Q45-2	Isoform 2 of Transmembrane protein 237 OS=Homo sapiens GN=TMEM237 - [TM237_HUMAN]	1,42	0,00
Q9Y624	Junctional adhesion molecule A OS=Homo sapiens GN=F11R PE=1 SV=1 - [JAM1_HUMAN]	1,98	0,00
Q9H9G7-2	Isoform 2 of Protein argonaute-3 OS=Homo sapiens GN=EIF2C3 - [AGO3_HUMAN]	1,41	0,00
Q68CQ7	Glycosyltransferase 8 domain-containing protein 1 OS=Homo sapiens GN=GLT8D1 PE=1 SV=2 - [GL8D1_HUMAN]	2,22	0,00
O14686	Histone-lysine N-methyltransferase MLL2 OS=Homo sapiens GN=MLL2 PE=1 SV=2 - [MLL2_HUMAN]	1,56	0,00
Q9Y5Y5	Peroxisomal membrane protein PEX16 OS=Homo sapiens GN=PEX16 PE=1 SV=2 - [PEX16_HUMAN]	1,74	0,00
Q9BWS9-3	Isoform 3 of Chitinase domain-containing protein 1 OS=Homo sapiens GN=CHID1 - [CHID1_HUMAN]	1,61	0,00
Q14534	Squalene monooxygenase OS=Homo sapiens GN=SQLE PE=2 SV=3 - [ERG1_HUMAN]	1,28	0,00
O14684	Prostaglandin E synthase OS=Homo sapiens GN=PTGES PE=1 SV=2 - [PTGES_HUMAN]	127,17	0,00
P29323-2	Isoform 2 of Ephrin type-B receptor 2 OS=Homo sapiens GN=EPHB2 - [EPHB2_HUMAN]	1,74	0,00
Q9BUA3	Uncharacterized protein C11orf84 OS=Homo sapiens GN=C11orf84 PE=1 SV=3 - [CK084_HUMAN]	1,36	0,00
P09493-5	Isoform 5 of Tropomyosin alpha-1 chain OS=Homo sapiens GN=TPM1 - [TPM1_HUMAN]	1,34	0,00
P62136	Serine/threonine-protein phosphatase PP1-alpha catalytic subunit OS=Homo sapiens GN=PPP1CA PE=1 SV=1 - [PP1A	1,26	0,00
Q6WCQ1-2	Isoform 2 of Myosin phosphatase Rho-interacting protein OS=Homo sapiens GN=MPRIP - [MPRIP_HUMAN]	1,33	0,00
P78356	Phosphatidylinositol-5-phosphate 4-kinase type-2 beta OS=Homo sapiens GN=PIP4K2B PE=1 SV=1 - [PI42B_HUMAN]	1,44	0,00
Q8N684-2	Isoform 2 of Cleavage and polyadenylation specificity factor subunit 7 OS=Homo sapiens GN=CPSF7 - [CPSF7_HUMAN	1,54	0,00
P49768-2	Isoform 2 of Presenilin-1 OS=Homo sapiens GN=PSEN1 - [PSN1_HUMAN]	1,54	0,00
Q9H173	Nucleotide exchange factor SIL1 OS=Homo sapiens GN=SIL1 PE=1 SV=1 - [SIL1_HUMAN]	1,60	0,00
Q8IWE4	DCN1-like protein 3 OS=Homo sapiens GN=DCUN1D3 PE=2 SV=1 - [DCNL3_HUMAN]	1,60	0,00
Q6XQN6-3	Isoform 3 of Nicotinate phosphoribosyltransferase OS=Homo sapiens GN=NAPRT1 - [PNCB_HUMAN]	48,20	0,00
P05165-2	Isoform 2 of Propionyl-CoA carboxylase alpha chain, mitochondrial OS=Homo sapiens GN=PCCA - [PCCA_HUMAN]	1,71	0,00
Q8WVM8	Sec1 family domain-containing protein 1 OS=Homo sapiens GN=SCFD1 PE=1 SV=4 - [SCFD1_HUMAN]	1,49	0,00
Q8N138-4	Isoform 2 of ORM1-like protein 3 OS=Homo sapiens GN=ORMDL3 - [ORML3_HUMAN]	1,61	0,00
Q9Y3E1	Hepatoma-derived growth factor-related protein 3 OS=Homo sapiens GN=HDGFRP3 PE=1 SV=1 - [HDGR3_HUMAN]	1,42	0,00
P08174	Complement decay-accelerating factor OS=Homo sapiens GN=CD55 PE=1 SV=4 - [DAF_HUMAN]	1,94	0,00
Q9Y256	CAAX prenyl protease 2 OS=Homo sapiens GN=RCE1 PE=1 SV=1 - [FACE2_HUMAN]	1,80	0,00

A375 vs. A375VR4

Q14112-2	Isoform 2 of Nidogen-2 OS=Homo sapiens GN=NID2 - [NID2_HUMAN]	1,33	0,00
Q14108	Lysosome membrane protein 2 OS=Homo sapiens GN=SCARB2 PE=1 SV=2 - [SCRB2_HUMAN]	1,73	0,00
Q9Y5L4	Mitochondrial import inner membrane translocase subunit Tim13 OS=Homo sapiens GN=TIMM13 PE=1 SV=1 - [TIM1	1,60	0,00
P02794	Ferritin heavy chain OS=Homo sapiens GN=FTH1 PE=1 SV=2 - [FRIH_HUMAN]	1,70	0,00
Q27J81-2	Isoform 2 of Inverted formin-2 OS=Homo sapiens GN=INF2 - [INF2_HUMAN]	1,34	0,00
Q96MX0	CKLF-like MARVEL transmembrane domain-containing protein 3 OS=Homo sapiens GN=CMTM3 PE=2 SV=1 - [CKLF3_I	1,70	0,00
Q15170-2	Isoform 2 of Transcription elongation factor A protein-like 1 OS=Homo sapiens GN=TCEAL1 - [TCAL1_HUMAN]	1,37	0,00
Q8N9N2-2	Isoform 2 of Activating signal cointegrator 1 complex subunit 1 OS=Homo sapiens GN=ASCC1 - [ASCC1_HUMAN]	1,45	0,00
Q9H1C4	Protein unc-93 homolog B1 OS=Homo sapiens GN=UNC93B1 PE=1 SV=2 - [UN93B_HUMAN]	1,48	0,00
Q86UL8-2	Isoform 2 of Membrane-associated guanylate kinase, WW and PDZ domain-containing protein 2 OS=Homo sapiens G	51,36	0,00
Q86W92-2	Isoform 2 of Liprin-beta-1 OS=Homo sapiens GN=PPFIBP1 - [LIPB1_HUMAN]	1,61	0,00
Q9NP84	Tumor necrosis factor receptor superfamily member 12A OS=Homo sapiens GN=TNFRSF12A PE=1 SV=1 - [TNR12_HU	1,70	0,00
Q9UI43	Putative ribosomal RNA methyltransferase 2 OS=Homo sapiens GN=FTSJ2 PE=1 SV=1 - [RRMJ2_HUMAN]	1,31	0,00
Q9H4L5-2	Isoform 1b of Oxysterol-binding protein-related protein 3 OS=Homo sapiens GN=OSBPL3 - [OSBL3_HUMAN]	1,32	0,00
Q68DH5	LMBR1 domain-containing protein 2 OS=Homo sapiens GN=LMBRD2 PE=1 SV=1 - [LMBD2_HUMAN]	1,51	0,00
Q7Z739	YTH domain family protein 3 OS=Homo sapiens GN=YTHDF3 PE=1 SV=1 - [YTHD3_HUMAN]	1,56	0,12
Q8NFJ5	Retinoic acid-induced protein 3 OS=Homo sapiens GN=GPRC5A PE=1 SV=2 - [RAI3_HUMAN]	2,51	0,12
Q8WUJ3-2	Isoform 2 of Protein KIAA1199 OS=Homo sapiens GN=KIAA1199 - [K1199_HUMAN]	1,44	0,12
Q96IG2-2	Isoform 2 of F-box/LRR-repeat protein 20 OS=Homo sapiens GN=FBXL20 - [FXL20_HUMAN]	1,63	0,12
Q9Y2K6	Ubiquitin carboxyl-terminal hydrolase 20 OS=Homo sapiens GN=USP20 PE=1 SV=2 - [UBP20_HUMAN]	1,89	0,12
Q9NZ56	Formin-2 OS=Homo sapiens GN=FMN2 PE=1 SV=4 - [FMN2_HUMAN]	1,55	0,12
Q14103-3	Isoform 3 of Heterogeneous nuclear ribonucleoprotein D0 OS=Homo sapiens GN=HNRNPD - [HNRPD_HUMAN]	1,55	0,12
Q8IUF8-4	Isoform 4 of MYC-induced nuclear antigen OS=Homo sapiens GN=MINA - [MINA_HUMAN]	1,36	0,12
P30443	HLA class I histocompatibility antigen, A-1 alpha chain OS=Homo sapiens GN=HLA-A PE=1 SV=1 - [1A01_HUMAN]	1,21	0,12
Q16832	Discoidin domain-containing receptor 2 OS=Homo sapiens GN=DDR2 PE=1 SV=2 - [DDR2_HUMAN]	1,95	0,12
Q7Z2T5	TRMT1-like protein OS=Homo sapiens GN=TRMT1L PE=1 SV=2 - [TRM1L_HUMAN]	1,40	0,12
Q8NB14	Ubiquitin carboxyl-terminal hydrolase 38 OS=Homo sapiens GN=USP38 PE=2 SV=2 - [UBP38_HUMAN]	1,40	0,12
P42166	Lamina-associated polypeptide 2, isoform alpha OS=Homo sapiens GN=TMPO PE=1 SV=2 - [LAP2A_HUMAN]	1,86	0,12
Q9H813	Transmembrane protein 206 OS=Homo sapiens GN=TMEM206 PE=1 SV=1 - [TM206_HUMAN]	1,33	0,12
Q6PIJ6-3	Isoform 3 of F-box only protein 38 OS=Homo sapiens GN=FBXO38 - [FBX38_HUMAN]	1,59	0,12
O43464-2	Isoform 2 of Serine protease HTRA2, mitochondrial OS=Homo sapiens GN=HTRA2 - [HTRA2_HUMAN]	1,69	0,12
O95159	Zinc finger protein-like 1 OS=Homo sapiens GN=ZFPL1 PE=1 SV=2 - [ZFPL1_HUMAN]	1,72	0,12
Q8IXT5	RNA-binding protein 12B OS=Homo sapiens GN=RBM12B PE=1 SV=2 - [RB12B_HUMAN]	1,64	0,12

A375 vs. A375VR4

Q13190-2	Isoform 2 of Syntaxin-5 OS=Homo sapiens GN=STX5 - [STX5_HUMAN]	1,50	0,12
A6NP61	ZAR1-like protein OS=Homo sapiens GN=ZAR1L PE=2 SV=2 - [ZAR1L_HUMAN]	2,44	0,12
Q8NBQ5	Estradiol 17-beta-dehydrogenase 11 OS=Homo sapiens GN=HSD17B11 PE=1 SV=3 - [DHB11_HUMAN]	2,16	0,12
Q9H7D7-2	Isoform 2 of WD repeat-containing protein 26 OS=Homo sapiens GN=WDR26 - [WDR26_HUMAN]	1,40	0,12
Q8TAD7	Overexpressed in colon carcinoma 1 protein OS=Homo sapiens GN=OCC1 PE=1 SV=2 - [OCC1_HUMAN]	1,70	0,12
P17677	Neuromodulin OS=Homo sapiens GN=GAP43 PE=1 SV=1 - [NEUM_HUMAN]	1,27	0,12
P02766	Transthyretin OS=Homo sapiens GN=TTR PE=1 SV=1 - [TTHY_HUMAN]	2,63	0,12
Q9P246	Stromal interaction molecule 2 OS=Homo sapiens GN=STIM2 PE=1 SV=2 - [STIM2_HUMAN]	1,54	0,12
O14786	Neuropilin-1 OS=Homo sapiens GN=NRP1 PE=1 SV=3 - [NRP1_HUMAN]	1,53	0,12
Q05193-3	Isoform 3 of Dynamin-1 OS=Homo sapiens GN=DNM1 - [DYN1_HUMAN]	1,39	0,12
Q5QP82-2	Isoform 2 of DDB1- and CUL4-associated factor 10 OS=Homo sapiens GN=DCAF10 - [DCA10_HUMAN]	1,54	0,12
Q9HAT2-2	Isoform 2 of Sialate O-acetylesterase OS=Homo sapiens GN=SIAE - [SIAE_HUMAN]	1,59	0,12
P61018	Ras-related protein Rab-4B OS=Homo sapiens GN=RAB4B PE=1 SV=1 - [RAB4B_HUMAN]	1,53	0,12
Q5BKT4	Dol-P-Glc:Glc(2)Man(9)GlcNAc(2)-PP-Dol alpha-1,2-glucosyltransferase OS=Homo sapiens GN=ALG10 PE=1 SV=1 - [AG	1,82	0,12
Q5T5Y3	Calmodulin-regulated spectrin-associated protein 1 OS=Homo sapiens GN=CAMSAP1 PE=1 SV=2 - [CAMP1_HUMAN]	1,42	0,12
P05114	Non-histone chromosomal protein HMG-14 OS=Homo sapiens GN=HMGN1 PE=1 SV=3 - [HMGN1_HUMAN]	1,46	0,12
Q8WV74-2	Isoform 2 of Nucleoside diphosphate-linked moiety X motif 8, mitochondrial OS=Homo sapiens GN=NUDT8 - [NUDT8	1,52	0,12
Q8IWT6	Leucine-rich repeat-containing protein 8A OS=Homo sapiens GN=LRRC8A PE=1 SV=1 - [LRC8A_HUMAN]	1,45	0,12
P13994	Coiled-coil domain-containing protein 130 OS=Homo sapiens GN=CCDC130 PE=1 SV=2 - [CC130_HUMAN]	1,31	0,12
Q96T83	Sodium/hydrogen exchanger 7 OS=Homo sapiens GN=SLC9A7 PE=1 SV=1 - [SL9A7_HUMAN]	1,35	0,12
Q15059	Bromodomain-containing protein 3 OS=Homo sapiens GN=BRD3 PE=1 SV=1 - [BRD3_HUMAN]	1,38	0,12
A2RUB6-3	Isoform 3 of Coiled-coil domain-containing protein 66 OS=Homo sapiens GN=CCDC66 - [CCD66_HUMAN]	1107,94	0,12
Q5M775-4	Isoform 4 of Cytospin-B OS=Homo sapiens GN=SPECC1 - [CYTSB_HUMAN]	1,49	0,12
Q5VYJ5	MAM and LDL-receptor class A domain-containing protein C10orf112 OS=Homo sapiens GN=C10orf112 PE=2 SV=3 - [	1,51	0,12
Q9BQ69	MACRO domain-containing protein 1 OS=Homo sapiens GN=MACROD1 PE=1 SV=2 - [MACD1_HUMAN]	1,34	0,12
P35527	Keratin, type I cytoskeletal 9 OS=Homo sapiens GN=KRT9 PE=1 SV=3 - [K1C9_HUMAN]	1,88	0,12
Q6YN16-2	Isoform 2 of Hydroxysteroid dehydrogenase-like protein 2 OS=Homo sapiens GN=HSDL2 - [HSDL2_HUMAN]	1,44	0,12
O15226	NF-kappa-B-repressing factor OS=Homo sapiens GN=NKRF PE=1 SV=2 - [NKRF_HUMAN]	1,69	0,12
Q6UW78	UPF0723 protein C11orf83 OS=Homo sapiens GN=C11orf83 PE=1 SV=2 - [CK083_HUMAN]	1,69	0,12
Q86VD7	Solute carrier family 25 member 42 OS=Homo sapiens GN=SLC25A42 PE=1 SV=2 - [S2542_HUMAN]	1,69	0,12
Q5NDL2-3	Isoform 3 of Uncharacterized glycosyltransferase AER61 OS=Homo sapiens GN=AER61 - [AER61_HUMAN]	1,44	0,12
P48960-2	Isoform 2 of CD97 antigen OS=Homo sapiens GN=CD97 - [CD97_HUMAN]	1,55	0,12
Q9NUP9	Protein lin-7 homolog C OS=Homo sapiens GN=LIN7C PE=1 SV=1 - [LIN7C_HUMAN]	1,30	0,12

A375 vs. A375VR4

Q8TB37-2	Isoform 2 of Iron-sulfur protein NUBPL OS=Homo sapiens GN=NUBPL - [NUBPL_HUMAN]	1,37	0,12
O00488	Zinc finger protein 593 OS=Homo sapiens GN=ZNF593 PE=1 SV=2 - [ZN593_HUMAN]	1,39	0,12
O60762	Dolichol-phosphate mannosyltransferase OS=Homo sapiens GN=DPM1 PE=1 SV=1 - [DPM1_HUMAN]	1,63	0,12
Q16877	6-phosphofructo-2-kinase/fructose-2,6-biphosphatase 4 OS=Homo sapiens GN=PFKFB4 PE=2 SV=6 - [F264_HUMAN]	1,28	0,12
Q6IPR1	LYR motif-containing protein 5 OS=Homo sapiens GN=LYRM5 PE=2 SV=1 - [LYRM5_HUMAN]	1,45	0,12
Q15771	Ras-related protein Rab-30 OS=Homo sapiens GN=RAB30 PE=1 SV=2 - [RAB30_HUMAN]	1,68	0,12
Q709F0-3	Isoform 3 of Acyl-CoA dehydrogenase family member 11 OS=Homo sapiens GN=ACAD11 - [ACD11_HUMAN]	1,49	0,12
Q0VAQ4	Small cell adhesion glycoprotein OS=Homo sapiens GN=SMAGP PE=1 SV=1 - [SMAGP_HUMAN]	1,30	0,12
Q9UMX1	Suppressor of fused homolog OS=Homo sapiens GN=SUFU PE=1 SV=2 - [SUFU_HUMAN]	1,32	0,12
Q14103-4	Isoform 4 of Heterogeneous nuclear ribonucleoprotein D0 OS=Homo sapiens GN=HNRNPD - [HNRPD_HUMAN]	1,42	0,12
Q6N075	Major facilitator superfamily domain-containing protein 5 OS=Homo sapiens GN=MFSD5 PE=2 SV=2 - [MFSD5_HUMA	1,35	0,12
P17252	Protein kinase C alpha type OS=Homo sapiens GN=PRKCA PE=1 SV=4 - [KPCA_HUMAN]	1,72	0,12
Q8N9R8	Protein SCAI OS=Homo sapiens GN=SCAI PE=1 SV=2 - [SCAI_HUMAN]	1,38	0,12
Q03135	Caveolin-1 OS=Homo sapiens GN=CAV1 PE=1 SV=4 - [CAV1_HUMAN]	2,01	0,12
Q9Y3Q7-2	Isoform 2 of Disintegrin and metalloproteinase domain-containing protein 18 OS=Homo sapiens GN=ADAM18 - [ADA	537719,75	0,12
Q9NXS2	Glutaminyl-peptide cyclotransferase-like protein OS=Homo sapiens GN=QPCTL PE=1 SV=2 - [QPCTL_HUMAN]	1,40	0,12
P05141	ADP/ATP translocase 2 OS=Homo sapiens GN=SLC25A5 PE=1 SV=7 - [ADT2_HUMAN]	1,64	0,12
P23743	Diacylglycerol kinase alpha OS=Homo sapiens GN=DGKA PE=1 SV=3 - [DGKA_HUMAN]	2,70	0,12
Q9NVV0	Trimeric intracellular cation channel type B OS=Homo sapiens GN=TMEM38B PE=1 SV=1 - [TM38B_HUMAN]	1,45	0,12
Q9UDY4	DnaJ homolog subfamily B member 4 OS=Homo sapiens GN=DNAJB4 PE=1 SV=1 - [DNJB4_HUMAN]	1,54	0,12
Q6PJQ5	Forkhead box protein R2 OS=Homo sapiens GN=FOXR2 PE=2 SV=1 - [FOXR2_HUMAN]	1,55	0,12
Q9Y2J2-2	Isoform B of Band 4,1-like protein 3 OS=Homo sapiens GN=EPB41L3 - [E41L3_HUMAN]	1,51	0,12
P05423	DNA-directed RNA polymerase III subunit RPC4 OS=Homo sapiens GN=POLR3D PE=1 SV=2 - [RPC4_HUMAN]	1,39	0,12
Q9BTX3	Transmembrane protein 208 OS=Homo sapiens GN=TMEM208 PE=2 SV=1 - [TM208_HUMAN]	1,37	0,12
P32004-2	Isoform 2 of Neural cell adhesion molecule L1 OS=Homo sapiens GN=L1CAM - [L1CAM_HUMAN]	3,53	0,12
P08779	Keratin, type I cytoskeletal 16 OS=Homo sapiens GN=KRT16 PE=1 SV=4 - [K1C16_HUMAN]	1,86	0,18
Q96DB5	Regulator of microtubule dynamics protein 1 OS=Homo sapiens GN=FAM82B PE=1 SV=1 - [RMD1_HUMAN]	1,38	0,18
Q9Y5U2-2	Isoform 2 of Protein TSSC4 OS=Homo sapiens GN=TSSC4 - [TSSC4_HUMAN]	1,53	0,18
O00186	Syntaxin-binding protein 3 OS=Homo sapiens GN=STXBP3 PE=1 SV=2 - [STXB3_HUMAN]	1,46	0,18
Q9UPA5	Protein bassoon OS=Homo sapiens GN=BSN PE=1 SV=4 - [BSN_HUMAN]	454776773,47	0,18
Q9UHR6	Zinc finger HIT domain-containing protein 2 OS=Homo sapiens GN=ZNHIT2 PE=1 SV=1 - [ZNHI2_HUMAN]	1,35	0,18
Q5SWX8	Protein odr-4 homolog OS=Homo sapiens GN=ODR4 PE=2 SV=1 - [ODR4_HUMAN]	1,50	0,18
Q969M1	Mitochondrial import receptor subunit TOM40B OS=Homo sapiens GN=TOMM40L PE=2 SV=1 - [TM40L_HUMAN]	1,35	0,18

A375 vs. A375VR4

Q9NQ34	Transmembrane protein 9B OS=Homo sapiens GN=TMEM9B PE=1 SV=1 - [TMM9B_HUMAN]	1,39	0,18
Q96I24	Far upstream element-binding protein 3 OS=Homo sapiens GN=FUBP3 PE=1 SV=2 - [FUBP3_HUMAN]	1,40	0,18
Q86YF9-2	Isoform 2 of Zinc finger protein DZIP1 OS=Homo sapiens GN=DZIP1 - [DZIP1_HUMAN]	192,29	0,18
Q9Y6H1	Coiled-coil-helix-coiled-coil-helix domain-containing protein 2, mitochondrial OS=Homo sapiens GN=CHCHD2 PE=1 S\	1,49	0,18
Q9Y394-2	Isoform 2 of Dehydrogenase/reductase SDR family member 7 OS=Homo sapiens GN=DHRS7 - [DHRS7_HUMAN]	1,58	0,18
Q6NUK1	Calcium-binding mitochondrial carrier protein SCaMC-1 OS=Homo sapiens GN=SLC25A24 PE=1 SV=2 - [SCMC1_HUMA	1,62	0,18
O75531	Barrier-to-autointegration factor OS=Homo sapiens GN=BANF1 PE=1 SV=1 - [BAF_HUMAN]	1,32	0,18
Q9ULI2	Beta-citryl-glutamate synthase B OS=Homo sapiens GN=RIMKLB PE=2 SV=2 - [RIMKB_HUMAN]	1,35	0,18
P62072	Mitochondrial import inner membrane translocase subunit Tim10 OS=Homo sapiens GN=TIMM10 PE=1 SV=1 - [TIM1	1,41	0,18
O00622	Protein CYR61 OS=Homo sapiens GN=CYR61 PE=1 SV=1 - [CYR61_HUMAN]	2,21	0,18
Q9C086	INO80 complex subunit B OS=Homo sapiens GN=INO80B PE=1 SV=2 - [IN80B_HUMAN]	1,32	0,18
Q13510	Acid ceramidase OS=Homo sapiens GN=ASAH1 PE=1 SV=5 - [ASAH1_HUMAN]	1,35	0,18
Q9BZI7-2	Isoform 2 of Regulator of nonsense transcripts 3B OS=Homo sapiens GN=UPF3B - [REN3B_HUMAN]	1,36	0,18
Q12805-2	Isoform 2 of EGF-containing fibulin-like extracellular matrix protein 1 OS=Homo sapiens GN=EFEMP1 - [FBLN3_HUMA	1,45	0,18
Q6PIU2	Neutral cholesterol ester hydrolase 1 OS=Homo sapiens GN=NCEH1 PE=1 SV=3 - [NCEH1_HUMAN]	1,66	0,18
Q9H160	Inhibitor of growth protein 2 OS=Homo sapiens GN=ING2 PE=1 SV=2 - [ING2_HUMAN]	1,51	0,18
Q8NE86-3	Isoform 3 of Calcium uniporter protein, mitochondrial OS=Homo sapiens GN=MCU - [MCU_HUMAN]	1,70	0,18
Q86UU0-3	Isoform 3 of B-cell CLL/lymphoma 9-like protein OS=Homo sapiens GN=BCL9L - [BCL9L_HUMAN]	1,67	0,18
Q9P2Y4	Zinc finger protein 219 OS=Homo sapiens GN=ZNF219 PE=1 SV=2 - [ZN219_HUMAN]	1,24	0,18
Q8N8Q8	Mitochondrial inner membrane protein COX18 OS=Homo sapiens GN=COX18 PE=2 SV=1 - [COX18_HUMAN]	1,37	0,18
Q8IZR5-3	Isoform 3 of CKLF-like MARVEL transmembrane domain-containing protein 4 OS=Homo sapiens GN=CMTM4 - [CKLF4	1,57	0,18
Q07617	Sperm-associated antigen 1 OS=Homo sapiens GN=SPAG1 PE=1 SV=3 - [SPAG1_HUMAN]	1,37	0,18
Q68CR1-3	Isoform 3 of Protein sel-1 homolog 3 OS=Homo sapiens GN=SEL1L3 - [SE1L3_HUMAN]	2,02	0,18
Q8IY33-3	Isoform 3 of MICAL-like protein 2 OS=Homo sapiens GN=MICALL2 - [MILK2_HUMAN]	1,45	0,18
P53680-2	Isoform 2 of AP-2 complex subunit sigma OS=Homo sapiens GN=AP2S1 - [AP2S1_HUMAN]	1,26	0,18
Q9BSD7	Cancer-related nucleoside-triphosphatase OS=Homo sapiens GN=NTPCR PE=1 SV=1 - [NTPCR_HUMAN]	1,55	0,18
Q86SQ0-2	Isoform 2 of Pleckstrin homology-like domain family B member 2 OS=Homo sapiens GN=PHLDB2 - [PHLB2_HUMAN]	1,29	0,18
Q9UDW1	Cytochrome b-c1 complex subunit 9 OS=Homo sapiens GN=UQCR10 PE=1 SV=3 - [QCR9_HUMAN]	1,38	0,18
P33897	ATP-binding cassette sub-family D member 1 OS=Homo sapiens GN=ABCD1 PE=1 SV=2 - [ABCD1_HUMAN]	1,59	0,18
Q9Y657	Spindlin-1 OS=Homo sapiens GN=SPIN1 PE=1 SV=3 - [SPIN1_HUMAN]	1,56	0,18
Q07157	Tight junction protein ZO-1 OS=Homo sapiens GN=TJP1 PE=1 SV=3 - [ZO1_HUMAN]	1,39	0,18
P35908	Keratin, type II cytoskeletal 2 epidermal OS=Homo sapiens GN=KRT2 PE=1 SV=2 - [K22E_HUMAN]	1,40	0,18
P35658-2	Isoform 2 of Nuclear pore complex protein Nup214 OS=Homo sapiens GN=NUP214 - [NU214_HUMAN]	1,20	0,18

A375 vs. A375VR4

Q99986	Serine/threonine-protein kinase VRK1 OS=Homo sapiens GN=VRK1 PE=1 SV=1 - [VRK1_HUMAN]	1,22	0,18
Q14332	Frizzled-2 OS=Homo sapiens GN=FZD2 PE=2 SV=1 - [FZD2_HUMAN]	1,86	0,18
P62699	Protein yippee-like 5 OS=Homo sapiens GN=YPEL5 PE=2 SV=1 - [YPEL5_HUMAN]	1,43	0,18
Q9BZE2	tRNA pseudouridine(38/39) synthase OS=Homo sapiens GN=PUS3 PE=1 SV=3 - [PUS3_HUMAN]	1,60	0,18
O95236-2	Isoform 2 of Apolipoprotein L3 OS=Homo sapiens GN=APOL3 - [APOL3_HUMAN]	3600,95	0,18
P53365	Arfaptin-2 OS=Homo sapiens GN=ARFIP2 PE=1 SV=1 - [ARFP2_HUMAN]	1,74	0,18
P55789	FAD-linked sulfhydryl oxidase ALR OS=Homo sapiens GN=GFER PE=1 SV=2 - [ALR_HUMAN]	1,44	0,18
Q9Y5J7	Mitochondrial import inner membrane translocase subunit Tim9 OS=Homo sapiens GN=TIMM9 PE=1 SV=1 - [TIM9_H	1,43	0,18
Q8WV07	Oral cancer-overexpressed protein 1 OS=Homo sapiens GN=ORAOV1 PE=2 SV=2 - [ORAV1_HUMAN]	1,54	0,18
P41219	Peripherin OS=Homo sapiens GN=PRPH PE=1 SV=2 - [PERI_HUMAN]	1024922,46	0,18
P45973	Chromobox protein homolog 5 OS=Homo sapiens GN=CBX5 PE=1 SV=1 - [CBX5_HUMAN]	1,30	0,18
Q9UHB9	Signal recognition particle 68 kDa protein OS=Homo sapiens GN=SRP68 PE=1 SV=2 - [SRP68_HUMAN]	1,27	0,18
Q96QE5	Transcription elongation factor, mitochondrial OS=Homo sapiens GN=TEFM PE=1 SV=1 - [TEFM_HUMAN]	1,33	0,18
Q96P16-3	Isoform 3 of Regulation of nuclear pre-mRNA domain-containing protein 1A OS=Homo sapiens GN=RPRD1A - [RPR1A	1,22	0,18
Q8TCD1	UPF0729 protein C18orf32 OS=Homo sapiens GN=C18orf32 PE=2 SV=1 - [CR032_HUMAN]	1,54	0,18
Q9NXC5-2	Isoform 2 of WD repeat-containing protein mio OS=Homo sapiens GN=MIOS - [MIO_HUMAN]	1,23	0,18
A2A2Y4-5	Isoform 5 of FERM domain-containing protein 3 OS=Homo sapiens GN=FRMD3 - [FRMD3_HUMAN]	1,48	0,18
Q8NFQ8	Torsin-1A-interacting protein 2 OS=Homo sapiens GN=TOR1AIP2 PE=1 SV=1 - [TOIP2_HUMAN]	1,54	0,18
Q15149-3	Isoform 3 of Plectin OS=Homo sapiens GN=PLEC - [PLEC_HUMAN]	1,64	0,18
Q5M9Q1	NKAP-like protein OS=Homo sapiens GN=NKAPL PE=1 SV=3 - [NKAPL_HUMAN]	1,38	0,18
Q13232	Nucleoside diphosphate kinase 3 OS=Homo sapiens GN=NME3 PE=1 SV=2 - [NDK3_HUMAN]	1,44	0,18
Q9H3H1-4	Isoform 4 of tRNA dimethylallyltransferase, mitochondrial OS=Homo sapiens GN=TRIT1 - [MOD5_HUMAN]	1,24	0,18
Q9BYD2	39S ribosomal protein L9, mitochondrial OS=Homo sapiens GN=MRPL9 PE=1 SV=2 - [RM09_HUMAN]	1,41	0,18
P04004	Vitronectin OS=Homo sapiens GN=VTN PE=1 SV=1 - [VTNC_HUMAN]	1,77	0,18
Q9H6V9	UPF0554 protein C2orf43 OS=Homo sapiens GN=C2orf43 PE=1 SV=1 - [CB043_HUMAN]	1,31	0,18
Q92805	Golgin subfamily A member 1 OS=Homo sapiens GN=GOLGA1 PE=1 SV=3 - [GOGA1_HUMAN]	1,39	0,18
P17813-2	Isoform Short of Endoglin OS=Homo sapiens GN=ENG - [EGLN_HUMAN]	1,57	0,18
Q6XZF7	Dynamin-binding protein OS=Homo sapiens GN=DNMBP PE=1 SV=1 - [DNMBP_HUMAN]	1,94	0,18
Q6ZUT1-3	Isoform 3 of Uncharacterized protein C11orf57 OS=Homo sapiens GN=C11orf57 - [CK057_HUMAN]	1,50	0,18
Q86VU5	Catechol O-methyltransferase domain-containing protein 1 OS=Homo sapiens GN=COMTD1 PE=1 SV=1 - [CMTD1_HU	1,44	0,18
Q6IAN0	Dehydrogenase/reductase SDR family member 7B OS=Homo sapiens GN=DHRS7B PE=1 SV=2 - [DRS7B_HUMAN]	1,45	0,18
Q8TCX5-2	Isoform 2 of Rhophilin-1 OS=Homo sapiens GN=RHPN1 - [RHPN1_HUMAN]	1,43	0,18
P0C2W1	F-box/SPRY domain-containing protein 1 OS=Homo sapiens GN=FBXO45 PE=1 SV=1 - [FBSP1_HUMAN]	1,38	0,18

A375 vs. A375VR4

Q96QD9	UAP56-interacting factor OS=Homo sapiens GN=FYTTD1 PE=1 SV=3 - [UIF_HUMAN]	1,46	0,18
Q92613	Protein Jade-3 OS=Homo sapiens GN=PHF16 PE=1 SV=1 - [JADE3_HUMAN]	1,43	0,18
Q9HD45	Transmembrane 9 superfamily member 3 OS=Homo sapiens GN=TM9SF3 PE=1 SV=2 - [TM9S3_HUMAN]	1,45	0,18
Q9NVT9	Armadillo repeat-containing protein 1 OS=Homo sapiens GN=ARMC1 PE=1 SV=1 - [ARMC1_HUMAN]	1,39	0,18
Q86SQ9-3	Isoform 3 of Dehydrodolichyl diphosphate synthase OS=Homo sapiens GN=DHDDS - [DHDDS_HUMAN]	1,36	0,18
Q9H9Q2-2	Isoform 2 of COP9 signalosome complex subunit 7b OS=Homo sapiens GN=COPS7B - [CSN7B_HUMAN]	1,51	0,18
Q9UHQ9	NADH-cytochrome b5 reductase 1 OS=Homo sapiens GN=CYB5R1 PE=1 SV=1 - [NB5R1_HUMAN]	1,49	0,18
P84157-2	Isoform 2 of Matrix-remodeling-associated protein 7 OS=Homo sapiens GN=MXRA7 - [MXRA7_HUMAN]	1,52	0,18
Q969U6	F-box/WD repeat-containing protein 5 OS=Homo sapiens GN=FBXW5 PE=1 SV=1 - [FBXW5_HUMAN]	1,81	0,18
Q14980	Nuclear mitotic apparatus protein 1 OS=Homo sapiens GN=NUMA1 PE=1 SV=2 - [NUMA1_HUMAN]	1,70	0,18
O94826	Mitochondrial import receptor subunit TOM70 OS=Homo sapiens GN=TOMM70A PE=1 SV=1 - [TOM70_HUMAN]	1,49	0,18
Q86SK9-2	Isoform 2 of Stearoyl-CoA desaturase 5 OS=Homo sapiens GN=SCD5 - [SCD5_HUMAN]	1,45	0,18
P83369	U7 snRNA-associated Sm-like protein LSm11 OS=Homo sapiens GN=LSM11 PE=1 SV=2 - [LSM11_HUMAN]	1,34	0,18
Q9BSU3	N-alpha-acetyltransferase 11 OS=Homo sapiens GN=NAA11 PE=1 SV=3 - [NAA11_HUMAN]	1,86	0,18
Q9P0I2	Transmembrane protein 111 OS=Homo sapiens GN=TMEM111 PE=1 SV=3 - [TM111_HUMAN]	1,48	0,18
Q9BX59	Tapasin-related protein OS=Homo sapiens GN=TAPBPL PE=1 SV=2 - [TPSNR_HUMAN]	1,38	0,18
O14828	Secretory carrier-associated membrane protein 3 OS=Homo sapiens GN=SCAMP3 PE=1 SV=3 - [SCAM3_HUMAN]	1,29	0,18
Q96D31	Calcium release-activated calcium channel protein 1 OS=Homo sapiens GN=ORAI1 PE=1 SV=2 - [CRCM1_HUMAN]	1,60	0,18
Q16637-4	Isoform SMN-delta57 of Survival motor neuron protein OS=Homo sapiens GN=SMN1 - [SMN_HUMAN]	1,29	0,18
Q96CB9-3	Isoform 3 of Putative methyltransferase NSUN4 OS=Homo sapiens GN=NSUN4 - [NSUN4_HUMAN]	1,28	0,18
Q8N4Q1	Mitochondrial intermembrane space import and assembly protein 40 OS=Homo sapiens GN=CHCHD4 PE=1 SV=1 - [M	1,54	0,18
Q9NQE9	Histidine triad nucleotide-binding protein 3 OS=Homo sapiens GN=HINT3 PE=1 SV=1 - [HINT3_HUMAN]	1,35	0,18
Q9NZW5	MAGUK p55 subfamily member 6 OS=Homo sapiens GN=MPP6 PE=1 SV=2 - [MPP6_HUMAN]	1,29	0,18
P80303	Nucleobindin-2 OS=Homo sapiens GN=NUCB2 PE=1 SV=2 - [NUCB2_HUMAN]	1,37	0,18
Q8N2Y8	Iporin OS=Homo sapiens GN=RUSC2 PE=1 SV=3 - [RUSC2_HUMAN]	1,43	0,18
Q03164-2	Isoform 14P-18B of Histone-lysine N-methyltransferase MLL OS=Homo sapiens GN=MLL - [MLL1_HUMAN]	1,38	0,18
P81605	Dermcidin OS=Homo sapiens GN=DCD PE=1 SV=2 - [DCD_HUMAN]	1,43	0,18
Q16394	Exostosin-1 OS=Homo sapiens GN=EXT1 PE=1 SV=2 - [EXT1_HUMAN]	1,83	0,18
Q03518	Antigen peptide transporter 1 OS=Homo sapiens GN=TAP1 PE=1 SV=2 - [TAP1_HUMAN]	1,26	0,18
Q6MZP7-4	Isoform 4 of Protein lin-54 homolog OS=Homo sapiens GN=LIN54 - [LIN54_HUMAN]	1,28	0,18
O43824	Putative GTP-binding protein 6 OS=Homo sapiens GN=GTPBP6 PE=2 SV=3 - [GTPB6_HUMAN]	2,01	0,18
Q13557-12	Isoform Delta 12 of Calcium/calmodulin-dependent protein kinase type II subunit delta OS=Homo sapiens GN=CAMK	1,29	0,18
Q9NW08	DNA-directed RNA polymerase III subunit RPC2 OS=Homo sapiens GN=POLR3B PE=1 SV=2 - [RPC2_HUMAN]	1,37	0,18

A375 vs. A375VR4

Q08379	Golgin subfamily A member 2 OS=Homo sapiens GN=GOLGA2 PE=1 SV=3 - [GOGA2_HUMAN]	1,42	0,18
O14763-2	Isoform Short of Tumor necrosis factor receptor superfamily member 10B OS=Homo sapiens GN=TNFRSF10B - [TR10I	1,30	0,18
P57105	Synaptojanin-2-binding protein OS=Homo sapiens GN=SYNJ2BP PE=1 SV=2 - [SYJ2B_HUMAN]	1,51	0,18
Q14197	Peptidyl-tRNA hydrolase ICT1, mitochondrial OS=Homo sapiens GN=ICT1 PE=1 SV=1 - [ICT1_HUMAN]	1,33	0,18
Q8WVQ1-2	Isoform 2 of Soluble calcium-activated nucleotidase 1 OS=Homo sapiens GN=CANT1 - [CANT1_HUMAN]	1,39	0,18
P27105	Erythrocyte band 7 integral membrane protein OS=Homo sapiens GN=STOM PE=1 SV=3 - [STOM_HUMAN]	1,35	0,18
Q8TC29	Enkurin OS=Homo sapiens GN=ENKUR PE=2 SV=1 - [ENKUR_HUMAN]	1,57	0,18
Q9NS15	Latent-transforming growth factor beta-binding protein 3 OS=Homo sapiens GN=LTBP3 PE=1 SV=4 - [LTBP3_HUMAN]	1,35	0,18
O14523	C2 domain-containing protein 2-like OS=Homo sapiens GN=C2CD2L PE=1 SV=3 - [C2C2L_HUMAN]	1,87	0,18
Q96IZ0	PRKC apoptosis WT1 regulator protein OS=Homo sapiens GN=PAWR PE=1 SV=1 - [PAWR_HUMAN]	1,56	0,18
Q70UQ0	Inhibitor of nuclear factor kappa-B kinase-interacting protein OS=Homo sapiens GN=IKBIP PE=1 SV=1 - [IKIP_HUMAN	1,45	0,18
Q6PCB8	Embigin OS=Homo sapiens GN=EMB PE=1 SV=1 - [EMB_HUMAN]	1,41	0,18
Q9UII2-3	Isoform 3 of ATPase inhibitor, mitochondrial OS=Homo sapiens GN=ATPIF1 - [ATIF1_HUMAN]	1,38	0,18
Q9C005	Protein dpy-30 homolog OS=Homo sapiens GN=DPY30 PE=1 SV=1 - [DPY30_HUMAN]	1,35	0,18
Q6UUV9-3	Isoform 3 of CREB-regulated transcription coactivator 1 OS=Homo sapiens GN=CRTC1 - [CRTC1_HUMAN]	1,55	0,18
P29279-2	Isoform 2 of Connective tissue growth factor OS=Homo sapiens GN=CTGF - [CTGF_HUMAN]	1,88	0,18
Q13563	Polycystin-2 OS=Homo sapiens GN=PKD2 PE=1 SV=3 - [PKD2_HUMAN]	1,51	0,18
Q07812-5	Isoform Epsilon of Apoptosis regulator BAX OS=Homo sapiens GN=BAX - [BAX_HUMAN]	1,40	0,18
Q9H3K2	Growth hormone-inducible transmembrane protein OS=Homo sapiens GN=GHITM PE=1 SV=2 - [GHITM_HUMAN]	1,56	0,18
P35226	Polycomb complex protein BMI-1 OS=Homo sapiens GN=BMI1 PE=1 SV=2 - [BMI1_HUMAN]	1,36	0,41
Q9BX10-2	Isoform 2 of GTP-binding protein 2 OS=Homo sapiens GN=GTPBP2 - [GTPB2_HUMAN]	1,41	0,41
P61011	Signal recognition particle 54 kDa protein OS=Homo sapiens GN=SRP54 PE=1 SV=1 - [SRP54_HUMAN]	1,25	0,41
O14773	Tripeptidyl-peptidase 1 OS=Homo sapiens GN=TPP1 PE=1 SV=2 - [TPP1_HUMAN]	1,34	0,41
O43766	Lipoyl synthase, mitochondrial OS=Homo sapiens GN=LIAS PE=2 SV=3 - [LIAS_HUMAN]	1,40	0,41
P11233	Ras-related protein Ral-A OS=Homo sapiens GN=RALA PE=1 SV=1 - [RALA_HUMAN]	1,47	0,41
Q8NBL1	Protein O-glucosyltransferase 1 OS=Homo sapiens GN=POGLUT1 PE=1 SV=1 - [PGLT1_HUMAN]	1,29	0,41
Q13488	V-type proton ATPase 116 kDa subunit a isoform 3 OS=Homo sapiens GN=TCIRG1 PE=1 SV=3 - [VPP3_HUMAN]	1,50	0,41
Q53GQ0	Estradiol 17-beta-dehydrogenase 12 OS=Homo sapiens GN=HSD17B12 PE=1 SV=2 - [DHB12_HUMAN]	1,50	0,41
Q9H869-6	Isoform 6 of YY1-associated protein 1 OS=Homo sapiens GN=YY1AP1 - [YYAP1_HUMAN]	1,25	0,41
Q9BVN2-2	Isoform 2 of RUN and SH3 domain-containing protein 1 OS=Homo sapiens GN=RUSC1 - [RUSC1_HUMAN]	1,63	0,41
P0C7T5	Ataxin-1-like OS=Homo sapiens GN=ATXN1L PE=1 SV=1 - [ATX1L_HUMAN]	1,36	0,41
O76094	Signal recognition particle 72 kDa protein OS=Homo sapiens GN=SRP72 PE=1 SV=3 - [SRP72_HUMAN]	1,25	0,41
P50416	Carnitine O-palmitoyltransferase 1, liver isoform OS=Homo sapiens GN=CPT1A PE=1 SV=2 - [CPT1A_HUMAN]	1,47	0,41

A375 vs. A375VR4

Q9Y6C9	Mitochondrial carrier homolog 2 OS=Homo sapiens GN=MTCH2 PE=1 SV=1 - [MTCH2_HUMAN]	1,36	0,41
P09382	Galectin-1 OS=Homo sapiens GN=LGALS1 PE=1 SV=2 - [LEG1_HUMAN]	1,49	0,41
P99999	Cytochrome c OS=Homo sapiens GN=CYCS PE=1 SV=2 - [CYC_HUMAN]	1,31	0,41
Q5JTD0-2	Isoform 2 of Tight junction-associated protein 1 OS=Homo sapiens GN=TJAP1 - [TJAP1_HUMAN]	1,47	0,41
Q6UWE0-3	Isoform 3 of E3 ubiquitin-protein ligase LRSAM1 OS=Homo sapiens GN=LRSAM1 - [LRSM1_HUMAN]	1,49	0,41
P02533	Keratin, type I cytoskeletal 14 OS=Homo sapiens GN=KRT14 PE=1 SV=4 - [K1C14_HUMAN]	1,31	0,41
Q14764	Major vault protein OS=Homo sapiens GN=MVP PE=1 SV=4 - [MVP_HUMAN]	2,40	0,41
O60907-2	Isoform 2 of F-box-like/WD repeat-containing protein TBL1X OS=Homo sapiens GN=TBL1X - [TBL1X_HUMAN]	1,66	0,41
O43920	NADH dehydrogenase [ubiquinone] iron-sulfur protein 5 OS=Homo sapiens GN=NDUFS5 PE=1 SV=3 - [NDUS5_HUMA	1,45	0,41
P51957-2	Isoform 2 of Serine/threonine-protein kinase Nek4 OS=Homo sapiens GN=NEK4 - [NEK4_HUMAN]	1,82	0,41
Q5BJD5-2	Isoform 2 of Transmembrane protein 41B OS=Homo sapiens GN=TMEM41B - [TM41B_HUMAN]	1,50	0,41
Q8NEZ5	F-box only protein 22 OS=Homo sapiens GN=FBXO22 PE=1 SV=1 - [FBX22_HUMAN]	2,63	0,41
P08107	Heat shock 70 kDa protein 1A/1B OS=Homo sapiens GN=HSPA1A PE=1 SV=5 - [HSP71_HUMAN]	1,73	0,41
Q13243	Serine/arginine-rich splicing factor 5 OS=Homo sapiens GN=SRSF5 PE=1 SV=1 - [SRSF5_HUMAN]	1,24	0,41
Q96HV5	Transmembrane protein 41A OS=Homo sapiens GN=TMEM41A PE=2 SV=1 - [TM41A_HUMAN]	1,50	0,41
Q6RI45-5	Isoform 5 of Bromodomain and WD repeat-containing protein 3 OS=Homo sapiens GN=BRWD3 - [BRWD3_HUMAN]	1,25	0,41
Q53TN4	Cytochrome b reductase 1 OS=Homo sapiens GN=CYBRD1 PE=1 SV=1 - [CYBR1_HUMAN]	1,36	0,41
Q2PZI1-2	Isoform 2 of Protein dpy-19 homolog 1 OS=Homo sapiens GN=DPY19L1 - [D19L1_HUMAN]	1,47	0,41
Q15070-2	Isoform 2 of Mitochondrial inner membrane protein OXA1L OS=Homo sapiens GN=OXA1L - [OXA1L_HUMAN]	1,38	0,41
Q9UJV9	Probable ATP-dependent RNA helicase DDX41 OS=Homo sapiens GN=DDX41 PE=1 SV=2 - [DDX41_HUMAN]	1,40	0,41
O14979-3	Isoform 3 of Heterogeneous nuclear ribonucleoprotein D-like OS=Homo sapiens GN=HNRPDL - [HNRDL_HUMAN]	1,42	0,41
Q9P0J0	NADH dehydrogenase [ubiquinone] 1 alpha subcomplex subunit 13 OS=Homo sapiens GN=NDUFA13 PE=1 SV=3 - [ND	1,52	0,41
Q96GC9	Vacuole membrane protein 1 OS=Homo sapiens GN=VMP1 PE=1 SV=1 - [VMP1_HUMAN]	1,42	0,41
P07948-2	Isoform 2 of Tyrosine-protein kinase Lyn OS=Homo sapiens GN=LYN - [LYN_HUMAN]	1,39	0,41
Q7L0J3-2	Isoform 2 of Synaptic vesicle glycoprotein 2A OS=Homo sapiens GN=SV2A - [SV2A_HUMAN]	1,30	0,41
P20930	Filaggrin OS=Homo sapiens GN=FLG PE=1 SV=3 - [FILA_HUMAN]	1,34	0,41
Q96C92-4	Isoform 4 of Serologically defined colon cancer antigen 3 OS=Homo sapiens GN=SDCCAG3 - [SDCG3_HUMAN]	1,41	0,41
P78540	Arginase-2, mitochondrial OS=Homo sapiens GN=ARG2 PE=1 SV=1 - [ARGI2_HUMAN]	2,03	0,41
P19256-2	Isoform 2 of Lymphocyte function-associated antigen 3 OS=Homo sapiens GN=CD58 - [LFA3_HUMAN]	1,31	0,41
Q9NQY0-2	Isoform 2 of Bridging integrator 3 OS=Homo sapiens GN=BIN3 - [BIN3_HUMAN]	1,63	0,41
Q9Y2M5	Kelch-like protein 20 OS=Homo sapiens GN=KLHL20 PE=1 SV=4 - [KLH20_HUMAN]	1,23	0,41
P33908	Mannosyl-oligosaccharide 1,2-alpha-mannosidase IA OS=Homo sapiens GN=MAN1A1 PE=1 SV=3 - [MA1A1_HUMAN]	1,64	0,41
O00469-2	Isoform 2 of Procollagen-lysine,2-oxoglutarate 5-dioxygenase 2 OS=Homo sapiens GN=PLOD2 - [PLOD2_HUMAN]	1,48	0,41

Q9P0L9-4	Isoform 4 of Polycystic kidney disease 2-like 1 protein OS=Homo sapiens GN=PKD2L1 - [PK2L1_HUMAN]	1,19	0,41
Q92878	DNA repair protein RAD50 OS=Homo sapiens GN=RAD50 PE=1 SV=1 - [RAD50_HUMAN]	1,36	0,41
Q3KR37-2	Isoform 2 of GRAM domain-containing protein 1B OS=Homo sapiens GN=GRAMD1B - [GRM1B_HUMAN]	1,47	0,41
Q7Z6M4	mTERF domain-containing protein 2 OS=Homo sapiens GN=MTERFD2 PE=1 SV=3 - [MTER2_HUMAN]	1,31	0,41
Q5VZF2-3	Isoform 3 of Muscleblind-like protein 2 OS=Homo sapiens GN=MBNL2 - [MBNL2_HUMAN]	1,38	0,41
P25686-2	Isoform 2 of DnaJ homolog subfamily B member 2 OS=Homo sapiens GN=DNAJB2 - [DNJB2_HUMAN]	1,22	0,41
Q08722-2	Isoform OA3-293 of Leukocyte surface antigen CD47 OS=Homo sapiens GN=CD47 - [CD47_HUMAN]	1,41	0,41
P22307	Non-specific lipid-transfer protein OS=Homo sapiens GN=SCP2 PE=1 SV=2 - [NLTP_HUMAN]	1,36	0,41
Q8N9A8	Transmembrane protein 188 OS=Homo sapiens GN=TMEM188 PE=2 SV=1 - [TM188_HUMAN]	1,44	0,41
Q8ND76-2	Isoform 2 of Cyclin-Y OS=Homo sapiens GN=CCNY - [CCNY_HUMAN]	1,41	0,41
O75971-2	Isoform 2 of snRNA-activating protein complex subunit 5 OS=Homo sapiens GN=SNAPC5 - [SNPC5_HUMAN]	1,30	0,41
P51116	Fragile X mental retardation syndrome-related protein 2 OS=Homo sapiens GN=FXR2 PE=1 SV=2 - [FXR2_HUMAN]	1,19	0,41
P22692	Insulin-like growth factor-binding protein 4 OS=Homo sapiens GN=IGFBP4 PE=1 SV=2 - [IBP4_HUMAN]	1,50	0,41
P15529-16	Isoform 3 of Membrane cofactor protein OS=Homo sapiens GN=CD46 - [MCP_HUMAN]	1,41	0,41
P05091	Aldehyde dehydrogenase, mitochondrial OS=Homo sapiens GN=ALDH2 PE=1 SV=2 - [ALDH2_HUMAN]	1,32	0,41
P58335-4	Isoform 4 of Anthrax toxin receptor 2 OS=Homo sapiens GN=ANTXR2 - [ANTR2_HUMAN]	1,36	0,41
Q8IWY9-1	Isoform 1 of Codanin-1 OS=Homo sapiens GN=CDAN1 - [CDAN1_HUMAN]	1,48	0,41
Q6ZUT3-2	Isoform 2 of FERM domain-containing protein 7 OS=Homo sapiens GN=FRMD7 - [FRMD7_HUMAN]	1,20	0,41
Q9UQR0	Sex comb on midleg-like protein 2 OS=Homo sapiens GN=SCML2 PE=1 SV=1 - [SCML2_HUMAN]	1,27	0,41
O95831-3	Isoform 3 of Apoptosis-inducing factor 1, mitochondrial OS=Homo sapiens GN=AIFM1 - [AIFM1_HUMAN]	1,43	0,41
P49959	Double-strand break repair protein MRE11A OS=Homo sapiens GN=MRE11A PE=1 SV=3 - [MRE11_HUMAN]	1,26	0,41
O15344-2	Isoform 2 of Midline-1 OS=Homo sapiens GN=MID1 - [TRI18_HUMAN]	1,54	0,41
P53985	Monocarboxylate transporter 1 OS=Homo sapiens GN=SLC16A1 PE=1 SV=3 - [MOT1_HUMAN]	1,44	0,41
**Gene ID**	**Gene Name**	**Fold Change**	**q-value(%)**
Q9UHT9	Putative uncharacterized protein PRO1768 OS=Homo sapiens GN=PRO1768 PE=5 SV=1 - [YN005_HUMAN]	0,51	0,00
Q96RW7-2	Isoform 2 of Hemicentin-1 OS=Homo sapiens GN=HMCN1 - [HMCN1_HUMAN]	0,58	0,00
P01011-2	Isoform 2 of Alpha-1-antichymotrypsin OS=Homo sapiens GN=SERPINA3 - [AACT_HUMAN]	0,58	0,00
Q9H4M7	Pleckstrin homology domain-containing family A member 4 OS=Homo sapiens GN=PLEKHA4 PE=1 SV=2 - [PKHA4_HU	0,59	0,00
Q9H5V8-2	Isoform 2 of CUB domain-containing protein 1 OS=Homo sapiens GN=CDCP1 - [CDCP1_HUMAN]	0,62	0,00
Q96MK3	Protein FAM20A OS=Homo sapiens GN=FAM20A PE=1 SV=4 - [FA20A_HUMAN]	0,52	0,00
Q92626	Peroxidasin homolog OS=Homo sapiens GN=PXDN PE=1 SV=2 - [PXDN_HUMAN]	0,56	0,00
Q7L311	Armadillo repeat-containing X-linked protein 2 OS=Homo sapiens GN=ARMCX2 PE=2 SV=1 - [ARMX2_HUMAN]	0,54	0,00
P06858	Lipoprotein lipase OS=Homo sapiens GN=LPL PE=1 SV=1 - [LIPL_HUMAN]	0,47	0,00

P98196	Probable phospholipid-transporting ATPase IH OS=Homo sapiens GN=ATP11A PE=2 SV=3 - [AT11A_HUMAN]	0,58	0,00
P14384	Carboxypeptidase M OS=Homo sapiens GN=CPM PE=1 SV=2 - [CBPM_HUMAN]	0,55	0,00
P15907	Beta-galactoside alpha-2,6-sialyltransferase 1 OS=Homo sapiens GN=ST6GAL1 PE=1 SV=1 - [SIAT1_HUMAN]	0,60	0,00
P09493-6	Isoform 6 of Tropomyosin alpha-1 chain OS=Homo sapiens GN=TPM1 - [TPM1_HUMAN]	0,64	0,00
P46013-2	Isoform Short of Antigen KI-67 OS=Homo sapiens GN=MKI67 - [KI67_HUMAN]	0,62	0,00
Q8WY21-3	Isoform 3 of VPS10 domain-containing receptor SorCS1 OS=Homo sapiens GN=SORCS1 - [SORC1_HUMAN]	0,56	0,00
P49448	Glutamate dehydrogenase 2, mitochondrial OS=Homo sapiens GN=GLUD2 PE=1 SV=2 - [DHE4_HUMAN]	0,63	0,00
Q9H568	Actin-like protein 8 OS=Homo sapiens GN=ACTL8 PE=2 SV=1 - [ACTL8_HUMAN]	0,61	0,00
Q96HQ2	CDKN2AIP N-terminal-like protein OS=Homo sapiens GN=CDKN2AIPNL PE=1 SV=1 - [C2AIL_HUMAN]	0,61	0,00
O96020	G1/S-specific cyclin-E2 OS=Homo sapiens GN=CCNE2 PE=1 SV=1 - [CCNE2_HUMAN]	0,66	0,00
P09471-2	Isoform Alpha-2 of Guanine nucleotide-binding protein G(o) subunit alpha OS=Homo sapiens GN=GNAO1 - [GNAO_HU	0,59	0,00
P16112-3	Isoform 3 of Aggrecan core protein OS=Homo sapiens GN=ACAN - [PGCA_HUMAN]	0,57	0,00
Q6ZNJ1-4	Isoform 4 of Neurobeachin-like protein 2 OS=Homo sapiens GN=NBEAL2 - [NBEL2_HUMAN]	0,66	0,00
P24557	Thromboxane-A synthase OS=Homo sapiens GN=TBXAS1 PE=1 SV=3 - [THAS_HUMAN]	0,55	0,00
Q8NI37	Protein phosphatase PTC7 homolog OS=Homo sapiens GN=PPTC7 PE=2 SV=1 - [PPTC7_HUMAN]	0,66	0,00
Q96JQ2	Calmin OS=Homo sapiens GN=CLMN PE=1 SV=1 - [CLMN_HUMAN]	0,59	0,00
Q16610	Extracellular matrix protein 1 OS=Homo sapiens GN=ECM1 PE=1 SV=2 - [ECM1_HUMAN]	0,64	0,00
P48681	Nestin OS=Homo sapiens GN=NES PE=1 SV=2 - [NEST_HUMAN]	0,58	0,00
Q05655	Protein kinase C delta type OS=Homo sapiens GN=PRKCD PE=1 SV=2 - [KPCD_HUMAN]	0,69	0,00
P18065	Insulin-like growth factor-binding protein 2 OS=Homo sapiens GN=IGFBP2 PE=1 SV=2 - [IBP2_HUMAN]	0,56	0,00
Q9Y4Y9-2	Isoform 2 of U6 snRNA-associated Sm-like protein LSm5 OS=Homo sapiens GN=LSM5 - [LSM5_HUMAN]	0,69	0,00
P20336	Ras-related protein Rab-3A OS=Homo sapiens GN=RAB3A PE=1 SV=1 - [RAB3A_HUMAN]	0,64	0,00
Q6EEV4-2	Isoform 5 of DNA-directed RNA polymerase II subunit GRINL1A, isoforms 4/5 OS=Homo sapiens GN=POLR2M - [GL1A	0,52	0,00
Q5VVJ2-2	Isoform 2 of Histone H2A deubiquitinase MYSM1 OS=Homo sapiens GN=MYSM1 - [MYSM1_HUMAN]	0,69	0,00
Q9ULX9	Transcription factor MafF OS=Homo sapiens GN=MAFF PE=1 SV=2 - [MAFF_HUMAN]	0,61	0,00
Q9Y3B1	Protein slowmo homolog 2 OS=Homo sapiens GN=SLMO2 PE=1 SV=2 - [SLMO2_HUMAN]	0,67	0,00
P20265-3	Isoform N-OCT 5B of POU domain, class 3, transcription factor 2 OS=Homo sapiens GN=POU3F2 - [PO3F2_HUMAN]	0,57	0,00
Q86T65	Disheveled-associated activator of morphogenesis 2 OS=Homo sapiens GN=DAAM2 PE=2 SV=3 - [DAAM2_HUMAN]	0,59	0,00
O75600	2-amino-3-ketobutyrate coenzyme A ligase, mitochondrial OS=Homo sapiens GN=GCAT PE=2 SV=1 - [KBL_HUMAN]	0,63	0,00
Q8IVL5	Prolyl 3-hydroxylase 2 OS=Homo sapiens GN=LEPREL1 PE=1 SV=1 - [P3H2_HUMAN]	0,65	0,00
O43293	Death-associated protein kinase 3 OS=Homo sapiens GN=DAPK3 PE=1 SV=1 - [DAPK3_HUMAN]	0,61	0,00
Q9P2F8	Signal-induced proliferation-associated 1-like protein 2 OS=Homo sapiens GN=SIPA1L2 PE=1 SV=2 - [SI1L2_HUMAN]	0,69	0,00
Q8NFZ8	Cell adhesion molecule 4 OS=Homo sapiens GN=CADM4 PE=1 SV=1 - [CADM4_HUMAN]	0,67	0,00

A375 vs. A375VR4

Q1L5Z9	LON peptidase N-terminal domain and RING finger protein 2 OS=Homo sapiens GN=LONRF2 PE=2 SV=3 - [LONF2_HU	0,55	0,00
Q9BUE6	Iron-sulfur cluster assembly 1 homolog, mitochondrial OS=Homo sapiens GN=ISCA1 PE=2 SV=1 - [ISCA1_HUMAN]	0,74	0,00
O00291	Huntingtin-interacting protein 1 OS=Homo sapiens GN=HIP1 PE=1 SV=5 - [HIP1_HUMAN]	0,72	0,00
Q08174-2	Isoform 2 of Protocadherin-1 OS=Homo sapiens GN=PCDH1 - [PCDH1_HUMAN]	0,64	0,00
Q9H246	Uncharacterized protein C1orf21 OS=Homo sapiens GN=C1orf21 PE=1 SV=1 - [CA021_HUMAN]	0,70	0,00
Q96AH8	Ras-related protein Rab-7b OS=Homo sapiens GN=RAB7B PE=2 SV=1 - [RAB7B_HUMAN]	0,58	0,00
O95425	Supervillin OS=Homo sapiens GN=SVIL PE=1 SV=2 - [SVIL_HUMAN]	0,71	0,00
Q14993	Collagen alpha-1(XIX) chain OS=Homo sapiens GN=COL19A1 PE=1 SV=3 - [COJA1_HUMAN]	0,63	0,00
P08294	Extracellular superoxide dismutase [Cu-Zn] OS=Homo sapiens GN=SOD3 PE=1 SV=2 - [SODE_HUMAN]	0,60	0,00
Q96M89	Coiled-coil domain-containing protein 138 OS=Homo sapiens GN=CCDC138 PE=1 SV=1 - [CC138_HUMAN]	0,73	0,00
A4D1P6-2	Isoform 2 of WD repeat-containing protein 91 OS=Homo sapiens GN=WDR91 - [WDR91_HUMAN]	0,67	0,00
O00712	Nuclear factor 1 B-type OS=Homo sapiens GN=NFIB PE=1 SV=2 - [NFIB_HUMAN]	0,68	0,00
O94925	Glutaminase kidney isoform, mitochondrial OS=Homo sapiens GN=GLS PE=1 SV=1 - [GLSK_HUMAN]	0,70	0,00
Q9HA77	Probable cysteine--tRNA ligase, mitochondrial OS=Homo sapiens GN=CARS2 PE=1 SV=1 - [SYCM_HUMAN]	0,68	0,00
Q86XN7	Proline and serine-rich protein 1 OS=Homo sapiens GN=PROSER1 PE=1 SV=2 - [PRSR1_HUMAN]	0,73	0,00
Q14699	Raftlin OS=Homo sapiens GN=RFTN1 PE=1 SV=4 - [RFTN1_HUMAN]	0,69	0,00
O94832	Myosin-Id OS=Homo sapiens GN=MYO1D PE=1 SV=2 - [MYO1D_HUMAN]	0,66	0,00
Q9NS69	Mitochondrial import receptor subunit TOM22 homolog OS=Homo sapiens GN=TOMM22 PE=1 SV=3 - [TOM22_HUM	0,55	0,00
P78395	Melanoma antigen preferentially expressed in tumors OS=Homo sapiens GN=PRAME PE=1 SV=1 - [PRAME_HUMAN]	0,70	0,00
Q8TDW5	Synaptotagmin-like protein 5 OS=Homo sapiens GN=SYTL5 PE=1 SV=1 - [SYTL5_HUMAN]	0,74	0,00
Q14644	Ras GTPase-activating protein 3 OS=Homo sapiens GN=RASA3 PE=1 SV=3 - [RASA3_HUMAN]	0,67	0,00
Q9UL26	Ras-related protein Rab-22A OS=Homo sapiens GN=RAB22A PE=1 SV=2 - [RB22A_HUMAN]	0,73	0,00
Q6IC98	GRAM domain-containing protein 4 OS=Homo sapiens GN=GRAMD4 PE=1 SV=1 - [GRAM4_HUMAN]	0,70	0,00
Q7Z699	Sprouty-related, EVH1 domain-containing protein 1 OS=Homo sapiens GN=SPRED1 PE=1 SV=2 - [SPRE1_HUMAN]	0,71	0,00
Q6PCB6	Abhydrolase domain-containing protein FAM108C1 OS=Homo sapiens GN=FAM108C1 PE=2 SV=2 - [F108C_HUMAN]	0,71	0,00
Q9Y5Z0-3	Isoform 3 of Beta-secretase 2 OS=Homo sapiens GN=BACE2 - [BACE2_HUMAN]	0,67	0,00
Q9NPH3	Interleukin-1 receptor accessory protein OS=Homo sapiens GN=IL1RAP PE=1 SV=2 - [IL1AP_HUMAN]	0,66	0,00
P48735	Isocitrate dehydrogenase [NADP], mitochondrial OS=Homo sapiens GN=IDH2 PE=1 SV=2 - [IDHP_HUMAN]	0,65	0,00
P22681	E3 ubiquitin-protein ligase CBL OS=Homo sapiens GN=CBL PE=1 SV=2 - [CBL_HUMAN]	0,72	0,00
Q5THK1-4	Isoform 4 of Protein PRR14L OS=Homo sapiens GN=PRR14L - [PR14L_HUMAN]	0,74	0,00
P05106-2	Isoform Beta-3B of Integrin beta-3 OS=Homo sapiens GN=ITGB3 - [ITB3_HUMAN]	0,67	0,00
P01023	Alpha-2-macroglobulin OS=Homo sapiens GN=A2M PE=1 SV=3 - [A2MG_HUMAN]	0,66	0,00
Q13813-3	Isoform 3 of Spectrin alpha chain, brain OS=Homo sapiens GN=SPTAN1 - [SPTA2_HUMAN]	0,71	0,00

Q96CG8	Collagen triple helix repeat-containing protein 1 OS=Homo sapiens GN=CTHRC1 PE=1 SV=1 - [CTHR1_HUMAN]	0,67	0,00
Q9BXJ8-2	Isoform 2 of Transmembrane protein 120A OS=Homo sapiens GN=TMEM120A - [T120A_HUMAN]	0,71	0,00
P49006	MARCKS-related protein OS=Homo sapiens GN=MARCKSL1 PE=1 SV=2 - [MRP_HUMAN]	0,74	0,00
P09601	Heme oxygenase 1 OS=Homo sapiens GN=HMOX1 PE=1 SV=1 - [HMOX1_HUMAN]	0,65	0,00
Q05682-3	Isoform 3 of Caldesmon OS=Homo sapiens GN=CALD1 - [CALD1_HUMAN]	0,75	0,00
A6NFQ2-3	Isoform 3 of Protein FAM115C OS=Homo sapiens GN=FAM115C - [F115C_HUMAN]	0,73	0,00
Q13469-2	Isoform B of Nuclear factor of activated T-cells, cytoplasmic 2 OS=Homo sapiens GN=NFATC2 - [NFAC2_HUMAN]	0,64	0,00
Q8TE99-2	Isoform 2 of Acid phosphatase-like protein 2 OS=Homo sapiens GN=ACPL2 - [ACPL2_HUMAN]	0,62	0,00
Q08431-3	Isoform 3 of Lactadherin OS=Homo sapiens GN=MFGE8 - [MFGM_HUMAN]	0,66	0,00
Q8IUW5	RELT-like protein 1 OS=Homo sapiens GN=RELL1 PE=1 SV=1 - [RELL1_HUMAN]	0,74	0,00
P58215	Lysyl oxidase homolog 3 OS=Homo sapiens GN=LOXL3 PE=2 SV=1 - [LOXL3_HUMAN]	0,67	0,00
Q10588	ADP-ribosyl cyclase 2 OS=Homo sapiens GN=BST1 PE=1 SV=2 - [BST1_HUMAN]	0,63	0,00
O75461	Transcription factor E2F6 OS=Homo sapiens GN=E2F6 PE=1 SV=1 - [E2F6_HUMAN]	0,67	0,00
O75190-3	Isoform C of DnaJ homolog subfamily B member 6 OS=Homo sapiens GN=DNAJB6 - [DNJB6_HUMAN]	0,75	0,00
O00462	Beta-mannosidase OS=Homo sapiens GN=MANBA PE=1 SV=3 - [MANBA_HUMAN]	0,74	0,00
Q8WXH0	Nesprin-2 OS=Homo sapiens GN=SYNE2 PE=1 SV=3 - [SYNE2_HUMAN]	0,68	0,00
O94921-3	Isoform 3 of Cyclin-dependent kinase 14 OS=Homo sapiens GN=CDK14 - [CDK14_HUMAN]	0,71	0,00
Q96P11	Putative methyltransferase NSUN5 OS=Homo sapiens GN=NSUN5 PE=1 SV=2 - [NSUN5_HUMAN]	0,73	0,00
Q9Y6Y0	Influenza virus NS1A-binding protein OS=Homo sapiens GN=IVNS1ABP PE=1 SV=3 - [NS1BP_HUMAN]	0,72	0,00
Q3KRA6	UPF0538 protein C2orf76 OS=Homo sapiens GN=C2orf76 PE=2 SV=3 - [CB076_HUMAN]	0,67	0,00
Q9Y3Q8	TSC22 domain family protein 4 OS=Homo sapiens GN=TSC22D4 PE=1 SV=2 - [T22D4_HUMAN]	0,68	0,00
Q04726-2	Isoform 2 of Transducin-like enhancer protein 3 OS=Homo sapiens GN=TLE3 - [TLE3_HUMAN]	0,72	0,00
Q14331	Protein FRG1 OS=Homo sapiens GN=FRG1 PE=1 SV=1 - [FRG1_HUMAN]	0,79	0,00
Q96P53	WD repeat and FYVE domain-containing protein 2 OS=Homo sapiens GN=WDFY2 PE=2 SV=2 - [WDFY2_HUMAN]	0,67	0,00
O95084	Serine protease 23 OS=Homo sapiens GN=PRSS23 PE=1 SV=1 - [PRS23_HUMAN]	0,76	0,00
Q8IWJ2	GRIP and coiled-coil domain-containing protein 2 OS=Homo sapiens GN=GCC2 PE=1 SV=4 - [GCC2_HUMAN]	0,78	0,00
Q9NZV5-2	Isoform 2 of Selenoprotein N OS=Homo sapiens GN=SEPN1 - [SELN_HUMAN]	0,67	0,00
Q9H4W6-2	Isoform Short of Transcription factor COE3 OS=Homo sapiens GN=EBF3 - [COE3_HUMAN]	0,71	0,00
Q14956-2	Isoform 2 of Transmembrane glycoprotein NMB OS=Homo sapiens GN=GPNMB - [GPNMB_HUMAN]	0,64	0,00
Q7Z4L5	Tetratricopeptide repeat protein 21B OS=Homo sapiens GN=TTC21B PE=1 SV=2 - [TT21B_HUMAN]	0,54	0,00
Q9BR77	Coiled-coil domain-containing protein 77 OS=Homo sapiens GN=CCDC77 PE=2 SV=1 - [CCD77_HUMAN]	0,80	0,00
P09543-2	Isoform CNPI of 2',3'-cyclic-nucleotide 3'-phosphodiesterase OS=Homo sapiens GN=CNP - [CN37_HUMAN]	0,73	0,00
Q9Y2D0	Carbonic anhydrase 5B, mitochondrial OS=Homo sapiens GN=CA5B PE=1 SV=1 - [CAH5B_HUMAN]	0,70	0,00

A375 vs. A375VR4

Q8TBP6	Solute carrier family 25 member 40 OS=Homo sapiens GN=SLC25A40 PE=2 SV=1 - [S2540_HUMAN]	0,71	0,00
Q99797	Mitochondrial intermediate peptidase OS=Homo sapiens GN=MIPEP PE=1 SV=2 - [MIPEP_HUMAN]	0,69	0,00
Q9Y666	Solute carrier family 12 member 7 OS=Homo sapiens GN=SLC12A7 PE=1 SV=3 - [S12A7_HUMAN]	0,74	0,00
Q16880	2-hydroxyacylsphingosine 1-beta-galactosyltransferase OS=Homo sapiens GN=UGT8 PE=2 SV=2 - [CGT_HUMAN]	0,67	0,00
P43005	Excitatory amino acid transporter 3 OS=Homo sapiens GN=SLC1A1 PE=1 SV=2 - [EAA3_HUMAN]	0,69	0,00
Q9BWF2	TRAF-interacting protein OS=Homo sapiens GN=TRAIP PE=1 SV=1 - [TRAIP_HUMAN]	0,77	0,00
Q9BXW7-2	Isoform 1 of Cat eye syndrome critical region protein 5 OS=Homo sapiens GN=CECR5 - [CECR5_HUMAN]	0,71	0,00
Q9UPN4-2	Isoform 2 of 5-azacytidine-induced protein 1 OS=Homo sapiens GN=AZI1 - [AZI1_HUMAN]	0,74	0,00
Q13315	Serine-protein kinase ATM OS=Homo sapiens GN=ATM PE=1 SV=3 - [ATM_HUMAN]	0,80	0,00
Q6P2I3	Fumarylacetoacetate hydrolase domain-containing protein 2B OS=Homo sapiens GN=FAHD2B PE=1 SV=1 - [FAH2B_H	0,68	0,00
Q92831	Histone acetyltransferase KAT2B OS=Homo sapiens GN=KAT2B PE=1 SV=3 - [KAT2B_HUMAN]	0,68	0,00
P25325	3-mercaptopyruvate sulfurtransferase OS=Homo sapiens GN=MPST PE=1 SV=3 - [THTM_HUMAN]	0,76	0,00
Q96GE4	Centrosomal protein of 95 kDa OS=Homo sapiens GN=CEP95 PE=1 SV=1 - [CEP95_HUMAN]	0,73	0,00
Q06330-3	Isoform APCR-3 of Recombining binding protein suppressor of hairless OS=Homo sapiens GN=RBPJ - [SUH_HUMAN]	0,80	0,00
P13667	Protein disulfide-isomerase A4 OS=Homo sapiens GN=PDIA4 PE=1 SV=2 - [PDIA4_HUMAN]	0,74	0,00
Q08117	Amino-terminal enhancer of split OS=Homo sapiens GN=AES PE=1 SV=4 - [AES_HUMAN]	0,76	0,00
Q969R8	Integrin-alpha FG-GAP repeat-containing protein 2 OS=Homo sapiens GN=ITFG2 PE=2 SV=1 - [ITFG2_HUMAN]	0,74	0,00
Q8NBN7-2	Isoform 2 of Retinol dehydrogenase 13 OS=Homo sapiens GN=RDH13 - [RDH13_HUMAN]	0,66	0,00
P80404	4-aminobutyrate aminotransferase, mitochondrial OS=Homo sapiens GN=ABAT PE=1 SV=3 - [GABT_HUMAN]	0,68	0,00
P50851-2	Isoform 2 of Lipopolysaccharide-responsive and beige-like anchor protein OS=Homo sapiens GN=LRBA - [LRBA_HUM/	0,55	0,00
Q9UKG9	Peroxisomal carnitine O-octanoyltransferase OS=Homo sapiens GN=CROT PE=1 SV=2 - [OCTC_HUMAN]	0,77	0,00
Q86VN1-2	Isoform 2 of Vacuolar protein-sorting-associated protein 36 OS=Homo sapiens GN=VPS36 - [VPS36_HUMAN]	0,73	0,00
Q8NDD1-3	Isoform 3 of Uncharacterized protein C1orf131 OS=Homo sapiens GN=C1orf131 - [CA131_HUMAN]	0,74	0,00
Q9UBU7	Protein DBF4 homolog A OS=Homo sapiens GN=DBF4 PE=1 SV=1 - [DBF4A_HUMAN]	0,70	0,00
Q9Y4H2	Insulin receptor substrate 2 OS=Homo sapiens GN=IRS2 PE=1 SV=2 - [IRS2_HUMAN]	0,80	0,00
P56693	Transcription factor SOX-10 OS=Homo sapiens GN=SOX10 PE=1 SV=1 - [SOX10_HUMAN]	0,66	0,00
Q06481-4	Isoform 4 of Amyloid-like protein 2 OS=Homo sapiens GN=APLP2 - [APLP2_HUMAN]	0,71	0,00
P57729	Ras-related protein Rab-38 OS=Homo sapiens GN=RAB38 PE=1 SV=1 - [RAB38_HUMAN]	0,73	0,00
Q8N4X5-2	Isoform 2 of Actin filament-associated protein 1-like 2 OS=Homo sapiens GN=AFAP1L2 - [AF1L2_HUMAN]	0,69	0,14
P56282	DNA polymerase epsilon subunit 2 OS=Homo sapiens GN=POLE2 PE=1 SV=2 - [DPOE2_HUMAN]	0,76	0,14
Q92520	Protein FAM3C OS=Homo sapiens GN=FAM3C PE=1 SV=1 - [FAM3C_HUMAN]	0,75	0,14
Q96IR7	4-hydroxyphenylpyruvate dioxygenase-like protein OS=Homo sapiens GN=HPDL PE=1 SV=1 - [HPDL_HUMAN]	0,66	0,14
Q92754	Transcription factor AP-2 gamma OS=Homo sapiens GN=TFAP2C PE=1 SV=1 - [AP2C_HUMAN]	0,70	0,14

A375 vs. A375VR4

Q06033-2	Isoform 2 of Inter-alpha-trypsin inhibitor heavy chain H3 OS=Homo sapiens GN=ITIH3 - [ITIH3_HUMAN]	0,75	0,14
P13612	Integrin alpha-4 OS=Homo sapiens GN=ITGA4 PE=1 SV=3 - [ITA4_HUMAN]	0,73	0,14
O75444	Transcription factor Maf OS=Homo sapiens GN=MAF PE=1 SV=2 - [MAF_HUMAN]	0,67	0,14
Q9Y6N5	Sulfide:quinone oxidoreductase, mitochondrial OS=Homo sapiens GN=SQRDL PE=1 SV=1 - [SQRD_HUMAN]	0,70	0,14
A6NHX0	GATS-like protein 2 OS=Homo sapiens GN=GATSL2 PE=2 SV=3 - [GATL2_HUMAN]	0,73	0,14
Q9UGP4	LIM domain-containing protein 1 OS=Homo sapiens GN=LIMD1 PE=1 SV=1 - [LIMD1_HUMAN]	0,63	0,14
Q8NHP6	Motile sperm domain-containing protein 2 OS=Homo sapiens GN=MOSPD2 PE=1 SV=1 - [MSPD2_HUMAN]	0,73	0,14
Q9H7C4-2	Isoform 2 of Syncoilin OS=Homo sapiens GN=SYNC - [SYNCI_HUMAN]	0,65	0,14
Q9UN79	Transcription factor SOX-13 OS=Homo sapiens GN=SOX13 PE=1 SV=3 - [SOX13_HUMAN]	0,74	0,14
P43007	Neutral amino acid transporter A OS=Homo sapiens GN=SLC1A4 PE=1 SV=1 - [SATT_HUMAN]	0,73	0,14
O15072	A disintegrin and metalloproteinase with thrombospondin motifs 3 OS=Homo sapiens GN=ADAMTS3 PE=2 SV=4 - [AT	0,75	0,14
O75084	Frizzled-7 OS=Homo sapiens GN=FZD7 PE=2 SV=2 - [FZD7_HUMAN]	0,75	0,14
P31323	cAMP-dependent protein kinase type II-beta regulatory subunit OS=Homo sapiens GN=PRKAR2B PE=1 SV=3 - [KAP3_	0,74	0,14
Q9BX66-9	Isoform 9 of Sorbin and SH3 domain-containing protein 1 OS=Homo sapiens GN=SORBS1 - [SRBS1_HUMAN]	0,71	0,14
Q8TED9	Actin filament-associated protein 1-like 1 OS=Homo sapiens GN=AFAP1L1 PE=2 SV=2 - [AF1L1_HUMAN]	0,82	0,14
O00505	Importin subunit alpha-3 OS=Homo sapiens GN=KPNA3 PE=1 SV=2 - [IMA3_HUMAN]	0,79	0,14
Q96F44-3	Isoform 3 of E3 ubiquitin-protein ligase TRIM11 OS=Homo sapiens GN=TRIM11 - [TRI11_HUMAN]	0,76	0,14
Q9Y6M7-2	Isoform 2 of Sodium bicarbonate cotransporter 3 OS=Homo sapiens GN=SLC4A7 - [S4A7_HUMAN]	0,69	0,14
O95427	GPI ethanolamine phosphate transferase 1 OS=Homo sapiens GN=PIGN PE=1 SV=1 - [PIGN_HUMAN]	0,69	0,14
Q6NSZ9-3	Isoform 3 of Zinc finger protein 498 OS=Homo sapiens GN=ZNF498 - [ZN498_HUMAN]	0,77	0,14
Q5JWF2	Guanine nucleotide-binding protein G(s) subunit alpha isoforms XLas OS=Homo sapiens GN=GNAS PE=1 SV=2 - [GNAS	0,74	0,14
Q03001	Dystonin OS=Homo sapiens GN=DST PE=1 SV=4 - [DYST_HUMAN]	0,75	0,14
Q14865-2	Isoform 2 of AT-rich interactive domain-containing protein 5B OS=Homo sapiens GN=ARID5B - [ARI5B_HUMAN]	0,78	0,14
Q7L3S4	Zinc finger protein 771 OS=Homo sapiens GN=ZNF771 PE=1 SV=1 - [ZN771_HUMAN]	0,73	0,14
O43759-2	Isoform 1B of Synaptogyrin-1 OS=Homo sapiens GN=SYNGR1 - [SNG1_HUMAN]	0,74	0,14
Q155Q3-2	Isoform 2 of Dixin OS=Homo sapiens GN=DIXDC1 - [DIXC1_HUMAN]	0,77	0,14
O95677-5	Isoform 5 of Eyes absent homolog 4 OS=Homo sapiens GN=EYA4 - [EYA4_HUMAN]	0,64	0,14
P33992	DNA replication licensing factor MCM5 OS=Homo sapiens GN=MCM5 PE=1 SV=5 - [MCM5_HUMAN]	0,71	0,14
O95235	Kinesin-like protein KIF20A OS=Homo sapiens GN=KIF20A PE=1 SV=1 - [KI20A_HUMAN]	0,72	0,14
Q86VW0	SEC14 domain and spectrin repeat-containing protein 1 OS=Homo sapiens GN=SESTD1 PE=1 SV=2 - [SESD1_HUMAN]	0,65	0,14
Q14186	Transcription factor Dp-1 OS=Homo sapiens GN=TFDP1 PE=1 SV=1 - [TFDP1_HUMAN]	0,73	0,14
Q93062-4	Isoform D of RNA-binding protein with multiple splicing OS=Homo sapiens GN=RBPMS - [RBPMS_HUMAN]	0,76	0,14
P33993	DNA replication licensing factor MCM7 OS=Homo sapiens GN=MCM7 PE=1 SV=4 - [MCM7_HUMAN]	0,74	0,14

A375 vs. A375VR4

A6NHR9	Structural maintenance of chromosomes flexible hinge domain-containing protein 1 OS=Homo sapiens GN=SMCHD1	0,70	0,14
Q96H20-2	Isoform 2 of Vacuolar-sorting protein SNF8 OS=Homo sapiens GN=SNF8 - [SNF8_HUMAN]	0,47	0,14
P26012	Integrin beta-8 OS=Homo sapiens GN=ITGB8 PE=1 SV=1 - [ITB8_HUMAN]	0,73	0,14
Q86X10-2	Isoform 2 of Ral GTPase-activating protein subunit beta OS=Homo sapiens GN=RALGAPB - [RLGPB_HUMAN]	0,77	0,14
O75925	E3 SUMO-protein ligase PIAS1 OS=Homo sapiens GN=PIAS1 PE=1 SV=2 - [PIAS1_HUMAN]	0,81	0,14
O75354-2	Isoform 2 of Ectonucleoside triphosphate diphosphohydrolase 6 OS=Homo sapiens GN=ENTPD6 - [ENTP6_HUMAN]	0,80	0,14
Q8NFV4-4	Isoform 4 of Abhydrolase domain-containing protein 11 OS=Homo sapiens GN=ABHD11 - [ABHDB_HUMAN]	0,75	0,14
Q8NE01	Metal transporter CNNM3 OS=Homo sapiens GN=CNNM3 PE=1 SV=1 - [CNNM3_HUMAN]	0,78	0,14
O75882-3	Isoform 3 of Attractin OS=Homo sapiens GN=ATRN - [ATRN_HUMAN]	0,71	0,14
O75030-10	Isoform M2 of Microphthalmia-associated transcription factor OS=Homo sapiens GN=MITF - [MITF_HUMAN]	0,66	0,14
Q9NQZ5	StAR-related lipid transfer protein 7, mitochondrial OS=Homo sapiens GN=STARD7 PE=1 SV=2 - [STAR7_HUMAN]	0,80	0,14
P35712-4	Isoform 4 of Transcription factor SOX-6 OS=Homo sapiens GN=SOX6 - [SOX6_HUMAN]	0,73	0,14
Q96BS2	Tescalcin OS=Homo sapiens GN=TESC PE=1 SV=3 - [TESC_HUMAN]	0,56	0,21
Q96CN9	GRIP and coiled-coil domain-containing protein 1 OS=Homo sapiens GN=GCC1 PE=1 SV=1 - [GCC1_HUMAN]	0,73	0,21
Q9NZU0	Leucine-rich repeat transmembrane protein FLRT3 OS=Homo sapiens GN=FLRT3 PE=1 SV=1 - [FLRT3_HUMAN]	0,77	0,21
Q8N0W4	Neuroligin-4, X-linked OS=Homo sapiens GN=NLGN4X PE=1 SV=1 - [NLGNX_HUMAN]	0,78	0,21
O95716	Ras-related protein Rab-3D OS=Homo sapiens GN=RAB3D PE=1 SV=1 - [RAB3D_HUMAN]	0,70	0,21
Q9BVL4	Selenoprotein O OS=Homo sapiens GN=SELO PE=2 SV=3 - [SELO_HUMAN]	0,74	0,21
Q92947-2	Isoform Short of Glutaryl-CoA dehydrogenase, mitochondrial OS=Homo sapiens GN=GCDH - [GCDH_HUMAN]	0,75	0,21
Q96HD1	Cysteine-rich with EGF-like domain protein 1 OS=Homo sapiens GN=CRELD1 PE=1 SV=3 - [CREL1_HUMAN]	0,75	0,21
Q14746	Conserved oligomeric Golgi complex subunit 2 OS=Homo sapiens GN=COG2 PE=1 SV=1 - [COG2_HUMAN]	0,75	0,21
Q96ND0	Protein FAM210A OS=Homo sapiens GN=FAM210A PE=2 SV=2 - [F210A_HUMAN]	0,69	0,21
Q9BZF3-2	Isoform 2 of Oxysterol-binding protein-related protein 6 OS=Homo sapiens GN=OSBPL6 - [OSBL6_HUMAN]	0,73	0,21
Q9HBA0-4	Isoform 4 of Transient receptor potential cation channel subfamily V member 4 OS=Homo sapiens GN=TRPV4 - [TRPV	0,71	0,21
Q7L5N7	Lysophosphatidylcholine acyltransferase 2 OS=Homo sapiens GN=LPCAT2 PE=1 SV=1 - [PCAT2_HUMAN]	0,74	0,21
Q15014	Mortality factor 4-like protein 2 OS=Homo sapiens GN=MORF4L2 PE=1 SV=1 - [MO4L2_HUMAN]	0,77	0,21
Q9NUT2-2	Isoform Short of ATP-binding cassette sub-family B member 8, mitochondrial OS=Homo sapiens GN=ABCB8 - [ABCB8	0,72	0,21
Q9UHK0	Nuclear fragile X mental retardation-interacting protein 1 OS=Homo sapiens GN=NUFIP1 PE=1 SV=2 - [NUFP1_HUMA	0,75	0,21
P33121-2	Isoform 2 of Long-chain-fatty-acid--CoA ligase 1 OS=Homo sapiens GN=ACSL1 - [ACSL1_HUMAN]	0,71	0,21
P42356	Phosphatidylinositol 4-kinase alpha OS=Homo sapiens GN=PI4KA PE=1 SV=3 - [PI4KA_HUMAN]	0,75	0,21
P42765	3-ketoacyl-CoA thiolase, mitochondrial OS=Homo sapiens GN=ACAA2 PE=1 SV=2 - [THIM_HUMAN]	0,73	0,21
Q4KMQ1	Taperin OS=Homo sapiens GN=TPRN PE=2 SV=2 - [TPRN_HUMAN]	0,78	0,21
Q8N474	Secreted frizzled-related protein 1 OS=Homo sapiens GN=SFRP1 PE=1 SV=1 - [SFRP1_HUMAN]	0,78	0,21

Q99595	Mitochondrial import inner membrane translocase subunit Tim17-A OS=Homo sapiens GN=TIMM17A PE=1 SV=1 - [TI	0,65	0,21
O75147-2	Isoform 2 of Obscurin-like protein 1 OS=Homo sapiens GN=OBSL1 - [OBSL1_HUMAN]	0,80	0,21
Q9P0W2-3	Isoform 3 of SWI/SNF-related matrix-associated actin-dependent regulator of chromatin subfamily E member 1-relat	0,79	0,21
P55809	Succinyl-CoA:3-ketoacid-coenzyme A transferase 1, mitochondrial OS=Homo sapiens GN=OXCT1 PE=1 SV=1 - [SCOT1_	0,75	0,21
Q5TFQ8	Signal-regulatory protein beta-1 isoform 3 OS=Homo sapiens GN=SIRPB1 PE=1 SV=1 - [SIRBL_HUMAN]	0,73	0,21
P39880-4	Isoform 5 of Homeobox protein cut-like 1 OS=Homo sapiens GN=CUX1 - [CUX1_HUMAN]	0,72	0,21
Q9UDR5	Alpha-aminoadipic semialdehyde synthase, mitochondrial OS=Homo sapiens GN=AASS PE=1 SV=1 - [AASS_HUMAN]	0,74	0,21
Q96GY3	Protein lin-37 homolog OS=Homo sapiens GN=LIN37 PE=1 SV=1 - [LIN37_HUMAN]	0,81	0,21
Q8N9F0	N-acetylaspartate synthetase OS=Homo sapiens GN=NAT8L PE=1 SV=3 - [NAT8L_HUMAN]	0,70	0,21
Q8IW92	Beta-galactosidase-1-like protein 2 OS=Homo sapiens GN=GLB1L2 PE=2 SV=1 - [GLBL2_HUMAN]	0,72	0,21
Q9Y2S0-2	Isoform 2 of DNA-directed RNA polymerases I and III subunit RPAC2 OS=Homo sapiens GN=POLR1D - [RPAC2_HUMAI	0,76	0,21
Q99674	Cell growth regulator with EF hand domain protein 1 OS=Homo sapiens GN=CGREF1 PE=2 SV=2 - [CGRE1_HUMAN]	0,75	0,21
Q7Z2Z1-2	Isoform 2 of Treslin OS=Homo sapiens GN=TICRR - [TICRR_HUMAN]	0,75	0,21
Q96RF0-2	Isoform 2 of Sorting nexin-18 OS=Homo sapiens GN=SNX18 - [SNX18_HUMAN]	0,69	0,21
Q9UJA3-2	Isoform 2 of DNA replication licensing factor MCM8 OS=Homo sapiens GN=MCM8 - [MCM8_HUMAN]	0,74	0,21
P52789	Hexokinase-2 OS=Homo sapiens GN=HK2 PE=1 SV=2 - [HXK2_HUMAN]	0,76	0,21
Q7Z7M9	Polypeptide N-acetylgalactosaminyltransferase 5 OS=Homo sapiens GN=GALNT5 PE=1 SV=1 - [GALT5_HUMAN]	0,69	0,21
Q86YB8	ERO1-like protein beta OS=Homo sapiens GN=ERO1LB PE=1 SV=2 - [ERO1B_HUMAN]	0,78	0,21
Q9Y255	PRELI domain-containing protein 1, mitochondrial OS=Homo sapiens GN=PRELID1 PE=2 SV=1 - [PRLD1_HUMAN]	0,81	0,21
O60861-2	Isoform 2 of Growth arrest-specific protein 7 OS=Homo sapiens GN=GAS7 - [GAS7_HUMAN]	0,63	0,21
Q3V6T2-4	Isoform 4 of Girdin OS=Homo sapiens GN=CCDC88A - [GRDN_HUMAN]	0,78	0,21
Q8WTV0-3	Isoform 2 of Scavenger receptor class B member 1 OS=Homo sapiens GN=SCARB1 - [SCRB1_HUMAN]	0,72	0,21
O00463	TNF receptor-associated factor 5 OS=Homo sapiens GN=TRAF5 PE=1 SV=2 - [TRAF5_HUMAN]	0,77	0,21
P27144	Adenylate kinase isoenzyme 4, mitochondrial OS=Homo sapiens GN=AK4 PE=1 SV=1 - [KAD4_HUMAN]	0,78	0,21
Q9H857-4	Isoform 4 of 5'-nucleotidase domain-containing protein 2 OS=Homo sapiens GN=NT5DC2 - [NT5D2_HUMAN]	0,69	0,21
O60568	Procollagen-lysine,2-oxoglutarate 5-dioxygenase 3 OS=Homo sapiens GN=PLOD3 PE=1 SV=1 - [PLOD3_HUMAN]	0,72	0,21
Q9H425	Uncharacterized protein C1orf198 OS=Homo sapiens GN=C1orf198 PE=1 SV=1 - [CA198_HUMAN]	0,76	0,21
Q8NFG4	Folliculin OS=Homo sapiens GN=FLCN PE=1 SV=1 - [FLCN_HUMAN]	0,77	0,21
O15235	28S ribosomal protein S12, mitochondrial OS=Homo sapiens GN=MRPS12 PE=1 SV=1 - [RT12_HUMAN]	0,82	0,21
Q9NUA8	Zinc finger and BTB domain-containing protein 40 OS=Homo sapiens GN=ZBTB40 PE=1 SV=4 - [ZBT40_HUMAN]	0,71	0,21
Q96CN5	Leucine-rich repeat-containing protein 45 OS=Homo sapiens GN=LRRC45 PE=2 SV=1 - [LRC45_HUMAN]	0,56	0,21
P30038	Delta-1-pyrroline-5-carboxylate dehydrogenase, mitochondrial OS=Homo sapiens GN=ALDH4A1 PE=1 SV=3 - [AL4A1_	0,78	0,21
Q8IWF2	FAD-dependent oxidoreductase domain-containing protein 2 OS=Homo sapiens GN=FOXRED2 PE=1 SV=1 - [FXRD2_H	0,77	0,21

A375 vs. A375VR4

Q2M1Z3	Rho GTPase-activating protein 31 OS=Homo sapiens GN=ARHGAP31 PE=1 SV=2 - [RHG31_HUMAN]	0,67	0,21
O60266	Adenylate cyclase type 3 OS=Homo sapiens GN=ADCY3 PE=1 SV=3 - [ADCY3_HUMAN]	0,72	0,21
Q14643-4	Isoform 4 of Inositol 1,4,5-trisphosphate receptor type 1 OS=Homo sapiens GN=ITPR1 - [ITPR1_HUMAN]	0,78	0,21
Q6NT16	MFS-type transporter C6orf192 OS=Homo sapiens GN=C6orf192 PE=2 SV=1 - [CF192_HUMAN]	0,73	0,21
Q16363-2	Isoform 2 of Laminin subunit alpha-4 OS=Homo sapiens GN=LAMA4 - [LAMA4_HUMAN]	0,76	0,21
P17483	Homeobox protein Hox-B4 OS=Homo sapiens GN=HOXB4 PE=1 SV=2 - [HXB4_HUMAN]	0,77	0,21
Q9P2N6-8	Isoform 8 of Uncharacterized protein KIAA1310 OS=Homo sapiens GN=KIAA1310 - [K1310_HUMAN]	0,77	0,21
P78312-4	Isoform 4 of Protein FAM193A OS=Homo sapiens GN=FAM193A - [F193A_HUMAN]	0,71	0,21
Q9UKX7-2	Isoform 2 of Nuclear pore complex protein Nup50 OS=Homo sapiens GN=NUP50 - [NUP50_HUMAN]	0,77	0,21
Q8IXJ9-2	Isoform 2 of Putative Polycomb group protein ASXL1 OS=Homo sapiens GN=ASXL1 - [ASXL1_HUMAN]	0,79	0,41
Q9NZ71-5	Isoform 5 of Regulator of telomere elongation helicase 1 OS=Homo sapiens GN=RTEL1 - [RTEL1_HUMAN]	0,78	0,41
Q8N9F7-3	Isoform 3 of Glycerophosphodiester phosphodiesterase domain-containing protein 1 OS=Homo sapiens GN=GDPD1 - [illegible]	0,75	0,41
Q9H6R3	Acyl-CoA synthetase short-chain family member 3, mitochondrial OS=Homo sapiens GN=ACSS3 PE=2 SV=1 - [ACSS3_[illegible]	0,72	0,41
Q99538	Legumain OS=Homo sapiens GN=LGMN PE=1 SV=1 - [LGMN_HUMAN]	0,83	0,41
P48729	Casein kinase I isoform alpha OS=Homo sapiens GN=CSNK1A1 PE=1 SV=2 - [KC1A_HUMAN]	0,79	0,41
Q8IZM8	Zinc finger protein 654 OS=Homo sapiens GN=ZNF654 PE=1 SV=3 - [ZN654_HUMAN]	0,81	0,41
P16885	1-phosphatidylinositol-4,5-bisphosphate phosphodiesterase gamma-2 OS=Homo sapiens GN=PLCG2 PE=1 SV=4 - [PL[illegible]	0,70	0,41
P59768	Guanine nucleotide-binding protein G(I)/G(S)/G(O) subunit gamma-2 OS=Homo sapiens GN=GNG2 PE=1 SV=2 - [GBG[illegible]	0,75	0,41
Q96MW5	Conserved oligomeric Golgi complex subunit 8 OS=Homo sapiens GN=COG8 PE=1 SV=2 - [COG8_HUMAN]	0,80	0,41
O15164-2	Isoform Short of Transcription intermediary factor 1-alpha OS=Homo sapiens GN=TRIM24 - [TIF1A_HUMAN]	0,76	0,41
P35711-2	Isoform 2 of Transcription factor SOX-5 OS=Homo sapiens GN=SOX5 - [SOX5_HUMAN]	0,83	0,41
Q9NR19	Acetyl-coenzyme A synthetase, cytoplasmic OS=Homo sapiens GN=ACSS2 PE=1 SV=1 - [ACSA_HUMAN]	0,57	0,41
O94782	Ubiquitin carboxyl-terminal hydrolase 1 OS=Homo sapiens GN=USP1 PE=1 SV=1 - [UBP1_HUMAN]	0,81	0,41
Q9HCN8	Stromal cell-derived factor 2-like protein 1 OS=Homo sapiens GN=SDF2L1 PE=1 SV=2 - [SDF2L_HUMAN]	0,72	0,41
O95208-2	Isoform 2 of Epsin-2 OS=Homo sapiens GN=EPN2 - [EPN2_HUMAN]	0,79	0,41
Q9Y248	DNA replication complex GINS protein PSF2 OS=Homo sapiens GN=GINS2 PE=1 SV=1 - [PSF2_HUMAN]	0,64	0,41
A0MZ66-5	Isoform 5 of Shootin-1 OS=Homo sapiens GN=KIAA1598 - [SHOT1_HUMAN]	0,62	0,41
B2RTY4-3	Isoform 3 of Myosin-IXa OS=Homo sapiens GN=MYO9A - [MYO9A_HUMAN]	0,68	0,41
P56377	AP-1 complex subunit sigma-2 OS=Homo sapiens GN=AP1S2 PE=1 SV=1 - [AP1S2_HUMAN]	0,83	0,41
P36897	TGF-beta receptor type-1 OS=Homo sapiens GN=TGFBR1 PE=1 SV=1 - [TGFR1_HUMAN]	0,69	0,41
O43709	Uncharacterized methyltransferase WBSCR22 OS=Homo sapiens GN=WBSCR22 PE=1 SV=2 - [WBS22_HUMAN]	0,84	0,41
Q9Y2G9-3	Isoform 2 of Protein strawberry notch homolog 2 OS=Homo sapiens GN=SBNO2 - [SBNO2_HUMAN]	0,83	0,41
Q9Y2V7-2	Isoform 2 of Conserved oligomeric Golgi complex subunit 6 OS=Homo sapiens GN=COG6 - [COG6_HUMAN]	0,76	0,41

A375 vs. A375VR4

P10244	Myb-related protein B OS=Homo sapiens GN=MYBL2 PE=1 SV=1 - [MYBB_HUMAN]	0,65	0,41
Q9NS91	E3 ubiquitin-protein ligase RAD18 OS=Homo sapiens GN=RAD18 PE=1 SV=2 - [RAD18_HUMAN]	0,79	0,41
Q92544	Transmembrane 9 superfamily member 4 OS=Homo sapiens GN=TM9SF4 PE=1 SV=2 - [TM9S4_HUMAN]	0,77	0,41
Q5M7Z0-2	Isoform 2 of RING finger and transmembrane domain-containing protein 1 OS=Homo sapiens GN=RNFT1 - [RNFT1_HU	0,80	0,41
Q9GZV5	WW domain-containing transcription regulator protein 1 OS=Homo sapiens GN=WWTR1 PE=1 SV=1 - [WWTR1_HUM	0,80	0,41
Q6UXH1-4	Isoform 4 of Cysteine-rich with EGF-like domain protein 2 OS=Homo sapiens GN=CRELD2 - [CREL2_HUMAN]	0,79	0,41
Q9UBZ4	DNA-(apurinic or apyrimidinic site) lyase 2 OS=Homo sapiens GN=APEX2 PE=1 SV=1 - [APEX2_HUMAN]	0,82	0,41
P13686	Tartrate-resistant acid phosphatase type 5 OS=Homo sapiens GN=ACP5 PE=1 SV=3 - [PPA5_HUMAN]	0,76	0,41
Q9ULM3	YEATS domain-containing protein 2 OS=Homo sapiens GN=YEATS2 PE=1 SV=2 - [YETS2_HUMAN]	0,76	0,41
Q6PCB5	Round spermatid basic protein 1-like protein OS=Homo sapiens GN=RSBN1L PE=1 SV=2 - [RSBNL_HUMAN]	0,76	0,41
O43865	Putative adenosylhomocysteinase 2 OS=Homo sapiens GN=AHCYL1 PE=1 SV=2 - [SAHH2_HUMAN]	0,79	0,41
Q6PIW4-2	Isoform 2 of Fidgetin-like protein 1 OS=Homo sapiens GN=FIGNL1 - [FIGL1_HUMAN]	0,83	0,41
Q6PJW8	Consortin OS=Homo sapiens GN=CNST PE=2 SV=3 - [CNST_HUMAN]	0,79	0,41
Q71F23	Centromere protein U OS=Homo sapiens GN=MLF1IP PE=1 SV=1 - [CENPU_HUMAN]	0,76	0,41
