# Supplementary material for: Silencing FLI or targeting CD13/ANPEP lead to dephosphorylation of EPHA2, a mediator of BRAF inhibitor resistance, and induce growth arrest or apoptosis in melanoma cells
Source: Cell Death Dis. 2017 Aug 31;8(8):e3029–. doi: 10.1038/cddis.2017.406 (PMC5596587; doi:10.1038/cddis.2017.406)
Supplement: Supplementary Table S3 [file cddis2017406x4.pdf]

Supplementary Table S3. Differentially expressed protein classes in the BRAFi resistant daughter cell lines compared to parental A375

**Differentially expressed protein classes**	
1	extracellular matrix protein (PC00102)
2	protease (PC00190)
3	cytoskeletal protein (PC00085)
4	transporter (PC00227)
	transmembrane receptor
5	regulatory/adaptor protein (PC00226)
6	transferase (PC00220)
7	oxidoreductase (PC00176)
8	lyase (PC00144)
9	cell adhesion molecule (PC00069)
10	ligase (PC00142)
11	nucleic acid binding (PC00171)
12	signaling molecule (PC00207)
13	enzyme modulator (PC00095)
14	calcium-binding protein (PC00060)
15	defense/immunity protein (PC00090)
16	hydrolase (PC00121)
17	transfer/carrier protein (PC00219)
18	membrane traffic protein (PC00150)
19	phosphatase (PC00181)
20	transcription factor (PC00218)
21	chaperone (PC00072)
22	cell junction protein (PC00070)
23	surfactant (PC00212)
24	structural protein (PC00211)
25	kinase (PC00137)
26	storage protein (PC00210)
27	receptor (PC00197)
28	isomerase (PC00135)
