# Supplementary material for: Silencing FLI or targeting CD13/ANPEP lead to dephosphorylation of EPHA2, a mediator of BRAF inhibitor resistance, and induce growth arrest or apoptosis in melanoma cells
Source: Cell Death Dis. 2017 Aug 31;8(8):e3029–. doi: 10.1038/cddis.2017.406 (PMC5596587; doi:10.1038/cddis.2017.406)
Supplement: Supplementary Table S4 [file cddis2017406x5.pdf]

Supplementary Table S4. EPHA2 gene copy number analysis of the A375 cell line and the resistant sublines was performed by Taqman assay in four replicates.

Cell line	CNA value	Average	Number
Parental A375	3,26	3,5825	3
Parental A375	3,39		3
Parental A375	3,77		4
Parental A375	3,91		4
A375PR1	3,82	3,645	4
A375PR1	3,6		4
A375PR1	3,72		4
A375PR1	3,44		3
A375VR3	5,9	4,34	6
A375VR3	4,25		4
A375VR3	3,44		3
A375VR3	3,77		4
A375VR4	3,61	3,7425	4
A375VR4	3,54		4
A375VR4	3,47		3
A375VR4	4,35		4
