# Supplementary material for: Silencing FLI or targeting CD13/ANPEP lead to dephosphorylation of EPHA2, a mediator of BRAF inhibitor resistance, and induce growth arrest or apoptosis in melanoma cells
Source: Cell Death Dis. 2017 Aug 31;8(8):e3029–. doi: 10.1038/cddis.2017.406 (PMC5596587; doi:10.1038/cddis.2017.406)
Supplement: Supplementary Table S5 [file cddis2017406x6.pdf]

Supplementary table S5. Primers used in the study for Sanger sequencing and qPCR.

Gene, primer	5' to 3´primers sequence	Reference sequence	Bases in sequence	Bases in GRCh38 assembly	Amplicon (gDNA)	PCR method
NRASex1-F	GCTCGCCAATTAACCCTGAT	NG_007572.1	682-701	Chr1:114716194-114716213	213 bp	58,BE
NRASex1-R	CGACAAGTGAGAGACAGGATCA	NG_007572.1	873-894	Chr1:114716022-114716001		
NRASex2-F	GGGCTTGAATAGTTAGATGCT	NG_007572.1	2802-2822	Chr1:114714093-114714073	543 bp	58,BE
NRASex2-R	GGTTCCAAGTCATTCCCAGTA	NG_007572.1	3324-3344	Chr1:114713571-114713551		
NRASex3-F	GCTGAGATTGCAGGCATGA	NG_007572.1	7087-7105	Chr1:114709790-114709808	285 bp	58,BE
NRASx3-R	CAACTGATGCAAACTCTTGCACA	NG_007572.1	7349-7371	Chr1:114709524-114709546		
KRASex1-F	GTCACATTTTCATTATTTTTATTATAA	NC_000012.12	5509-5535	Chr12:25245397-25245423	155 bp	60,NA
KRASex1-R	TTTACCTCTATTGTTGGATCATATTC	NC_000012.12	5638-5663	Chr12:25245269-25245294		
KRASex2-F	GGTGCACTGTAATAATCCAGACTGT	NC_000012.12	23479-23503	Chr12:25227429-25227453	301 bp	60,NA
KRASex2-R	GCATGGCATTAGCAAAGACTCA	NC_000012.12	23758-23779	Chr12:25227153-25227174		
KRASex3-F	GATATTTGTGTTACTAATGACTGTGCTATA	NC_000012.12	25110-25139	Chr12:25225793-25225822	211 bp	60,NA
KRASex3-R	ACCTGTCTTGTCTTTGCTGAT	NC_000012.12	25300-25320	Chr12:25225612-25225632		
RRASex1-F	GCGAAGGCAGCAGCAGCGGT	NC_000019.10	20-39	Chr19:49640105-49640124	201 bp	58,BE
RRASex1-R	CTCCCGGGTGAGGGCCCACTA	NC_000019.10	200-220	Chr19:49639924-49639944		
RRASex2-F	GTCTGCCGGGGCACTGCAGTA	NC_000019.10	2969-2989	Chr19:49637155-49637175	173 bp	58,BE
RRASex2-R	GACACCACCCCCATCCATCAT	NC_000019.10	3121-3141	Chr19:49637003-49637023		
RRASex3-F	GGAGCCTGGTCCCCACGAT	NC_000019.10	3176-3194	Chr19:49636950-49636968	201bp	58,BE
RRASex3-R	GAGCCCAGGTCCTCCCCACA	NC_000019.10	3357-3376	Chr19:49636768-49636787		

RRASex4-F	CCTCATGCCTCCGGCTTCACT	NC_000019.10	3391-3411	Chr19:49636733-49636753	189 bp	58,BE
RRASex4-R	GGAAGGAGCCTCCCAGACTGAGAT	NC_000019.10	3556-3579	Chr19:49636565-49636588		
RRASex5_6-F	CCTGAGTGCAGGGACCTGACT	NC_000019.10	4247-4267	Chr19:49635877-49635897	374 bp	58,BE
RRASex5_6-R	CGAAGGCAGCTAGTCCCGAGAGCT	NC_000019.10	4597-4620	Chr19:49635524-49635547		
PTENex1-F	GCAGCTTCTGCCATCTCTCT	NG_007466.2	980-999	Chr10:87864418-87864437	200 bp	63,BE
PTENex1-R	CATCCGTCTACTCCCACGTT	NG_007466.2	1160-1179	Chr10:87864598-87864617		
PTENex3-F	GTGGGGAAAACTTTCTTTTCA	NG_007466.2	30482-30502	Chr10:87893920-87893940	282 bp	63,BE
PTENex3-R	TCTTTTTCTGTGGCTTAGAAATCTT	NG_007466.2	30739-30763	Chr10:87894177-87894201		
PTENex4-F	AATCTGTCTTTTGGTTTTTCTTGA	NG_007466.2	61977-62000	Chr10:87925415-87925438	233 bp	62,BE
PTENex4-R	ACTCTACCTCACTCTAACAAGCAGA	NG_007466.2	62185-62209	Chr10:87925623-87925647		
PTENex5-F	AAAGATTCAGGCAATGTTTGTT	NG_007466.2	67516-67537	Chr10:87930954-87930975	235bp	62,BE
PTENex5-R	TCTCACTCGATAATCTGGATGAC	NG_007466.2	67728-67750	Chr10:87931166-87931188		
PTENex6i-F	GGAATCCAGTGTTTCTTTTAAATACC	NG_007466.2	69452-69477	Chr10:87932890-87932915	261 bp	62,BE
PTENex6i-R	TTCGTCCCTTTCCAGCTTTA	NG_007466.2	69693-69712	Chr10:87933131-87933150		
PTENex6ii-F	TGAAGATCTTGACCAATGGCT	NG_007466.2	69636-69656	Chr10:87933074-87933094	222 bp	63,BE
PTENex6ii-R	CAGATCCAGGAAGAGGAAAGG	NG_007466.2	69837-69857	Chr10:87933275-87933295		
PTENex10-F	GGCTACGACCCAGTTACCAT	NG_007466.2	88588-88607	Chr10:87952026-87952045	271 bp	62,BE
PTENex10-R	TCCAATACATGGAAGGATGAGA	NG_007466.2	88837-88858	Chr10:87952275-87952296		
PTENex11-F	CAGTTAAAGGCATTTCCTGTG	NG_007466.2	94363-94383	Chr10:87957801-87957821	262 bp	62,BE
PTENex11-R	TCCTTATTTTGGATATTTCTCCC	NG_007466.2	94602-94624	Chr10:87958040-87958062		

PTENex12-F	GGACAAAATGTTTCACTTTTGGGTA	NG_007466.2	97455-97479	Chr10:87960893-87960917	260 bp	62,BE
PTENex12-R	CAACAACCCCCACAAAATGT	NG_007466.2	97695-97714	Chr10:87961133-87961152		
PTENex13-F	GATGAGTCATATTTGTGGGTTTTC	NG_007466.2	101803-101826	Chr10:87965241-87965264	272 bp	62,BE
PTENex13-R	TTATTTTCATGGTGTTTTATCCC	NG_007466.2	102052-102074	Chr10:87965490-87965512		
CDKN2Aex1a-F	GAAGAAAGAGGAGGGGCTG	NC_000009.11	19561-19579	Chr9:21974913-21974931	340 bp	60,BE
CDKN2Aex1a-R	GCGCTACCTGATTCCAATTC	NC_000009.11	19881-19900	Chr9:21974592-21974611		
CDKN2Aex1b-F	GCGCTGAGGGTGGGAAGAT	NC_000009.12	21994706-21994724	Chr9:21994706-21994724	678 bp	56,PCRx
CDKN2Aex1b-R	GCCTGGGCTAGAGACGAATTATCTG	NC_000009.12	21994071-21994047	Chr9:21994071-21994047		
CDKN2Aex2-F	GGAAATTGGAAACTGGAAGC	NC_000009.11	23134-23153	Chr9:21971339-21971358	574 bp	59,BE
CDKN2Aex2-R	GTGAGCTGAGGCAAGACC	NC_000009.11	23690-23707	Chr9:21970785-21970802		
CDKN2Aex3-F	CAGGCGGGCAGTGGACTAGC	NC_000009.11	26051-26070	Chr9:21968422-21968441	319 bp	60,DM
CDKN2Aex3-R	CTACGAAAGCGGGGTGGGTTGTG	NC_000009.11	26347-26369	Chr9:21968123-21968145		
CDK4-F	GGTGATAGGAGTCTGTGATTGTA	NG_007484.2	688-710	Chr12:57751738-57751760	213 bp	62,NA
CDK4-R	ACCTCACGAACTGTGCTGAT	NG_007484.2	881-900	Chr12:57751548-57751567		
MC1R-F	GGTCCAGAGGTGTCGAAAT	NG_012026.1	1044-1062	Chr16:89918922-89918940	1324 bp	58,BE
MC1R-R	TGCCCAGCACACTTAAAGC	NG_012026.1	2349-2367	Chr16:89920227-89920245		
MC1R-S1, seq primer	CCATCTCTGACGGGCTCT	NG_012026.1	1496-1513	Chr16:89919374-89919391	NA	58,BE
MC1R-S3, seq primer	TCCAGCCTCTGCTTCCTG	NG_012026.1	1769-1785	Chr16:89919646-89919663	NA	
RAC1ex1F	CCGCCGCTTCCTATCTCA	NG_029431.1	184-201	Chr7:6374678-6374695	227 bp	58,BE
RAC1ex1R	CCGCGACACAGGCCCA	NG_029431.1	395-410	Chr7: 6374904-6374889		
RAC1ex2F	GTGAAAGCTAAGATTACAT	NG_029431.1	12660-12679	Chr7:6387154-6387172	231 bp	58,BE
RAC1ex2R	GTTCATATGAGGATTAAAGATGGCT	NG_029431.1	12866-12890	Chr7:6387360-6387384		

RAC1ex3F	CTAGGATGGCTGGGACAGT	NG_029431.1	17363-17381	Chr7: 6391857-6391875	271 bp	58,BE
RAC1ex3R	GCAAGTCCATAGGTAACATGCACT	NG_029431.1	17610-17633	Chr7: 6392104-6392127		
RAC1ex4F	GTTGTCCTCAGTCTGTACTAAACTCA	NG_029431.1	24118-24143	Chr7: 6398612-6398637	209 bp	58,BE
RAC1ex4R	CACTCAAGGAGCTTGAGGCT	NG_029431.1	24307-24326	Chr7: 6398801-6398820		
RAC1ex5F	GTAGAAAGCAAAGTGCATGCTTCA	NG_029431.1	25562-25585	Chr7: 6400056-6400079	183 bp	58,BE
RAC1ex5R	CTGAGAGGATCATTCTATAACTTACAT	NG_029431.1	25718-25744	Chr7: 6400238-6400212		
RAC1ex6F	GTGATTTAGGTGAAGGACATCTGTA	NG_029431.1	27309-27333	Chr7: 6401803-6401827	291 bp	58,BE
RAC1ex6R	GGACTCCAGTCTCTGTCACTACAAT	NG_029431.1	27575-27599	Chr7: 6402093-6402069		
RAC1ex7F	GGTGGTGTGATCAGAAGAGAGT	NG_029431.1	27759-27780	Chr7: 6402253-6402274	226 bp	58,BE
RAC1ex7R	GGGACAGGACCAAGAACGA	NG_029431.1	27966-27984	Chr7: 6402478-6402460		
METex1F	CTGAACTGCTCTCGCCTTGA	NG_008996.1	26633-26652	Chr7: 116699037-116699056	703 bp	58,BE
METex1intR	GCCTTCTCACTGATATCGAATGCA	NG_008996.1	27312-27335	Chr7: 116699739-116699716		
METex1intF	CCGGTTCATCAACTTCTTTGTA	NG_008996.1	27250-27271	Chr7: 116699654-116699675	713 bp	58,BE
METex1R	GCCCTGGATATTCTTTTTGAGACT	NG_008996.1	27939-27962	Chr7: 116700366-116700343		
METex2F	CAGGCTCTGAAAATACACACTGA	NG_008996.1	59176-59198	Chr7: 116731580-116731602	411 bp	58,BE
METex2R	CAGTCTGTGTTCCAGTTTCTACTATGAT	NG_008996.1	59559-59586	Chr7: 116731990-116731963		
METex3F	ACTGAGCTTGTTGGAATAAGGAT	NG_008996.1	67500-67522	Chr7: 116739904-116739926	251 bp	58,BE
METex3R	GTGACACTGGTTGTAAATATGCAT	NG_008996.1	67727-67750	Chr7: 116740154-116740131		
METex16F	CACTGTTCCATAATGAAGTTAATGTCT	NG_008996.1	106326-106352	Chr7: 116778730-116778756	336 bp	58,BE
METex16R	TGTGCATCTTTGGCTACTAGCT	NG_008996.1	106640-106661	Chr7: 116779065-116779044		
METex17F	GGTCAAAATTAGAACAGTAGATGCTTA	NG_008996.1	109525-109551	Chr7: 116781929-116781955	229 bp	58,BE

METex17R	GGAAACAGATTCCTCCTTGTCACT	NG_008996.1	109730-109753	Chr7: 116782157-116782134		
METex18F	GGATTTCAAATACTGAAGCCACT	NG_008996.1	110798-110820	Chr7: 116783202-116783224	418 bp	58,BE
METex18R	GTACGTATCAGACAAGTCCATTTTTACA T	NG_008996.1	111187-111215	Chr7: 116783619-116783591		
METex19_20F	ATGTATGGTCACATCTCTCACCTCA	NG_008996.1	123201-123225	Chr7: 116795605-116795629	564 bp	58,BE
METex19_20R	CCATTGGACAAAGTGTGGACTGT	NG_008996.1	123742-123764	Chr7: 11679614-116796168		
**Expression primers used in the study**						
**Primer**	**Sequence 5' to 3'**	**Ref sequence**	**Bases**	**Coding transcripts**	**Amplicon size**	**PCR (A_{temp}, mix)**
EPHA2cDNA-F	CCGCAAGAAGGGAGACTCCA	NM_004431.3	c.1419-1438	NM_004431	164 bp	60, Max
EPHA2cDNA-R	ACAGCGTCTGGAATTCGTGC	NM_004431.3	c.1563-1582			
FLIcDNA-F	GGATCCAGGGAGTCTCCGGT	NM_002017.4	c.223-242	NM_002017, NM_001271010, NM_001167681	192 bp	60, Max
FLIcDNA-R	CACATGCTCCTGTGTCCACAGT	NM_002017.4	c.393-414			
EFNA1cDNA-F	CCACACCGTCTTCTGGAACA	NM_004428.2	c.59-79	NM_004428, NM_182685	213 bp	60, Max
EFNA1cDNA-R	GCCAGCGGACTTGGTCCT	NM_004428.2	c.254-271			
ANPEPcDNA-F	CCAAAATCAGACCAGATTGGC	NM_001150.2	c. 1015-1035	NM_001150	209 bp	60, Max
ANPEPcDNA-R	CACAGGTCATTCCACCACTCTA	NM_001150.2	c. 1202-1223			
CAV1cDNA-F	GTGGTCAAGATTGACTTTGAAGAT	NM_001753.4	c. 187-210	NM_001753, NM_001172896, NM_001172897, NM_001172895	249 bp	60, Max
CAV1cDNA-R	GCTGATGCACTGAATCTCAATCA	NM_001753.4	c.413-435			
METcDNA-F	CTGGGAAGAAGATCACGAAGAT	NM_001127500 .1	c. 1517-1538	NM_001127500, NM_000245	128 bp	60, Max
METcDNA-R	CGATCGCACACATTTGTCGT	NM_001127500 .1	c. 1625-1644			
FH1_CCND1[A]	GCCTCTAAGATGAAGGAGAC	NM_053056.2	c.328-347	NM_053056	94 bp	60, Max
RH1_CCND1[A]	CCATTTGCAGCAGCTC	NM_053056.2	c.406-421			
FH3_EGFR[A]	TCTTAAAGACCATCCAGGAG	NM_005228.3	c.233-252	NM_005228.3, NM_201282.1, NM_201283.1, NM_201284.1	85 bp	60, Max
RH_EGFR[A]	ATCTGCAGGTTTTCCAAAG	NM_005228.3	c.299-317			
KRAScDNA	GATCCAACAATAGAGGATTCCTACAG	NM_004985.3	c.97-122	NM_004985, NM_033360	82 bp	60, Max
KRAScDNA-R	CTGCTGTGTCGAGAATATCCAAG	NM_004985.3	c.156-178			

MITFcDNA-F	CCCAAGTCAAATGATCCAGAC	NM_198159.2, NM_000248.3	c.997-1017 c.694-714	NM_198159, NM_198177, NM_006722, NM_000248, NM_198158, NM_198178, NM_001184967	129 bp	60, Max
MITFcDNA-R	CAGTTTCTTCTGTCGGTTTTCAAGT	NM_198159.2, NM_000248.3	c.1104-1125 c.801-822			
RRAScDNA-F	CGCTGACCATCCAGTTCAT	NM_006270.4	c.131-149	NM_006270	124 bp	60, Max
RRAScDNA-R	GCGGTGTCCAGGATGTCCA	NM_006270.4	c. 236-254			
SOX10cDNA-F	GCTCTGGAGGCTGCTGA	NM_006941.3	c.420-436	NM_006941	207 bp	60, Max
SOX10cDNA-R	CTCTTGTAGTGGGCCTGGAT	NM_006941.3	c.607-626			
MAP3K8cDNA-F	CTGGAATTTTATTAAACATGGTCATCACT	NM_005204.3	c.317-345	NM_005204, NM_001244134	181 bp	60, Max
MAP3K8cDNA-R	CACGCCATTCTTTTCTTCGTCT	NM_005204.3	c.476-497			
HPRT1cDNA-F	GACACTGGCAAAACAATGCAGAC	NM_000194.2	c.412-434	NM_000194	93 bp	60, Max
HPRT1cDNA-R	GGTCCTTTTCACCAGCAAGCT	NM_000194.2	c.484-504			
HBMScDNA-F	ACAACCGGGTGGGGCAGA	NM_000190.3	c.596-613	NM_000190, NM_001024382	111 bp	60, Max
HBMScDNA-R	CCACCAGATCCAAGATGTCCTG	NM_000190.3	c.685-706			

A Commercial expression primer sequences from SigmaAldrich.

PCR method is given as annealing temperature, additives BE=betaine 1 M final concentration, NA=no additives, PCRx= PCRx Enhancer system, ThermoFisher Scientific), DM=DMSO 5% final concentration, Max=Maxima SYBR Green qPCR Master Mix, ThermoFisher Scientific.

BRAF mutational analyses on genomic DNA were performed using primers published in Davies H. et al. (2002) Nature 417(6892):949-954.
