# Supplementary material for: Silencing FLI or targeting CD13/ANPEP lead to dephosphorylation of EPHA2, a mediator of BRAF inhibitor resistance, and induce growth arrest or apoptosis in melanoma cells
Source: Cell Death Dis. 2017 Aug 31;8(8):e3029–. doi: 10.1038/cddis.2017.406 (PMC5596587; doi:10.1038/cddis.2017.406)
Supplement: Supplementary Figures [file cddis2017406x7.ppt]

## Slide 1
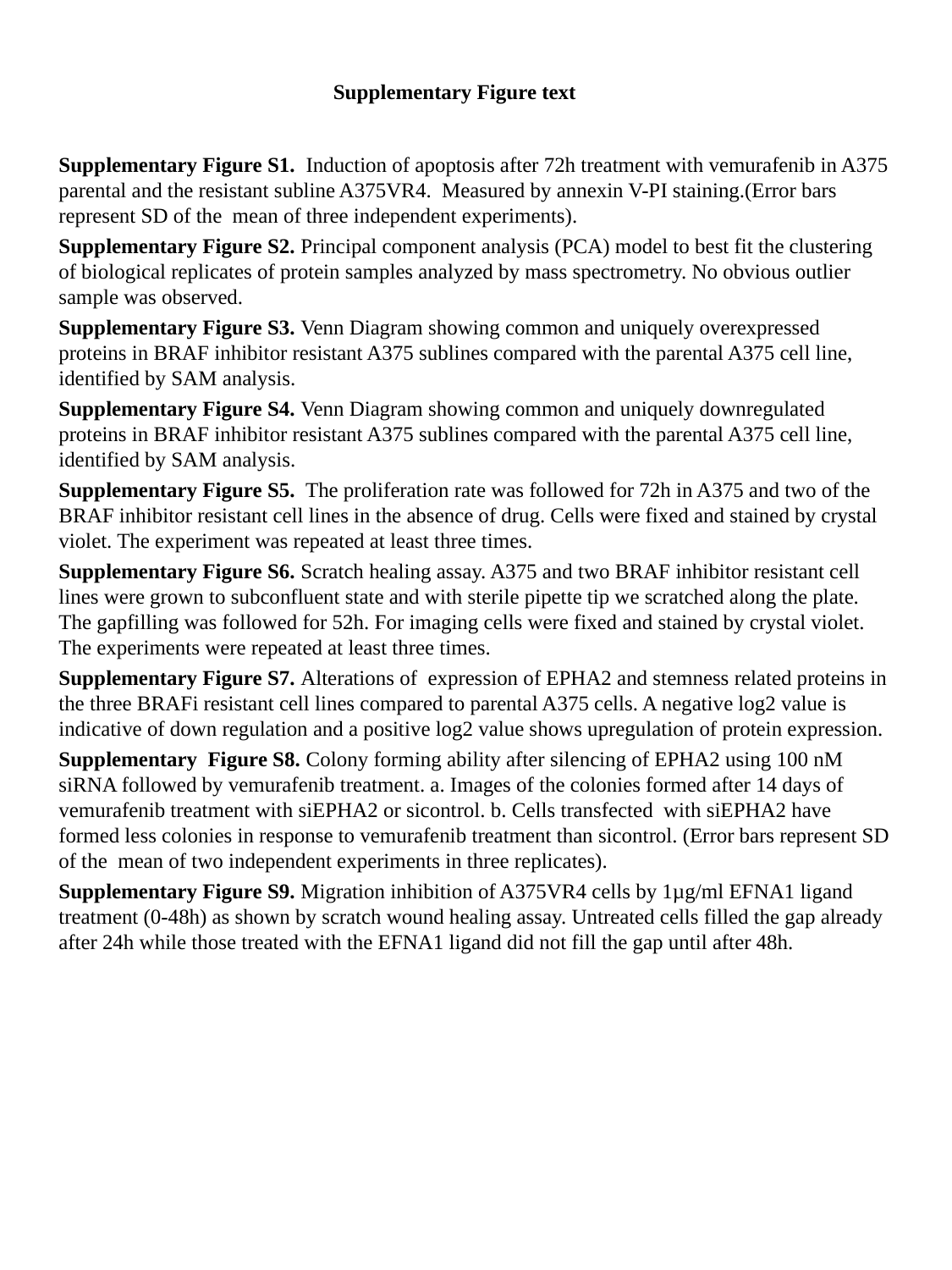

# Supplementary Figure text
Supplementary Figure S1. Induction of apoptosis after 72h treatment with vemurafenib in A375 parental and the resistant subline A375VR4. Measured by annexin V-PI staining.(Error bars represent SD of the mean of three independent experiments).
Supplementary Figure S2. Principal component analysis (PCA) model to best fit the clustering of biological replicates of protein samples analyzed by mass spectrometry. No obvious outlier sample was observed.
Supplementary Figure S3. Venn Diagram showing common and uniquely overexpressed proteins in BRAF inhibitor resistant A375 sublines compared with the parental A375 cell line, identified by SAM analysis.
Supplementary Figure S4. Venn Diagram showing common and uniquely downregulated proteins in BRAF inhibitor resistant A375 sublines compared with the parental A375 cell line, identified by SAM analysis.
Supplementary Figure S5. The proliferation rate was followed for 72h in A375 and two of the BRAF inhibitor resistant cell lines in the absence of drug. Cells were fixed and stained by crystal violet. The experiment was repeated at least three times.
Supplementary Figure S6. Scratch healing assay. A375 and two BRAF inhibitor resistant cell lines were grown to subconfluent state and with sterile pipette tip we scratched along the plate. The gapfilling was followed for 52h. For imaging cells were fixed and stained by crystal violet. The experiments were repeated at least three times.
Supplementary Figure S7. Alterations of expression of EPHA2 and stemness related proteins in the three BRAFi resistant cell lines compared to parental A375 cells. A negative log2 value is indicative of down regulation and a positive log2 value shows upregulation of protein expression.
Supplementary Figure S8. Colony forming ability after silencing of EPHA2 using 100 nM siRNA followed by vemurafenib treatment. a. Images of the colonies formed after 14 days of vemurafenib treatment with siEPHA2 or sicontrol. b. Cells transfected with siEPHA2 have formed less colonies in response to vemurafenib treatment than sicontrol. (Error bars represent SD of the mean of two independent experiments in three replicates).
Supplementary Figure S9. Migration inhibition of A375VR4 cells by 1µg/ml EFNA1 ligand treatment (0-48h) as shown by scratch wound healing assay. Untreated cells filled the gap already after 24h while those treated with the EFNA1 ligand did not fill the gap until after 48h.

## Slide 2
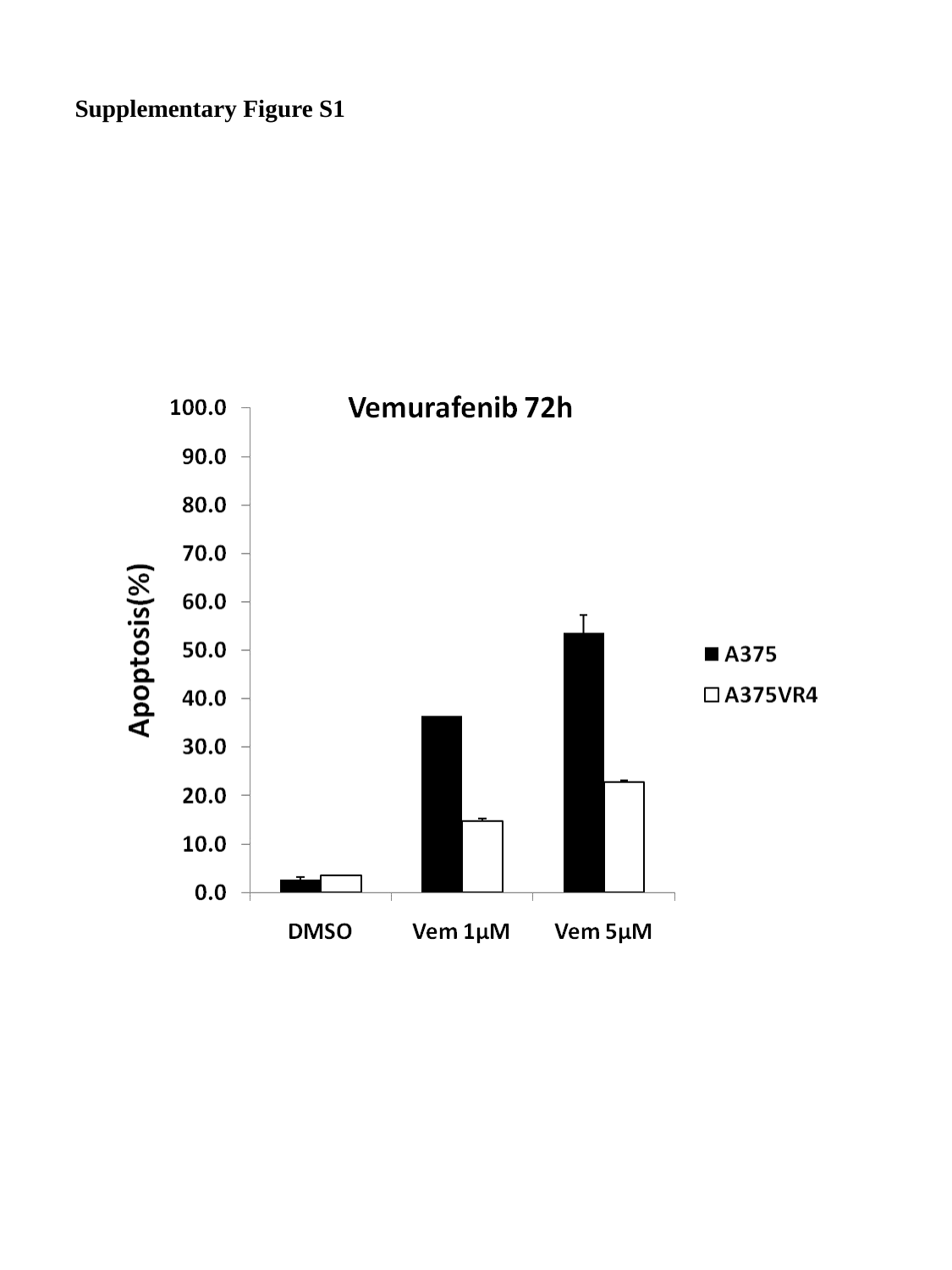

# Supplementary Figure S1

## Slide 3
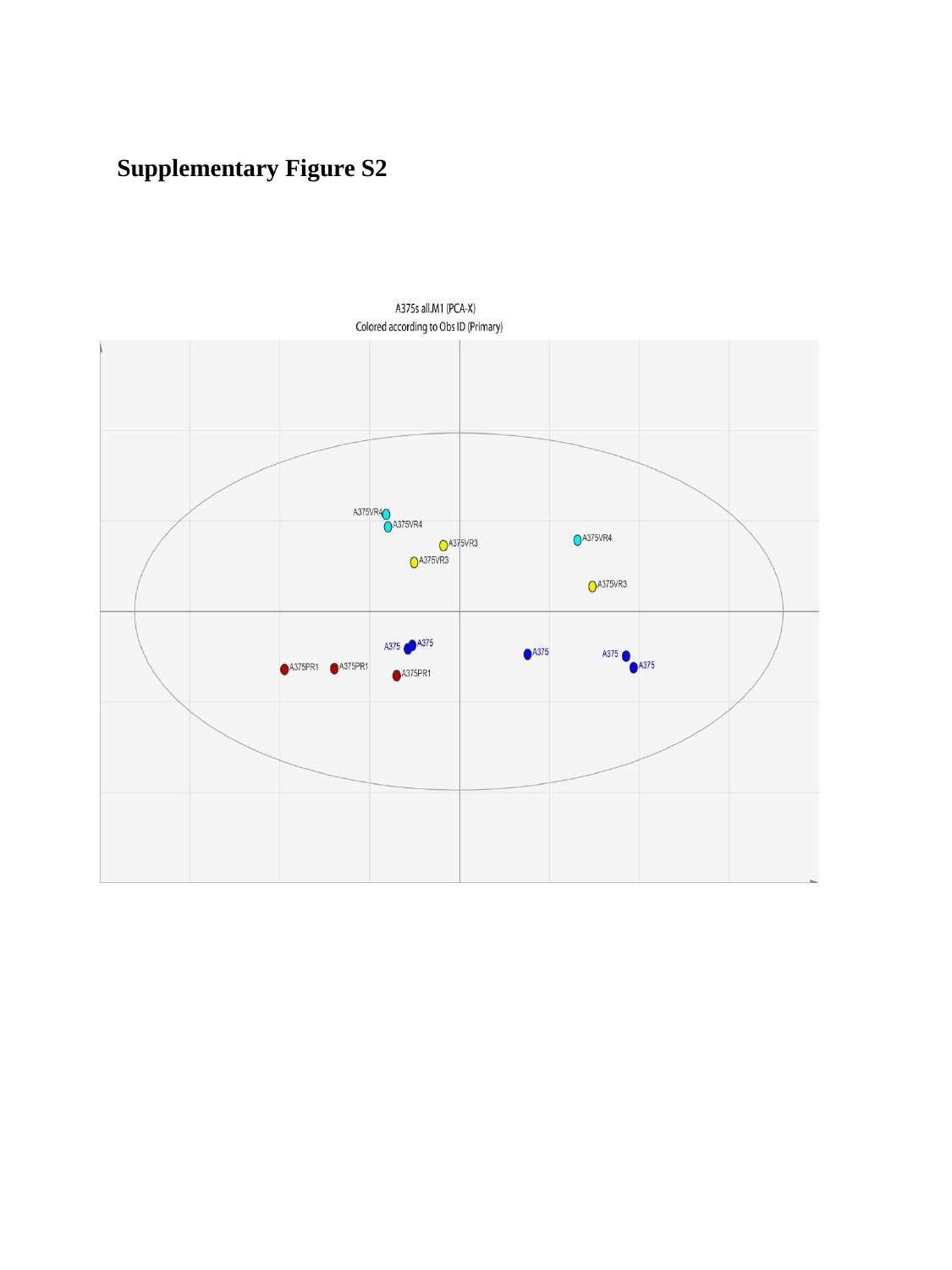

# Supplementary Figure S2

## Slide 4
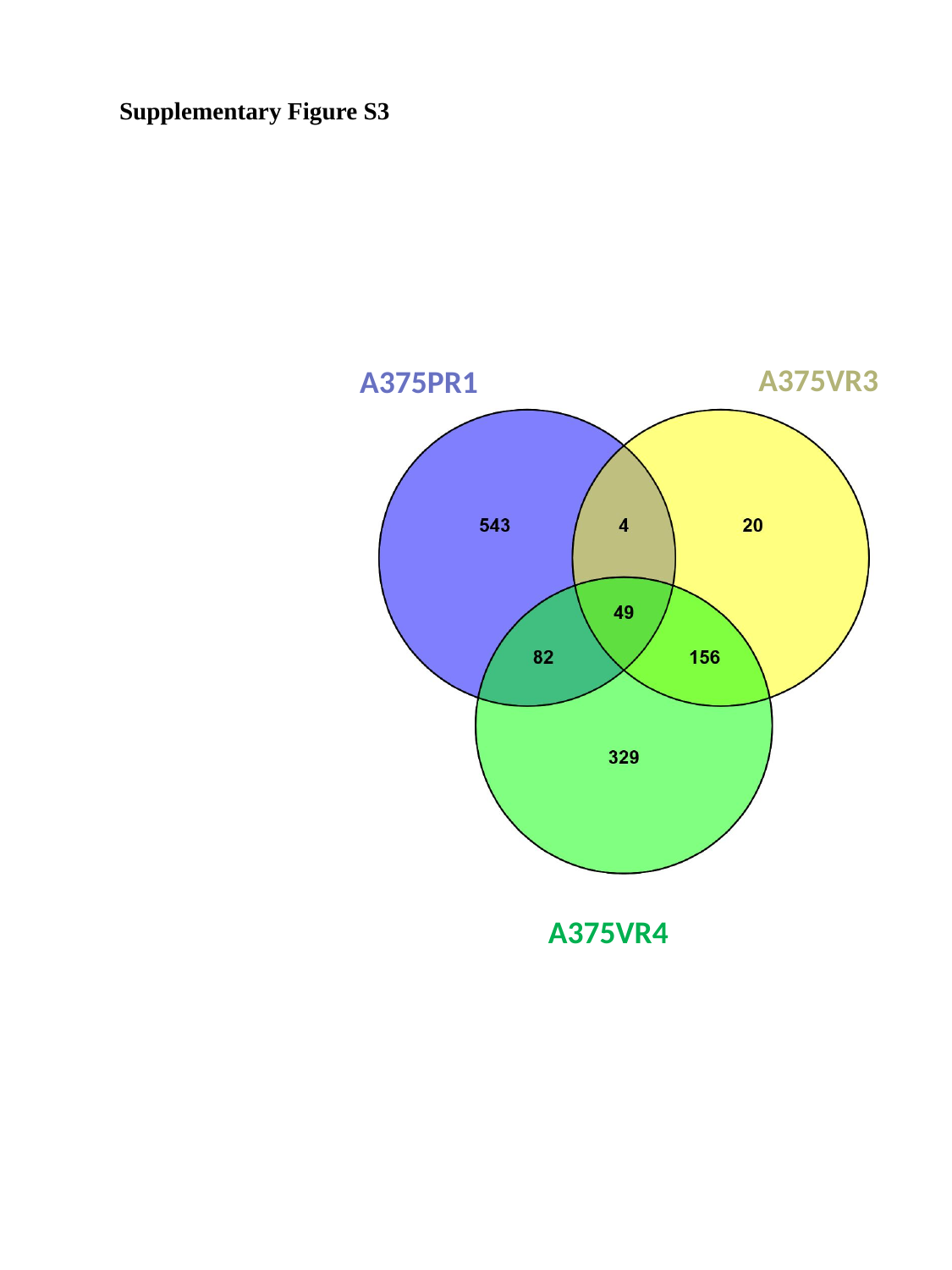

# Supplementary Figure S3
A375VR3
A375PR1
A375VR4

## Slide 5
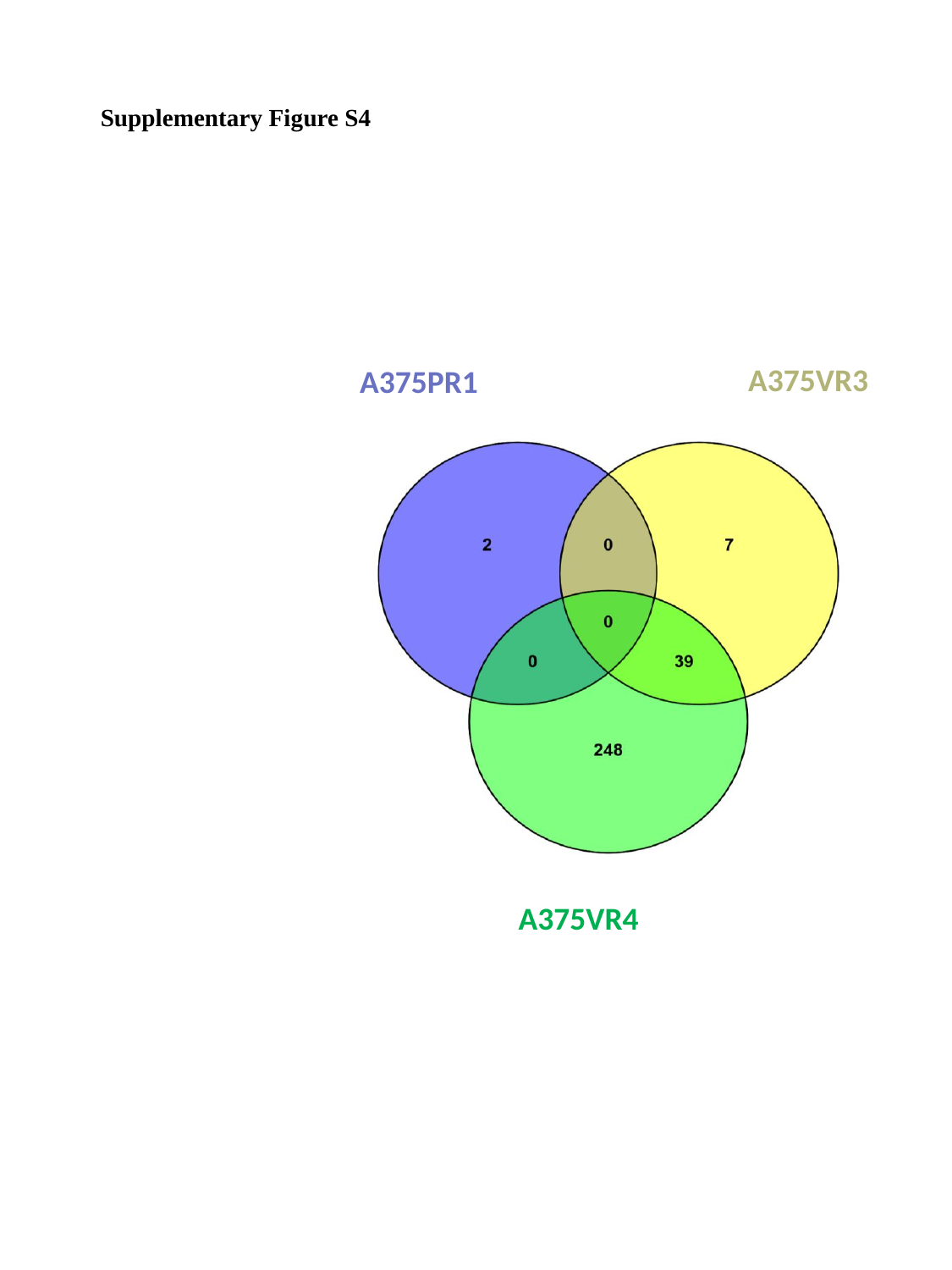

# Supplementary Figure S4
A375VR3
A375PR1
A375VR4

## Slide 6
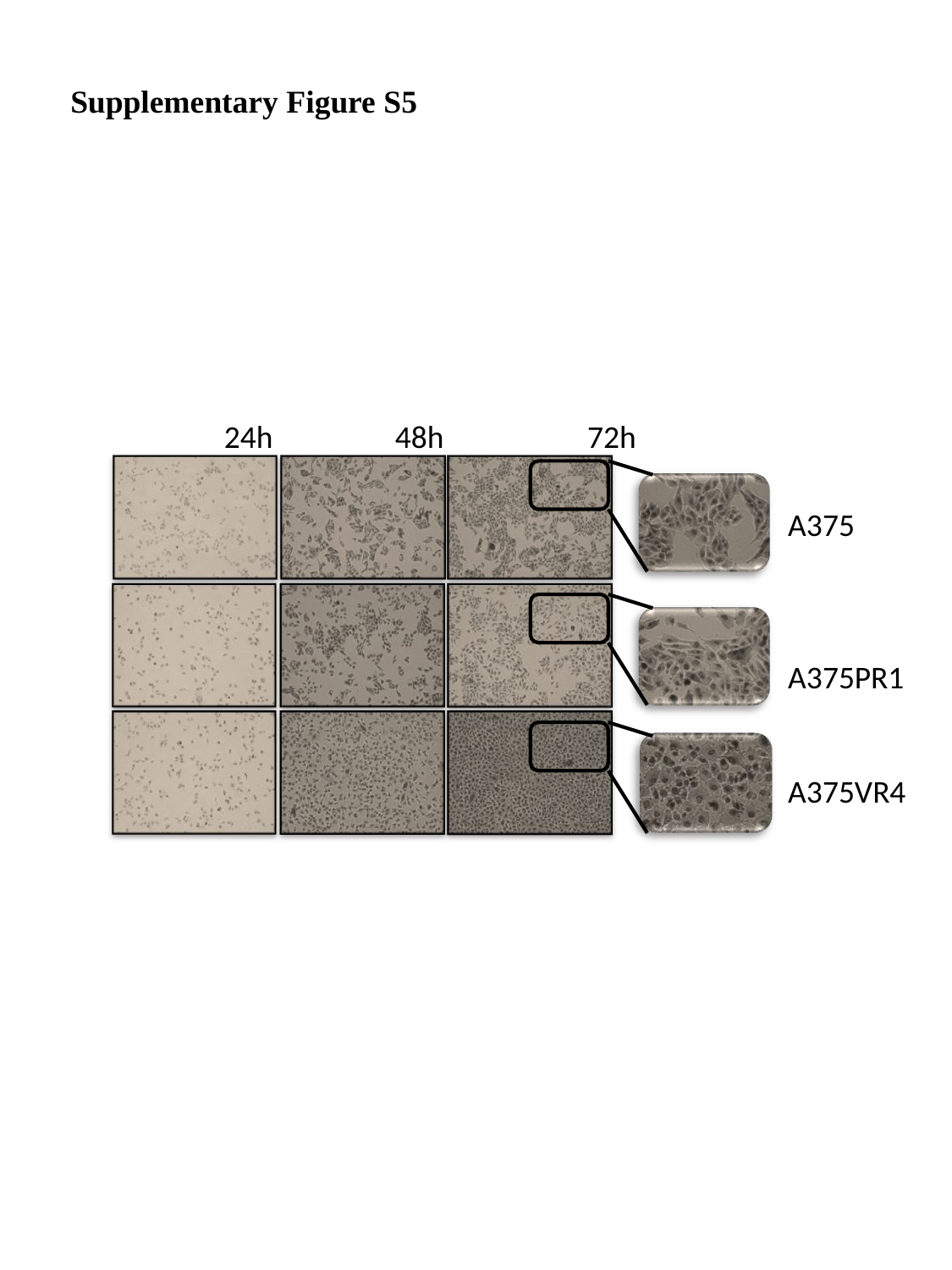

Supplementary Figure S5
24h 48h 72h
A375
A375PR1
A375VR4

## Slide 7
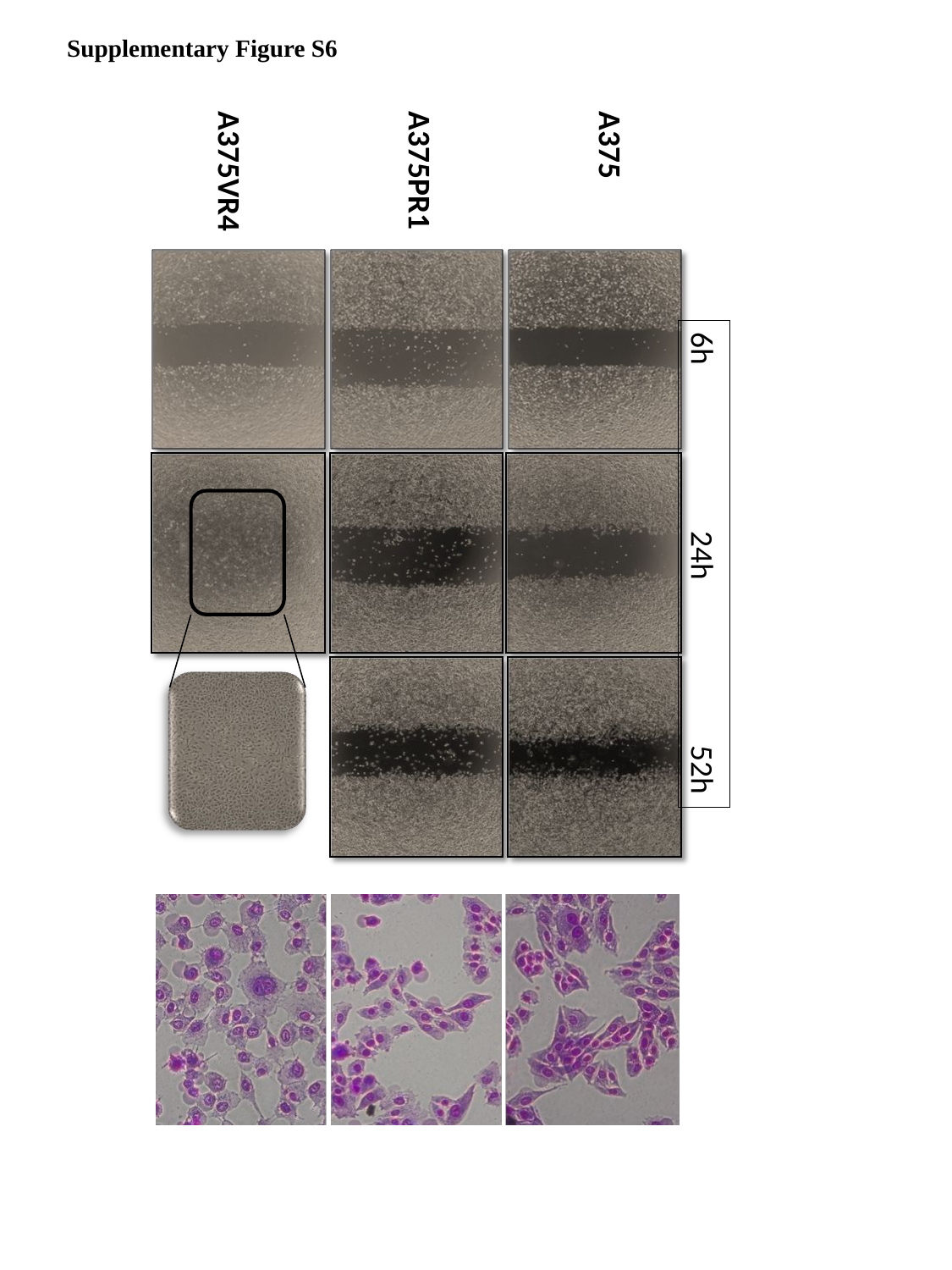

A375
A375PR1
A375VR4
Supplementary Figure S6
6h	 24h 52h

## Slide 8
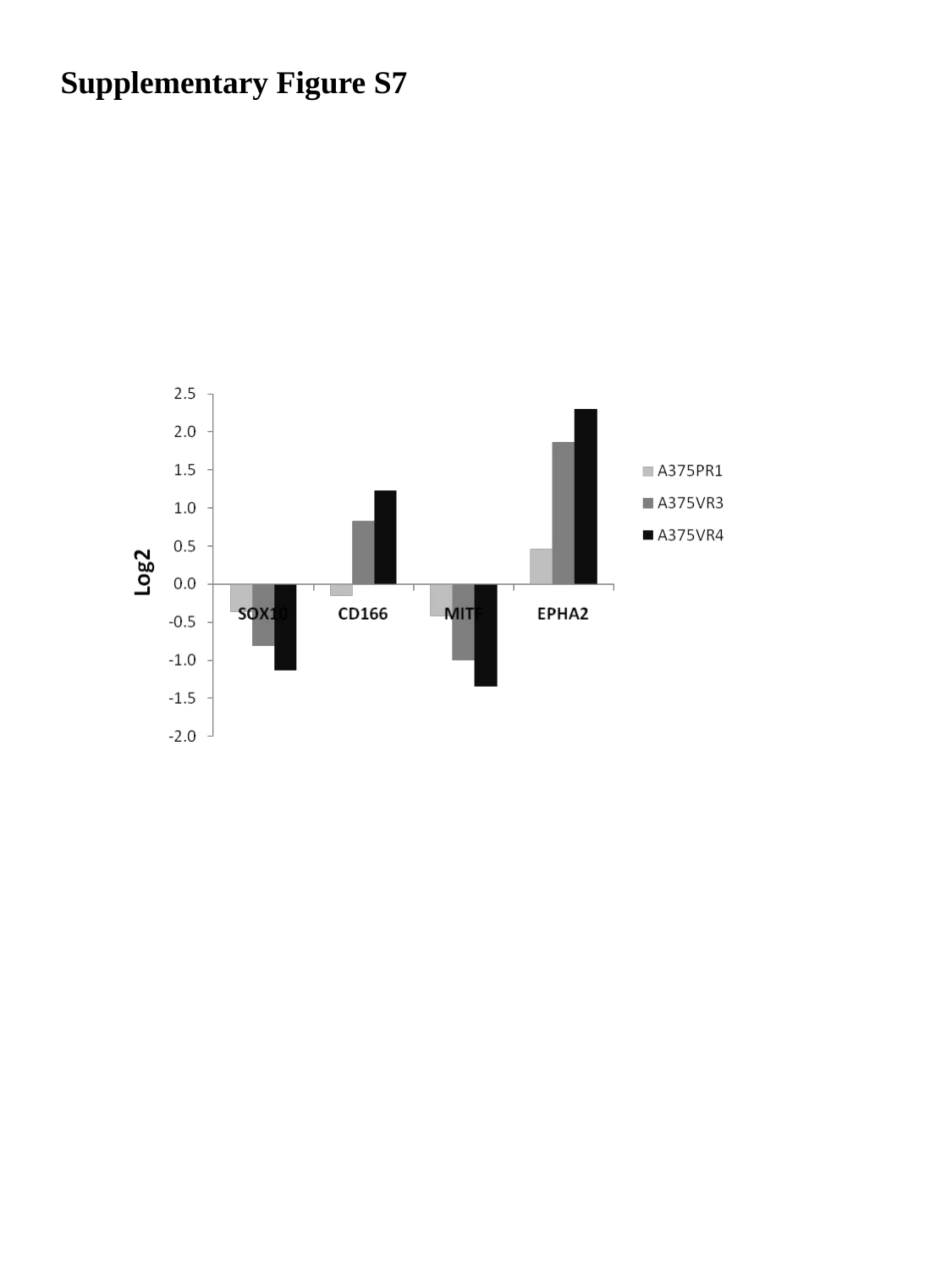

Supplementary Figure S7

## Slide 9
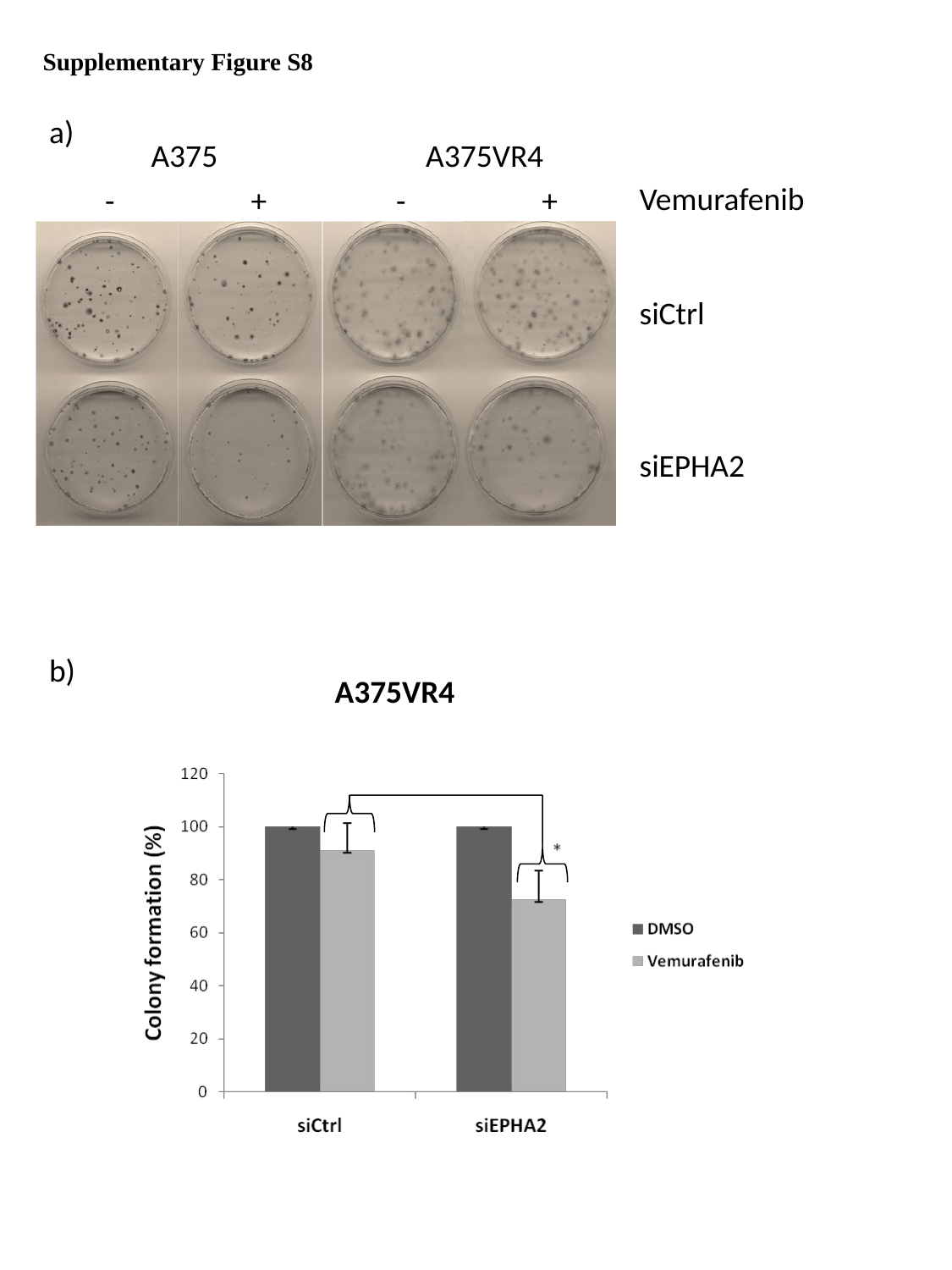

# Supplementary Figure S8
a)
 A375 A375VR4
Vemurafenib
siCtrl
siEPHA2
 - + - +
b)
A375VR4

## Slide 10
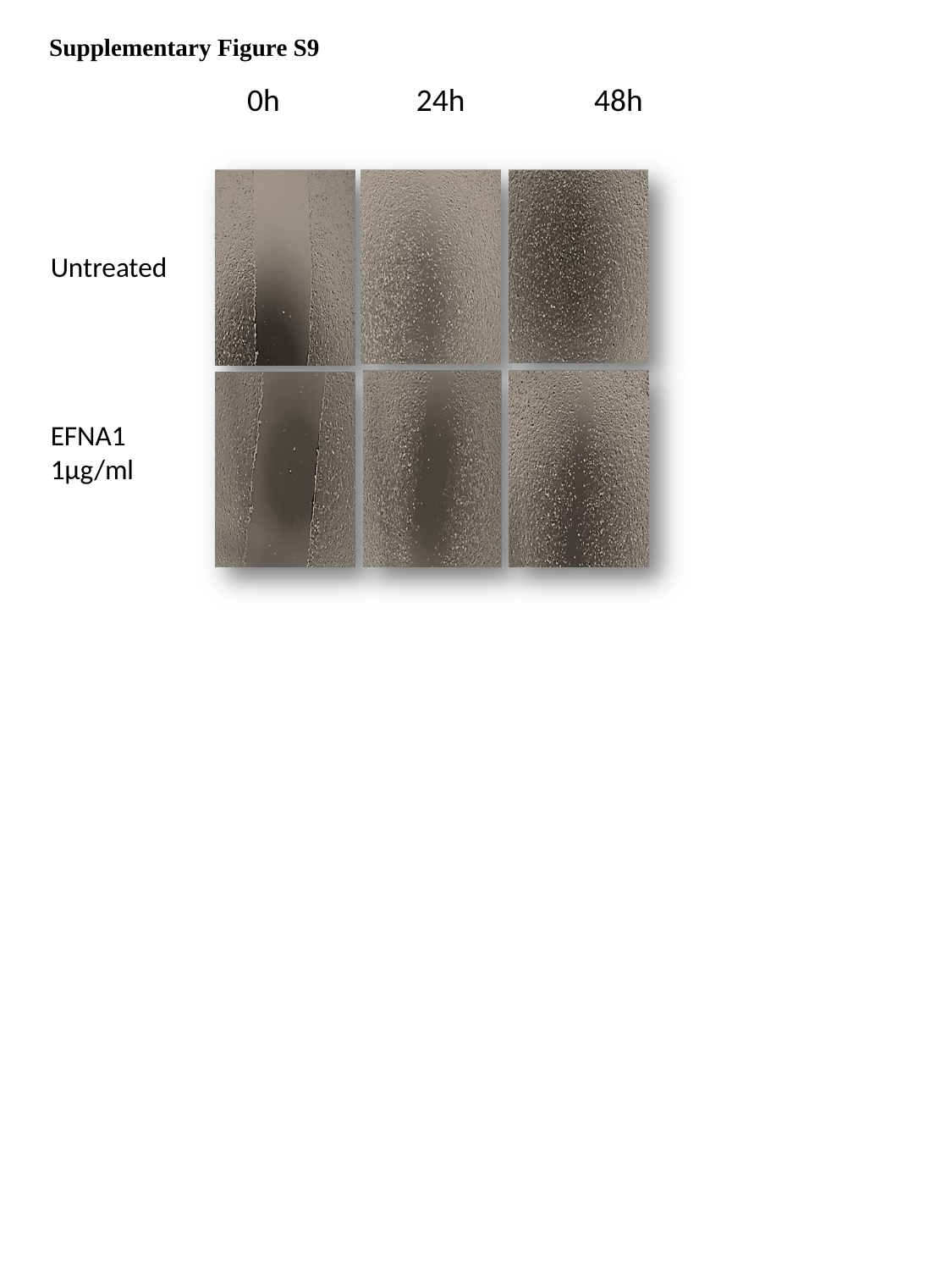

# Supplementary Figure S9
 0h 24h 48h
Untreated
EFNA1
1µg/ml
